# Supplementary material for: Modular access to alkylgermanes via reductive germylative alkylation of activated olefins under nickel catalysis
Source: Nat Commun. 2023 Nov 23;14:7669. doi: 10.1038/s41467-023-43561-z (PMC10667229; doi:10.1038/s41467-023-43561-z)
Supplement: Supplementary file 1 — Supplementary Information [file 41467_2023_43561_MOESM1_ESM.pdf]

Supplementary Information

Modular Access to Alkylgermanes via Reductive Germylative Alkylation of Activated Olefins Under Nickel Catalysis

Rui Gu,[1,2] Xiujuan Feng,[2] Ming Bao[2*] and Xuan Zhang[1,2*]

[1]School of Chemistry and Materials Science, Institute of Advanced Materials and Flexible Electronics (IAMFE), Nanjing University of Information Science and Technology, 219 Ningliu Road, Nanjing 210044, China.

[2]State Key Laboratory of Fine Chemicals, School of Chemical Engineering, Dalian University of Technology, 2 Linggong Road, Dalian 116024, China

E-mail: mingbao@dlut.edu.cn; xuanzhang@nuist.edu.cn

Table of Contents

1. Supplementary Methods S2

2. Optimization of Reaction Conditions S3

2.1 Optimization of Reaction Conditions S3

3. Supplementary Notes.. S6

3.1 Synthesis of Substrates.. S6

3.2 Experimental Procedures and Characterization of Products S14

3.3. Gram-scale Reactions and Synthetic Applications............. S49

3.4 Mechanistic Studies S54

3.5 Copies of NMR Spectra.. S57

4. Supplementary References... S140

1. Supplementary Methods

All reactions were carried out under an atmosphere of nitrogen in vial equipped with magnetic stirring. Anhydrous *N*, *N*-Dimethylacetamide (DMA), *N*, *N*-dimethylformamide (DMF), *N*-methylpyrrolidone (NMP), CH_3CN, EtOH, Dichloroethane (DCE), Ethyl acetate (EA), Dimethyl sulfoxide (DMSO), Tetrahydrofuran (THF) and Acetone were purchased from J&K Scientific and Adamas-beta.

Nickel catalysts, ligands, manganese powder and substrates were purchased from Adamas-beta, TCI, Strem, Alfa Aesar, Acros Organics, Energy Chemical, Bidepharm, SCRC, J&K Scientific, Macklin, or synthesized according to the procedures outlined below. Unless otherwise noted, materials obtained from commercial suppliers were used without further purification.

^{1}H NMR, ^{13}C NMR and ^{19}F NMR spectra were collected on JEOL JNM-ECS 400M spectrometer, Bruker Avance II 400 and Vaian DLG400 at room temperature carried out in deuterochloroform ($CDCl_3$). ^{1}H NMR spectra were reported in parts per million (ppm) and were referenced to the signal of $CHCl_3$ (7.26 ppm). Data were reported as follows: chemical shift (ppm), multiplicity (s = singlet, d = doublet, t = triplet, q = quartet, dd = doublet of doublets, m = multiplet), coupling constant (Hz), integration and assignment. ^{13}C NMR spectra were reported in ppm relative to residual $CHCl_3$ (77.00 ppm). Coupling constants, J, are reported in hertz (Hz). High resolution mass spectrometry (HRMS) were recorded on Bruker MTQ III q-TOF (ESI), Thermo Q Exactive Plus, Thermo Exactive GC. Melting points were uncorrected. GC spectra were performed on Shimadzu QP2010 (EI Source). LC spectra were performed on Agilent 1100 and Agilent HP1100. 3-(4,5-Dimethylthiazol-2-yl)-2,5-diphenyltetrazolium bromide (MTT) assay were performed on Tecan Austria GMBH, A-5082.

Thin layer chromatography was carried out using JIANGYOU HSGF254 TLC plates. Flash chromatography was performed using TITAN silica gel (200-300 mesh). TLC plates were analyzed by an exposure to ultraviolet (UV) light and/or submersion in $KMnO_4$ solution/water.

2. Optimization of Reaction Conditions

2.1 Optimization of Reaction Conditions

Supplementary Table 1: Screening of ligands.[a,b]

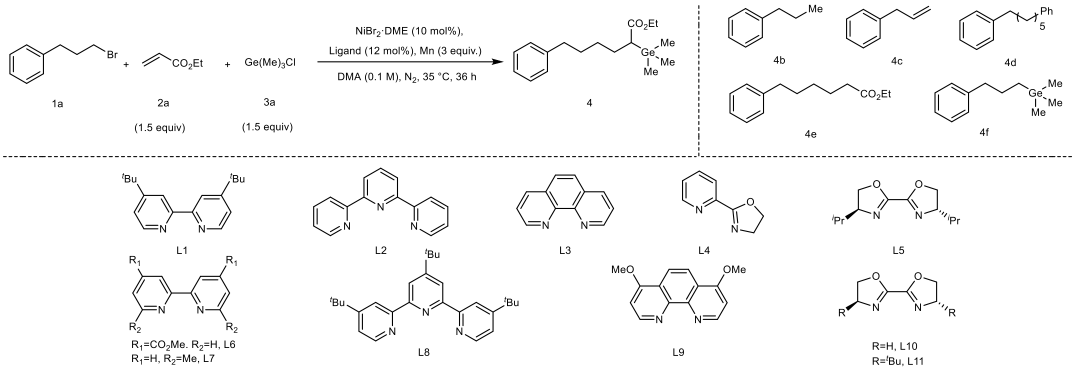

Entry	Ligand	T.M. (%)	S.M. (%)	Yield of 4b (%)	Yield of 4c (%)	Yield of 4d (%)	Yield of 4e (%)	Yield of 4f (%)
1	L1	15	41	3	4	0	21	0
2	L2	4	1	1	10	40	0	3
3	L3	19	57	7	4	5	4	Trace
4	L4	3	62	6	4	3	2	0
**5**	**L5**	**35**	**50**	**8**	**6**	**0**	**1**	**1**
6	L6	14	Trace	13	5	16	1	2
7	L7	8	Trace	5	3	0	Trace	0
8	L8	5	3	3	2	44	1	0
9	L9	13	33	2	3	20	1	2
10	L10	5	26	6	3	6	5	0
11	L11	30	48	10	10	0	1	0

[a] All reactions were performed on a 0.1 mmol scale. [b] Yield was determined by GC-FID analysis with dodecane (22.6 μL, 0.1 mmol) as an internal standard.

Supplementary Table 2: Screening of nickel catalysts.[a,b]

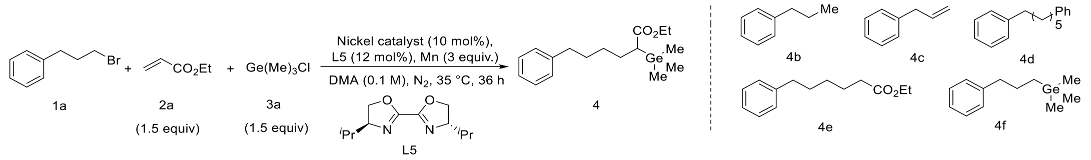

Entry	Nickel catalyst	T.M. (%)	S.M. (%)	Yield of 4b (%)	Yield of 4c (%)	Yield of 4d (%)	Yield of 4e (%)	Yield of 4f (%)
1	$NiBr_2(DME)$	45	50	8	6	0	1	1
**2**	**$NiBr_2$**	**73**	**7**	**8**	**6**	**1**	**2**	**1**
3	$NiCl_2$	39	34	6	9	1	1	1
4	NiI_2	58	trace	12	13	4	2	1
5	$Ni(COD)_2$	37	49	6	6	0	1	trace
6	$Ni(acac)_2$	30	50	7	6	trace	3.8	trace
7	$Ni(OTf)_2$	9	76	3	5	0	1	0
8	$NiCl_2(PPh_3)_2$	5	87	2	2	0	trace	0

[a] All reactions were performed on a 0.1 mmol scale. [b] Yield was determined by GC-FID analysis with dodecane (22.6 μL, 0.1 mmol) as an internal standard.

Supplementary Table 3: Screening of ligand 5.[a,b]

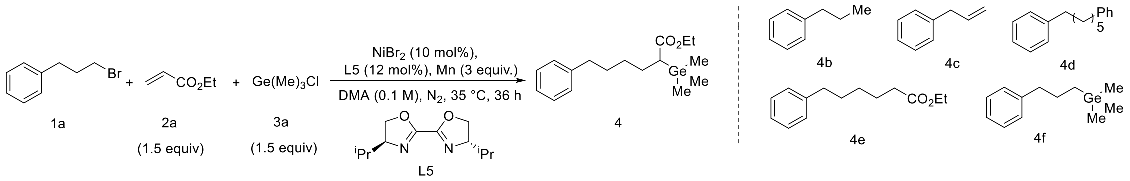

Entry	Ligand	T.M. (%)	S.M. (%)	Yield of 4b (%)	Yield of 4c (%)	Yield of 4d (%)	Yield of 4e (%)	Yield of 4f (%)
**1**	**Without Ligand**	**86**	**2**	**5**	**9**	**0**	**1**	**/**
2	With Ligand	73	7	8	6	1	2	1

[a] All reactions were performed on a 0.1 mmol scale. [b] Yield was determined by GC-FID analysis with dodecane (22.6 μL, 0.1 mmol) as an internal standard.

Supplementary Table 4: Screening of solvent.[a,b]

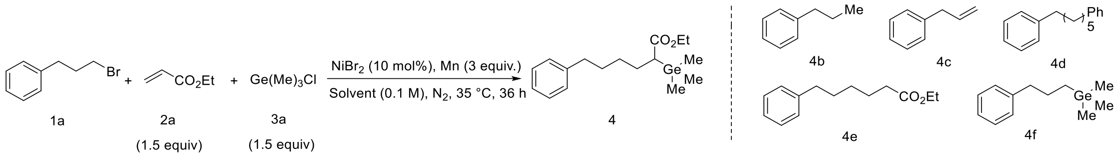

Entry	Solvent	T.M. (%)	S.M. (%)	Yield of 4b (%)	Yield of 4c (%)	Yield of 4d (%)	Yield of 4e (%)	Yield of 4f (%)
**1**	**DMA**	**86**	**2**	**5**	**9**	**0**	**1**	**0**
2	DMF	58	25	4	3	0	1	1
3	NMP	31	44	7	5	0	3	0
4	DMSO	trace	37	2	0	0	0	0
5	CH_3CN	5	91	10	3	3	7	0
6	1,4-Dioxane	0	107	0	0	0	0	0
7	THF	28	0	23	6	1	8	0
8	DME	16	69	6	4	0	2	0
9	EA	0	100	0	0	0	0	0
10	CH_3OH	0	100	0	0	0	0	0

[a] All reactions were performed on a 0.1 mmol scale. [b] Yield was determined by GC-FID analysis with dodecane (22.6 μL, 0.1 mmol) as an internal standard.

Supplementary Table 5: Screening of reductant.[a,b]

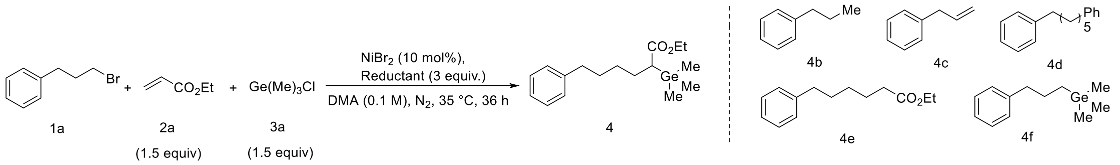

Entry	Reductant	T.M. (%)	S.M. (%)	Yield of 4b (%)	Yield of 4c (%)	Yield of 4d (%)	Yield of 4e (%)	Yield of 4f (%)
**1**	**Mn**	**86**	**2**	**5**	**9**	**0**	**1**	**0**
2	Zn	6	88	6	4	0	1	1
3	Mg	trace	trace	23	2	12	1	0
4	TDAE	1	43	0	2	4	1	0

[a] All reactions were performed on a 0.1 mmol scale. [b] Yield was determined by GC-FID analysis with dodecane (22.6 μL, 0.1 mmol) as an internal standard. [c] TDAE: Tetrakis(dimethylamino)ethylene

Supplementary Table 6: Screening of catalysts and reductant equivalence ratio.[a,b]

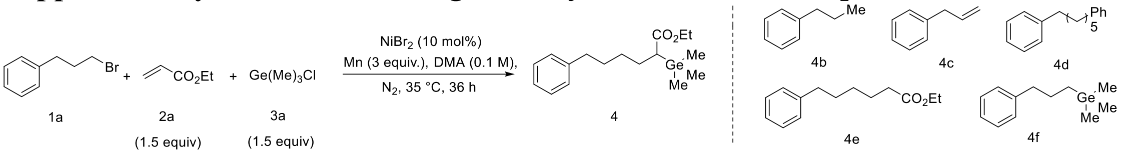

Entry	Nickel catalyst	Reductant	T.M. (%)	S.M. (%)	Yield of 4b (%)	Yield of 4c (%)	Yield of 4d (%)	Yield of 4e (%)	Yield of 4f (%)
1	$NiBr_2$ (5 mol %)	Mn (3 equiv)	53	25	4	7	0	1	1
**2**	**$NiBr_2$ (10 mol %)**	**Mn (3 equiv)**	**86**	**2**	**5**	**9**	**0**	**1**	**0**
3	$NiBr_2$ (15 mol %)	Mn (3 equiv)	77	0	6	11	0	2	1
4	$NiBr_2$ (10 mol %)	Mn (2 equiv)	62	17	5	12	0	1	0
5	$NiBr_2$ (10 mol %)	Mn (4 equiv)	82	3	5	9	0	1	1

[a] All reactions were performed on a 0.1 mmol scale. [b] Yield was determined by GC-FID analysis with dodecane (22.6 μL, 0.1 mmol) as an internal standard.

Supplementary Table 7: Screening of alkene and germanium reagent equivalence ratio.[a,b]

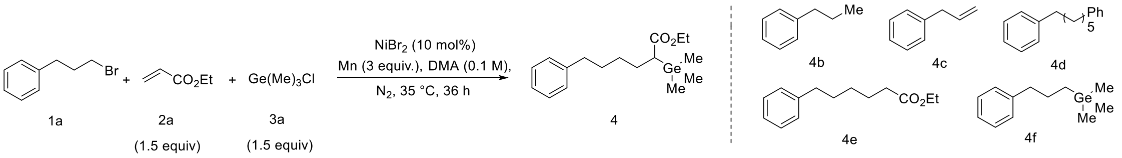

Entry	Reductant	T.M. (%)	S.M. (%)	Yield of 4b (%)	Yield of 4c (%)	Yield of 4d (%)	Yield of 4e (%)	Yield of 4f (%)
1	Alkene (1.2 equiv) Ge reagent (1.2 equiv)	60	**trace**	**10**	**14**	**0**	**4**	**trace**
2	Alkene (1.5 equiv) Ge reagent (1.2 equiv)	77	1	5	9	0	1	1
3	Alkene (1.2 equiv) Ge reagent (1.5 equiv)	71	1	9	12	0	1	1
**4**	**Alkene (1.5 equiv) Ge reagent (1.5 equiv)**	**86**	**0**	**5**	**9**	**0**	**1**	**0**
5	Alkene (2 equiv) Ge reagent (2 equiv)	83	1	4	8	0	1	1

[a] All reactions were performed on a 0.1 mmol scale. [b] Yield was determined by GC-FID analysis with dodecane (22.6 μL, 0.1 mmol) as an internal standard.

3. Supplementary Notes

3.1 Synthesis of Substrates

3.1.1 Synthesis of alkyl bromides

The alkyl bromides used in this paper:

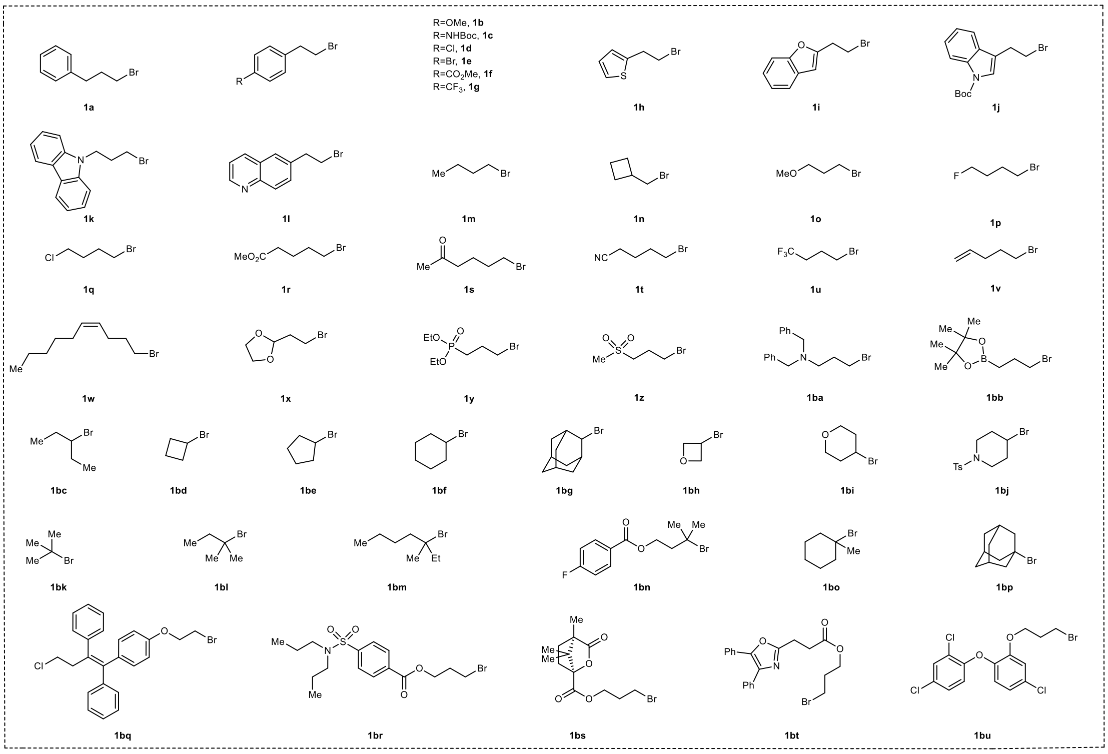

Alkyl bromides **1a-1b, 1d-1h, 1m-1bi, 1bk-bl, 1bp** are commercially available. **1c, 1i, 1j, 1k, 1l, 1bj, 1bm, 1bn, 1bo, 1br, 1bu** are known compounds, and they were synthesized according to the literature procedure.

The preparation of **1bq, 1bs** and **1bt**, and their characterization data are provided as follows.

Tert-butyl (4-(2-bromoethyl)phenyl)carbamate (1c)

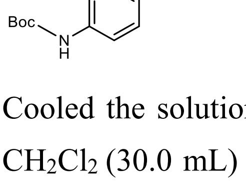

Cooled the solution of (4-(2-hydroxyethyl)phenyl)carbamate (0.948 g, 4.0 mmol) in CH_2Cl_2 (30.0 mL) to 0 °C, added CBr_4 (1.986 g, 6.0 mmol) to the resulting solution followed by portion-wise addition of PPh_3 (1.572 g, 6.0 mmol), the reaction mixture stirred for 30 minutes at 0 °C and warmed to room temperature and stirred for overnight. Removed half of the solvent in vacuo, added hexane to the mixture, filtered the resulting precipitate through Celite, purified by flash chromatography (Petroleum ether: EtOAc = 5: 1) to provide the title compound as a white solid (0.584 g, 49% yield). ^{1}H NMR

(400 MHz, $CDCl_3$) δ: 7.31 (d, J = 8.3 Hz, 2H), 7.13 (d, J = 8.3 Hz, 2H), 6.46 (s, 1H), 3.52 (t, J = 7.7 Hz, 2H), 3.10 (t, J = 7.6 Hz, 2H), 1.51 (s, 9H). All data are in accordance with the literature[1].

2-(2-bromoethyl)benzofuran (1i)

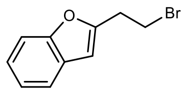

Cooled the solution of 2-(benzofuran-2-yl)ethan-1-ol (0.324 g, 2.0 mmol) in CH_2Cl_2 (15.0 mL) to 0 °C, added CBr_4 (1.016 g, 3.0 mmol) to the resulting solution followed by portion-wise addition of PPh_3 (0.787 g, 3.0 mmol), the reaction mixture stirred for 30 minutes at 0 °C and warmed to room temperature and stirred for 2 h. Removed half of the solvent in vacuo, added hexane to the mixture, filtered the resulting precipitate through Celite, purified by flash chromatography (Petroleum ether: EtOAc = 4: 1) to provide the title compound as a colorless oil (0.289 g, 65% yield). ^{1}H NMR (400 MHz, $CDCl_3$) δ: 7.65-7.51 (m, 2H), 7.39-7.28 (m, 2H), 6.61 (s, 1H), 3.75 (t, J = 7.2 Hz, 2H), 3.39 (t, J = 7.2 Hz, 2H). All data are in accordance with the literature[2].

Tert-butyl 3-(2-bromoethyl)-1*H*-indole-1-carboxylate (1j)

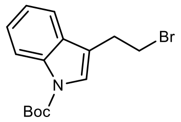

To a solution of 3-(2-bromoethyl)-1*H*-indole (1.0 g, 4.4 mmol), Et_3N (0.880 g, 8.8 mmol) and DMAP (0.120 g, 0.88 mmol) in CH_2Cl_2 (10.0 mL) at 0 °C, added $(Boc)_2O$ (1.030 g, 4.67 mmol) in CH_2Cl_2 (6.0 mL) over 30 minutes. After stirring for 1 h at room temperature, the reaction mixture was washed three times with water, dried over sodium sulfate and concentrated, purified by flash chromatography (Petroleum ether: EtOAc = 10: 1) to provide the title compound as a white solid (1.318 g, 91% yield). ^{1}H NMR (400 MHz, $CDCl_3$) δ: 8.14 (s, 1H), 7.54-7.44 (m, 2H), 7.36-7.29 (m, 1H), 7.26-7.21 (m, 1H), 3.63 (t, J = 7.6 Hz, 2H), 3.27 (t, J = 7.4 Hz, 2H), 1.66 (s, 9H). All data are in accordance with the literature[3].

9-(3-bromopropyl)-9H-carbazole (1k)

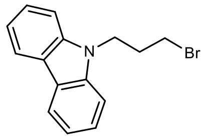

The 9*H*-carbazole (3.340 g, 20.0 mmol), 1,3-dibromopropane (6.1 mL, 60.0 mmol), NaOH (10.0 g, 25.0 mmol), tetrabutylammonium iodide (0.307 g, 0.83 mmol) were

dissolved in H_2O (10.0 mL), The mixture was stirred at room temperature for 6 h and quenched by addition of ice, the crude product was diluted with CH_2Cl_2 and washed with brine. The organic phase was dried anhydrous sodium sulfate and filtered, purified by flash chromatography (Petroleum ether: EtOAc = 3: 1) to provide the title compound as a white solid (1.830 g, 32% yield); ^{1}H NMR (400 MHz, $CDCl_3$) δ: 8.13 (d, *J* = 7.2 Hz, 2H), 7.50 (d, *J* = 3.6 Hz, 4H), 7.30-7.26 (m, 2H), 4.51 (t, *J* = 6.5 Hz, 2H), 3.40 (t, J = 6.1 Hz, 2H), 2.46 (q, J = 6.3 Hz, 2H). All data are in accordance with the literature[4].

6-(2-bromoethyl)quinoline (1l)

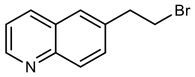

Cooled the solution of 2-(quinolin-6-yl)ethan-1-ol (0.519 g, 3.0 mmol) in THF (4.5 mL) to 0 °C, added CBr_4 (1.490 g, 4.5 mmol) to the resulting solution followed by portion-wise addition of PPh_3 (1.180 g, 4.5 mmol), the reaction mixture stirred for 30 minutes at 0 °C and warmed to room temperature and stirred for overnight. Removed half of the solvent in vacuo, added hexane to the mixture, filtered the resulting precipitate through Celite, purified by flash chromatography (Petroleum ether: EtOAc = 1: 1 with 3% Et_3N) to provide the title compound as a yellow liquid (0.456 g, 63% yield). ^{1}H NMR (400 MHz, $CDCl_3$) δ: 8.90 (dd, *J* = 4.2 Hz, 1.7 Hz, 1H), 8.13 (d, *J* = 8.3 Hz, 1H), 8.07 (d, *J* = 8.6 Hz, 1H), 7.66 (s, 1H), 7.58 (dd, *J* = 8.6 Hz, 2.0 Hz, 1H), 7.40 (dd, *J* = 8.3 Hz, 4.2 Hz, 1H), 3.68 (t, *J* = 7.4 Hz, 2H), 3.36 (t, *J* = 7.4 Hz, 2H). All data are in accordance with the literature[5].

4-bromo-1-tosylpiperidine (1bj)

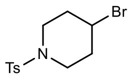

Added Et_3N (0.6 mL) to a solution of 4-bromopiperidine (0.490 g, 2.0 mmol) and *p*-toluenesulfonyl chloride (0.420 g, 2.2 mmol) in CH_2Cl_2 (20.0 mL) at 0 °C, the reaction mixture warmed to room temperature and stirred for overnight. Diluted the reaction mixture with water and brine, dried the resultant over anhydrous magnesium chloride, purified by flash chromatography (silica gel: 50% DCM in Petroleum ether) to provide the title compound as a white solid (0.341 g, 54% yield). ^{1}H NMR (400 MHz, $CDCl_3$) δ: 7.65 (d, *J* = 8.3 Hz, 2H), 7.34 (d, *J* = 8.0 Hz, 2H), 4.25 (dt, *J* = 6.7 Hz, 3.2 Hz, 1H), 3.23-3.17 (m, 2H), 3.13-3.07 (m, 2H), 2.45 (s, 3H), 2.23-2.15 (m, 2H), 2.09-2.01 (m, 2H). All data are in accordance with the literature[6].

3-bromo-3-methylheptane (1bm)

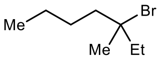

Added LiBr (0.274 g, 3.2 mmol) in 48 wt% aqueous HBr (2.1 mL) to a solution of 3-methylheptan-3-ol (0.273 g, 2.1 mmol) at 0 °C, the reaction mixture warmed to room temperature and stirred for overnight. Diluted the mixture with Et_2O, washed with water, saturated $NaHCO_3$ and brine. Dried over anhydrous magnesium chloride and concentrated to provide the title compound as a colorless oil (0.152 g, 37% yield). ^{1}H NMR (400 MHz, $CDCl_3$) δ: 1.92-1.75 (m, 4H), 1.70 (s, 3H), 1.49-1.40 (m, 2H), 1.34 (q, J = 8.0 Hz, 2H), 1.03 (t, J = 7.3 Hz, 3H), 0.93 (t, J = 7.2 Hz, 3H). All data are in accordance with the literature[7].

3-bromo-3-methylbutyl 4-fluorobenzoate (1bn)

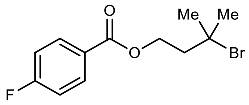

Added LiBr (1.106 g, 12.7 mmol) in 48 wt% aqueous HBr (8.5 mL) to a solution of 3-hydroxy-3-methylbutyl 4-fluorobenzoate (1.920 g, 8.49 mmol) at 0 °C, the reaction mixture warmed to room temperature and stirred for overnight. Diluted the mixture with Et_2O, washed with water, saturated $NaHCO_3$ and brine. Dried over anhydrous magnesium chloride and concentrated to provide the title compound as a yellow liquid (2.210 g, 90% yield). ^{1}H NMR (400 MHz, $CDCl_3$) δ: 8.05 (dd, J = 9.0 Hz, 5.4 Hz, 2H), 7.11 (t, J = 8.7 Hz, 2H), 4.58 (t, J = 6.7 Hz, 2H), 2.30 (t, J = 6.8 Hz, 2H), 1.85 (s, 6H). All data are in accordance with the literature[8].

1-bromo-1-methylcyclohexane (1bo)

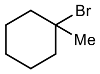

Added LiBr (1.302 g, 15.0 mmol) in 48 wt% aqueous HBr (10.0 mL) to a solution of 1-methylcyclohexan-1-ol (1.141g, 10.0 mmol) at 0 °C, the reaction mixture warmed to room temperature and stirred for overnight. Diluted the mixture with Et_2O, washed with water, saturated $NaHCO_3$ and brine. Dried over anhydrous magnesium chloride and concentrated to provide the title compound as a yellow liquid (0.740 g, 42% yield). ^{1}H NMR (400 MHz, $CDCl_3$) δ: 2.09 (dt, J = 14.3 Hz, 3.4 Hz, 2H), 1.83 (s, 3H), 1.76-1.57 (m, 5H), 1.50-1.43 (m, 2H), 1.26-1.17 (m, 1H). All data are in accordance with the literature[9].

(Z)-(1-(4-(2-bromoethoxy)phenyl)-4-chlorobut-1-ene-1,2-diyl)dibenzene (1bq)

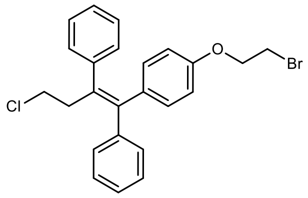

Cooled the solution of Ospemifen (0.76 g, 2.0 mmol) in CH_2Cl_2 (10.0 mL) to 0 °C, added CBr_4 (1.016 g, 3.0 mmol) in 2mL CH_2Cl_2 to the resulting solution followed by portion-wise addition of PPh_3 (0.787 g, 3.0 mmol), the reaction mixture stirred for 30 minutes at 0 °C and warmed to room temperature and stirred for 2 h. Removed half of the solvent in vacuo, added hexane to the mixture, filtered the resulting precipitate through Celite, purified by flash chromatography (Petroleum ether) to provide the title compound as a white solid (0.67 g, 76% yield). **^{1}H NMR (400 MHz, CDCl$_3$)** δ: 7.39-7.28 (m, 5H), 7.22-7.13 (m, 5H), 6.80 (d, J = 6.6 Hz, 2H), 6.56 (d, J = 8.2 Hz, 2H), 4.15 (t, J = 6.3 Hz, 2H). 3.55 (t, J = 6.3 Hz, 2H), 3.42 (t, J = 7.4 Hz, 2H), 2.92 (t, J = 7.4 Hz, 2H). **^{13}C NMR (100 MHz, CDCl$_3$)** δ:156.22, 142.73, 141.52, 140.82, 135.50, 135.17, 131.78, 129.50, 129.36, 128.35, 128.22, 126.97, 126.62, 113.58, 66.30, 42.37, 38.51, 27.96.

HRMS (ESI): $[M+H]^+$ calculated for $C_{24}H_{22}BrClO$ = 441.0616, found: 441.0618.

3-bromopropyl 4-(*N,N*-dipropylsulfamoyl)benzoate (1br)

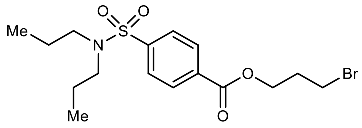

Mixed 4-(*N,N*-dipropylsulfamoyl)benzoic acid (1.141 g, 4.0 mmol) and K_2CO_3 (0.841 g, 6.1 mmol) in DMF (18.0 mL), added 1,3-dibromopropane (1.228 g, 6.1 mmol) to the mixture and stirred the mixture at room temperature for 12 h. Diluted the mixture with DCM, washed the mixture with water, separated the organic layer, dried the resultant over anhydrous sodium sulfate and concentrated the mixture under reduced pressure, purified by flash chromatography (Petroleum ether: EtOAc = 4: 1) to provide the title compound as a white solid (1.095 g, 68% yield). **^{1}H NMR (400 MHz, CDCl$_3$)** δ: 8.13 (d, J = 8.7 Hz, 2H), 7.86 (d, J = 8.3 Hz, 2H), 4.49 (t, J = 6.0 Hz, 2H), 3.54 (t, J = 6.5 Hz, 2H), 3.10-3.06 (m, 4H), 2.33 (p, J = 6.3 Hz, 2H), 1.57-1.48 (m, 4H), 0.85 (t, J = 7.4 Hz, 6H). All data are in accordance with the literature[10].

HRMS (ESI): $[M+H]^+$ calculated for $C_{16}H_{24}BrNO_4S$ = 406.0682, found: 406.0686.

3-bromopropyl (1S,4R)-4,7,7-trimethyl-3-oxo-2-oxabicyclo[2.2.1]heptane-1-carboxylate (1bs)

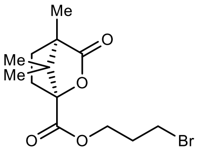

Mixed (1R)-(+)-Camphanic acid (0.99 g, 5.0 mmol) and K_2CO_3 (1.04 g, 7.5 mmol) in DMF (20.0 mL) to the mixture, dropwise added 1,3-dibromopropane (1.51 g, 7.5 mmol) at 0 ^oC and stirred the mixture at room temperature for 12 h. Diluted the mixture with DCM, washed the mixture with water, separated the organic layer, dried the resultant over anhydrous sodium sulfate and concentrated the mixture under reduced pressure, purified by flash chromatography (in Petroleum ether) to provide the title compound as clear colorless oil (1.03 g, 65% yield). **^{1}H NMR (400 MHz, $CDCl_3$)** δ: 4.40-4.35 (m, 2H), 3.48 (t, *J* = 6.4Hz, 2H), 2.47-2.40 (m, 1H), 2.26-2.22 (m, 2H), 2.07-.2.00 (m, 1H), 1.96-1.89 (m, 1H), 1.73-1.66 (m, 1H), 1.12 (s, 3H), 1.06 (s, 3H), 0.96 (s, 3H); **^{13}C NMR (100 MHz, $CDCl_3$)** δ: 178.13, 167.43, 91.04, 63.19, 54.76, 54.24, 31.21, 30.64, 29.07, 28.88, 16.71, 9.69.

3-bromopropyl 3-(4,5-diphenyloxazol-2-yl)propanoate (1bt)

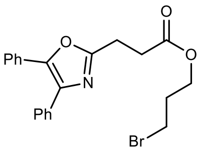

Mixed Oxaprozin (1.47 g, 5.0 mmol) and K_2CO_3 (1.04 g, 7.5 mmol) in DMF (20.0 mL) to the mixture, dropwise added 1,3-dibromopropane (1.51 g, 7.5 mmol) at 0 ^oC and stirred the mixture at room temperature for 12 h. Diluted the mixture with DCM, washed the mixture with water, separated the organic layer, dried the resultant over anhydrous sodium sulfate and concentrated the mixture under reduced pressure, purified by flash chromatography (Petroleum ether: EtOAc = 6: 1) to provide the title compound (1.64 g, 80% yield). **^{1}H NMR (400 MHz, $CDCl_3$)** δ: 7.26 (dd, *J* = 8.4 Hz, 1.8 Hz, 2H), 7.56 (dd, , *J* = 8.2 Hz, 1.7 Hz, 2H), 7.39-7.30 (m, 6H), 4.28 (t, , *J* = 6.0 Hz, 2H), 3.43 (t, , *J* = 7.2 Hz, 2H), 3.19 (t, , *J* = 7.7 Hz, 2H), 2.93 (t, , *J* = 7.3 Hz, 2H), 2.17 (m, 2H). All data are in accordance with the literature [11].

2-(3-bromopropoxy)-4-chloro-1-(2,4-dichlorophenoxy)benzene (1bu)

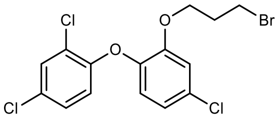

Mixed Triclosan (1.45 g, 5.0 mmol) and K_2CO_3 (1.04 g, 7.5 mmol) in DMF (7.0 mL) to the mixture, dropwise added 1,3-dibromopropane (1.03 mL, 10 mmol) at 0 °C and stirred for 2h, the mixture was heated up to 70 °C for another 2 h. Diluted the mixture with DCM, washed the mixture with water, separated the organic layer, dried the resultant over anhydrous sodium sulfate and concentrated the mixture under reduced pressure, purified by flash chromatography (DCM: PE = 1: 8) to provide the title compound (1.35 g, 66% yield). **^{1}H NMR (400 MHz, $CDCl_3$)** δ: 7.43 (d, *J* = 2.5 Hz, 1H), 7.10 (dd, *J* = 8.8 Hz, 2.5 Hz, 1H), 7.00-6.93 (m, 3H), 6.64 (t, *J* = 8.8 Hz, 1H), 4.06 (t, *J* = 5.6 Hz, 2H), 3.28 (m, *J* = 6.2 Hz, 2H), 2.18-2.12 (m, 2H). All data are in accordance with the literature[12].

3.1.2 Synthesis of olefins

The olefins used in this paper:

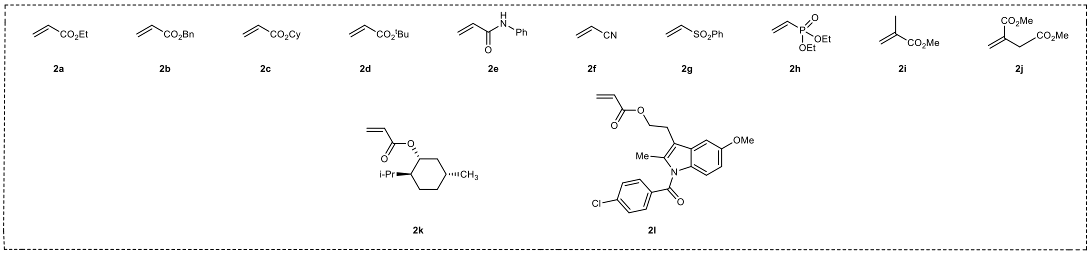

Alkyl bromides **2a-j** are commercially available.

The preparation of **2k** and **2l** are known compounds, and they were synthesized according to the literature procedure.

(1R,2S,5R)-2-isopropyl-5-methylcyclohexyl acrylate (2k)

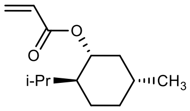

The reaction was carried out with the corresponding alcohol (0.78 g, 5.0 mmol), acryloyl chloride (490 uL, 6 mmol), Et_3N (834 uL, 7.5 mmol), DCM (16.0 mL). The crude product was purified by flash column chromatography on silica gel (Petroleum ether) to afford **2j** (0.89 g, 84% yield). **^{1}H NMR (400 MHz, $CDCl_3$)** δ: 6.38 (d, *J* = 17.4 Hz, 1H), 6.16-6.07 (m, 1H), 5.79 (d, *J* = 10.4 Hz, 1H), 4.76 (td, *J* = 10.7 Hz, 4.4 Hz, 1H), 2.04-2.01 (m, 1H), 1.90-1.83 (m, 1H), 1.70-1.64 (m, 2H), 1.56-1.25 (m, 3H), 1.12-0.99 (m, 2H), 0.90 (t, *J* = 6.1 Hz, 6H), 0.76 (d, *J* = 7.0 Hz, 3H).All data are in accordance with the literature[13].

2-(1-(4-chlorobenzoyl)-5-methoxy-2-methyl-1H-indol-3-yl) ethyl acrylate (2l)

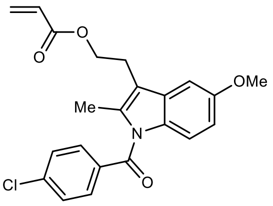

The reaction was carried out with the corresponding alcohol (1.38 g, 4.0 mmol), acryloyl chloride (390 uL, 4.8 mmol), Et_3N (834 uL, 6.0 mmol), DCM (13.3 mL). The crude product was purified by flash column chromatography on silica gel (PE: EA = 10:1) to afford **2k** (1.12 g, 70% yield). **^{1}H NMR (400 MHz, $CDCl_3$)** δ: 7.66 (d, J = 8.6 Hz, 2H), 7.48 (d, J = 8.6 Hz, 2H), 6.98 (d, J = 2.5 Hz, 1H), 6.88 (d, J = 8.9 Hz, 1H), 6.68 (dd, J = 2.5, 9.0 Hz, 1H), 6.42 (dd, J = 1.5, 17.3 Hz, 1H), 6.15 (dd, J = 10.4, 17.4, 1H), 5.86 (dd, J = 1.5, 10.4 Hz, 1H), 4.35 (t, J = 7.1 Hz, 2H), 3.85 (s, 3H), 3.04 (t, J = 7.2 Hz, 2H), 2.37 (s, 3H). All data are in accordance with the literature[14].

3.1.3 Synthesis of chlorogermananes

The chlorogermananes used in this paper:

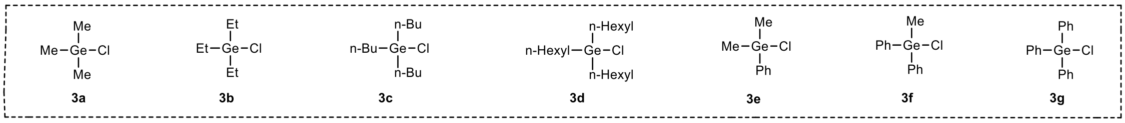

Chlorogermanane **3a-g** are commercially available.

3.1.4 Unsuccessful alkyl bromides and alkenes

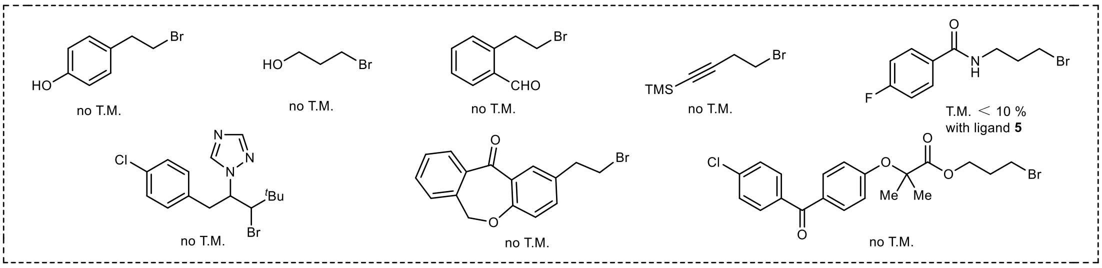

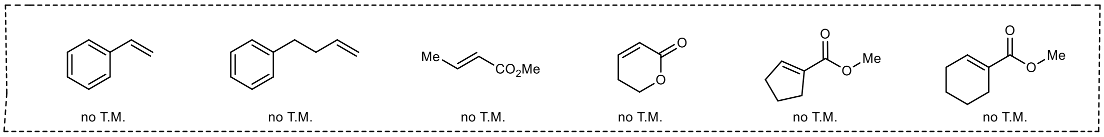

3.2 Experimental Procedures and Characterization of Products

3.2.1 General Procedure

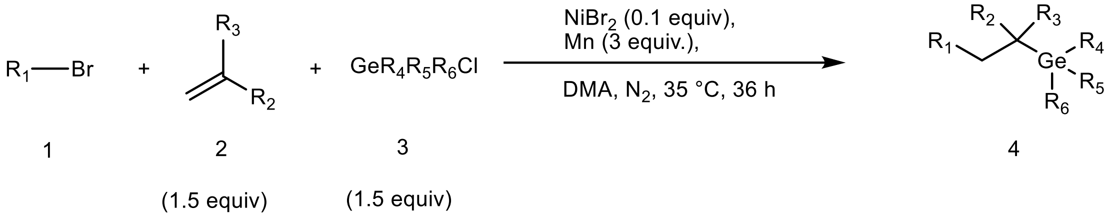

The procedure was conducted in a nitrogen-filled glove box. In an oven-dried 8 mL reaction vial equipped with a magnetic stir bar, alkene (0.45 mmol, 1.5 equiv), germanium reagent (0.45 mmol, 1.5 equiv), $NiBr_2$ (0.03 mmol, 0.1 equiv), Mn (0.9 mmol, 3.0 equiv), DMA (0.1M) was charged and pre-stirred at 35 ºC for 0.5 h, then alkyl bromine (0.3 mmol, 1.0 equiv) was added under N_2, stirring at 35 ºC for another 36 h, subsequently quenched with water (10.0 mL) and extracted with dichloromethane (3 × 15.0 mL). The combined organic layers were washed with water, brine, dried over anhydrous Na_2SO_4, and concentrated under reduced pressure. The residue was purified by flash chromatography on silica gel to afford product.

3.2.2 Characterization Data of Products

Ethyl 6-phenyl-2-(trimethylgermyl)hexanoate (4)

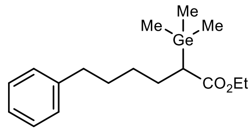

In an oven-dried 8 mL reaction vial equipped with a magnetic stir bar, ethyl acrylate (48.9 μL, 0.45 mmol), trimethylchlorogermane (56.4 μL, 0.45 mmol), $NiBr_2$ (6.6 mg, 0.03 mmol), Mn (49.5 mg, 0.9 mmol), DMA (3.0 mL) were charged under N_2 atmosphere at 35 ºC for 0.5 h, then (3-bromopropyl)benzene (59.7 mg, 0.3 mmol) were added under N_2, stirring at 35 ºC for another 36 h.

The crude material was purified by flash chromatography (Petroleum ether: EtOAc = 40: 1) to provide the title compound as a clear colorless oil (81.0 mg, 80% yield).

^{1}H NMR (400 MHz, CDCl$_3$) δ: 7.29-7.15 (m, 5H), 4.12-4.06 (m, 2H), 2.66-2.53 (m, 2H), 2.07 (dd, J = 11.5 Hz, 3.2 Hz, 1H), 1.90-1.83 (m, 1H), 1.63-1.58 (m, 2H)1.41-1.35 (m, 2H), 1.26-1.21 (m, 4H), 0.20 (s, 9H); **^{13}C NMR (100 MHz, CDCl$_3$)** δ: 175.75, 142.65, 128.36, 128.20, 125.57, 59.52, 36.97, 35.76, 31.24, 29.93, 26.87, 14.54, -2.90. HRMS (ESI): $[M+H]^+$ calculated for $C_{17}H_{28}GeO_2$ = 339.1379, found: 339.1374.

Ethyl 5-(4-methoxyphenyl)-2-(trimethylgermyl)pentanoate (5)

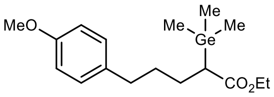

In an oven-dried 8mL reaction vial equipped with a magnetic stir bar, ethyl acrylate (48.9 μL, 0.45 mmol), trimethylchlorogermane (56.4 μL, 0.45 mmol), $NiBr_2$ (6.6 mg, 0.03 mmol), Mn (49.5 mg, 0.9 mmol), DMA (3.0 mL) were charged under N_2 atmosphere at 35 ºC for 0.5 h, then 1-(2-Bromoethyl)-4-Methoxybenzene (64.5 mg, 0.3 mmol) were added under N_2, stirring at 35 ºC for another 36 h.

The crude material was purified by flash chromatography (Petroleum ether: EtOAc = 30: 1) to provide the title compound as a clear pale yellow oil (71.0 mg, 67% yield).

^{1}H NMR (400 MHz, $CDCl_3$) δ: 7.08 (d, *J* = 8.4 Hz, 2H), 6.81 (d, *J* = 8.4 Hz, 2H), 4.12-4.06 (m, 2H), 3.78 (s, 3H), 2.54 (t, *J* = 7.8 Hz, 2H), 2.10 (dd, *J* = 11.5 Hz, 3.5 Hz, 1H). 1.91-1.84 (m, 1H). 1.74-1.65 (m, 1H), 1.52-1.47 (m, 1H), 1.43-1.38 (m, 1H), 1.23 (t, *J* = 7.2 Hz, 3H), 0.20 (s, 9H); **^{13}C NMR (100 MHz, $CDCl_3$)** δ: 175.67, 157.59, 134.44, 129.17, 113.63, 59.53, 55.21, 36.83, 34.69, 32.28, 26.66, 14.54, -2.89.

HRMS (ESI): $[M+H]^+$ calculated for $C_{17}H_{28}GeO_3$ = 355.1328, found: 355.1322.

Ethyl 5-(4-((tert-butoxycarbonyl)amino)phenyl)-2-(trimethylgermyl)pentanoate (6)

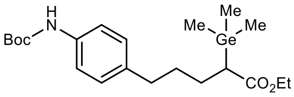

In an oven-dried 8 mL reaction vial equipped with a magnetic stir bar, ethyl acrylate (48.9 μL, 0.45 mmol), trimethylchlorogermane (56.4 μL, 0.45 mmol), $NiBr_2$ (6.6 mg, 0.03 mmol), Mn (49.5 mg, 0.9 mmol), DMA (3.0 mL) were charged under N_2 atmosphere at 35 ºC for 0.5 h, then 3-(Boc-amino)phenethylbromide (90.0 mg, 0.3 mmol) were added under N_2, stirring at 35 ºC for another 36 h.

The crude material was purified by flash chromatography (Petroleum ether: EtOAc = 20: 1) to provide the title compound as a clear pale yellow solid (72.3 mg, 54% yield), melting point: 43-45 ºC.

^{1}H NMR (400 MHz, $CDCl_3$) δ: 7.25 (d, *J* = 8.4 Hz, 2H), 7.08 (d, *J* = 8.4 Hz, 2H), 6.41 (s, 1H), 4.12-4.06 (m, 2H), 2.54 (t, *J* = 7.7 Hz, 2H), 2.10 (dd, *J* = 11.5 Hz, 3.4 Hz,1H), 1.91-1.82 (m, 1H), 1.74-1.61 (m, 2H), 1.51 (s, 9H), 1.44-1.37 (m, 1H), 1.23 (t, *J* = 7.1 Hz, 3H), 0.20 (s, 9H); **^{13}C NMR (100 MHz, $CDCl_3$)** δ: 175.65, 152.85, 137.02, 135.96, 128.73, 118.55, 59.52, 36.80, 34.87, 32.05, 29.65, 28.30, 26.60, 14.52, -2.90.

HRMS (ESI): $[M+H]^+$ calculated for $C_{21}H_{35}GeNO_4$ = 440.1856, found: 440.1851.

Ethyl 5-(4-chlorophenyl)-2-(trimethylgermyl)pentanoate (7)

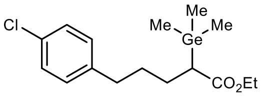

In an oven-dried 8 mL reaction vial equipped with a magnetic stir bar, ethyl acrylate (48.9 μL, 0.45 mmol), trimethylchlorogermane (56.4 μL, 0.45 mmol), $NiBr_2$ (6.6 mg, 0.03 mmol), Mn (49.5 mg, 0.9 mmol), DMA (3.0 mL) were charged under N_2 atmosphere at 35 ºC for 0.5 h, then 4-chlorophenethyl bromide (65.9 mg, 0.3 mmol) were added under N_2, stirring at 35 ºC for another 36 h.

The crude material was purified by flash chromatography (Petroleum ether: EtOAc = 50: 1) to provide the title compound as a clear yellow oil (85.4 mg, 80% yield).

^{1}H NMR (400 MHz, $CDCl_3$) δ: 7.23 (d, J = 8.3 Hz, 2H), 7.09 (d, J = 8.2 Hz, 2H), 4.12-4.06 (m, 2H), 2.57 (t, J = 7.8 Hz, 2H), 2.10 (dd, J = 11.6 Hz, 3.4Hz, 1H), 1.90-1.82 (m, 1H), 1.74-1.64 (m, 1H), 1.55-1.48 (m, 1H), 1.40-1.38 (m, 1H),1.23 (t, J = 7.1 Hz, 3H), , 0.20 (s, 9H); **^{13}C NMR (100 MHz, $CDCl_3$)** δ: 175.59, 140.72, 131.30, 129.66, 128.31, 59.59, 36.78, 34.96, 31.88, 26.60, 14.54, -2.88.

HRMS (ESI): $[M+H]^+$ calculated for $C_{16}H_{25}ClGeO_2$ = 359.0833, found: 359.0833.

Ethyl 5-(4-bromophenyl)-2-(trimethylgermyl)pentanoate (8)

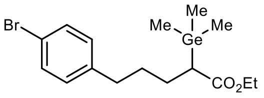

In an oven-dried 8 mL reaction vial equipped with a magnetic stir bar, ethyl acrylate (48.9 μL, 0.45 mmol), trimethylchlorogermane (56.4 μL, 0.45 mmol), $NiBr_2$ (6.6 mg, 0.03 mmol), Mn (49.5 mg, 0.9 mmol), DMA (3.0 mL) were charged under N_2 atmosphere at 35 ºC for 0.5 h, then 1-bromo-4-(2-bromoethyl)benzene (79.2 mg, 0.3 mmol) were added under N_2, stirring at 35 ºC for another 36 h.

The crude material was purified by flash chromatography (Petroleum ether: EtOAc = 40: 1) to provide the title compound as a clear yellow oil (77.9 mg, 65% yield).

^{1}H NMR (400 MHz, $CDCl_3$) δ: 7.38 (d, J = 8.3 Hz, 2H), 7.04 (d, J = 8.3 Hz, 2H), 4.10-4.08 (m, 2H), 2.55 (t, J = 7.8 Hz, 2H), 2.09 (dd, J = 11.6 Hz, 3.4 Hz, 1H), 1.91-1.83 (m, 1H), 1.74-1.66 (m, 1H), 1.53-1.47 (m, 1H), 1.44-1.38 (m, 1H), 1.23 (t, J = 7.2 Hz, 3H), 0.20 (m, 9H); **^{13}C NMR (100 MHz, $CDCl_3$)** δ: 175.56, 141.23, 131.25, 130.08, 119.32, 59.58, 36.77, 35.01, 31.81, 26.59, 14.53, -2.89.

HRMS (ESI): $[M+H]^+$ calculated for $C_{16}H_{25}BrGeO_2$ = 403.0328, found: 403.0323.

Methyl 4-(5-ethoxy-5-oxo-4-(trimethylgermyl)pentyl)benzoate (9)

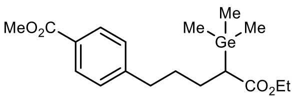

In an oven-dried 8 mL reaction vial equipped with a magnetic stir bar, ethyl acrylate (48.9 μL, 0.45 mmol), trimethylchlorogermane (56.4 μL, 0.45 mmol), $NiBr_2$ (6.6 mg, 0.03 mmol), Mn (49.5 mg, 0.9 mmol), DMA (3.0 mL) were charged under N_2 atmosphere at 35 ºC for 0.5 h, then Methyl 4-(2-bromoethyl)benzoate (72.9 mg, 0.3 mmol) were added under N_2, stirring at 35 ºC for another 36 h.

The crude material was purified by flash chromatography (Petroleum ether: EtOAc = 30: 1) to provide the title compound as a clear yellow oil (72.3 mg, 63% yield).

^{1}H NMR (400 MHz, $CDCl_3$) δ: 7.94 (d, *J* = 8.3 Hz, 2H), 7.23 (d, *J* = 8.2 Hz, 2H), 4.12-4.06 (m, 2H), 3.90 (s, 3H), 2.66 (t, *J* = 7.8 Hz, 2H), 2.10 (dd, *J* = 11.9 Hz, 3.4 Hz, 1H), 1.94-1.84 (m, 1H), 1.78-1.70 (m, 1H), 1.56-1.49 (m, 1H), 1.45-1.37 (m, 1H), 1.23 (t, *J* = 7.2 Hz, 3H), 0.20 (s, 9H); **^{13}C NMR (100 MHz, $CDCl_3$)** δ: 175.56, 167.17, 147.88, 129.63, 128.36, 127.65, 59.60, 51.95, 36.77, 35.65, 31.58, 26.63, 14.53, -2.90.

HRMS (ESI): $[M+H]^+$ calculated for $C_{18}H_{28}GeO_4$ = 383.1277, found: 383.1272.

Ethyl 5-(4-(trifluoromethyl)phenyl)-2-(trimethylgermyl)pentanoate (10)

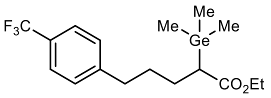

In an oven-dried 8 mL reaction vial equipped with a magnetic stir bar, ethyl acrylate (48.9 μL, 0.45 mmol), trimethylchlorogermane (56.4 μL, 0.45 mmol), $NiBr_2$ (6.6 mg, 0.03 mmol), Mn (49.5 mg, 0.9 mmol), DMA (3.0 mL) were charged under N_2 atmosphere at 35 ºC for 0.5 h, then 1-(2-bromoethyl)-4-(trifluoromethyl)benzene (75.9 mg, 0.3 mmol) were added under N_2, stirring at 35 ºC for another 36 h.

The crude material was purified by flash chromatography (Petroleum ether: EtOAc = 30: 1) to provide the title compound as a clear yellow oil (67.9 mg, 58% yield).

^{1}H NMR (400 MHz, $CDCl_3$) δ: 7.52 (d, *J* = 8.0 Hz, 2H), 7.27 (d, *J* = 8.1 Hz, 2H), 4.12-4.06 (m, 2H), 2.66 (t, *J* = 7.9 Hz, 2H), 2.11 (dd, *J* = 11.5 Hz, 3.3 Hz, 1H), 1.95-1.85 (m, 1H), 1.78-1.69 (m, 1H), 1.56-1.49 (m, 1H), 1.46-1.39 (m, 1H), 1.23 (t, *J* = 7.2 Hz, 3H), 0.20 (s, 9H); **^{13}C NMR (100 MHz, $CDCl_3$)** δ: 175.56, 146.42, 128.60, 128.18, 127.86, 125.70, 125.17 (q, *J* = 3.8 Hz), 59.62, 36.78, 35.48, 31.70, 26.66, 14.52, -2.90; **^{19}F NMR (376 MHz, $CDCl_3$)** δ: -62.16.

HRMS (ESI): $[M+H]^+$ calculated for $C_{17}H_{25}F_3GeO_2$ = 393.1096, found: 393.1091.

Ethyl 5-(thiophen-2-yl)-2-(trimethylgermyl)pentanoate (11)

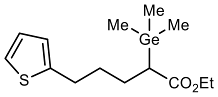

In an oven-dried 8 mL reaction vial equipped with a magnetic stir bar, ethyl acrylate (48.9 μL, 0.45 mmol), trimethylchlorogermane (56.4 μL, 0.45 mmol), $NiBr_2$ (6.6 mg, 0.03 mmol), Mn (49.5 mg, 0.9 mmol), DMA (3.0 mL) were charged under N_2 atmosphere at 35 ºC for 0.5 h, then 2-(2-bromoethyl)thiophene (57.3 mg, 0.3 mmol) were added under N_2, stirring at 35 ºC for another 36 h.
The crude material was purified by flash chromatography (Petroleum ether: EtOAc = 80: 1) to provide the title compound as a clear yellow oil (61.1 mg, 62% yield).
^{1}H NMR (400 MHz, $CDCl_3$) δ: 7.10 (d, *J* = 5.2 Hz, 1H), 6.91-6.89 (m, 1H), 6.78-6.77 (m, 1H), 4.13-4.07 (m, 2H), 2.82 (t, *J* = 7.7 Hz, 2H), 2.11 (dd, *J* = 11.5 Hz, 3.3 Hz, 1H), 1.96-1.86 (m, 1H), 1.83-1.73 (m, 1H), 1.50-1.42 (m, 1H), 1.24 (t, *J* = 7.1 Hz, 3H), 0.21 (s, 9H); **^{13}C NMR (100 MHz, $CDCl_3$)** δ: 175.54, 145.13, 126.61, 123.98, 122.81, 59.58, 36.65, 32.22, 29.54, 26.44, 14.53, -2.90.
HRMS (ESI): $[M+H]^+$ calculated for $C_{14}H_{24}GeO_2S$ = 331.0868, found: 331.0782.

Ethyl 5-(benzofuran-2-yl)-2-(trimethylgermyl)pentanoate (12)

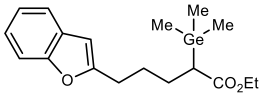

In an oven-dried 8 mL reaction vial equipped with a magnetic stir bar, ethyl acrylate (48.9 μL, 0.45 mmol), trimethylchlorogermane (56.4 μL, 0.45 mmol), $NiBr_2$ (6.6 mg, 0.03 mmol), (4R,4'R)-4,4'-diisopropyl-4,4',5,5'-tetrahydro-2,2'-bioxazole (0.036mmol, 8.1 mg), Mn (49.5 mg, 0.9 mmol), DMA (3.0 mL) were charged under N_2 atmosphere at 35 ºC for 0.5 h, then 2-(2-bromoethyl)benzofuran (67.2 mg, 0.3 mmol) were added under N_2, stirring at 35 ºC for another 36 h.
The crude material was purified by flash chromatography (Petroleum ether: EtOAc = 10: 1, DCM) to provide the title compound as a clear yellow oil (42.6 mg, 39% yield).
^{1}H NMR (400 MHz, $CDCl_3$) δ: 7.47 (d, *J* = 6.8 Hz, 1H), 7.39 (d, *J* = 8.0 Hz, 1H), 7.22-7.14 (m, 2H), 6.38 (s, 1H), 4.14-4.08 (m, 2H), 2.77 (t, *J* = 7.5 Hz, 2H), 2.15 (dd, *J* = 11.54 Hz, 3.3 Hz ,1H), 2.00-1.79 (m, 2H), 1.74-1.65 (m, 1H), 1.50-1.43 (m, 1H), 1.24 (t, *J* = 7.2 Hz, 3H), 0.21 (s, 9H); **^{13}C NMR (100 MHz, $CDCl_3$)** δ: 175.54, 159.15, 154.56, 128.92, 123.03, 122.33, 120.17, 110.65, 101.84, 59.63, 36.71, 28.14, 28.10, 26.57, 14.53, -2.87.
HRMS (ESI): $[M+H]^+$ calculated for $C_{18}H_{26}GeO_3$ = 365.1172, found: 365.1167.

Tert-butyl 3-(5-ethoxy-5-oxo-4-(trimethylgermyl)pentyl)-1H-indole-1-carboxylate (13)

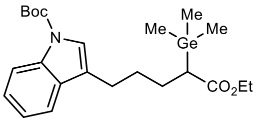

In an oven-dried 8 mL reaction vial equipped with a magnetic stir bar, ethyl acrylate (48.9 µL, 0.45 mmol), trimethylchlorogermane (56.4 µL, 0.45 mmol), $NiBr_2$ (6.6 mg, 0.03 mmol), (4R,4'R)-4,4'-diisopropyl-4,4',5,5'-tetrahydro-2,2'-bioxazole (0.036mmol, 8.1 mg), Mn (49.5 mg, 0.9 mmol), DMA (3.0 mL) were charged under N_2 atmosphere at 35 ºC for 0.5 h, then tert-butyl 3-(2-bromoethyl)-3a,7a-dihydro-1H-indole-1-carboxylate (96.9 mg, 0.3 mmol) were added under N_2, stirring at 35 ºC for another 36 h.

The crude material was purified by flash chromatography (Petroleum ether: EtOAc = 50: 1) to provide the title compound as a clear yellow oil (63.3 mg, 46% yield).

^{1}H NMR (400 MHz, CDCl$_3$) δ: 8.12 (s, 1H), 7.50 (d, *J* = 8.2 Hz, 1H), 7.34-7.26 (m, 2H), 7.22 (t, *J* = 8.2 Hz, 1H), 4.13-4.07 (m, 2H), 2.67 (t, *J* = 7.1 Hz, 2H), 2.15 (dd, *J* = 11.6 Hz, 3.4 Hz, 1H), 2.02-1.92 (m, 1H), 1.86-1.76 (m, 1H), 1.66 (s, 9H), 1.62-1.60 (m, 1H), 1.52-1.46 (m, 1H), 1.23 (t, *J* = 7.1 Hz, 3H), 0.22 (s, 9H); **^{13}C NMR (100 MHz, CDCl$_3$)** δ: 175.63, 149.82, 130.68, 124.14, 122.19, 120.94, 118.94, 115.17, 83.20, 59.58, 36.76, 29.70, 28.20, 26.91, 24.57, 14.53, -2.88.

HRMS (ESI): $[M+H]^+$ calculated for $C_{23}H_{35}GeNO_4$ = 464.1856, found: 464.1851.

Ethyl 6-(9H-carbazol-9-yl)-2-(trimethylgermyl)hexanoate (14)

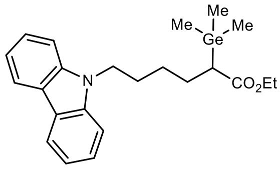

In an oven-dried 8 mL reaction vial equipped with a magnetic stir bar, ethyl acrylate (48.9 µL, 0.45 mmol), trimethylchlorogermane (56.4 µL, 0.45 mmol), $NiBr_2$ (6.6 mg, 0.03 mmol), Mn (49.5 mg, 0.9 mmol), DMA (3.0 mL) were charged under N_2 atmosphere at 35 ºC for 0.5 h, then 9-(3-bromopropyl)-4a,9a-dihydro-9H-carbazole (86.7 mg, 0.3 mmol) were added under N_2, stirring at 35 ºC for another 36 h.

The crude material was purified by flash chromatography (Petroleum ether: EtOAc = 40: 1) to provide the title compound as a clear yellow oil (70.4 mg, 55% yield).

^{1}H NMR (400 MHz, CDCl$_3$) δ: 8.11 (d, *J* = 7.8 Hz, 2H), 7.49-7.45 (m, 2H), 7.40 (d, *J* = 8.2 Hz, 2H), 7.23 (t, *J* = 7.8 Hz, 2H), 4.30 (t, *J* = 7.3 Hz, 2H), 4.12-4.06 (m, 2H), 2.04

(dd, J = 11.5 Hz, 5.3 Hz, 1H), 1.93-1.83 (m, 3H), 1.56-1.47 (m, 1H), 1.41-1.31 (m, 2H), 1.22 (t, J = 7.2 Hz, 3H), 0.19 (s, 9H). **^{13}C NMR (100 MHz, $CDCl_3$)** δ: 175.59, 140.30, 125.51, 122.73, 120.27, 118.66, 108.59, 59.56, 42.83, 36.80, 28.68, 27.93, 26.73, 14.49, -2.96.

HRMS (ESI): $[M+H]^+$ calculated for $C_{23}H_{31}GeNO_2$ = 428.1645, found: 428.1640.

Ethyl 5-(quinolin-6-yl)-2-(trimethylgermyl)pentanoate (15)

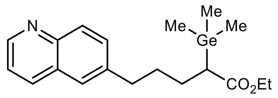

In an oven-dried 8 mL reaction vial equipped with a magnetic stir bar, ethyl acrylate (48.9 μL, 0.45 mmol), trimethylchlorogermane (56.4 μL, 0.45 mmol), $NiBr_2$ (6.6 mg, 0.03 mmol), (4R,4'R)-4,4'-diisopropyl-4,4',5,5'-tetrahydro-2,2'-bioxazole (0.036mmol, 8.1 mg), Mn (49.5 mg, 0.9 mmol), DMA (3.0 mL) were charged under N_2 atmosphere at 35 ºC for 0.5 h, then 6-(2-bromoethyl)quinoline (70.5 mg, 0.3 mmol) were added under N_2, stirring at 35 ºC for another 36 h.

The crude material was purified by flash chromatography (Petroleum ether: EtOAc = 80: 1) to provide the title compound as a clear yellow oil (30.7 mg, 27% yield).

^{1}H NMR (400 MHz, $CDCl_3$) δ: 8.85 (dd, J = 4.2 Hz, 1.7 Hz, 1H), 8.09 (d, J = 8.2 Hz, 1H), 8.01 (d, J = 8.5 Hz, 1H), 7.57-7.54 (m, 2H), 7.38-7.35 (m, 1H), 4.14-4.06 (m, 2H), 2.80 (t, J = 7.6 Hz, 2H), 2.14 (dd, J = 11.4 Hz, 3.3 Hz, 1H), 1.99-1.90 (m, 1H), 1.87-1.78 (m, 1H), 1.70-1.63 (m, 1H), 1.51-1.43 (m, 1H), 1.23 (t, J = 7.1 Hz, 3H), 0.21 (s, 9H); **^{13}C NMR (100 MHz, $CDCl_3$)** δ: 175.58, 149.58, 147.04, 140.73, 135.53, 130.91, 129.18, 128.26, 125.98, 121.02, 59.59, 36.80, 35.56, 31.74, 26.71, 14.53, -2.89.

HRMS (ESI): $[M+H]^+$ calculated for $C_{19}H_{27}GeNO_2$ = 376.1332, found: 376.1326.

Ethyl 2-(trimethylgermyl)heptanoate (16)

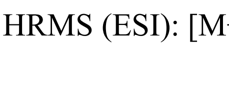

In an oven-dried 8 mL reaction vial equipped with a magnetic stir bar, ethyl acrylate (48.9 μL, 0.45 mmol), trimethylchlorogermane (56.4 μL, 0.45 mmol), $NiBr_2$ (6.6 mg, 0.03 mmol), Mn (49.5 mg, 0.9 mmol), DMA (3.0 mL) were charged under N_2 atmosphere at 35 ºC for 0.5 h, then butyl bromide (41.1 mg, 0.3 mmol) was added under N_2, stirring at 35 ºC for another 36 h.

The crude material was purified by flash chromatography (Petroleum ether: EtOAc = 70: 1) to provide the title compound as a clear yellow oil (52.6 mg, 64% yield).

^{1}H NMR (400 MHz, CDCl$_3$) δ: 4.12-4.07 (m, 2H), 2.07 (dd, J = 11.5 Hz, 3.1 Hz, 1H), 1.85-1.78 (m, 1H), 1.37-1.34 (m, 2H), 1.29-1.22 (m, 8H), 0.87 (t, J = 6.8 Hz, 3H), 0.2 (s, 9H); **^{13}C NMR (100 MHz, CDCl$_3$)** δ: 175.83, 59.46, 37.09, 31.58, 29.95, 26.97, 22.49, 14.54, 14.04, -2.90

HRMS (ESI): $[M+H]^+$ calculated for $C_{12}H_{26}GeO_2$ = 277.1223, found: 277.1218.

Ethyl 4-cyclobutyl-2-(trimethylgermyl)butanoate (17)

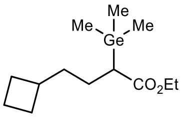

In an oven-dried 8 mL reaction vial equipped with a magnetic stir bar, ethyl acrylate (48.9 μL, 0.45 mmol), trimethylchlorogermane (56.4 μL, 0.45 mmol), $NiBr_2$ (6.6 mg, 0.03 mmol), Mn (49.5 mg, 0.9 mmol), DMA (3.0 mL) were charged under N_2 atmosphere at 35 ºC for 0.5 h, then (bromomethyl)cyclobutane (44.7 mg, 0.3 mmol) were added under N_2, stirring at 35 ºC for another 36 h.

The crude material was purified by flash chromatography (Petroleum ether: EtOAc = 40: 1) to provide the title compound as a clear pale yellow oil (59.9 mg, 73% yield).

^{1}H NMR (400 MHz, CDCl$_3$) δ: 4.13-4.07 (m, 2H), 2.26-2.19 (m, 1H), 2.05-1.97 (m, 3H), 1.84-1.59 (m, 4H), 1.56-1.51 (m, 1H), 1.43-1.28 (m, 3H), 1.24 (t, J = 7.2 Hz, 3H), 0.2 (s, 9H); **^{13}C NMR (100 MHz, CDCl$_3$)** δ: 175.79, 59.47, 37.48, 36.97, 35.76, 28.35, 28.05, 24.51, 18.45, 14.55, -2.89.

HRMS (ESI): $[M+H]^+$ calculated for $C_{13}H_{26}GeO_2$ = 289.1223, found: 289.1218.

Ethyl 6-methoxy-2-(trimethylgermyl)hexanoate (18)

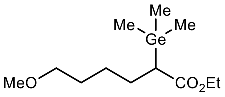

In an oven-dried 8 mL reaction vial equipped with a magnetic stir bar, ethyl acrylate (48.9 μL, 0.45 mmol), trimethylchlorogermane (56.4 μL, 0.45 mmol), $NiBr_2$ (6.6 mg, 0.03 mmol), Mn (49.5 mg, 0.9 mmol), DMA (3.0 mL) were charged under N_2 atmosphere at 35 ºC for 0.5 h, then 1-bromo-3-methoxypropane (45.9 mg, 0.3 mmol) were added under N_2, stirring at 35 ºC for another 36 h.

The crude material was purified by flash chromatography (Petroleum ether: EtOAc = 20: 1) to provide the title compound as a clear yellow oil (47.3 mg, 54% yield).

^{1}H NMR (400 MHz, CDCl$_3$) δ: 4.12-4.07 (m, 2H), 3.37-3.32 (m, 5H), 2.08 (dd, J = 11.5 Hz, 3.1 Hz, 1H), 1.88-1.81 (m, 1H), 1.56-1.53 (m, 2H), 1.41-1.31 (m, 3H), 1.23 (t, J = 7.1 Hz, 3H), 0.21 (s, 9H); **^{13}C NMR (100 MHz, CDCl$_3$)** δ: 175.69, 72.64, 59.52,

58.49, 37.01, 29.36, 26.89, 26.82, 14.52, -2.92.

HRMS (ESI): $[M+H]^+$ calculated for $C_{12}H_{26}GeO_3$ = 293.1172, found: 293.1166.

Ethyl 7-fluoro-2-(trimethylgermyl)heptanoate (19)

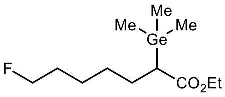

In an oven-dried 8 mL reaction vial equipped with a magnetic stir bar, ethyl acrylate (48.9 μL, 0.45 mmol), trimethylchlorogermane (56.4 μL, 0.45 mmol), $NiBr_2$ (6.6 mg, 0.03 mmol), Mn (49.5 mg, 0.9 mmol), DMA (3.0 mL) were charged under N_2 atmosphere at 35 ºC for 0.5 h, then 1-bromo-4-fluorobutane (46.5 mg, 0.3 mmol) were added under N_2, stirring at 35 ºC for another 36 h.

The crude material was purified by flash chromatography (Petroleum ether: EtOAc = 40: 1) to provide the title compound as a clear yellow oil (61.3 mg, 70% yield).

^{1}H NMR (400 MHz, CDCl$_3$) δ:4.48 (t, J = 6.2 Hz, 1H), 4.37 (t, J =6.2Hz, 1H), 4.13-4.07 (m, 2H), 2.08 (dd, J = 11.8 Hz, 3.1 Hz, 1H), 1.88-1.80 (m, 1H), 1.73-1.63 (m, 2H), 1.44-1.35 (m, 4H), 1.29-1.28 (m, 1H), 1.24 (t, J = 7.1 Hz, 3H), 0.21 (s, 9H); **^{13}C NMR (100 MHz, CDCl$_3$)** δ: 175.67, 84.04 (d, J = 164.7 Hz), 59.51, 36.91, 30.16 (d, J = 19.3 Hz), 29.80, 26.82, 24.88 (d, J = 5.4 Hz), 14.49, -2.96; **^{19}F NMR (376 MHz, CDCl$_3$)** δ: -218.01

HRMS (ESI): $[M+H]^+$ calculated for $C_{12}H_{25}FGeO_2$ = 295.1128, found: 295.1123.

Ethyl 7-chloro-2-(trimethylgermyl)heptanoate (20)

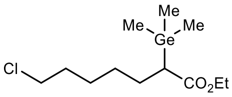

In an oven-dried 8 mL reaction vial equipped with a magnetic stir bar, ethyl acrylate (48.9 μL, 0.45 mmol), trimethylchlorogermane (56.4 μL, 0.45 mmol), $NiBr_2$ (6.6 mg, 0.03 mmol), Mn (49.5 mg, 0.9 mmol), DMA (3.0 mL) were charged under N_2 atmosphere at 35 ºC for 0.5 h, then 1-bromo-4-chlorobutane (51.4 mg, 0.3 mmol) were added under N_2, stirring at 35 ºC for another 36 h.

The crude material was purified by flash chromatography (Petroleum ether: EtOAc = 40: 1) to provide the title compound as a clear yellow oil (67.5 mg, 73% yield).

^{1}H NMR (400 MHz, CDCl$_3$) δ: 4.13-4.07 (m, 2H), 3.52 (t, J = 6.7 Hz, 2H), 2.07 (dd, J = 11.9 Hz, 2.9 Hz, 1H), 1.87-1.73 (m, 3H), 1.45-1.28 (m, 5H), 1.24 (t, J = 7.1 Hz, 3H), 0.21 (s, 9H); **^{13}C NMR (100 MHz, CDCl$_3$)** δ: 175.61, 59.52, 45.01, 36.91, 32.37, 29.47, 26.80, 26.59, 14.52, -2.93.

HRMS (ESI): $[M+H]^+$ calculated for $C_{12}H_{25}ClGeO_2$ = 311.0833, found: 311.0828.

1-ethyl 8-methyl 2-(trimethylgermyl)octanedioate (21)

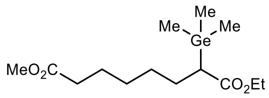

In an oven-dried 8 mL reaction vial equipped with a magnetic stir bar, ethyl acrylate (48.9 μL, 0.45 mmol), trimethylchlorogermane (56.4 μL, 0.45 mmol), $NiBr_2$ (6.6 mg, 0.03 mmol), Mn (49.5 mg, 0.9 mmol), DMA (3.0 mL) were charged under N_2 atmosphere at 35 ºC for 0.5 h, then methyl 5-bromovalerate (58.5 mg, 0.3 mmol) were added under N_2, stirring at 35 ºC for another 36 h.

The crude material was purified by flash chromatography (Petroleum ether: EtOAc = 10: 1) to provide the title compound as a clear pale yellow oil (77.2 mg, 77% yield).

^{1}H NMR (400 MHz, $CDCl_3$) δ: 4.12-4.06 (m, 2H), 3.66 (s, 3H), 2.29 (t, *J* = 7.6 Hz, 2H), 2.06 (dd, *J* = 11.6 Hz, 3.0 Hz, 1H), 1.86-1.78 (m, 1H), 1.64-1.61 (m, 2H), 1.38-1.25 (m, 5H), 1.23 (t, *J* = 7.2 Hz, 3H), 0.20 (s, 9H); **^{13}C NMR (100 MHz, $CDCl_3$)** δ: 175.69, 174.21, 59.52, 51.43, 37.00, 34.00, 29.89, 28.88, 26.82, 24.76, 14.45, -2.90.

HRMS (ESI): $[M+H]^+$ calculated for $C_{14}H_{28}GeO_4$ = 335.1277, found: 335.1272.

Ethyl 8-oxo-2-(trimethylgermyl)nonanoate (22)

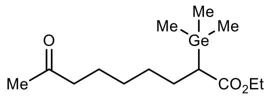

In an oven-dried 8 mL reaction vial equipped with a magnetic stir bar, ethyl acrylate (48.9 μL, 0.45 mmol), trimethylchlorogermane (56.4 μL, 0.45 mmol), $NiBr_2$ (6.6 mg, 0.03 mmol), Mn (49.5 mg, 0.9 mmol), DMA (3.0 mL) were charged under N_2 atmosphere at 35 ºC for 0.5 h, then 1-bromo-5-hexanone (53.7 mg, 0.3 mmol) were added under N_2, stirring at 35 ºC for another 36 h.

The crude material was purified by flash chromatography (Petroleum ether: EtOAc = 15: 1) to provide the title compound as a clear yellow oil (47.3 mg, 50% yield).

^{1}H NMR (400 MHz, $CDCl_3$) δ: 4.12-4.06 (m, 2H), 2.40 (t, *J* = 7.4 Hz, 2H), 2.13 (s, 3H), 2.09-2.04 (m, 1H), 1.85-1.78 (m, 1H), 1.59-1.52 (m, 2H), 1.42-1.28 (m, 5H), 1.23 (t, *J* = 7.2 Hz, 3H), 0.20 (s, 9H); **^{13}C NMR (100 MHz, $CDCl_3$) δ:** 209.29, 175.71, 59.49, 43.64, 36.96, 29.97, 28.86, 26.78, 23.58, 14.51, -2.94

HRMS (ESI): $[M+H]^+$ calculated for $C_{14}H_{28}GeO_3$ = 319.1328, found: 319.1323.

Ethyl 7-cyano-2-(trimethylgermyl)heptanoate (23)

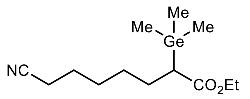

In an oven-dried 8 mL reaction vial equipped with a magnetic stir bar, ethyl acrylate (48.9 μL, 0.45 mmol), trimethylchlorogermane (56.4 μL, 0.45 mmol), $NiBr_2$ (6.6 mg, 0.03 mmol), Mn (49.5 mg, 0.9 mmol), DMA (3.0 mL) were charged under N_2 atmosphere at 35 ºC for 0.5 h, then 5-bromovaleronitrile (48.6 mg, 0.3 mmol) were added under N_2, stirring at 35 ºC for another 36 h.

The crude material was purified by flash chromatography (Petroleum ether: EtOAc = 20: 1) to provide the title compound as a clear pale yellow oil (56.3 mg, 62% yield).

^{1}H NMR (400 MHz, $CDCl_3$) δ: 4.13-4.07 (m, 2H), 2.33 (t, J = 7.1 Hz, 2H), 2.07 (dd, J = 11.8 Hz, 2.9 Hz, 1H), 1.88-1.80 (m, 1H), 1.69-1.62 (m, 2H), 1.49-1.28 (m, 5H), 1.24 (t, J = 7.2 Hz, 3H), 0.21 (s, 9H); **^{13}C NMR (100 MHz, $CDCl_3$)** δ: 175.59, 119.74, 59.60, 36.87, 29.35, 28.35, 26.62, 25.13, 17.04, 14.52, -2.91.

HRMS (ESI): $[M+H]^+$ calculated for $C_{13}H_{25}GeNO_2$ = 302.1175, found: 302.1170.

Ethyl 7,7,7-trifluoro-2-(trimethylgermyl)heptanoate (24)

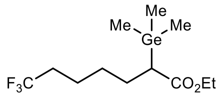

In an oven-dried 8 mL reaction vial equipped with a magnetic stir bar, ethyl acrylate (48.9 μL, 0.45 mmol), trimethylchlorogermane (56.4 μL, 0.45 mmol), $NiBr_2$ (6.6 mg, 0.03 mmol), Mn (49.5 mg, 0.9 mmol), DMA (3.0 mL) were charged under N_2 atmosphere at 35 ºC for 0.5 h, then 1-bromo-4,4,4-trifluorobutane (57.3 mg, 0.3 mmol) were added under N_2, stirring at 35 ºC for another 36 h.

The crude material was purified by flash chromatography (Petroleum ether: EtOAc = 70: 1) to provide the title compound as a clear pale yellow oil (56.0 mg, 57% yield).

^{1}H NMR (400 MHz, $CDCl_3$) δ: 4.13-4.07 (m, 2H), 2.09-2.02 (m, 3H), 1.90-1.81 (m, 1H), 1.56-1.50 (m, 2H), 1.43-1.28 (m, 3H), 1.24 (t, J = 7.1 Hz, 3H), 0.22 (s, 9H); **^{13}C NMR (100 MHz, $CDCl_3$)** δ: 175.50, 59.62, 36.76, 33.53 (q, J = 28.4 Hz), 29.33, 26.59, 21.65, 14.50, -2.94; **^{19}F NMR (376 MHz, $CDCl_3$)** δ: -66.33

HRMS (ESI): $[M+H]^+$ calculated for $C_{12}H_{23}F_3GeO_2$ = 331.0940, found: 331.0934.

Ethyl 2-(trimethylgermyl)oct-7-enoate (25)

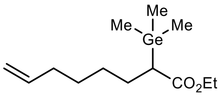

In an oven-dried 8 mL reaction vial equipped with a magnetic stir bar, ethyl acrylate (48.9 μL, 0.45 mmol), trimethylchlorogermane (56.4 μL, 0.45 mmol), $NiBr_2$ (6.6 mg, 0.03 mmol), Mn (49.5 mg, 0.9 mmol), DMA (3.0 mL) were charged under N_2 atmosphere at 35 ºC for 0.5 h, then 5-bromo-1-pentene (44.7 mg, 0.3 mmol) were added under N_2, stirring at 35 ºC for another 36 h.

The crude material was purified by flash chromatography (Petroleum ether: EtOAc = 80: 1) to provide the title compound as a clear yellow oil (51.0 mg, 59% yield).

^{1}H NMR (400 MHz, $CDCl_3$) δ: 5.84-5.74 (m, 1H), 5.01-4.91 (m, 2H), 4.12-4.07 (m, 2H), 2.09-2.00 (m, 3H), 1.87-1.78 (m, 1H), 1.42-1.29 (m, 5H), 1.23 (t, J = 7.1 Hz, 3H), 0.21 (s, 9H); **^{13}C NMR (100 MHz, $CDCl_3$)** δ: 175.73, 138.92 ,114.25, 59.49, 37.01, 33.58, 29.75, 28.63, 26.86, 14.54, -2.91.

HRMS (ESI): $[M+H]^+$ calculated for $C_{13}H_{26}GeO_2$ = 289.1223, found: 289.1217.

Ethyl (Z)-2-(trimethylgermyl)tridec-7-enoate (26)

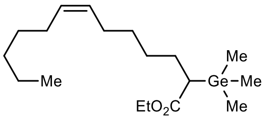

In an oven-dried 8 mL reaction vial equipped with a magnetic stir bar, ethyl acrylate (48.9 μL, 0.45 mmol), trimethylchlorogermane (56.4 μL, 0.45 mmol), $NiBr_2$ (6.6 mg, 0.03 mmol), Mn (49.5 mg, 0.9 mmol), DMA (3.0 mL) were charged under N_2 atmosphere at 35 ºC for 0.5 h, then (Z)-1-bromodec-4-ene (65.4 mg, 0.3 mmol) were added under N_2, stirring at 35 ºC for another 36 h.

The crude material was purified by flash chromatography (Petroleum ether: EtOAc = 60: 1) to provide the title compound as a clear yellow oil (70.1 mg, 65% yield).

^{1}H NMR (400 MHz, $CDCl_3$) δ: 5.37-5.29 (m, 2H), 4.13-4.07 (m, 2H), 2.08-1.98 (m, 5H), 1.87-1.79 (m, 1H), 1.38-1.22 (m, 14H), 0.88 (t, J = 6.8 Hz, 3H), 0.21 (s, 9H); **^{13}C NMR (100 MHz, $CDCl_3$)** δ: 175.77, 130.04, 129.58, 59.48, 37.02, 31.50, 29.92, 29.48, 29.42, 27.16, 27.03, 26.93, 22.56, 14.55, 14.06, -2.90.

HRMS (ESI): $[M+H]^+$ calculated for $C_{18}H_{36}GeO_2$ = 359.2005, found: 359.2001.

Ethyl 5-(1,3-dioxolan-2-yl)-2-(trimethylgermyl)pentanoate (27)

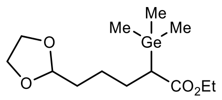

In an oven-dried 8 mL reaction vial equipped with a magnetic stir bar, ethyl acrylate (48.9 μL, 0.45 mmol), trimethylchlorogermane (56.4 μL, 0.45 mmol), $NiBr_2$ (6.6 mg, 0.03 mmol), Mn (49.5 mg, 0.9 mmol), DMA (3.0 mL) were charged under N_2 atmosphere at 35 ºC for 0.5 h, then 2-(2-bromoethyl)-1,3-dioxolane (54.3 mg, 0.3 mmol) were added under N_2, stirring at 35 ºC for another 36 h.

The crude material was purified by flash chromatography (Petroleum ether: EtOAc = 20: 1) to provide the title compound as a clear yellow oil (62.0 mg, 65% yield).

^{1}H NMR (400 MHz, $CDCl_3$) δ: 4.83 (t, *J* = 4.8 Hz, 1H), 4.12-4.06 (m, 2H), 3.97-3.93 (m, 2H), 3.85-3.81 (m, 2H), 2.08 (dd, *J* = 11.4 Hz, 3.1 Hz, 1H), 1.92-1.83 (m, 1H), 1.67-1.63 (m, 2H), 1.55-1.33 (m, 3H), 1.23 (t, *J* = 7.2 Hz, 3H), 0.21 (s, 9H); **^{13}C NMR (100 MHz, $CDCl_3$)** δ: 175.59, 104.41, 64.81, 59.55, 36.98, 33.59, 26.96, 24.73, 14.54, -2.90.

HRMS (ESI): $[M+H]^+$ calculated for $C_{13}H_{26}GeO_4$ = 321.1121, found: 321.1115.

Ethyl 6-(diethoxyphosphoryl)-2-(trimethylgermyl)hexanoate (28)

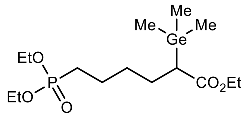

In an oven-dried 8 mL reaction vial equipped with a magnetic stir bar, ethyl acrylate (48.9 μL, 0.45 mmol), trimethylchlorogermane (56.4 μL, 0.45 mmol), $NiBr_2$ (6.6 mg, 0.03 mmol), Mn (49.5 mg, 0.9 mmol), DMA (3.0 mL) were charged under N_2 atmosphere at 35 ºC for 0.5 h, then diethyl(3-bromopropyl)phosphonate (77.7 mg, 0.3 mmol) were added under N_2, stirring at 35 ºC for another 36 h.

The crude material was purified by flash chromatography (Petroleum ether: EtOAc = 1: 1) to provide the title compound as a clear pale yellow oil (64.4 mg, 54% yield).

^{1}H NMR (400 MHz, $CDCl_3$) δ: 4.13-4.03 (m, 6H), 2.06 (dd, *J* = 11.5 Hz, 3.1 Hz, 1H), 1.88-1.80 (m, 1H), 1.76-1.67 (m, 2H), 1.61-1.35 (m, 5H), 1.31 (t, *J* = 7.0 Hz, 6H), 1.23 (t, *J* = 7.2 Hz, 3H), 0.20 (s, 9H); **^{13}C NMR (100 MHz, $CDCl_3$)** δ: 175.59, 61.37, 59.57, 36.77, 31.28, 31.11, 26.52, 26.20, 24.80, 22.25, 16.40, 14.51, -2.92.

HRMS (ESI): $[M+H]^+$ calculated for $C_{15}H_{33}GeO_5P$ = 399.1355, found: 399.1350.

Ethyl 6-(methylsulfonyl)-2-(trimethylgermyl)hexanoate (29)

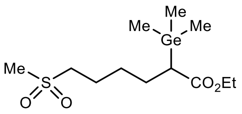

In an oven-dried 8 mL reaction vial equipped with a magnetic stir bar, ethyl acrylate (48.9 μL, 0.45 mmol), trimethylchlorogermane (56.4 μL, 0.45 mmol), $NiBr_2$ (6.6 mg, 0.03 mmol), Mn (49.5 mg, 0.9 mmol), DMA (3.0 mL) were charged under N_2 atmosphere at 35 ºC for 0.5 h, then 1-bromo-3-(methylsulfonyl)propane (60.0 mg, 0.3 mmol) were added under N_2, stirring at 35 ºC for another 36 h.

The crude material was purified by flash chromatography (Petroleum ether: EtOAc = 2: 1) to provide the title compound as a clear yellow oil (54.9 mg, 54% yield).

^{1}H NMR (400 MHz, $CDCl_3$) δ: 4.14-4.05 (m, 2H), 3.00 (t, *J* = 9.3 Hz, 2H), 2.89 (s, 3H), 2.08 (dd, *J* = 11.9 Hz, 2.9 Hz, 1H), 1.91-1.79 (m, 3H), 1.56-1.48 (m, 1H), 1.45-1.36 (m, 2H), 1.24 (t, *J* = 7.1 Hz, 3H), 0.22 (s, 9H); **^{13}C NMR (100 MHz, $CDCl_3$)** δ: 175.42, 59.69, 54.54, 40.41, 36.56, 28.95 26.36, 22.14, 14.49, -2.92.

HRMS (ESI): $[M+H]^+$ calculated for $C_{12}H_{26}GeO_4S$ = 341.0842, found: 341.0837.

Ethyl 6-(dibenzylamino)-2-(trimethylgermyl)hexanoate (30)

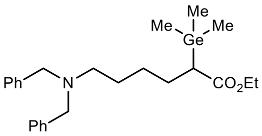

In an oven-dried 8 mL reaction vial equipped with a magnetic stir bar, ethyl acrylate (48.9 μL, 0.45 mmol), trimethylchlorogermane (56.4 μL, 0.45 mmol), $NiBr_2$ (6.6 mg, 0.03 mmol), (4R,4'R)-4,4'-diisopropyl-4,4',5,5'-tetrahydro-2,2'-bioxazole (0.036mmol, 8.1 mg), Mn (49.5 mg, 0.9 mmol), DMA (3.0 mL) were charged under N_2 atmosphere at 35 ºC for 0.5 h, then N,N-dibenzyl-3-bromopropan-1-amine (95.5 mg, 0.3 mmol) were added under N_2, stirring at 35 ºC for another 36 h.

The crude material was purified by flash chromatography (Petroleum ether: EtOAc = 100: 1+1% Et_3N) to provide the title compound as a clear pale yellow oil (49.5 mg, 36% yield).

^{1}H NMR (400 MHz, $CDCl_3$) δ: 7.36-7.27 (m, 8H), 7.23-7.19 (m, 2H), 4.10-4.05 (m, 2H), 3.53 (s, 4H), 2.38 (t, *J* = 7.2 Hz, 2H), 2.03 (dd, *J* = 11.5 Hz, 3.2 Hz, 1H), 1.81-1.72 (m, 1H), 1.53-1.46 (m, 2H), 1.41-1.26 (m, 3H), 1.22 (t, *J* = 7.2 Hz, 3H), 0.18 (s, 9H); **^{13}C NMR (100 MHz, $CDCl_3$)** δ: 175.69, 139.93, 128.68, 128.07, 126.65, 59.46, 58.18, 53.09, 36.96, 27.79, 26.86, 26.65, 14.53, -2.93.

HRMS (ESI): $[M+H]^+$ calculated for $C_{25}H_{37}GeNO_2$ = 458.2114, found: 458.2109.

Ethyl 6-(4,4,5,5-tetramethyl-1,3,2-dioxaborolan-2-yl)-2-(trimethylgermyl)hexanoate (31)

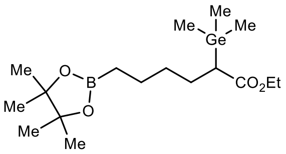

In an oven-dried 8 mL reaction vial equipped with a magnetic stir bar, ethyl acrylate (48.9 μL, 0.45 mmol), trimethylchlorogermane (56.4 μL, 0.45 mmol), $NiBr_2$ (6.6 mg, 0.03 mmol), Mn (49.5 mg, 0.9 mmol), DMA (3.0 mL) were charged under N_2 atmosphere at 35 ºC for 0.5 h, then 2-(3-bromopropyl)-4,4,5,5-tetramethyl-1,3,2-dioxaborolane (74.7 mg, 0.3 mmol) were added under N_2, stirring at 35 ºC for another 36 h.

The crude material was purified by flash chromatography (Petroleum ether: EtOAc = 30: 1) to provide the title compound as a clear pale yellow oil (64.0 mg, 55% yield).

^{1}H NMR (400 MHz, CDCl$_3$) δ: 4.11-4.05 (m, 2H), 2.06 (dd, J = 11.4 Hz, 3.1Hz, 1H), 1.85-1.77 (m, 1H), 1.47-1.31 (m, 4H), 1.27-1.19 (m, 16H), 0.75 (t, J = 8.0 Hz, 2H), 0.19 (s, 9H); **^{13}C NMR (100 MHz, CDCl$_3$)** δ: 175.75, 82.84, 59.44, 36.99, 32.95, 26.83, 24.79, 24.78, 23.79, 14.54, -2.90.

HRMS (ESI): $[M+H]^+$ calculated for $C_{17}H_{35}BGeO_4$ = 389.1918, found: 383.1912.

Ethyl 4-ethyl-2-(trimethylgermyl)hexanoate (32)

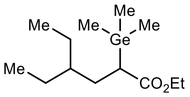

In an oven-dried 8 mL reaction vial equipped with a magnetic stir bar, ethyl acrylate (48.9 μL, 0.45 mmol), trimethylchlorogermane (56.4 μL, 0.45 mmol), $NiBr_2$ (6.6 mg, 0.03 mmol), Mn (49.5 mg, 0.9 mmol), DMA (3.0 mL) were charged under N_2 atmosphere at 35 ºC for 0.5 h, then 3-bromopentane (45.3 mg, 0.3 mmol) were added under N_2, stirring at 35 ºC for another 36 h.

The crude material was purified by flash chromatography (Petroleum ether: EtOAc = 50: 1) to provide the title compound as a clear yellow oil (66.2 mg, 76% yield).

^{1}H NMR (400 MHz, CDCl$_3$) δ: 4.12-4.06 (m, 2H), 2.18 (dd, J = 12.4 Hz, 2.8 Hz, 1H), 1.91-1.85 (m, 1H), 1.37-1.19 (m, 9H), 0.85 (t, J = 7.3 Hz, 3H), 0.80 (t, J = 7.4 Hz, 3H), 0.21 (s, 9H); **^{13}C NMR (100 MHz, CDCl$_3$)** δ: 175.89, 59.49, 40.50, 34.56, 30.04, 25.58, 24.16, 14.54, 11.02, 10.15, -2.97.

HRMS (ESI): $[M+H]^+$ calculated for $C_{13}H_{28}GeO_2$ = 291.1379, found: 291.1374.

Ethyl 3-cyclobutyl-2-(trimethylgermyl)propanoate (33)

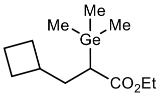

In an oven-dried 8 mL reaction vial equipped with a magnetic stir bar, ethyl acrylate (48.9 μL, 0.45 mmol), trimethylchlorogermane (56.4 μL, 0.45 mmol), $NiBr_2$ (6.6 mg, 0.03 mmol), Mn (49.5 mg, 0.9 mmol), DMA (3.0 mL) were charged under N_2 atmosphere at 35 ºC for 0.5 h, then cyclobutyl bromide (40.5 mg, 0.3 mmol) were added under N_2, stirring at 35 ºC for another 36 h.

The crude material was purified by flash chromatography (Petroleum ether: EtOAc = 50: 1) to provide the title compound as a clear yellow oil (70.4 mg, 86% yield).

^{1}H NMR (400 MHz, $CDCl_3$) δ: 4.11-4.05 (m, 2H), 2.30-2.22 (m, 1H), 1.99-1.93 (m, 4H), 1.84-1.72 (m, 2H), 1.59-1.49 (m, 2H), 1.40-1.35 (m, 1H), 1.23 (t, J = 7.2 Hz, 3H), 0.20 (s, 9H); **^{13}C NMR (100 MHz, $CDCl_3$)** δ: 175.78, 59.48, 36.39, 34.68, 33.98, 28.22, 27.84, 18.16, 14.52, -3.02.

HRMS (ESI): $[M+H]^+$ calculated for $C_{12}H_{24}GeO_2$ = 275.1066, found: 275.1060.

Ethyl 3-cyclopentyl-2-(trimethylgermyl)propanoate (34)

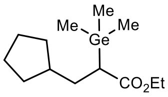

In an oven-dried 8 mL reaction vial equipped with a magnetic stir bar, ethyl acrylate (48.9 μL, 0.45 mmol), trimethylchlorogermane (56.4 μL, 0.45 mmol), $NiBr_2$ (6.6 mg, 0.03 mmol), Mn (49.5 mg, 0.9 mmol), DMA (3.0 mL) were charged under N_2 atmosphere at 35 ºC for 0.5 h, then bromocyclopentane (44.7 mg, 0.3 mmol) were added under N_2, stirring at 35 ºC for another 36 h.

The crude material was purified by flash chromatography (Petroleum ether: EtOAc = 50: 1) to provide the title compound as a clear yellow oil (66.2 mg, 76% yield).

^{1}H NMR (400 MHz, $CDCl_3$) δ: 4.12-4.07 (m, 2H), 2.14 (dd, J = 11.7 Hz, 3.2 Hz, 1H), 2.00-1.93 (m, 1H), 1.79-1.71 (m, 3H) 1.57-1.47 (m, 4H), 1.28-1.22 (m, 4H), 1.09-0.97 (m, 2H), 0.20 (s, 9H); **^{13}C NMR (100 MHz, $CDCl_3$)** δ: 175.94, 59.48, 40.59, 36.19, 33.08, 32.78, 31.96, 25.11, 24.96, 14.54, -3.00.

HRMS (ESI): $[M+H]^+$ calculated for $C_{13}H_{26}GeO_2$ = 289.1223, found: 289.1218.

Ethyl 3-cyclohexyl-2-(trimethylgermyl)propanoate (35)

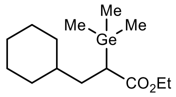

In an oven-dried 8 mL reaction vial equipped with a magnetic stir bar, ethyl acrylate (48.9 μL, 0.45 mmol), trimethylchlorogermane (56.4 μL, 0.45 mmol), $NiBr_2$ (6.6 mg, 0.03 mmol), Mn (49.5 mg, 0.9 mmol), DMA (3.0 mL) were charged under N_2 atmosphere at 35 ºC for 0.5 h, then bromocyclohexane (48.9 mg, 0.3 mmol) were added under N_2, stirring at 35 ºC for another 36 h.

The crude material was purified by flash chromatography (Petroleum ether: EtOAc = 50: 1) to provide the title compound as a clear yellow oil (76.6 mg, 85% yield).

^{1}H NMR (400 MHz, $CDCl_3$) δ: 4.12-4.06 (m, 2H), 2.20 (dd, *J* = 11.9 Hz, 3.0 Hz, 1H), 1.86-1.73 (m, 6H), 1.23 (t, *J* = 7.1 Hz, 3H), 1.19-1.09 (m, 5H), 0.92-0.72 (m, 2H), 0.20 (s, 9H); **^{13}C NMR (100 MHz, $CDCl_3$)** δ: 175.96, 59.46, 37.89, 34.38, 34.22, 33.64, 32.30, 26.57, 26.27, 26.18, 14.52, -3.01.

HRMS (ESI): $[M+H]^+$ calculated for $C_{14}H_{28}GeO_2$ = 303.1379, found: 303.1374.

Ethyl 3-((1r,3r,5r,7r)-adamantan-2-yl)-2-(trimethylgermyl)propanoate (36)

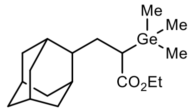

In an oven-dried 8 mL reaction vial equipped with a magnetic stir bar, ethyl acrylate (48.9 μL, 0.45 mmol), trimethylchlorogermane (56.4 μL, 0.45 mmol), $NiBr_2$ (6.6 mg, 0.03 mmol), Mn (49.5 mg, 0.9 mmol), DMA (3.0 mL) were charged under N_2 atmosphere at 35 ºC for 0.5 h, then 2-bromoadamantane (64.5 mg, 0.3 mmol) were added under N_2, stirring at 35 ºC for another 36 h.

The crude material was purified by flash chromatography (Petroleum ether: EtOAc = 70: 1) to provide the title compound as a clear pale yellow oil (101.6 mg, 95% yield).

^{1}H NMR (400 MHz, $CDCl_3$) δ: 4.11-4.06 (m, 2H), 2.12 (dd, *J* = 11.6 Hz, 2.8 Hz, 1H), 1.97-1.77 (m, 7H), 1.70-1.64 (m, 7H), 1.60-1.57 (m, 1H), 1.50-1.46 (m, 2H), 1.23 (t, *J* = 7.2 Hz, 3H), 0.22 (s, 9H); **^{13}C NMR (100 MHz, $CDCl_3$)** δ: 175.95, 59.49, 44.54, 39.15, 39.14, 38.34, 34.86, 32.46, 31.65, 31.42, 30.54, 29.58, 28.24, 28.03, 14.54, -2.94.

HRMS (ESI): $[M+H]^+$ calculated for $C_{18}H_{32}GeO_2$ = 355.1692, found: 355.1687.

Ethyl 3-(oxetan-3-yl)-2-(trimethylgermyl)propanoate (37)

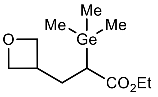

In an oven-dried 8 mL reaction vial equipped with a magnetic stir bar, ethyl acrylate (48.9 μL, 0.45 mmol), trimethylchlorogermane (56.4 μL, 0.45 mmol), $NiBr_2$ (6.6 mg, 0.03 mmol), Mn (49.5 mg, 0.9 mmol), DMA (3.0 mL) were charged under N_2 atmosphere at 35 ºC for 0.5 h, then 3-bromooxetane (41.1 mg, 0.3 mmol) were added under N_2, stirring at 35 ºC for another 36 h.

The crude material was purified by flash chromatography (Petroleum ether: EtOAc = 15: 1) to provide the title compound as a clear yellow oil (53.3 mg, 64% yield).

^{1}H NMR (400 MHz, $CDCl_3$) δ: 4.77-4.73 (m, 2H), 4.38-4.29 (m, 2H), 4.11-4.05 (m, 2H), 3.01-2.94 (m, 1H), 2.24-2.16 (m, 1H), 1.99 (dd, J = 12.0 Hz, 3.0 Hz, 1H), 1.81-1.74 (m, 1H), 1.23 (t, J = 7.8 Hz, 3H), 0.24 (s, 9H); **^{13}C NMR (100 MHz, $CDCl_3$)** δ: 175.07, 77.39, 77.16, 59.76, 35.39, 34.30, 30.89, 14.45, -2.98.

HRMS (ESI): $[M+H]^+$ calculated for $C_{11}H_{22}GeO_3$ = 277.0859, found: 277.0854.

Ethyl 3-(tetrahydro-2H-pyran-4-yl)-2-(trimethylgermyl)propanoate (38)

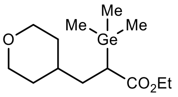

In an oven-dried 8 mL reaction vial equipped with a magnetic stir bar, ethyl acrylate (48.9 μL, 0.45 mmol), trimethylchlorogermane (56.4 μL, 0.45 mmol), $NiBr_2$ (6.6 mg, 0.03 mmol), Mn (49.5 mg, 0.9 mmol), DMA (3.0 mL) were charged under N_2 atmosphere at 35 ºC for 0.5 h, then 4-bromo-tetrahydropyran (49.5 mg, 0.3 mmol) were added under N_2, stirring at 35 ºC for another 36 h.

The crude material was purified by flash chromatography (Petroleum ether: EtOAc = 12: 1) to provide the title compound as a clear yellow oil (70.9 mg, 77% yield).

^{1}H NMR (400 MHz, $CDCl_3$) δ: 4.13-4.07 (m, 2H), 3.96-3.90 (m, 2H), 3.37-3.30 (m, 2H), 2.20 (dd, J = 12.1 Hz, 2.9 Hz, 1H), 1.95-1.87 (m, 1H), 1.64-1.45 (m, 2H), 1.32-1.10 (m, 6H), 0.21 (s, 9H); **^{13}C NMR (100 MHz, $CDCl_3$)** δ: 172.68, 67.98, 60.03, 35.13, 33.90, 33.64, 33.38, 32.14, 14.50, -3.02

HRMS (ESI): $[M+H]^+$ calculated for $C_{13}H_{26}GeO_3$ = 305.1172, found: 305.1166.

Ethyl 3-(1-tosylpiperidin-4-yl)-2-(trimethylgermyl)propanoate (39)

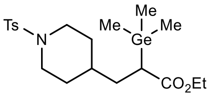

In an oven-dried 8 mL reaction vial equipped with a magnetic stir bar, ethyl acrylate (48.9 μL, 0.45 mmol), trimethylchlorogermane (56.4 μL, 0.45 mmol), $NiBr_2$ (6.6 mg, 0.03 mmol), Mn (49.5 mg, 0.9 mmol), DMA (3.0 mL) were charged under N_2 atmosphere at 35 ºC for 0.5 h, then 4-bromo-1-tosylpiperidine (95.0 mg, 0.3 mmol) were added under N_2, stirring at 35 ºC for another 36 h.

The crude material was purified by flash chromatography (Petroleum ether: EtOAc = 20: 1) to provide the title compound as white solid (109.3 mg, 80% yield), melting point: 120 -122ºC.

^{1}H NMR (400 MHz, CDCl3) δ: 7.62 (d, *J* = 8.2 Hz, 2H), 7.31 (d, *J* = 8.2 Hz, 2H), 4.08-3.99 (m, 2H), 3.78-3.73 (m, 2H), 2.43 (s, 3H), 2.19-2.13 (m, 3H), 1.91-1.85 (m, 1H), 1.77-1.75 (m, 1H), 1.68-1.65 (m, 1H), 1.42-1.28 (m, 2H), 1.20 (t, *J* = 7.1 Hz, 3H), 1.14-1.09 (m, 2H), 0.19 (m, 9H); **^{13}C NMR (100 MHz, CDCl3)** δ: 175.30, 143.27, 132.80, 129.44, 127.60, 59.53, 46.33, 35.07, 33.63, 33.00, 31.83, 30.30, 21.40, 14.40, -3.10.

HRMS (ESI): $[M+H]^+$ calculated for $C_{20}H_{33}GeNO_4S$ = 458.1420, found: 458.1415.

Ethyl 4,4-dimethyl-2-(trimethylgermyl)pentanoate (40)

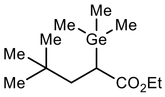

In an oven-dried 8 mL reaction vial equipped with a magnetic stir bar, ethyl acrylate (48.9 μL, 0.45 mmol), trimethylchlorogermane (56.4 μL, 0.45 mmol), $NiBr_2$ (6.6 mg, 0.03 mmol), Mn (49.5 mg, 0.9 mmol), DMA (3.0 mL) were charged under N_2 atmosphere at 35 ºC for 0.5 h, then 2-bromo-2-methylpropane (41.1 mg, 0.3 mmol) were added under N_2, stirring at 35 ºC for another 36 h.

The crude material was purified by flash chromatography (Petroleum ether: EtOAc = 80: 1) to provide the title compound as a clear pale yellow oil (57.5 mg, 69% yield).

^{1}H NMR (400 MHz, CDCl3) δ: 4.14-4.03 (m, 2H), 2.12 (d, *J* = 11.5 Hz, 1H), 2.01-1.95 (m, 1H), 1.29-1.22 (m, 4H), 0.84 (s, 9H), 0.20 (s, 9H); **^{13}C NMR (100 MHz, CDCl3)** δ: 173.59, 59.51, 41.07, 33.97, 32.03, 28.85, 14.49, -3.20.

HRMS (ESI): $[M+H]^+$ calculated for $C_{12}H_{26}GeO_2$ = 277.1223, found: 277.1218.

Ethyl 4,4-dimethyl-2-(trimethylgermyl)hexanoate (41)

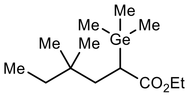

In an oven-dried 8 mL reaction vial equipped with a magnetic stir bar, ethyl acrylate (48.9 μL, 0.45 mmol), trimethylchlorogermane (56.4 μL, 0.45 mmol), $NiBr_2$ (6.6 mg, 0.03 mmol), Mn (49.5 mg, 0.9 mmol), DMA (3.0 mL) were charged under N_2 atmosphere at 35 °C for 0.5 h, then 2-bromo-2-methylbutane (45.3 mg, 0.3 mmol) were added under N_2, stirring at 35 °C for another 36 h.

The crude material was purified by flash chromatography (Petroleum ether: EtOAc = 100: 1) to provide the title compound as a clear pale yellow oil (59.6 mg, 68% yield).

^{1}H NMR (400 MHz, $CDCl_3$) δ: 4.11-4.05 (m, 2H), 2.11 (dd, J = 11.4 Hz, 0.8 Hz, 1H), 1.98-1.92 (m, 1H), 1.25-1.17 (m, 6H), 0.78-0.77 (m, 9H), 0.20 (s, 9H); **^{13}C NMR (100 MHz, $CDCl_3$)** δ: 176.61, 59.52, 38.23, 34.48, 33.71, 32.52, 26.02, 25.79, 14.49, 8.36, -3.15.

HRMS (ESI): $[M+H]^+$ calculated for $C_{13}H_{28}GeO_2$ = 291.1382, found: 291.1374.

Ethyl 4-ethyl-4-methyl-2-(trimethylgermyl)octanoate (42)

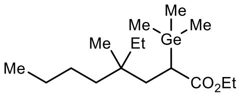

In an oven-dried 8 mL reaction vial equipped with a magnetic stir bar, ethyl acrylate (48.9 μL, 0.45 mmol), trimethylchlorogermane (56.4 μL, 0.45 mmol), $NiBr_2$ (6.6 mg, 0.03 mmol), Mn (49.5 mg, 0.9 mmol), DMA (3.0 mL) were charged under N_2 atmosphere at 35 °C for 0.5 h, then 3-bromo-3-methylheptane (57.6 mg, 0.3 mmol) were added under N_2, stirring at 35 °C for another 36 h.

The crude material was purified by flash chromatography (Petroleum ether: EtOAc = 80: 1) to provide the title compound as a clear pale yellow oil (70.2 mg, 70% yield).

^{1}H NMR (400 MHz, $CDCl_3$) δ: 4.13-4.02 (m, 2H), 2.09 (dd, J = 11.4 Hz, 0.8 Hz, 1H), 1.97-1.91 (m, 1H), 1.24 (t, J = 7.1 Hz, 6H), 1.20-1.11 (m, 6H), 0.88 (t, J = 7.3 Hz, 3H), 0.77-0.72 (m, 6H), 0.20 (s, 9H); **^{13}C NMR (100 MHz, $CDCl_3$)** δ: 176.60, 59.51, 38.01, 37.83, 36.67, 35.89, 32.14, 30.92, 30.71, 25.72, 25.64, 23.85, 23.75, 23.63, 14.48, 14.16, 7.99, 7.90, -3.14.

HRMS (ESI): $[M+H]^+$ calculated for $C_{16}H_{34}GeO_2$= 333.1849, found: 333.1844.

6-ethoxy-3,3-dimethyl-6-oxo-5-(trimethylgermyl)hexyl 4-fluorobenzoate (43)

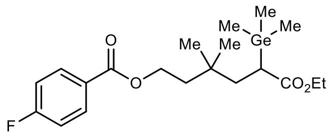

In an oven-dried 8 mL reaction vial equipped with a magnetic stir bar, ethyl acrylate (48.9 μL, 0.45 mmol), trimethylchlorogermane (56.4 μL, 0.45 mmol), $NiBr_2$ (6.6 mg, 0.03 mmol), Mn (49.5 mg, 0.9 mmol), DMA (3.0 mL) were charged under N_2 atmosphere at 35 ºC for 0.5 h, then 3-bromo-3-methylbutyl 4-fluorobenzoate (86.4 mg, 0.3 mmol) were added under N_2, stirring at 35 ºC for another 36 h.

The crude material was purified by flash chromatography (Petroleum ether: EtOAc = 30: 1) to provide the title compound as a clear pale yellow oil (98.8 mg, 77% yield).

^{1}H NMR (400 MHz, $CDCl_3$) δ: 8.06-8.02 (m, 2H), 7.10 (t, J = 8.7 Hz, 2H), 4.34 (t, J = 11.9 Hz, 2H), 4.15-4.03 (m, 2H), 2.18-2.06 (m, 2H), 1.70-1.64 (m, 2H), 1.31-1.27 (m, 1H), 1.23 (t, J = 7.1 Hz, 3H), 0.91 (s, 6H), 0.21 (s, 9H); **^{13}C NMR (100 MHz, $CDCl_3$)** δ: 176.33, 165.64, 165.64 (d, J = 252.3 Hz), 132.00 (d, J = 9.2 Hz), 126.61, 115.42 (d, J = 21.8 Hz), 62.36, 59.67, 39.68, 39.17, 33.99, 32.41, 26.47, 26.36, 14.46, -3.16; **^{19}F NMR (376 MHz, $CDCl_3$)** δ: -105.87

HRMS (ESI): $[M+H]^+$ calculated for $C_{20}H_{31}FGeO_4$ = 429.1496, found: 429.1491.

Ethyl 3-(1-methylcyclohexyl)-2-(trimethylgermyl)propanoate (44)

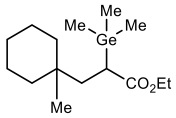

In an oven-dried 8 mL reaction vial equipped with a magnetic stir bar, ethyl acrylate (48.9 μL, 0.45 mmol), trimethylchlorogermane (56.4 μL, 0.45 mmol), $NiBr_2$ (6.6 mg, 0.03 mmol), Mn (49.5 mg, 0.9 mmol), DMA (3.0 mL) were charged under N_2 atmosphere at 35 ºC for 0.5 h, then 1-bromo-1-methylcyclohexane (52.8 mg, 0.3 mmol) were added under N_2, stirring at 35 ºC for another 36 h.

The crude material was purified by flash chromatography (Petroleum ether: EtOAc = 70: 1) to provide the title compound as a clear pale yellow oil (73.8 mg, 78% yield).

^{1}H NMR (400 MHz, $CDCl_3$) δ: 4.13-4.03 (m, 2H), 2.14 (dd, J = 11.4 Hz, 0.6 Hz, 1H), 2.03-1.96 (m, 1H), 1.47-1.35 (m, 5H), 1.23 (t, J = 7.1 Hz, 6H), 1.20-1.15 (m, 3H), 0.79 (s, 3H), 0.20 (s, 9H); **^{13}C NMR (100 MHz, $CDCl_3$)** δ: 176.68, 59.51, 37.42, 37.12, 34.31, 31.86, 26.45, 22.05, 21.99, 14.49, -3.15.

HRMS (ESI): $[M+H]^+$ calculated for $C_{15}H_{30}GeO_2$= 317.1536, found: 317.1531.

Ethyl 3-((3r,5r,7r)-adamantan-1-yl)-2-(trimethylgermyl)propanoate (45)

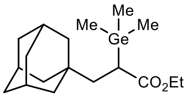

In an oven-dried 8 mL reaction vial equipped with a magnetic stir bar, ethyl acrylate (48.9 μL, 0.45 mmol), trimethylchlorogermane (56.4 μL, 0.45 mmol), $NiBr_2$ (6.6 mg, 0.03 mmol), Mn (49.5 mg, 0.9 mmol), DMA (3.0 mL) were charged under N_2 atmosphere at 35 ºC for 0.5 h, then 1-bromoadamantanee (64.5 mg, 0.3 mmol) were added under N_2, stirring at 35 ºC for another 36 h.

The crude material was purified by flash chromatography (Petroleum ether: EtOAc = 70: 1) to provide the title compound as a clear pale yellow oil (66.9 mg, 63% yield).

^{1}H NMR (400 MHz, $CDCl_3$) δ: 4.12-4.05 (m, 2H), 2.17 (dd, *J* = 11.5 Hz, 1.0 Hz, 1H), 1.92-1.89 (m, 3H), 1.68-1.57 (m, 7H), 1.49-1.46 (m, 3H), 1.36-1.32 (m, 3H), 1.24 (t, *J* = 7.2 Hz, 3H), 1.06-1.02 (m, 1H), 0.19 (s, 9H); **^{13}C NMR (100 MHz, $CDCl_3$)** δ: 176.63, 59.52, 41.81, 41.03, 37.04, 33.76, 30.86, 28.62, 14.52, -3.13.

HRMS (ESI): $[M+H]^+$ calculated for $C_{18}H_{32}GeO_2$= 355.1692, found: 355.1686.

Benzyl 6-phenyl-2-(trimethylgermyl)hexanoate (46)

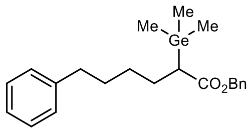

In an oven-dried 8 mL reaction vial equipped with a magnetic stir bar, benzyl acrylate (67.6 μL, 0.45 mmol), trimethylchlorogermane (56.4 μL, 0.45 mmol), $NiBr_2$ (6.6 mg, 0.03 mmol), Mn (49.5 mg, 0.9 mmol), DMA (3.0 mL) were charged under N_2 atmosphere at 35 ºC for 0.5 h, then (3-bromopropyl)benzene (59.7 mg, 0.3 mmol) were added under N_2, stirring at 35 ºC for another 36 h.

The crude material was purified by flash chromatography (Petroleum ether: EtOAc = 50: 1) to provide the title compound as clear pale yellow oil (91.2 mg, 76% yield).

^{1}H NMR (400 MHz, $CDCl_3$) δ: 7.36-7.28 (m, 6H), 7.25-7.15 (m, 4H), 5.08 (d, *J* = 6.5 Hz, 2H), 2.62-2.52 (m, 2H), 2.14 (dd, *J* = 11.6 Hz, 3.2 Hz, 1H), 1.93-1.84 (m, 1H), 1.62-1.60 (m, 1H), 1.43-1.22 (m, 4H), 0.16 (s, 9H); **^{13}C NMR (100 MHz, $CDCl_3$)** δ: 175.66, 142.62, 136.48, 128.44, 128.37, 128.22, 128.03, 125.59, 65.67, 36.96, 35.77, 31.22, 29.97, 26.94, -2.84.

HRMS (ESI): $[M+H]^+$ calculated for $C_{22}H_{30}GeO_2$= 401.1536, found: 401.1531.

Cyclohexyl 6-phenyl-2-(trimethylgermyl)hexanoate (47)

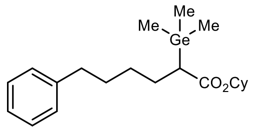

In an oven-dried 8 mL reaction vial equipped with a magnetic stir bar, cyclohexyl acrylate (71.2 μL, 0.45 mmol), trimethylchlorogermane (56.4 μL, 0.45 mmol), $NiBr_2$ (6.6 mg, 0.03 mmol), Mn (49.5 mg, 0.9 mmol), DMA (3.0 mL) were charged under N_2 atmosphere at 35 ºC for 0.5 h, then (3-bromopropyl)benzene (59.7 mg, 0.3 mmol) were added under N_2, stirring at 35 ºC for another 36 h.

The crude material was purified by flash chromatography (Petroleum ether: EtOAc = 80: 1) to provide the title compound as clear pale yellow oil (76.3 mg, 65% yield).

^{1}H NMR (400 MHz, $CDCl_3$) δ: 7.30-7.24 (m, 2H), 7.18-7.15 (m, 3H), 4.77-4.71 (m, 1H), 2.61-2.56 (m, 2H), 2.04 (dd, J = 11.7 Hz, J = 3.1 Hz, 1H), 1.87-1.59 (m, 7H), 1.44-1.25 (m, 9H), -0.2 (s, 9H); **^{13}C NMR (100 MHz, $CDCl_3$)** δ: 175.13, 142.67, 128.37, 128.20, 125.57, 71.76, 37.22, 35.79, 32.09, 32.01, 31.22, 29.88, 26.98, 25.42, 23.91, -2.89.

HRMS (ESI): $[M+H]^+$ calculated for $C_{21}H_{34}GeO_2$= 393.1849, found: 393.1844.

Tert-butyl 6-phenyl-2-(trimethylgermyl)hexanoate (48)

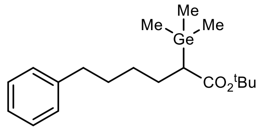

In an oven-dried 8 mL reaction vial equipped with a magnetic stir bar, tert-butyl acrylate (65.3 μL, 0.45 mmol), trimethylchlorogermane (56.4 μL, 0.45 mmol), $NiBr_2$ (6.6 mg, 0.03 mmol), Mn (49.5 mg, 0.9 mmol), DMA (3.0 mL) were charged under N_2 atmosphere at 35 ºC for 0.5 h, then (3-bromopropyl)benzene (59.7 mg, 0.3 mmol) were added under N_2, stirring at 35 ºC for another 36 h.

The crude material was purified by flash chromatography (Petroleum ether: EtOAc = 80: 1) to provide the title compound as clear pale yellow oil (67.3 mg, 61% yield).

^{1}H NMR (400 MHz, $CDCl_3$) δ: 7.28-7.25 (m, 2H), 7.18-7.16 (m, 3H), 2.62-2.57 (m, 2H), 1.96 (dd, J = 11.5 Hz, 3.0 Hz, 1H), 1.86-1.75 (m, 1H), 1.66-1.60 (m, 2H), 1.41 (s, 9H), 1.36-1.25 (m, 3H), 0.2 (s, 9H); **^{13}C NMR (100 MHz, $CDCl_3$)** δ: 175.03, 142.68, 128.36, 128.19, 125.54, 79.24, 37.89, 35.77, 31.25, 29.81, 28.32, 27.02, -2.89.

HRMS (ESI): $[M+H]^+$ calculated for $C_{19}H_{32}GeO_2$= 367.1692, found: 367.1687.

N,6-diphenyl-2-(trimethylgermyl)hexanamide (49)

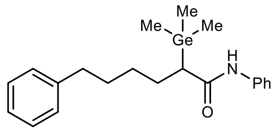

In an oven-dried 8 mL reaction vial equipped with a magnetic stir bar, N-Phenylacrylamide (8.1 mg, 0.45 mmol), trimethylchlorogermane (56.4 μL, 0.45 mmol), $NiBr_2$ (6.6 mg, 0.03 mmol), (4R,4'R)-4,4'-diisopropyl-4,4',5,5'-tetrahydro-2,2'-bioxazole (0.036mmol, 8.1 mg), Mn (49.5 mg, 0.9 mmol), DMA (3.0 mL) were charged under N_2 atmosphere at 35 ºC for 0.5 h, then (3-bromopropyl)benzene (59.7 mg, 0.3 mmol) were added under N_2, stirring at 35 ºC for another 36 h.

The crude material was purified by flash chromatography (Petroleum ether: EtOAc = 20: 1) to provide the title compound as clear pale yellow oil (38.2 mg, 33% yield).

^{1}H NMR (400 MHz, $CDCl_3$) δ: 7.47 (d, J = 8.0 Hz, 2H), 7.32-7.24 (m, 4H), 7.17-7.15 (m, 3H), 7.08 (t, J = 7.3 Hz, 1H), 6.89 (s, 1H), 2.65-2.55 (m, 2H), 2.05-1.92 (m, 2H), 1.68-1.64 (m, 1H), 1.53-1.43 (m, 2H), 1.37-1.25 (m, 2H), 0.25 (s, 9H); **^{13}C NMR (100 MHz, $CDCl_3$)** δ: 173.47, 142.59, 138.15, 128.92, 128.37, 128.22, 125.60, 123.74, 120.15, 40.54, 35.74, 31.34, 29.95, 27.73, 27.30, -2.87.

HRMS (ESI): $[M+H]^+$ calculated for $C_{21}H_{29}GeNO$= 386.1539, found: 386.1534.

6-phenyl-2-(trimethylgermyl)hexanenitrile (50)

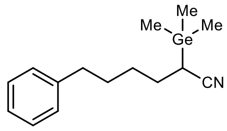

In an oven-dried 8 mL reaction vial equipped with a magnetic stir bar, acrylonitrile (29.6 μL, 0.45 mmol), trimethylchlorogermane (56.4 μL, 0.45 mmol), $NiBr_2$ (9.9 mg, 0.045 mmol), (4R,4'R)-4,4'-diisopropyl-4,4',5,5'-tetrahydro-2,2'-bioxazole (0.054mmol, 12.2 mg), Mn (49.5 mg, 0.9 mmol), DMA (3.0 mL) were charged under N_2 atmosphere at 35 ºC for 0.5 h, then (3-bromopropyl)benzene (59.7 mg, 0.3 mmol) were added under N_2, stirring at 35 ºC for another 60 h.

The crude material was purified by flash chromatography (Petroleum ether: EtOAc = 20: 1) to provide the title compound as clear pale yellow oil (59.2 mg, 68% yield).

^{1}H NMR (400 MHz, $CDCl_3$) δ: 7.30-7.26 (m, 2H), 7.20-7.16 (m, 3H), 2.63 (t, J = 7.9 Hz, 2H), 1.83 (dd, J = 10.7 Hz, J = 4.2 Hz, 1H), 1.68-1.59 (m, 4H), 1.53-1.43 (m, 2H), 0.33 (s, 9H); **^{13}C NMR (100 MHz, $CDCl_3$)** δ: 142.17, 128.35, 128.32, 125.79, 122.97, 35.66, 30.82, 29.55, 27.30, 17.45, -4.10.

HRMS (ESI): [M+H]$^+$ calculated for $C_{15}H_{23}GeN$= 292.1120, found: 292.1114.

Trimethyl(5-phenyl-1-(phenylsulfonyl)pentyl)germane (51)

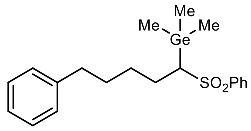

In an oven-dried 8 mL reaction vial equipped with a magnetic stir bar, (vinylsulfonyl)benzene (75.6 mg, 0.45 mmol), trimethylchlorogermane (56.4 µL, 0.45 mmol), $NiBr_2$ (9.9 mg, 0.045 mmol), Mn (49.5 mg, 0.9 mmol), DMA (3.0 mL) were charged under N_2 atmosphere at 35 ºC for 0.5 h, then (3-bromopropyl)benzene (59.7 mg, 0.3 mmol) were added under N_2, stirring at 35 ºC for another 36 h.
The crude material was purified by flash chromatography (Petroleum ether: EtOAc = 10: 1 and DCM: PE=2: 1) to provide the title compound as clear pale yellow oil (45.1 mg, 37% yield).
^{1}H NMR (400 MHz, $CDCl_3$) δ: 7.85-7.83 (m, 2H), 7.58-7.56 (m, 1H), 7.51 (t, J = 7.8 Hz, 2H), 7.24-7.22 (m, 2H), 7.16 (t, J = 7.2 Hz, 1H), 7.02-7.00 (m, 2H), 2.67 (t, J = 5.8 Hz, 1H), 2.39 (t, J = 8.3 Hz, 2H), 1.68-1.63 (m, 2H), 1.41-1.33 (m, 3H), 1.20-1.17 (m, 1H), 0.44 (s, 9H); **^{13}C NMR (100 MHz, $CDCl_3$)** δ: 141.87, 140.80, 132.82, 128.94, 128.25, 128.23, 127.85, 125.74, 56.35, 35.22, 30.92, 28.02, 26.90, -0.48.
HRMS (ESI): [M+H]+ calculated for $C_{20}H_{28}GeO_2S$= 406.1022, found: 406.1016.

Diethyl (5-phenyl-1-(trimethylgermyl)pentyl)phosphonate (52)

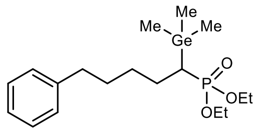

In an oven-dried 8 mL reaction vial equipped with a magnetic stir bar, diethyl vinylphosphonate (69.2 µL, 0.45 mmol), trimethylchlorogermane (56.4 µL, 0.45 mmol), $NiBr_2$ (9.9 mg, 0.045 mmol), Mn (49.5 mg, 0.9 mmol), DMA (3.0 mL) were charged under N_2 atmosphere at 35 ºC for 0.5 h, then (3-bromopropyl)benzene (59.7 mg, 0.3 mmol) were added under N_2, stirring at 35 ºC for another 36 h.
The crude material was purified by flash chromatography (Petroleum ether: EtOAc = 2: 1) to provide the title compound as clear yellow oil (41.0 mg, 34% yield).
^{1}H NMR (400 MHz, $CDCl_3$) δ: 7.30-7.25 (m, 2H), 7.18-7.15 (m, 3H), 4.10-3.98 (m, 4H), 2.61 (t, J = 7.4 Hz, 2H), 1.84-1.75 (m, 1H), 1.58-1.32 (m, 6H), 1.28 (t, J = 7.2 Hz, 6H), 0.26 (s, 9H); **^{13}C NMR (100 MHz, $CDCl_3$)** δ: 142.02, 128.36, 128.22, 125.62,

60.97, 35.67, 31.42, 30.32, 30.24, 25.55, 16.49, -1.22.

HRMS (ESI): $[M+H]^+$ calculated for $C_{18}H_{33}GeO_3P$= 403.1457, found: 403.1452.

Methyl 2-methyl-6-phenyl-2-(trimethylgermyl)hexanoate (53)

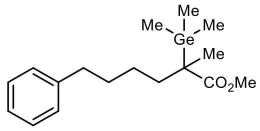

In an oven-dried 8 mL reaction vial equipped with a magnetic stir bar, methyl methacrylate (47.8 µL, 0.45 mmol), trimethylchlorogermane (56.4 µL, 0.45 mmol), $NiBr_2$ (6.6 mg, 0.03 mmol), Mn (49.5 mg, 0.9 mmol), DMA (3.0 mL) were charged under N_2 atmosphere at 35 ºC for 0.5 h, then (3-bromopropyl)benzene (59.7 mg, 0.3 mmol) were added under N_2, stirring at 35 ºC for another 36 h.

The crude material was purified by flash chromatography (Petroleum ether: EtOAc = 60: 1) to provide the title compound as clear yellow oil (49.0 mg, 48% yield).

^{1}H NMR (400 MHz, CDCl$_3$) δ: 7.29-7.25 (m, 2H), 7.18-7.16 (m, 3H), 3.62 (s, 3H), 2.60 (t, J = 8.4 Hz, 2H), 2.01-1.94 (m, 1H), 1.65-1.53 (m, 2H), 1.49-1.23 (m, 3H), 1.19 (s, 3H), 0.15 (s, 9H); **^{13}C NMR (100 MHz, CDCl$_3$)** δ: 177.83, 142.63, 128.35, 128.20, 125.57, 51.04, 38.10, 35.80, 33.93, 31.84, 24.96, 16.68, -4.66.

HRMS (ESI): $[M+H]^+$ calculated for $C_{17}H_{28}GeO_2$= 339.1379, found: 339.1374.

Dimethyl 2-(4-phenylbutyl)-2-(trimethylgermyl)succinate (54)

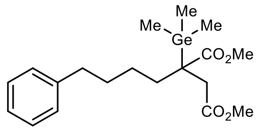

In an oven-dried 8 mL reaction vial equipped with a magnetic stir bar, dimethyl itaconate (63.0 µL, 0.45 mmol), trimethylchlorogermane (56.4 µL, 0.45 mmol), $NiBr_2$ (6.6 mg, 0.03 mmol), Mn (49.5 mg, 0.9 mmol), DMA (3.0 mL) were charged under N_2 atmosphere at 35 ºC for 0.5 h, then (3-bromopropyl)benzene (59.7 mg, 0.3 mmol) were added under N_2, stirring at 35 ºC for another 36 h.

The crude material was purified by flash chromatography (Petroleum ether: EtOAc = 50: 1) to provide the title compound as clear colourless oil (79.6 mg, 67% yield).

^{1}H NMR (400 MHz, CDCl$_3$) δ: 7.26-7.22 (m, 2H), 7.16-7.13 (m, 3H), 3.62 (s, 6H), 2.76 (d, J = 16.5 Hz, 1H), 2.61-2.56 (m, 3H), 1.95 (td, J = 4.8 Hz, 9.3 Hz, 1H), 1.77 (td, J = 4.4 Hz, 12.3 Hz, 1H), 1.63-1.56 (m, 2H), 1.38-1.17 (m, 2H), 0.17 (s, 9H). **^{13}C NMR (100 MHz, CDCl$_3$)** δ: 176.37, 172.53, 142.40, 128.31, 128.17, 125.57, 51.97, 51.21, 40.70,

35.77, 35.59, 32.43, 31.88, 25.50, 2.52.

HRMS (ESI): $[M+Na]^+$ calculated for $C_{19}H_{30}GeO_4$= 419.1248, found: 419.1261.

Ethyl 6-phenyl-2-(triethylgermyl)hexanoate (55)

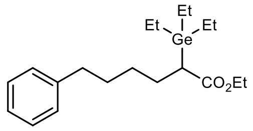

In an oven-dried 8 mL reaction vial equipped with a magnetic stir bar, ethyl acrylate (48.9 µL, 0.45 mmol), triethylgermanium chloride (87.8 mg, 0.45 mmol), $NiBr_2$ (6.6 mg, 0.03 mmol), Mn (49.5 mg, 0.9 mmol), DMA (3.0 mL) were charged under N_2 atmosphere at 35 ºC for 0.5 h, then (3-bromopropyl)benzene (59.7 mg, 0.3 mmol) were added under N_2, stirring at 35 ºC for another 36 h.

The crude material was purified by flash chromatography (Petroleum ether: EtOAc = 40: 1) to provide the title compound as a clear pale yellow oil (88.0 mg, 77% yield).

^{1}H NMR (400 MHz, $CDCl_3$) δ: 7.34-7.30 (m, 2H), 7.23-7.22 (m, 3H), 4.14 (q, *J* = 7.6 Hz, 2H), 2.71-2.59 (m, 2H), 2.24 (dd, *J* = 12.2 Hz, 3.0 Hz, 1H), 2.04-1.95 (m, 1H), 1.71-1.63 (m, 2H), 1.53-1.42 (m, 2H), 1.41-1.25 (m, 5H), 1.10 (t, *J* = 7.9 Hz, 9H), 0.92-0.86 (m, 6H); **^{13}C NMR (100 MHz, $CDCl_3$)** δ: 176.06, 142.58, 128.31, 128.14, 125.51, 59.45, 34.73, 34.06, 31.14, 30.07, 27.06, 14.38, 8.67, 3.70.

HRMS (ESI): $[M+Na]^+$ calculated for $C_{20}H_{34}GeO_2$= 403.1663, found: 403.1666.

Ethyl 6-phenyl-2-(tributylgermyl)hexanoate (56)

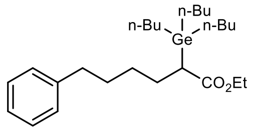

In an oven-dried 8 mL reaction vial equipped with a magnetic stir bar, ethyl acrylate (48.9 µL, 0.45 mmol), tri-n-butylgermanium chloride (125.7 mg, 0.45 mmol), $NiBr_2$ (6.6 mg, 0.03 mmol), Mn (49.5 mg, 0.9 mmol), DMA (3.0 mL) were charged under N_2 atmosphere at 35 ºC for 0.5 h, then (3-bromopropyl)benzene (59.7 mg, 0.3 mmol) were added under N_2, stirring at 35 ºC for another 36 h.

The crude material was purified by flash chromatography (Petroleum ether: EtOAc = 80: 1) to provide the title compound as a clear pale yellow oil (93.4 mg, 67% yield).

^{1}H NMR (400 MHz, $CDCl_3$) δ: 7.30-7.17 (m, 5H), 4.13-4.06 (m, 2H), 2.67-2.55 (m, 2H), 2.17 (dd, *J* = 11.9 Hz, 2.8 Hz, 1H), 1.97-1.92 (m, 1H), 1.69-1.57 (m, 2H), 1.50-1.31 (m, 15H), 1.25 (t, *J* = 7.0 Hz, 3H), 0.92 (t, *J* = 6.9 Hz, 9H), 0.83 (t, *J* = 8.5 Hz,

6H).; **^{13}C NMR (100 MHz, CDCl$_3$)** δ: 176.08, 142.63, 128.33, 128.16, 59.45, 35.75, 34.67, 34.14, 30.08, 27.13 27.04, 26.51, 14.40, 13.65, 12.05.
HRMS (ESI): $[M+Na]^+$ calculated for $C_{26}H_{46}GeO_2$= 487.2602, found: 487.2606.

Ethyl 6-phenyl-2-(trihexylgermyl)hexanoate (57)

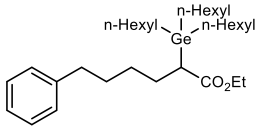

In an oven-dried 8 mL reaction vial equipped with a magnetic stir bar, ethyl acrylate (48.9 μL, 0.45 mmol), tri-n-trihexylgermanium chloride (163.5 mg, 0.45 mmol), $NiBr_2$ (6.6 mg, 0.03 mmol), Mn (49.5 mg, 0.9 mmol), DMA (3.0 mL) were charged under N_2 atmosphere at 35 ºC for 0.5 h, then (3-bromopropyl)benzene (59.7 mg, 0.3 mmol) were added under N_2, stirring at 35 ºC for another 36 h.
The crude material was purified by flash chromatography (Petroleum ether: EtOAc = 100: 1) to provide the title compound as a clear pale yellow oil (101.7 mg, 62% yield).
^{1}H NMR (400 MHz, CDCl$_3$) δ: 7.29-7.25 (m, 2H), 7.18-7.15 (m, 3H), 4.11-4.06 (m, 2H), 2.66-2.54 (m, 2H), 2.16 (dd, *J* = 12.1 Hz, 2.8 Hz, 1H), 1.97-1.88 (m, 1H), 1.66-1.56 (m, 2H), 1.39-1.26 (m, 23H), 1.24 (t, *J* = 7.0 Hz, 6H), 0.92-0.88 (m, 10H), 0.81 (t, *J* = 8.7 Hz, 6H); **^{13}C NMR (100 MHz, CDCl$_3$)** δ: 176.09, 142.64, 128.34, 128.17, 125.54, 59.46, 35.76, 34.69, 33.24, 31.42, 31.15, 30.10, 27.15, 24.80, 22.58, 14.42, 14.07, 12.40.
HRMS (ESI): $[M+H]^+$ calculated for $C_{32}H_{58}GeO_2$= 549.3722, found: 549.3719.

Ethyl 2-(dimethyl(phenyl)germyl)-6-phenylhexanoate (58)

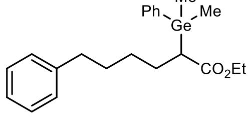

In an oven-dried 8 mL reaction vial equipped with a magnetic stir bar, ethyl acrylate (48.9 μL, 0.45 mmol), chlorodimethylphenylsilane (96.9 mg, 0.45 mmol), $NiBr_2$ (6.6 mg, 0.03 mmol), Mn (49.5 mg, 0.9 mmol), DMA (3.0 mL) were charged under N_2 atmosphere at 35 ºC for 0.5 h, then (3-bromopropyl)benzene (59.7 mg, 0.3 mmol) were added under N_2, stirring at 35 ºC for another 36 h.
The crude material was purified by flash chromatography (Petroleum ether: EtOAc = 60: 1) to provide the title compound as a clear pale yellow oil (93.7 mg, 78% yield).
^{1}H NMR (400 MHz, CDCl$_3$) δ: 7.35-7.34 (m, 2H), 7.26-7.25 (m, 3H), 7.15 (t, *J* = 7.6

Hz, 2H), 7.06-7.01 (m, 3H), 4.00-3.87 (m, 2H), 2.48-2.39 (m, 2H), 2.20 (dd, J = 11.5 Hz, 2.9 Hz, 1H), 1.81-1.72 (m, 1H), 1.48-1.43 (m, 2H), 1.33-1.26 (m, 2H), 1.18-1.12 (m, 1H), 1.02 (t, J = 7.1 Hz, 3H), 0.39 (t, J = 6.6 Hz, 6H).; **^{13}C NMR (100 MHz, $CDCl_3$)** δ: 175.31, 142.49, 138.63, 133.28, 128.80, 128.28, 128.12, 127.97, 125.50, 59.57, 36.95, 34.93, 30.99, 29.72, 27.04, 14.26.
HRMS (ESI): $[M+Na]^+$ calculated for $C_{22}H_{30}GeO_2$= 423.1350, found: 423.1348.

Ethyl 2-(methyldiphenylgermyl)-6-phenylhexanoate (59)

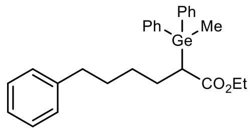

In an oven-dried 8 mL reaction vial equipped with a magnetic stir bar, ethyl acrylate (48.9 μL, 0.45 mmol), chloro(methyl)diphenylgermane (124.8 mg, 0.45 mmol), $NiBr_2$ (6.6 mg, 0.03 mmol), Mn (49.5 mg, 0.9 mmol), DMA (3.0 mL) were charged under N_2 atmosphere at 35 ºC for 0.5 h, then (3-bromopropyl)benzene (59.7 mg, 0.3 mmol) were added under N_2, stirring at 35 ºC for another 36 h.
The crude material was purified by flash chromatography (Petroleum ether: EtOAc = 60: 1) to provide the title compound as a clear colorless oil (121.9 mg, 88% yield).
^{1}H NMR (400 MHz, $CDCl_3$) δ: 7.44-7.41 (m, 4H), 7.29-7.26 (m, 6H), 7.16 (t, J = 7.6 Hz, 2H), 7.08-7.02 (m, 3H), 3.87-3.82 (m, 1H), 3.75-3.70 (m, 1H), 2.59 (dd, J = 11.5 Hz, 3.3 Hz, 1H), 2.49-2.40 (m, 2H), 1.94-1.88 (m, 1H), 1.51-1.31 (m, 4H), 1.22-1.16 (m, 1H), 0.89 (t, J = 7.1 Hz, 3H), 0.67 (s, 3H); **^{13}C NMR (100 MHz, $CDCl_3$)** δ: 175.14, 142.44, 136.75, 136.61, 134.12, 134.09, 129.04, 128.99, 128.28, 128.13, 128.02, 125.51, 59.24, 36.28, 35.57, 30.90, 29.75, 27.50, 13.97.
HRMS (ESI): $[M+Na]^+$ calculated for $C_{27}H_{32}GeO_2$= 485.1506, found: 485.1503.

Ethyl 6-phenyl-2-(triphenylgermyl)hexanoate (60)

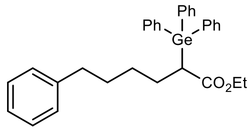

In an oven-dried 8 mL reaction vial equipped with a magnetic stir bar, ethyl acrylate (48.9 μL, 0.45 mmol), triphenylchlorogermane (152.7 mg, 0.45 mmol), $NiBr_2$ (6.6 mg, 0.03 mmol), Mn (49.5 mg, 0.9 mmol), DMA (3.0 mL) were charged under N_2 atmosphere at 35 ºC for 0.5 h, then (3-bromopropyl)benzene (59.7 mg, 0.3 mmol) were added under N_2, stirring at 35 ºC for another 36 h.

The crude material was purified by flash chromatography (Petroleum ether: EtOAc = 100: 1) to provide the title compound as a clear colorless oil (85.9 mg, 55% yield).

^{1}H NMR (400 MHz, CDCl$_3$) δ: 7.52 (d, *J* = 8.6 Hz, 6H), 7.39-7.33 (m, 9H), 7.24-7.20 (m, 2H), 7.15-7.08 (m, 3H), 3.89-3.81 (m, 1H), 3.69-3.61 (m, 1H), 2.92 (dd, *J* = 11.8 Hz, 3.1 Hz, 1H), 2.51 (t, *J* = 7.6 Hz, 2H), 2.13-2.03 (m, 1H), 1.66-1.54 (m, 4H), 1.43-1.26 (m, 1H), 0.88 (t, *J* = 7.2 Hz, 3H); **^{13}C NMR (100 MHz, CDCl$_3$)** δ: 175.11, 142.45, 135.27, 134.89, 129.23, 128.31, 128.15, 125.54, 59.91, 36.45, 35.58, 30.87, 29.92, 28.36, 13.80.

HRMS (ESI): $[M+Na]^+$ calculated for $C_{32}H_{34}GeO_2$= 547.1663, found: 547.1665.

Ethyl (Z)-5-(4-(4-chloro-1,2-diphenylbut-1-en-1-yl)phenoxy)-2-(trimethylgermyl)pentanoate (61)

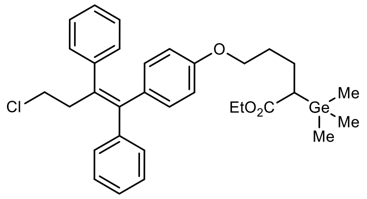

In an oven-dried 8 mL reaction vial equipped with a magnetic stir bar, ethyl acrylate (32.6 μL, 0.3 mmol), trimethylchlorogermane (37.6 μL, 0.3 mmol), $NiBr_2$ (4.4 mg, 0.02 mmol), Mn (33.0 mg, 0.6 mmol), DMA (2.0 mL) were charged under N_2 atmosphere at 35 ºC for 0.5 h, then (Z)-(1-(4-(2-bromoethoxy)phenyl)-4-chlorobut-1-ene-1,2-diyl)dibenzene (88.0 mg, 0.2 mmol) were added under N_2, stirring at 35 ºC for another 36 h.

The crude material was purified by flash chromatography (Petroleum ether: EtOAc = 40: 1) to provide the title compound as a clear pale yellow oil (52.0 mg, 45% yield).

^{1}H NMR (400 MHz, CDCl$_3$) δ: 7.39-7.27 (m, 5H), 7.22-7.13 (m, 5H), 6.77 (d, *J* = 8.8 Hz, 2H), 6.53 (d, *J* = 8.8 Hz, 2H), 4.10-4.02 (m, 2H), 3.85-3.76 (m, 2H), 3.41 (t, *J* = 7.26 Hz, 2H), 2.91 (t, *J* = 7.4 Hz, 2H), 2.09 (dd, *J* = 11.4 Hz, 3.3 Hz, 1H), 1.92-1.75 (m, 2H), 1.69-1.58 (m, 2H), 1.21 (t, *J* = 7.2 Hz, 3H), 0.20 (s, 9H); **^{13}C NMR (100 MHz, CDCl$_3$)** δ: 175.48, 157.20, 142.90, 141.73, 140.96, 134.97, 134.54, 131.63, 129.49, 129.35, 128.28, 128.17, 126.87, 126.51, 113.33, 67.11, 59.59, 42.85, 38.57, 36.50, 29.62, 23.54, 14.51, -2.89.

HRMS (ESI): $[M+Na]^+$ calculated for $C_{32}H_{39}ClGeO_3$= 603.1692, found: 603.1682.

6-Ethoxy-6-oxo-5-(trimethylgermyl)hexyl 4-(N,N-dipropylsulfamoyl)benzoate (62)

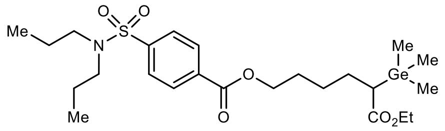

In an oven-dried 8 mL reaction vial equipped with a magnetic stir bar, ethyl acrylate (32.6 μL, 0.3 mmol), trimethylchlorogermane (37.6 μL, 0.3 mmol), $NiBr_2$ (4.4 mg, 0.02 mmol), Mn (33.0 mg, 0.6 mmol), DMA (2.0 mL) were charged under N_2 atmosphere at 35 °C for 0.5 h, then 3-bromopropyl 4-(N,N-dipropylsulfamoyl)benzoate (81.0 mg, 0.2 mmol) were added under N_2, stirring at 35 °C for another 36 h.

The crude material was purified by flash chromatography (Petroleum ether: EtOAc = 10: 1) to provide the title compound as a clear pale yellow oil (69.8 mg, 64% yield).

^{1}H NMR (400 MHz, $CDCl_3$) δ: 8.14 (d, J = 8.4 Hz, 2H), 7.86 (d, J = 8.3 Hz, 2H), 4.33 (dt, J = 6.3 Hz, 1.8 Hz, 2H), 4.13-4.06 (m, 2H), 3.09 (t, J = 7.6 Hz, 4H), 2.10 (dd, J = 11.6 Hz, 3.0 Hz, 1H), 1.96-1.87 (m, 1H), 1.82-1.72 (m, 2H), 1.57-1.50 (m, 5H), 1.46-1.35 (m, 2H), 1.23 (t, J = 7.1 Hz, 3H), 0.87 (t, J = 7.4 Hz, 6H), 0.22 (s, 9H); **^{13}C NMR (100 MHz, $CDCl_3$)** δ: 175.46, 165.16, 143.99, 133.62, 130.08, 126.86, 65.45, 59.54, 49.81, 36.85, 28.34, 26.65, 26.63, 21.82, 14.46, 11.06, -2.97.

HRMS (ESI): $[M+H]^+$ calculated for $C_{24}H_{41}GeNO_6S$= 546.1944, found: 546.1939.

6-Ethoxy-6-oxo-5-(trimethylgermyl)hexyl (1S,4R)-4,7,7-trimethyl-3-oxo-2-oxabicyclo[2.2.1]heptane-1-carboxylate (63)

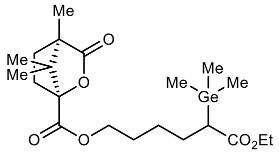

In an oven-dried 8 mL reaction vial equipped with a magnetic stir bar, ethyl acrylate (32.6 μL, 0.3 mmol), trimethylchlorogermane (37.6 μL, 0.3 mmol), $NiBr_2$ (4.4 mg, 0.02 mmol), Mn (33.0 mg, 0.6 mmol), DMA (2.0 mL) were charged under N_2 atmosphere at 35 °C for 0.5 h, then 3-bromopropyl (1S,4R)-4,7,7-trimethyl-3-oxo-2-oxabicyclo[2.2.1]heptane-1-carboxylate (63.6 mg, 0.2 mmol) were added under N_2, stirring at 35 °C for another 36 h.

The crude material was purified by flash chromatography (Petroleum ether: EtOAc = 9: 2 and DCM) to provide the title compound as a clear pale yellow oil (43.2 mg, 47% yield, d.r.=1:1.2).

^{1}H NMR (400 MHz, $CDCl_3$) δ: 4.25-4.17 (m, 2H), 4.12-4.06 (m, 2H), 2.45-2.38 (m, 1H), 2.09-1.81 (m, 4H), 1.72-1.64 (m, 3H), 1.46-1.28 (m, 3H), 1.23 (t, J = 7.2 Hz, 3H),

1.11 (s, 3H), 1.05 (s, 3H), 0.95 (s, 3H), 0.21 (s, 9H); **^{13}C NMR (100 MHz, CDCl$_3$)** δ: 178.17, 175.50, 167.49, 91.13, 65.48, 59.60, 54.73, 54.08, 35.86, 30.58, 28.91, 28.37, 26.59, 26.53, 26.50, 16.75, 16.71, 14.52, 9.68, 2.92.

HRMS (ESI): $[M+H]^+$ calculated for $C_{21}H_{36}GeO_6$= 459.1802, found: 459.1798.

HPLC analysis was performed on Agilent 1100 using Daicel Chiralpak AD Column. Chiral HPLC Analysis Conditions: a) Column CHIRALPAK®AD-H, 0.46 cmI.D.*25 cmL; b) Mobile phase: n-Hexane/EtOH= 95/5 (v/v); c) Flow rate: 1.0 mL/min; d) Abs, detector : 220 nm.

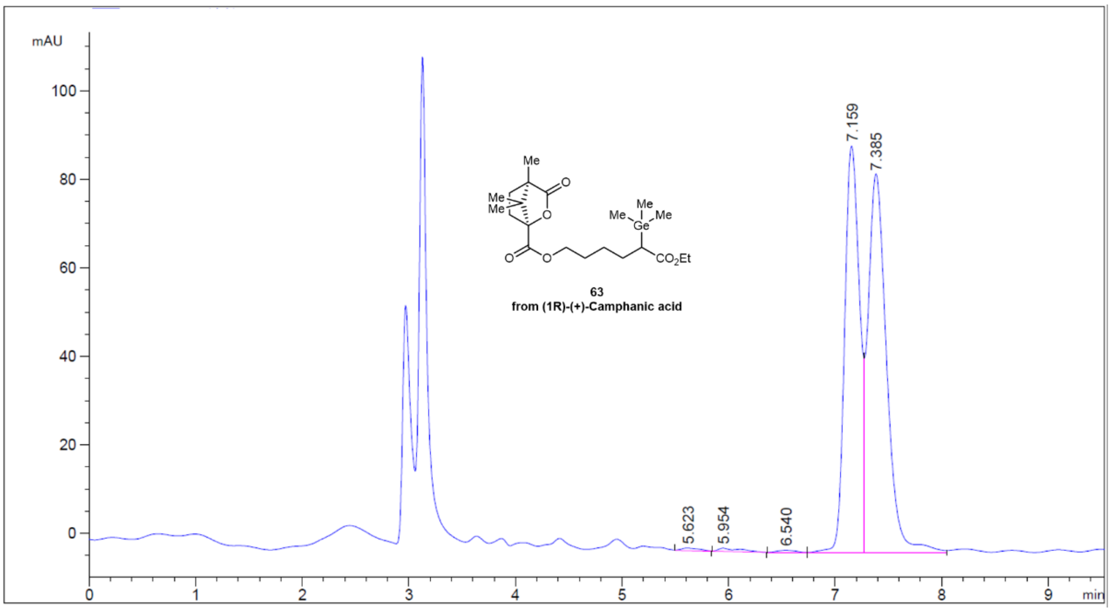

Peak#	RetTime[min]	Type	Width[min]	Area mAU*S	Area %
1	5.623	VB	0.2031	7.11379	0.3653
2	5.954	BB	0.1945	10.06648	0.5169
3	6.540	BB	0.1909	5.17639	0.2658
4	7.159	BV	0.1539	891.58026	45.7858
5	7.385	VV	0.1821	1033.34912	53.0661

Ethyl 6-((3-(4,5-diphenyloxazol-2-yl)propanoyl)oxy)-2-(trimethylgermyl)hexanoate (64)

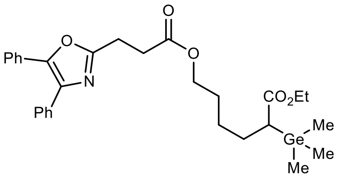

In an oven-dried 8 mL reaction vial equipped with a magnetic stir bar, ethyl acrylate (32.6 μL, 0.3 mmol), trimethylchlorogermane (37.6 μL, 0.3 mmol), $NiBr_2$ (4.4 mg, 0.02 mmol), Mn (33.0 mg, 0.6 mmol), DMA (2.0 mL) were charged under N_2 atmosphere at

35 ºC for 0.5 h, then 3-bromopropyl 3-(4,5-diphenyloxazol-2-yl)propanoate (82.6 mg, 0.2 mmol) were added under N_2, stirring at 35 ºC for another 36 h.

The crude material was purified by flash chromatography (Petroleum ether: EtOAc = 10: 1) to provide the title compound as a clear pale yellow oil (60.3 mg, 55% yield).

^{1}H NMR (400 MHz, CDCl$_3$) δ: 7.63 (d, J = 8.2 Hz, 2H), 7.57 (d, J = 8.4 Hz, 2H), 7.38-7.30 (m, 6H), 4.12-4.05 (m, 4H), 3.18 (t, J = 7.1 Hz, 2H), 2.90 (t, J = 7.2 Hz, 2H), 2.08-2.04 (m, 1H), 1.89-1.80 (m, 1H), 1.67-1.62 (m, 2H), 1.42-1.28 (m, 3H), 1.23 (t, J = 7.6 Hz, 3H), 0.20 (s, 9H); **^{13}C NMR (100 MHz, CDCl$_3$)** δ: 175.49, 171.98, 161.72, 145.30, 135.02, 132.37, 128.89, 128.56, 128.47, 128.36, 127.97, 127.81, 126.37, 64.65, 59.52, 36.81, 31.07, 28.31, 26.59, 26.49, 23.47, 14.48, -2.95.

HRMS (ESI): $[M+H]^+$ calculated for $C_{29}H_{37}GeNO_5$= 554.1962, found: 554.1957.

Ethyl 6-(5-chloro-2-(2,4-dichlorophenoxy)phenoxy)-2-(trimethylgermyl)hexanoate (65)

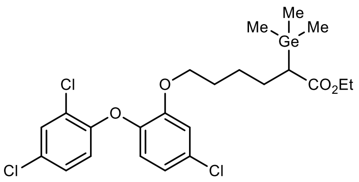

In an oven-dried 8 mL reaction vial equipped with a magnetic stir bar, ethyl acrylate (32.6 μL, 0.3 mmol), trimethylchlorogermane (37.6 μL, 0.3 mmol), $NiBr_2$ (4.4 mg, 0.02 mmol), Mn (33.0 mg, 0.6 mmol), DMA (2.0 mL) were charged under N_2 atmosphere at 35 ºC for 0.5 h, then 2-(3-bromopropoxy)-4-chloro-1-(2,4-dichlorophenoxy)benzene (81.6 mg, 0.2 mmol) were added under N_2, stirring at 35 ºC for another 36 h.

The crude material was purified by flash chromatography (Petroleum ether: EtOAc = 40: 1) to provide the title compound as a clear pale yellow oil (66.7 mg, 61% yield).

^{1}H NMR (400 MHz, CDCl$_3$) δ: 7.42 (d, J = 2.5 Hz, 1H), 7.08 (dd, J = 8.8 Hz, J = 2.5 Hz, 1H), 6.96-6.89 (m, 3H), 6.63 (d, J = 8.8 Hz, 1H), 4.11-4.05 (m, 2H), 3.94-3.84 (m, 2H), 2.01 (dd, J = 11.6 Hz, J = 2.9 Hz, 1H), 1.83-1.75 (m, 1H), 1.63-1.59 (m, 1H), 1.37-1.26 (m, 2H), 1.22 (t, J = 6.0 Hz, 3H), 0.91-0.77 (m, 2H), 0.20 (s, 9H); **^{13}C NMR (100 MHz, CDCl$_3$)** δ: 175.59, 152.53, 150.92, 142.93, 130.53, 130.03, 127.66, 127.51, 124.32, 122.06, 120.80, 117.84, 114.64, 68.80, 59.54, 36.81, 28.68, 26.61, 26.37, 14.51, 2.93.

HRMS (ESI): $[M+H]^+$ calculated for $C_{23}H_{29}C_{13}GeO_4$= 549.0421, found: 549.0416.

(1R,2S,5R)-2-isopropyl-5-methylcyclohexyl 3-(1-tosylpiperidin-4-yl)-2-(trimethylgermyl)propanoate (66)

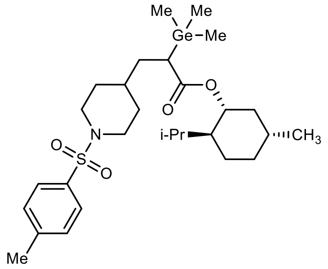

In an oven-dried 8 mL reaction vial equipped with a magnetic stir bar, (1R,2S,5R)-2-isopropyl-5-methylcyclohexyl acrylate (63.0 mg, 0.3 mmol), trimethylchlorogermane (37.6 μL, 0.3 mmol), $NiBr_2$ (4.4 mg, 0.02 mmol), Mn (33.0 mg, 0.6 mmol), DMA (2.0 mL) were charged under N_2 atmosphere at 35 ºC for 0.5 h, then 4-bromo-1-tosylpiperidine (63.4 mg, 0.2 mmol) were added under N_2, stirring at 35 ºC for another 36 h.

The crude material was purified by flash chromatography (Petroleum ether: EtOAc = 10: 1 and DCM: PE=1: 1) to provide the title compound as a clear pale yellow oil (74.8 mg, 66% yield), d.r.=1:1.4.

^{1}H NMR (400 MHz, $CDCl_3$) δ: 7.62 (d, J = 7.2 Hz, 2H), 7.31 (d, J = 7.6 Hz, 2H), 4.64-4.52 (m, 1H), 3.78-3.69 (m, 2H), 2.42 (s, 3H), 2.15-2.02 (m, 3H), 1.89-1.64 (m, 6H), 1.51-1.37 (m, 1H), 1.37-1.25 (m, 3H), 1.18-1.09 (m, 3H), 1.01-0.94 (m, 1H), 0.88-0.79 (m,8H), 0.61 (dd, J = 20.1 Hz, J = 6.7 Hz, 3H), 0.19 (m, 9H); **^{13}C NMR (100 MHz, $CDCl_3$)** δ: 174.93, 143.31, 132.72, 129.43, 127.62, 73.64, 73.24, 53.38, 46.97, 46.86, 46.44, 46.40, 41.23, 41.14, 35.34, 35.16, 34.34, 34.11, 33.57, 33.45, 32.98, 32.03, 31.92, 31.32, 30.18, 30.08, 25.98, 25.46, 22.99, 22.58, 21.96, 21.40, 20.84, 20.65, 15.86, 15.50, -3.04, -3.08.

HRMS (ESI): $[M+H]^+$ calculated for $C_{28}H_{47}GeNO_4S$= 568.2516, found: 568.2512.

2-(1-(4-chlorobenzoyl)-5-methoxy-2-methyl-1H-indol-3-yl)ethyl 3-(1-tosylpiperidin-4-yl)-2-(trimethylgermyl)propanoate (67)

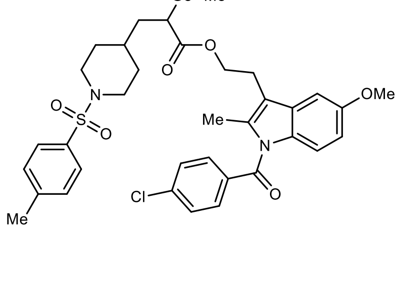

In an oven-dried 8 mL reaction vial equipped with a magnetic stir bar, 2-(1-(4-chlorobenzoyl)-5-methoxy-2-methyl-1H-indol-3-yl)ethyl acrylate (119.1 mg, 0.3 mmol), trimethylchlorogermane (37.6 μL, 0.3 mmol), $NiBr_2$ (4.4 mg, 0.02 mmol), Mn (33.0 mg, 0.6 mmol), DMA (2.0 mL) were charged under N_2 atmosphere at 35 ºC for 0.5 h, then 4-bromo-1-tosylpiperidine (63.4 mg, 0.2 mmol) were added under N_2, stirring at 35 ºC for another 36 h.

The crude material was purified by flash chromatography (Petroleum ether: EtOAc = 10: 1) to provide the title compound as a clear pale yellow oil (70.1 mg, 46% yield).

^{1}H NMR (400 MHz, $CDCl_3$) δ: 7.64-7.59 (m, 4H), 7.47 (d, *J* = 8.4 Hz, 2H), 7.31-7.29 (m, 2H), 6.95 (s, 1H), 6.82 (d, *J* = 9.0 Hz, 1H), 6.62 (d, *J* = 9.0 Hz, 1H), 4.20-4.09 (m, 2H), 3.82 (s, 3H), 3.71 (t, *J* = 11.6 Hz, 2H), 2.94 (t, *J* = 7.4 Hz, 2H), 2.42 (s, 3H), 2.35 (s, 3H), 2.15-2.04 (m, 3H), 1.88-1.82 (m, 1H), 1.28-1.25 (m, 3H), 1.14-1.09 (m, 3H), 0.16 (s, 9H); **^{13}C NMR (100 MHz, $CDCl_3$)** δ: 175.44, 168.20, 155.88, 143.34, 139.19, 135.21, 133.92, 132.94, 131.09, 130.85, 129.53, 129.10, 127.69, 115.36, 114.92, 111.11, 101.31, 62.85, 55.68, 46.33, 35.13, 33.81, 33.11, 31.85, 30.36, 23.81, 21.48, 13.27, -3.01.

HRMS (ESI): $[M+H]^+$ calculated for $C_{37}H_{45}ClGeN_2O_6S$= 755.1977, found: 755.1972.

6-(((1R,2S,5R)-2-isopropyl-5-methylcyclohexyl)oxy)-6-oxo-5-(trimethylgermyl)hexyl 4-(N,N-dipropylsulfamoyl)benzoate (68)

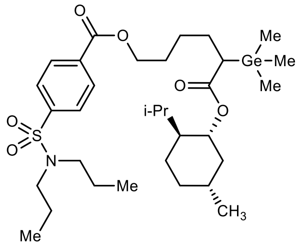

In an oven-dried 8 mL reaction vial equipped with a magnetic stir bar, (1R,2S,5R)-2-isopropyl-5-methylcyclohexyl acrylate (63.0 mg, 0.3 mmol), trimethylchlorogermane (37.6 μL, 0.3 mmol), $NiBr_2$ (4.4 mg, 0.02 mmol), Mn (33.0 mg, 0.6 mmol), DMA (2.0 mL) were charged under N_2 atmosphere at 35 ºC for 0.5 h, then 3-bromopropyl 4-(N,N-dipropylsulfamoyl)benzoate (81.0 mg, 0.2 mmol) were added under N_2, stirring at 35 ºC for another 36 h.

The crude material was purified by flash chromatography (Petroleum ether: EtOAc = 15: 1 and DCM: PE=1: 1) to provide the title compound as a clear pale yellow oil (60.2 mg, 46% yield, d.r.=1:1.4).

^{1}H NMR (400 MHz, $CDCl_3$) δ: 8.14 (d, *J* = 8.6 Hz, 2H), 7.86 (d, *J* = 8.6 Hz, 2H), 4.73-4.59 (m, 1H), 4.34-4.28 (m, 2H), 3.09 (t, *J* = 7.7 Hz, 4H), 2.09-2.04 (m, 1H), 2.01-1.88

(m, 3H), 1.84-1.75 (m, 2H), 1.71-1.63 (m, 3H), 1.55-1.50 (m, 5H), 1.46-1.28 (m, 5H), 0.89-0.82 (m, 12H), 0.75-0.71 (m, 4H), 0.22 (s, 9H); **^{13}C NMR (100 MHz, $CDCl_3$)** δ: 175.03, 165.20, 144.04, 133.63, 130.12, 126.90, 73.74, 73.30, 65.53, 49.85, 47.06, 46.95, 41.31, 41.22, 37.60, 36.81, 34.18, 31.36, 31.31, 28.37, 28.29, 27.31, 26.87, 26.72, 26.60, 26.16, 25.57, 23.20, 22.69, 22.02, 21.85, 20.91, 20.69, 16.10, 15.63, 11.08, -2.89. HRMS (ESI): $[M+H]^+$ calculated for $C_{32}H_{55}GeNO_6S$= 656.3040, found: 656.3035.
HPLC analysis was performed on Agilent HP1100 using Daicel Chiralpak AD Column. Chiral HPLC Analysis Conditions: a) Column CHIRALPAK®AD-H, 0.46 cmI.D.*25 cmL; b) Mobile phase: n-Hexane/EtOH= 90/10 (v/v); c) Flow rate: 1.0 mL/min; d) Abs, detector : 254 nm.

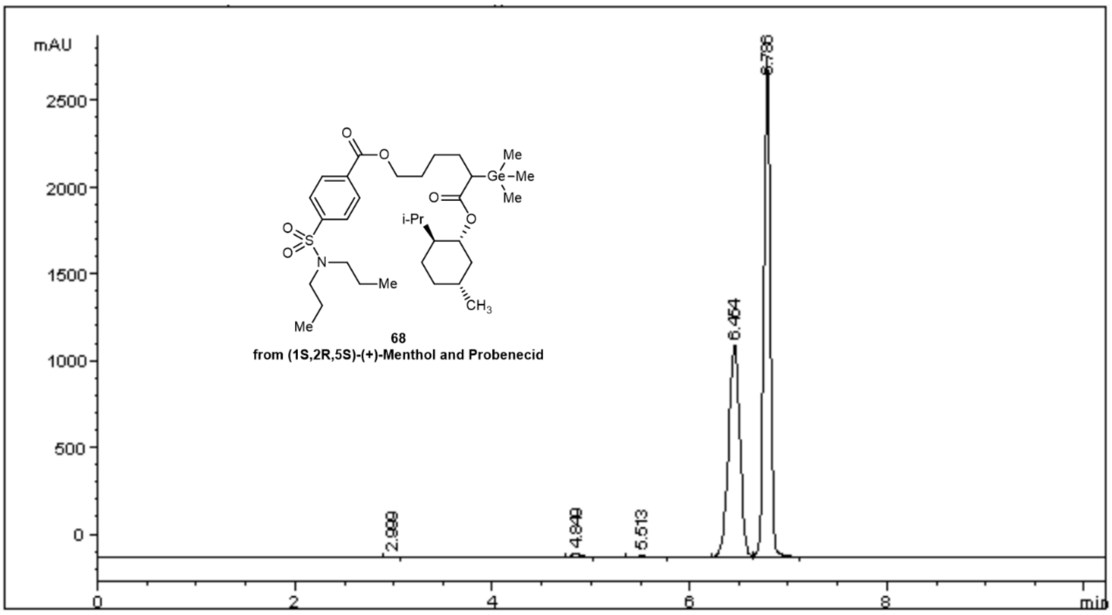

Peak #	RetTime [min]	Type	Width [min]	Area mAU *s	Height [mAU]	Area %
1	2.999	PV	0.0855	10.97230	2.01124	0.0468
2	4.849	BB	0.0918	104.36163	17.76285	0.4453
3	5.513	BB	0.1500	34.48658	3.49812	0.1471
4	6.454	BV	0.1264	9861.90430	1219.43945	42.0784
5	6.786	VB	0.0734	1.34253e4	2874.53784	57.2824

3.3. Gram-scale Reactions and Synthetic Applications

3.3.1 Gram-scale reaction for 4

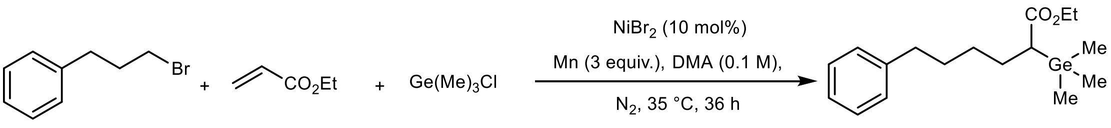

1a 5 mmol, 1g | **2a** (1.5 equiv) | **3a** (1.5 equiv) | **4** 1.33g, 79%

In an oven-dried 100 mL round-bottom flask equipped with a magnetic stir bar, ethyl acrylate (815 μL, 7.5 mmol), trimethylchlorogermane (927 μL, 7.5 mmol), $NiBr_2$ (110 mg, 0.5 mmol), Mn (825 mg, 15 mmol), DMA (50 mL) were charged under N_2 atmosphere at 35 ºC for 0.5 h, then (3-bromopropyl)benzene (5.0 mmol, 1 g) were added under N_2, stirring at 35 ºC for another 36 h.
The crude material was purified by flash chromatography (Petroleum ether: EtOAc = 40: 1) to provide the title compound as a clear colorless oil (1.33 g, 79% yield).

3.3.2 Synthetic transformations of 4

Procedure for synthesis of 69

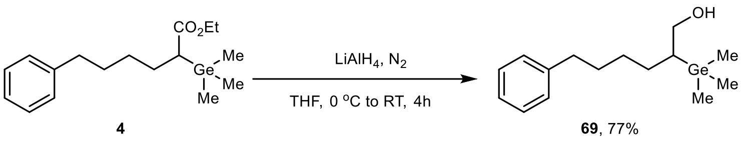

To a solution of ethyl 6-phenyl-2-(trimethylgermyl)hexanoate (67.6 mg, 0.2 mmol) in THF (2 mL), $LiAlH_4$ (2.5 mmol/L, 40 μL) was dropwise added at 0 ºC under N_2. The mixture was moved to the room temperature for another 4 h stirring, quenched by NH_4Cl aq solution and extracted by EA. The crude material was purified by flash chromatography (Petroleum ether: EtOAc = 15: 1) to provide the compound as a clear pale yellow oil (46.0 mg, 77% yield)
^{1}H NMR (400 MHz, $CDCl_3$) δ: 7.37-7.33 (m, 2H), 7.26-7.23 (m, 3H), 3.88-3.84 (m, 1H), 3.79-3.74 (m, 1H), 2.69 (t, J = 7.8 Hz, 2H), 1.74-1.57 (m, 4H), 1.51-1.34 (m, 3H), 1.24-1.18 (m, 1H), 0.21 (s, 9H). **^{13}C NMR (100 MHz, $CDCl_3$)** δ: 142.66, 128.35, 128.21, 125.58, 64.60, 35.83, 32.02, 31.74, 28.71, 28.09, -2.58.
HRMS (ESI): $[M+Na]^+$ calculated for $C_{15}H_{26}GeO$= 319.1087, found: 319.1081.

Procedure for synthesis of 70

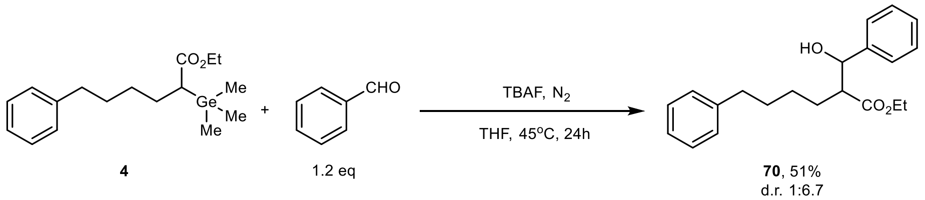

To a solution of ethyl 6-phenyl-2-(trimethylgermyl)hexanoate (67.6 mg, 0.2 mmol) and benzaldehyde (25 μL, 0.24 mmol) in THF (0.4 mL), TBAF (1 mmol/mL, 400 μL) was dropwise added at room temperature under N_2, and sitrred at 45 ºC for another 24 h. The crude material was purified by flash chromatography (Petroleum ether: EtOAc = 10: 1) to provide the title compound as a clear colorless oil (33.3 mg, 51% yield,

d.r.=1:6.7).

^{1}H NMR (400 MHz, CDCl$_3$) δ: 7.34-7.10 (m, 10H), 4.93 (d, J = 5.6 Hz, 1H), 4.03 (q, J = 7.1 Hz, 2H), 2.84 (s, 1H), 2.71-2.66 (m, 1H), 2.53 (td, J = 2.8 Hz, J = 7.7 Hz, 2H), 1.82-1.72 (m, 1H), 1.68-1.60 (m, 2H), 156-1.49 (m, 1H), 1.38-1.28 (m, 1H), 1.24-1.19 (m, 1H), 1.10 (t, J = 7.1 Hz, 3H); **^{13}C NMR (100 MHz, CDCl$_3$)** δ: 175.05, 142.40, 141.55, 128.34, 128.25, 128.19, 127.66, 126.16, 125.60, 74.19, 60.49, 52.36, 35.56, 31.14, 27.05, 26.72, 14.04.

HRMS (ESI): $[M+Na]^+$ calculated for $C_{21}H_{26}O_3$= 349.1774, found: 349.1774.

Procedure for synthesis of 71

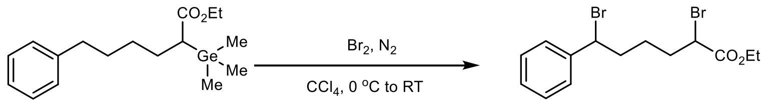

4

71, 56%
d.r.=1:1.1

To a solution of ethyl 6-phenyl-2-(trimethylgermyl)hexanoate (67.6 mg, 0.2 mmol) in CCl_4 (10 mL), Br_2 (0.2 mmol in 20 mL CCl_4) was dropwise added at 0 ºC under N_2. The mixture was stirred for 1.5 h at 0 ºC and another 2 h at room temperature. The crude material was purified by flash chromatography (Petroleum ether: EtOAc = 40: 1) to provide the title compound as a clear colorless oil (42.6 mg, 56% yield, d.r.=1:1.1).

^{1}H NMR (400 MHz, CDCl$_3$) δ: 7.39-7.26 (m, 5H), 4.95-4.91 (m, 1H), 4.20-4.15 (m, 3H), 2.37-2.25 (m, 1H), 2.20-1.97 (m, 3H), 1.76-1.59 (m, 1H), 1.52-1.34 (m, 1H), 1.28 (td, J = 1.6 Hz, J = 7.1 Hz, 3H); **^{13}C NMR (100 MHz, CDCl$_3$)** δ: 169.54, 141.71, 128.73, 128.45, 127.15, 61.39, 54.64, 54.60, 45.44, 45.40, 39.06, 34.02, 34.01, 25.99, 13.91.

HRMS (ESI): $[M+Na]^+$ calculated for $C_{15}H_{21}BrO_3$= 351.0566, found: 351.0563 (The compound **71** was solvolyzed during the mesurement using $MeOH/H_2O$ as the eluent).

Procedure for synthesis of 72

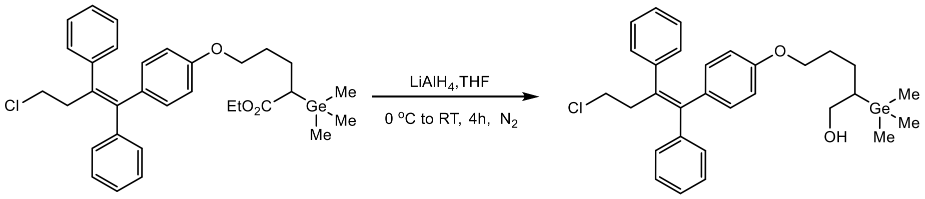

61

72

To a solution of **61** (116.0 mg, 0.2 mmol) in THF (2 mL), $LiAlH_4$ (2.5 mmol/L, 40 μL) was dropwise added at 0 ºC under N_2. The mixture was moved to the room temperature for another 4 h stirring, quenched by aq. NH_4Cl solution and extracted by DCM. The crude material was purified by flash chromatography (Petroleum ether: EtOAc = 5: 1)

to provide the compound as a clear pale yellow oil (82.9 mg, 77% yield). **^{1}H NMR (400 MHz, CDCl$_3$)** δ: 7.39-7.36 (m, 2H), 7.30-7.26 (m, 3H), 7.22-7.14 (m, 5H), 6.78 (d, *J* = 8.7 Hz, 2H), 6.55 (d, *J* = 8.7 Hz, 2H), 3.84-3.79 (m, 3H), 3.73-3.68 (m, 1H), 3.42 (t, *J* = 7.5 Hz, 2H), 2.93 (t, *J* = 7.5 Hz, 2H), 1.80-1.72 (m, 2H), 1.66-1.60 (m, 2H), 1.35 (br, 1H), 1.19-1.13 (m, 1H), 0.14 (s, 9H); **^{13}C NMR (100 MHz, CDCl$_3$)** δ: 157.19, 142.91, 141.65, 140.99, 135.02, 134.61, 131.67, 129.52, 129.37, 128.30, 128.19, 126.90, 126.53, 113.37, 67.76, 64.45, 42.86, 38.58, 31.64, 28.65, 24.71, 2.63. HRMS (ESI): $[M+Na]^+$ calculated for $C_{30}H_{37}ClGeO_2$= 561.1586, found: 561.1573.

Procedure for synthesis of 73

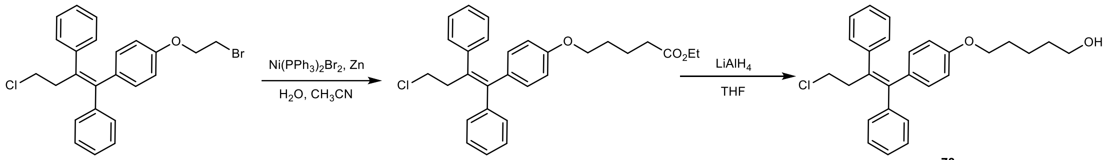

In an oven-dried 8 mL reaction vial equipped with a magnetic stir bar, 1**bq** (440.0 mg, 1.0 mmol), ethyl acrylate (435 μL, 4.0 mmol), $NiCl_2(PPh_3)_2$ (65.4 mg, 0.1 mmol), Zn (163.5 mg, 2.5 mmol), H_2O (18 μL, 1mmol), CH_3CN (2.5 mL) were charged under N_2 atmosphere at 80 ºC for 12 h. The crude material was purified by flash chromatography (Petroleum ether: EtOAc = 40: 1) to provide the title compound as a clear pale yellow oil (286.5 mg, 62% yield).

^{1}H NMR (400 MHz, CDCl$_3$) δ: 7.40-7.30 (m, 5H), 7.21-7.15 (m, 5H), 6.80 (d, *J* = 8.7 Hz), 6.55 (d, *J* = 8.8 Hz), 4.12 (q, *J* = 7.2 Hz, 2H), 8.84 (t, *J* = 5.2 Hz, 2H), 3.43 (t, *J* = 7.5 Hz, 2H), 2.94 (t, *J* = 7.4 Hz, 2H), 2.35 (t, *J* = 6.6 Hz, 2H), 1.76 (t, *J* = 3.8 Hz, 4H), 1.24 (t, *J* = 7.2 Hz, 3H). **^{1}H NMR (400 MHz, CDCl$_3$)** δ: 7.40-7.30 (m, 5H), 7.21-7.15 (m, 5H), 6.80 (d, *J* = 8.7 Hz), 6.55 (d, *J* = 8.8 Hz), 4.12 (q, *J* = 7.2 Hz, 2H), 8.84 (t, *J* = 5.2 Hz, 2H), 3.43 (t, *J* = 7.5 Hz, 2H), 2.94 (t, *J* = 7.4 Hz, 2H), 2.35 (t, *J* = 6.6 Hz, 2H), 1.76 (t, *J* = 3.8 Hz, 4H), 1.24 (t, *J* = 7.2 Hz, 3H). HRMS (ESI): $[M+Na]^+$ calculated for $C_{29}H_{31}ClO_3$= 485.1854, found: 485.1850.

To a solution of previous product (92.4 mg, 0.2 mmol) in THF (2 mL), $LiAlH_4$ (2.5 mmol/L, 40 μL) was dropwise added at 0 ºC under N_2. The mixture was moved to the room temperature for another 4 h stirring, quenched by aq. NH_4Cl solution and extracted by DCM. The crude material was purified by flash chromatography (Petroleum ether: EtOAc = 5: 1) to provide the compound as a clear pale yellow oil (67.2 mg, 80% yield).

^{1}H NMR (600 MHz, CDCl$_3$) δ: 7.39-7.29 (m, 5H), 7.22-7.14 (m, 5H), 6.79 (d, *J* = 8.7

Hz, 2H), 6.55 (d, *J* = 8.7 Hz, 2H), 3.83 (t, *J* = 6.4 Hz, 2H), 3.63 (t, *J* = 6.5 Hz, 2H), 3.43 (t, *J* = 7.5 Hz, 2H), 2.94 (t, *J* = 7.5 Hz, 2H), 1.76-1.71 (m,, 2H), 1.62-1.57 (m, 3H), 1.51-1.45 (m, 2H). **^{13}C NMR (100 MHz, $CDCl_3$)** δ: 157.19, 142.87, 141.72, 140.96, 135.00, 134.57, 131.62, 129.48, 129.32, 128.27, 128.15, 126.86, 126.49, 113.32, 67.44, 62.64, 42.81, 38.54, 32.29, 28.92, 22.24. HRMS (ESI): $[M+Na]^+$ calculated for $C_{27}H_{29}ClO_2$= 443.1748, found: 443.1736.

3.3.3 *In vitro* experiments

Cell Culture and Cytotoxicity Assay

MCF-7 and MDA-MB-231 cells were cultured in DMEM containing 10% FBS and 1% antibiotics (penicillin-streptomycin, 10000 U/mL) at 37°C in 5% CO_2. MCF-7, MDA-MB-231, MCF-10A cells were seeded in a 96-well plate, treated with a medium containing various concentration drugs and incubated for 96 h. Then per well was added 20 μL MTT (5 mg/mL) and incubated for another 4 h. And the cells were tested by classical MTT assay to assess the cell viability.

Hemolysis Assay

The erythrocyte was collected by washing blood with PBS, then treated with deionized, saline and different concentration of drug for 6 h at room temperature. After centrifugation (1000 g), the absorbance of the supernatants was recorded at 541 nm. Hemolysis equation: Hemolysis = $(A_{sample}-A_{negative})/(A_{positive} -A_{negative})$.

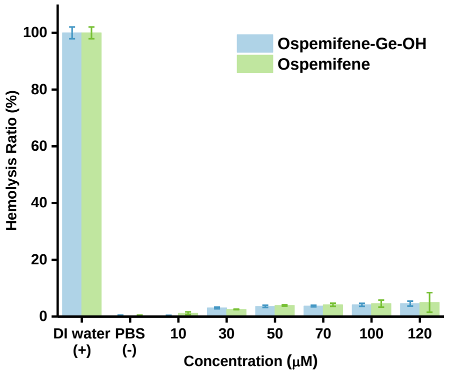

Supplementary Fig. 1. Hemolysis experiment against red blood cells of **72** and ospemifene. Data are presented as mean values ± SD (n = 3 biologically independent samples).

***In vitro* mouse microsomal stability assay**

The tests were measured by Shanghai Medicilon Inc.

Preheat 100mM K-buffer with 5mM $MgCl_2$ pH 7.41.

Piking solutions of test and reference compounds.

500 μM spiking solution (Add 5 μL of 10 mM stock solution of compound and reference into 95 μL of CH_3CN).

1.5 μM spiking solution in microsomes (0.75 mg/mL): add 1.5 μL of 500 μM spiking solution and 18.75 μL of 20 mg/mL liver microsomes into 479.75μL of K/Mg-Buffer.

Nicotinamide adenine dinucleotide phosphate (NADPH) stock solution (6 mM, 5 mg/mL) is prepared by dissolving NADPH into K/Mg-buffer.

Dispense 30 μL of 1.5 μM spiking solution containing 0.75 mg/mL microsomes solution to the assay plates designated for different time points (0, 15, 30, 45, 45 min) and pre-incubate other plate at 37 °C for 5 minutes. For 0-min, add 150 μL of CH_3CN containing internal standard to the wells before adding 15 μL of NADPH stock solution (6 mM). For other time points, adding 15 μL of NADPH stock solution (6 mM) to the wells to start the reaction and timing. At 5-min, 15-min, 30-min, 45-min, adding 150 μL of CH_3CN containing internal standard to the wells of corresponding plates respectively to stop the reaction. After quenching, shake the plates for 10 min (200 g) and then centrifuge at 2000 g for 15 min. Transfer 80 μL of the supernatant from each well into a 96-well sample plate containing 140 μL of pure water for LC/MS analysis (Shimadzu Nexera LC-40 & AB SCIEX TQ-6500+, positive ion ESI, internal standard for tolbutamide, column: ACQUITY UPLC HSS T3 (1.8 μm, 2.10×50 mm)).

3.4 Mechanistic Studies

3.4.1 Radical-trapping experiment

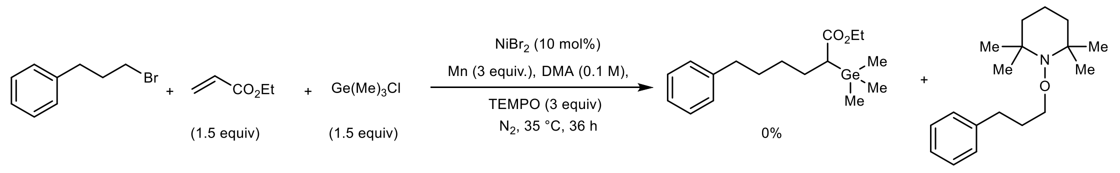

74
Detected by HRMS
M+H+=276.2337

In an oven-dried 8 mL reaction vial equipped with a magnetic stir bar, ethyl acrylate (48.9 μL, 0.45 mmol), trimethylchlorogermane (56.4 μL, 0.45 mmol), $NiBr_2$ (6.6 mg, 0.03 mmol), Mn (49.5 mg, 0.9 mmol), DMA (3.0 mL) were charged under N_2 atmosphere at 35 ºC for 0.5 h, then (3-bromopropyl)benzene (0.3 mmol, 59.7 mg) and TEMPO (141 mg, 0.9 mmol)were added under N_2, stirring at 35 ºC for another 36 h. The combined organic layers were washed with water, brine, dried over anhydrous Na_2SO_4, and concentrated under reduced pressure. In this reaction system, no target

product was detected by ^{1}H NMR analysis with dibromomethane (21.0 µL, 0.3 mmol) as an internal standard. The TEMPO-trapped product was detected by HRMS.

The HRMS spectrum of crude reaction mixture with the TEMPO additive:

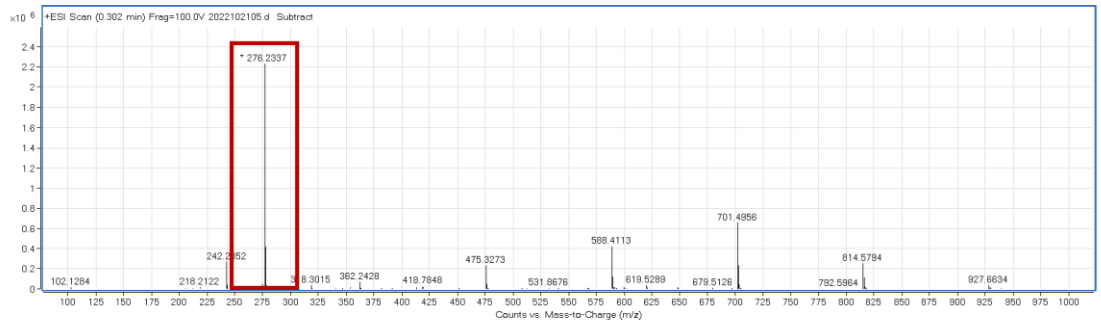

3.4.2 Radical-clock experiment

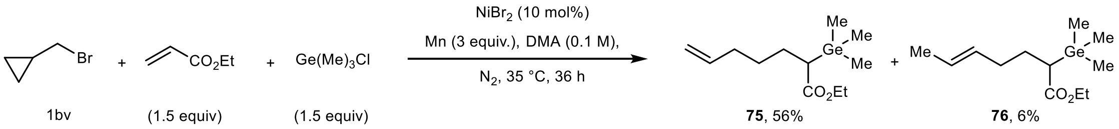

In an oven-dried 8 mL reaction vial equipped with a magnetic stir bar, ethyl acrylate (48.9 µL, 0.45 mmol), trimethylchlorogermane (56.4 µL, 0.45 mmol), $NiBr_2$ (6.6 mg, 0.03 mmol), Mn (49.5 mg, 0.9 mmol), DMA (3.0 mL) were charged under N_2 atmosphere at 35 ºC for 0.5 h, then (bromomethyl)cyclopropane (0.3 mmol, 40.5 mg) were added under N_2, stirring at 35 ºC for another 36 h.

The crude material was purified by flash chromatography (Petroleum ether: EtOAc = 100: 1) to provide the title compound as a clear yellow oil (**75**, 56% yield and **76**, 6% yield).

3.4.2 Radical-block experiment

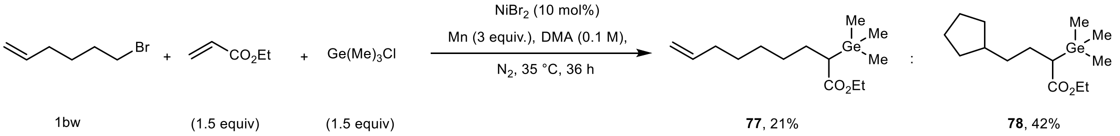

In an oven-dried 8 mL reaction vial equipped with a magnetic stir bar, ethyl acrylate (48.9 µL, 0.45 mmol), trimethylchlorogermane (56.4 µL, 0.45 mmol), $NiBr_2$ (6.6 mg, 0.03 mmol), Mn (49.5 mg, 0.9 mmol), DMA (3.0 mL) were charged under N_2 atmosphere at 35 ºC for 0.5 h, then 5-hexenyl bromide (0.3 mmol, 48.9 mg) were added under N_2, stirring at 35 ºC for another 36 h.

The crude material was purified by flash chromatography (Petroleum ether: EtOAc = 80: 1) to provide the title compound as a clear yellow oil (**77**, 21% yield and **78**, 42%

yield).

3.4.3 Control experiments

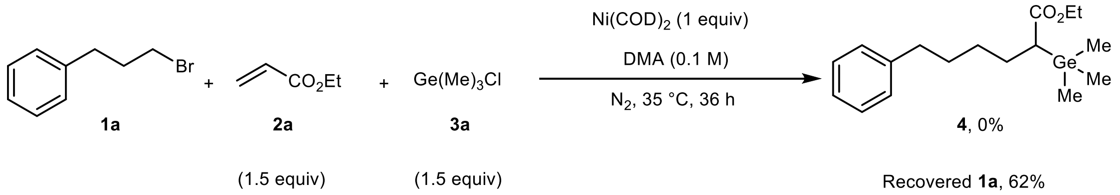

In an oven-dried 8 mL reaction vial equipped with a magnetic stir bar, ethyl acrylate (16.3 μL, 0.15 mmol), trimethylchlorogermane (18.8 μL, 0.15 mmol), Ni(COD)$_2$ (27.0 mg, 0.1 mmol), DMA (1.0 mL) were charged under N_2 atmosphere at 35 ºC for 0.5 h, then (3-bromopropyl)benzene (19.9 mg, 0.1 mmol) were added under N_2, stirring at 35 ºC for another 36 h.

The yields of **4** and recovered **1a** was determined by ^{1}H NMR analysis with dibromomethane (7.0 μL, 0.1 mmol) as an internal standard.

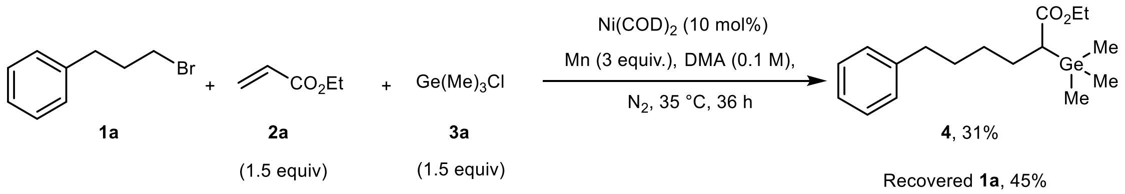

In an oven-dried 8 mL reaction vial equipped with a magnetic stir bar, ethyl acrylate (16.3 μL, 0.15 mmol), trimethylchlorogermane (18.8 μL, 0.15 mmol), Ni(COD)$_2$ (2.7 mg, 0.01 mmol), Mn (16.5 mg, 0.3 mmol), DMA (1.0 mL) were charged under N_2 atmosphere at 35 ºC for 0.5 h, then (3-bromopropyl)benzene (19.9 mg, 0.1 mmol) were added under N_2, stirring at 35 ºC for another 36 h.

The yields of **4** and recovered **1a** were determined by GC analysis with dodecane as an internal standard.

3.5 Copies of NMR Spectra

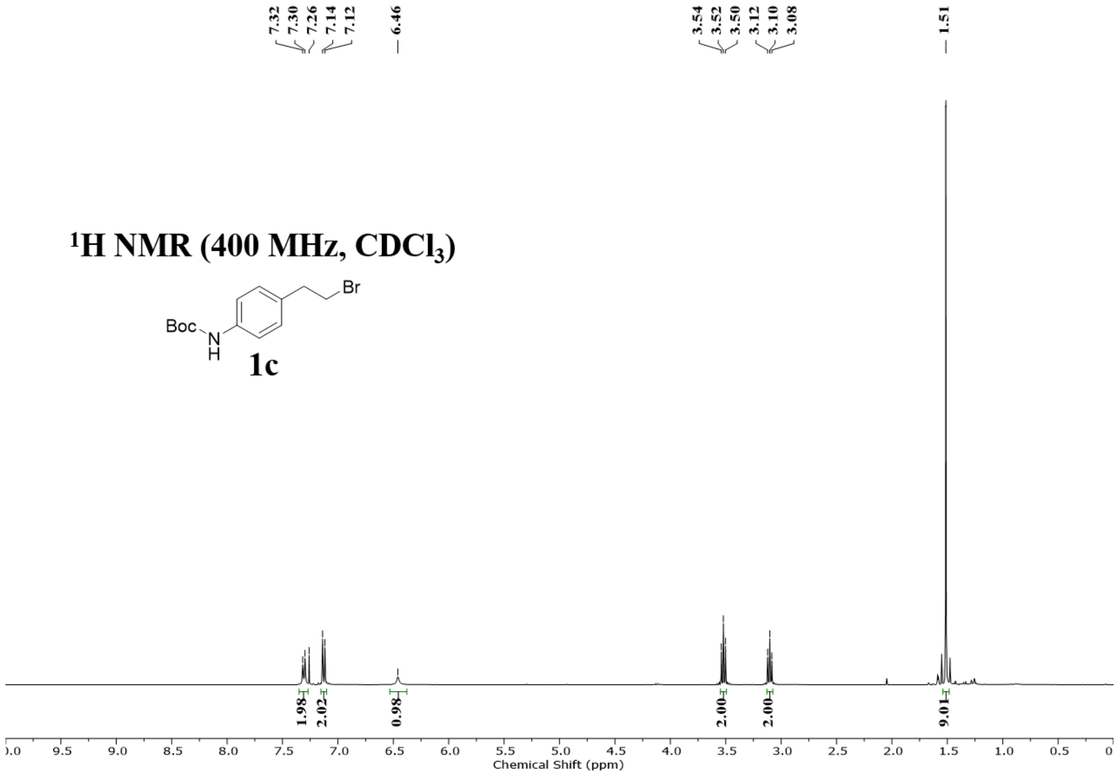

Supplementary Fig. 2. ¹H NMR of compound 1c. The sample has been recorded in 400 MHz, $CDCl_3$ at 25 ºC

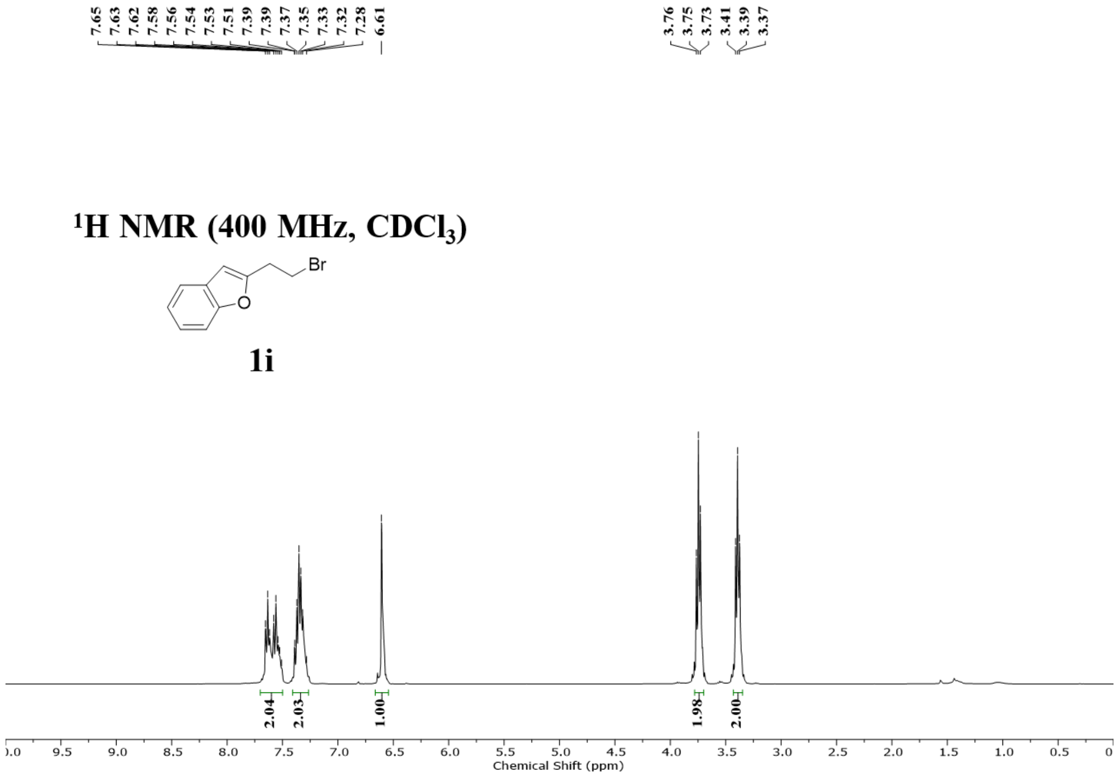

Supplementary Fig. 3. ¹H NMR of compound 1i. The sample has been recorded in 400 MHz, $CDCl_3$ at 25 ºC

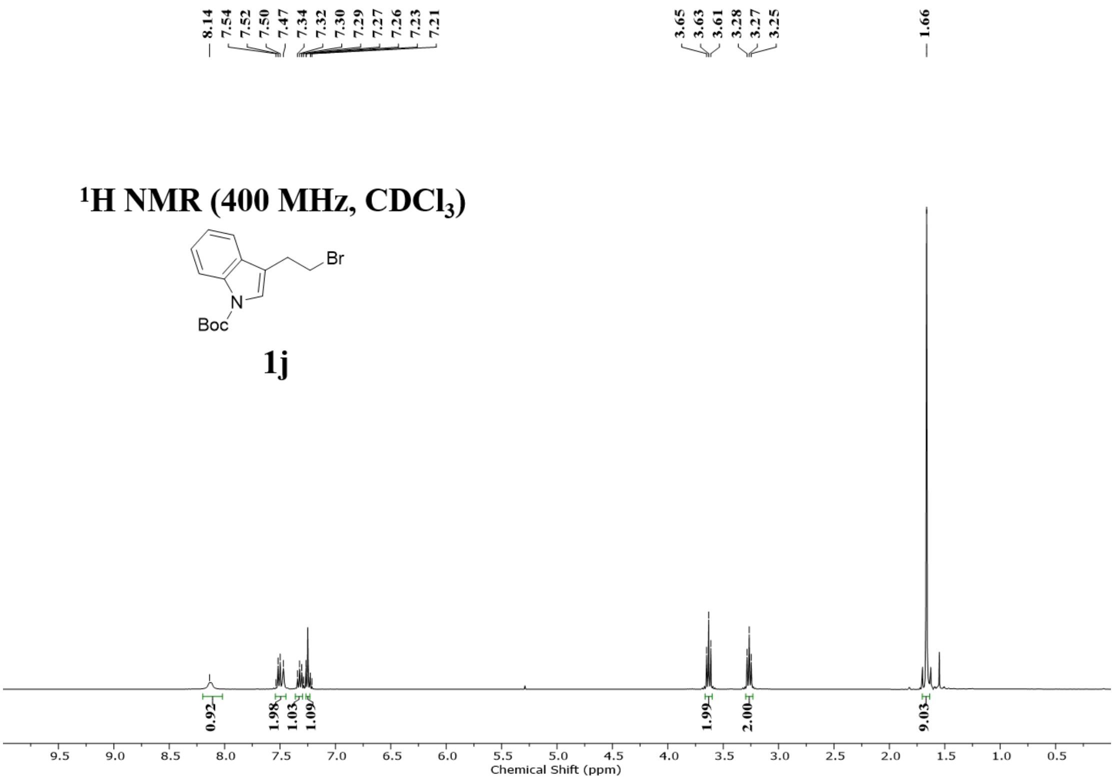

Supplementary Fig. 4. ¹H NMR of compound 1j. The sample has been recorded in 400 MHz, $CDCl_3$ at 25 ºC

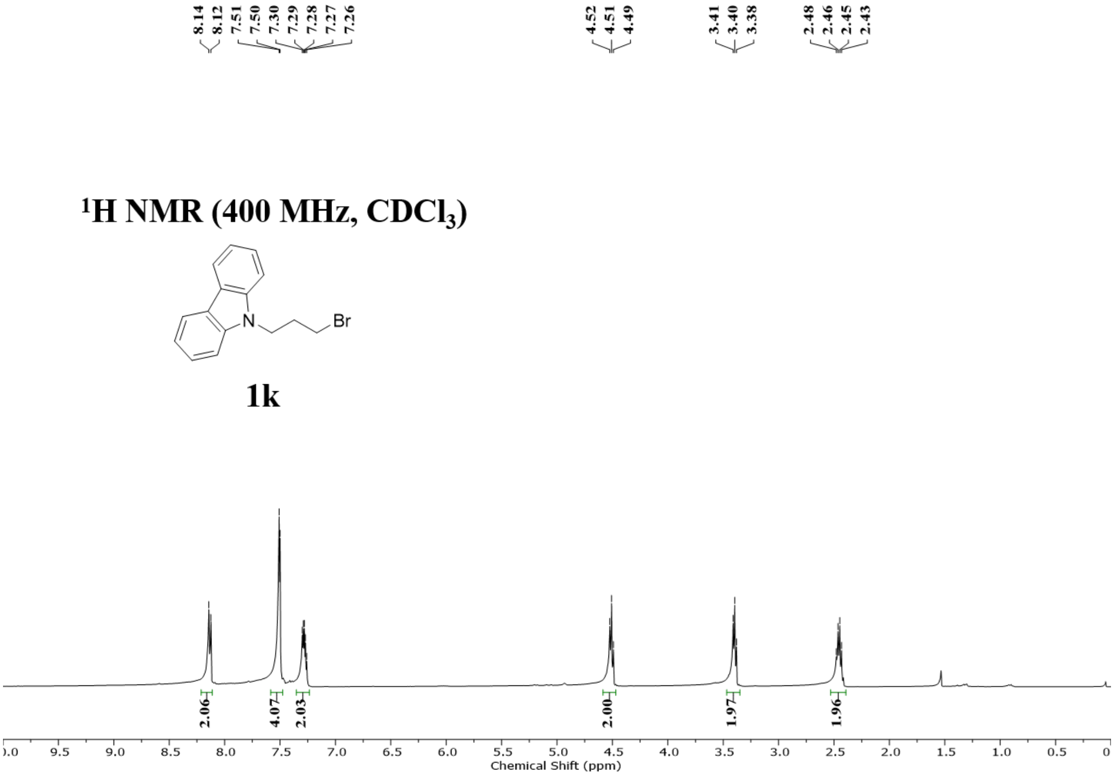

Supplementary Fig. 5. ¹H NMR of compound 1k. The sample has been recorded in 400 MHz, $CDCl_3$ at 25 ºC

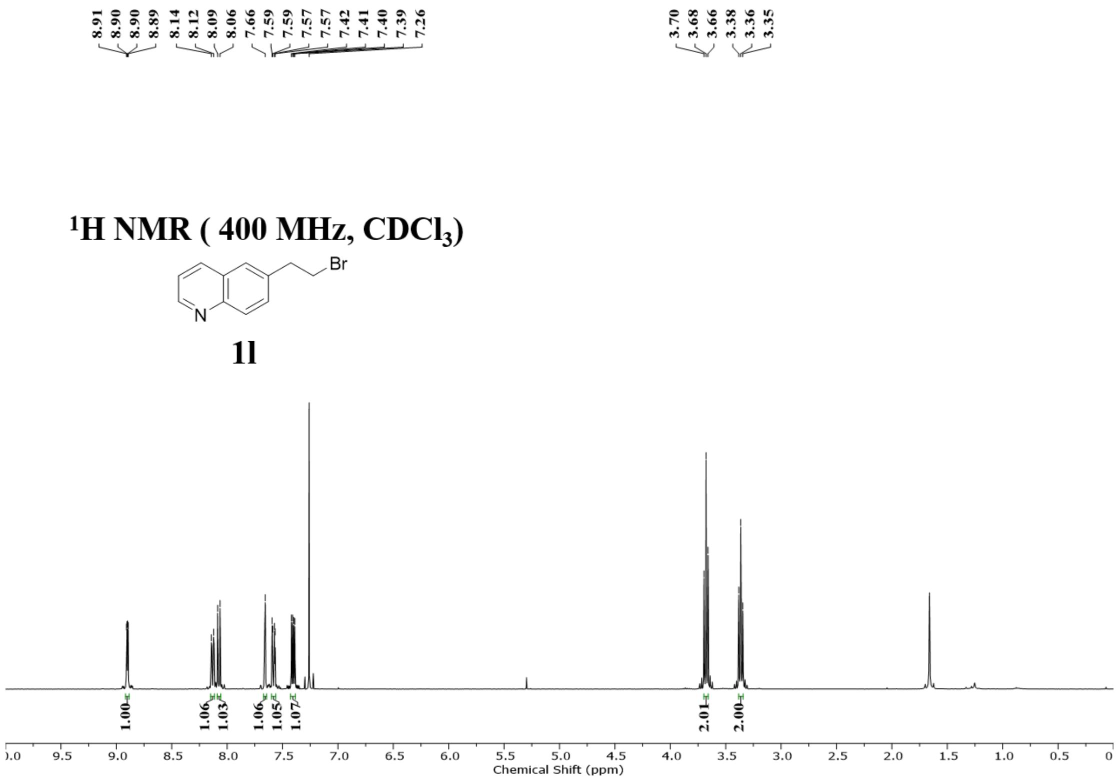

Supplementary Fig. 6. ^{1}H NMR of compound 1l. The sample has been recorded in 400 MHz, $CDCl_3$ at 25 ºC

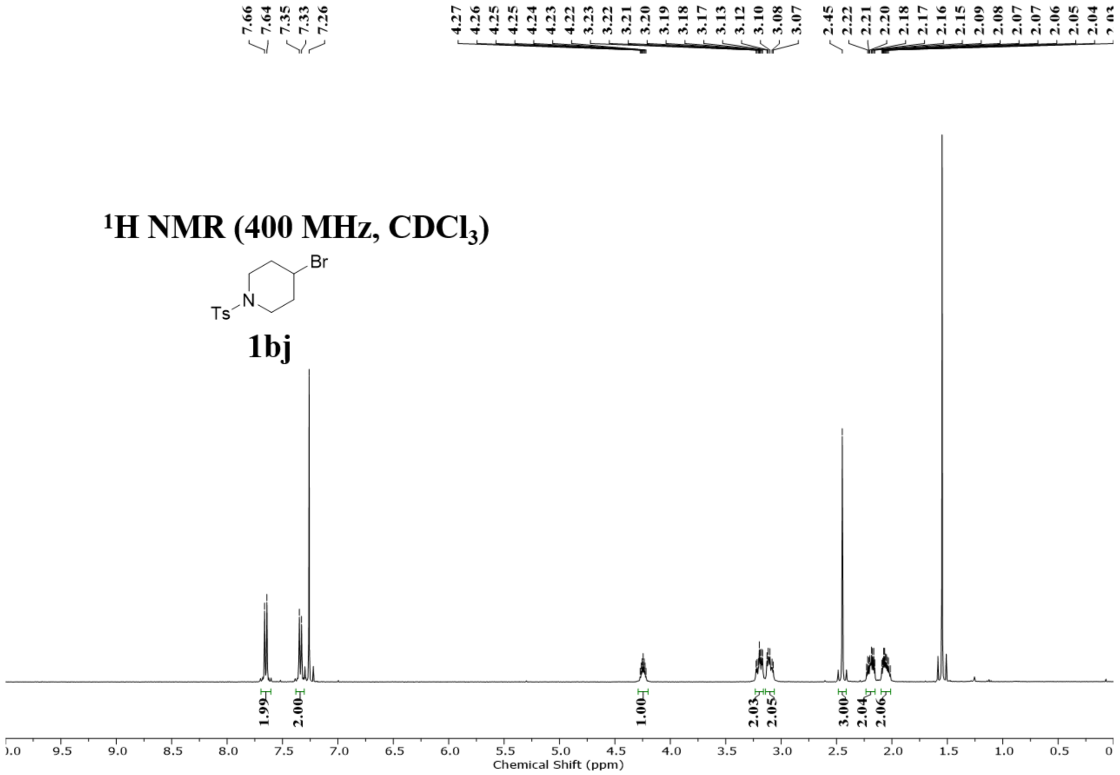

Supplementary Fig. 7. ^{1}H NMR of compound 1bj. The sample has been recorded in 400 MHz, $CDCl_3$ at 25 ºC

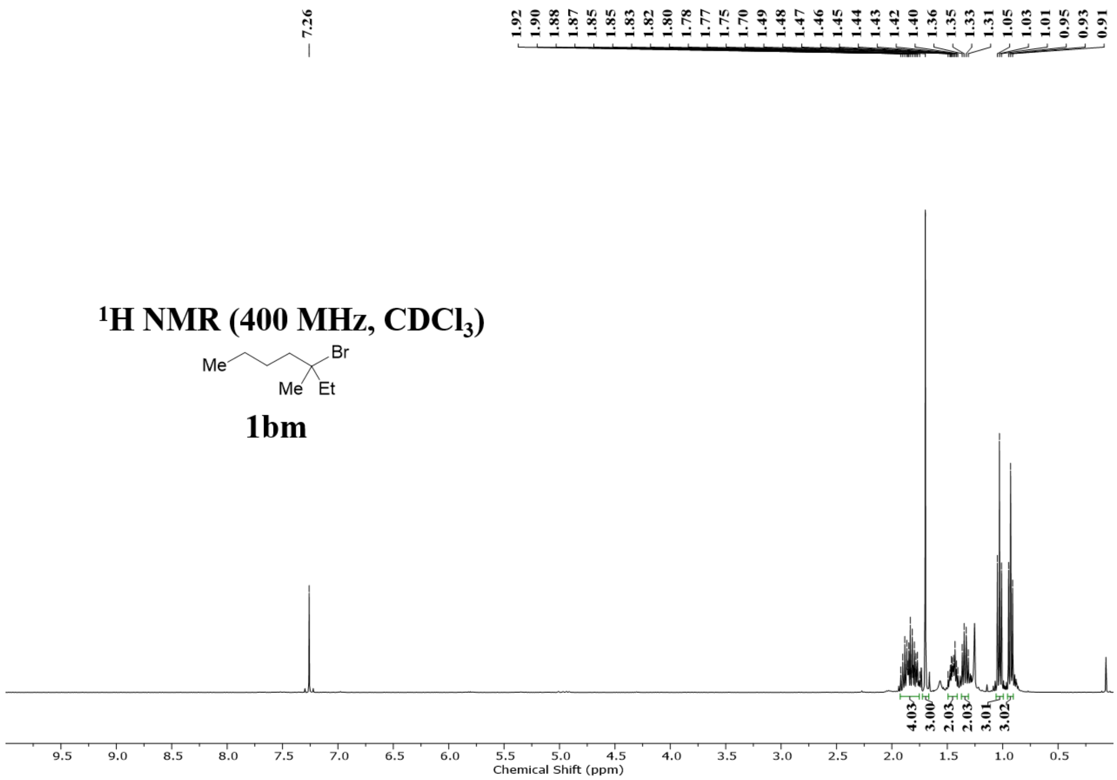

Supplementary Fig. 8. ^{1}H NMR of compound 1bm. The sample has been recorded in 400 MHz, $CDCl_3$ at 25 ºC

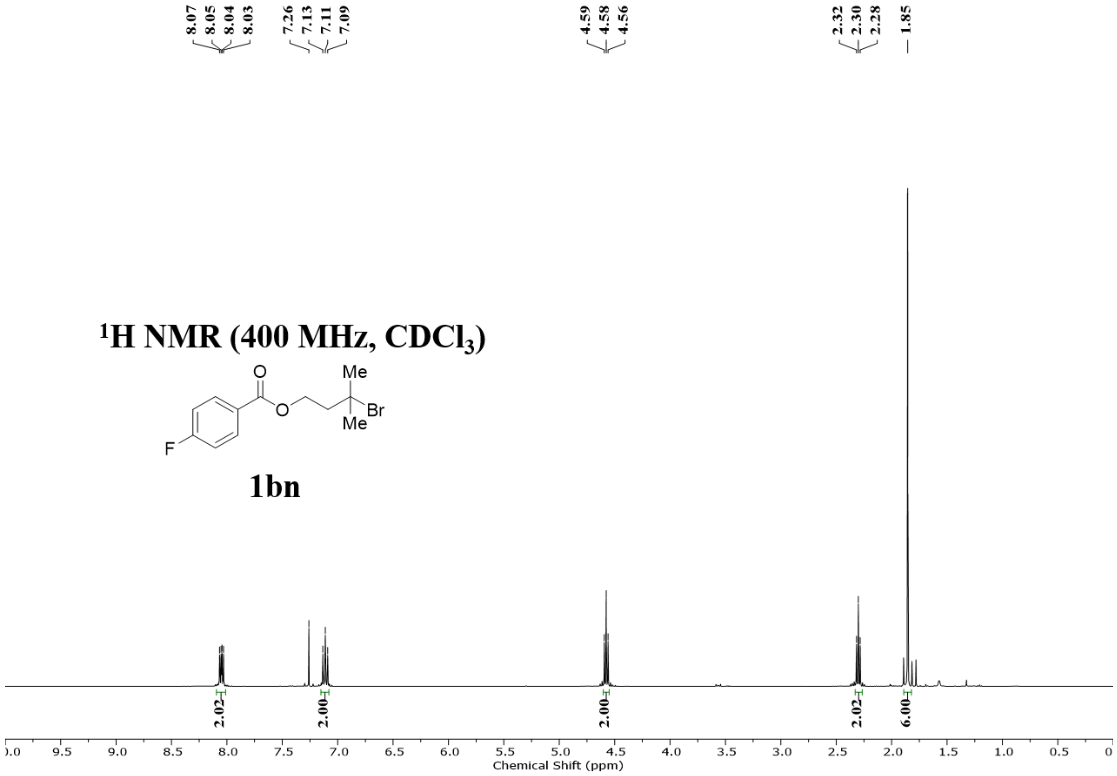

Supplementary Fig. 9. ^{1}H NMR of compound 1bn. The sample has been recorded in 400 MHz, $CDCl_3$ at 25 ºC

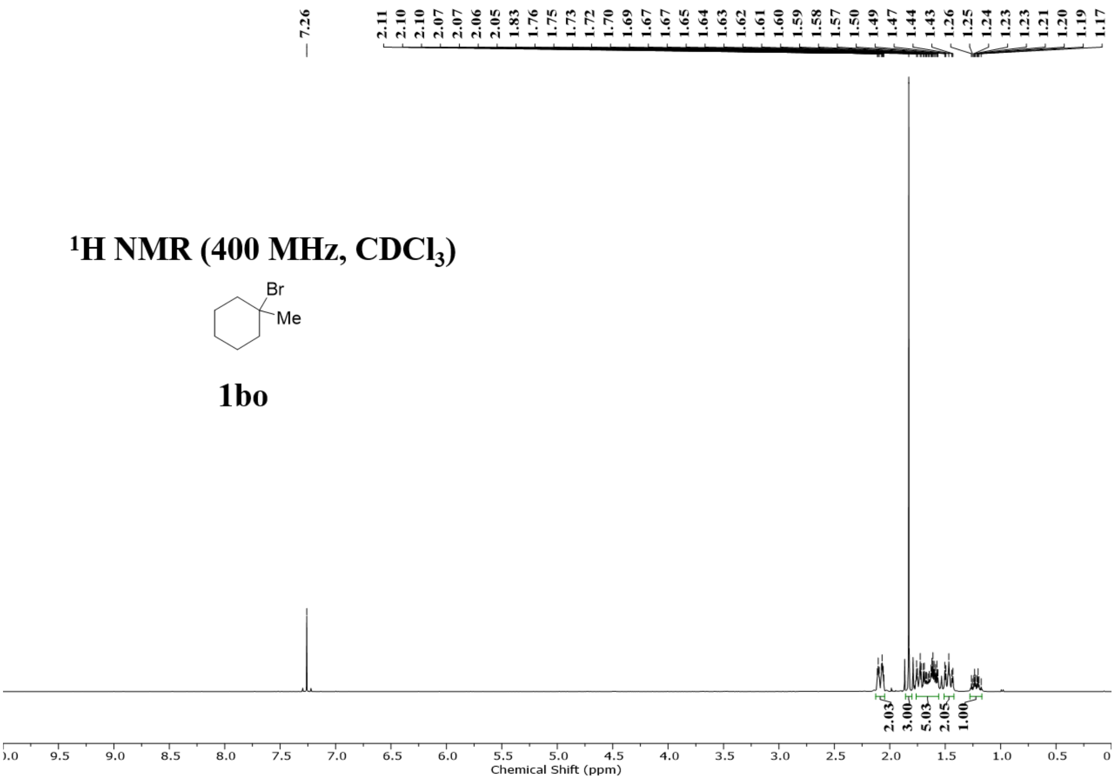

Supplementary Fig. 10. ¹H NMR of compound 1bo. The sample has been recorded in 400 MHz, $CDCl_3$ at 25 ºC

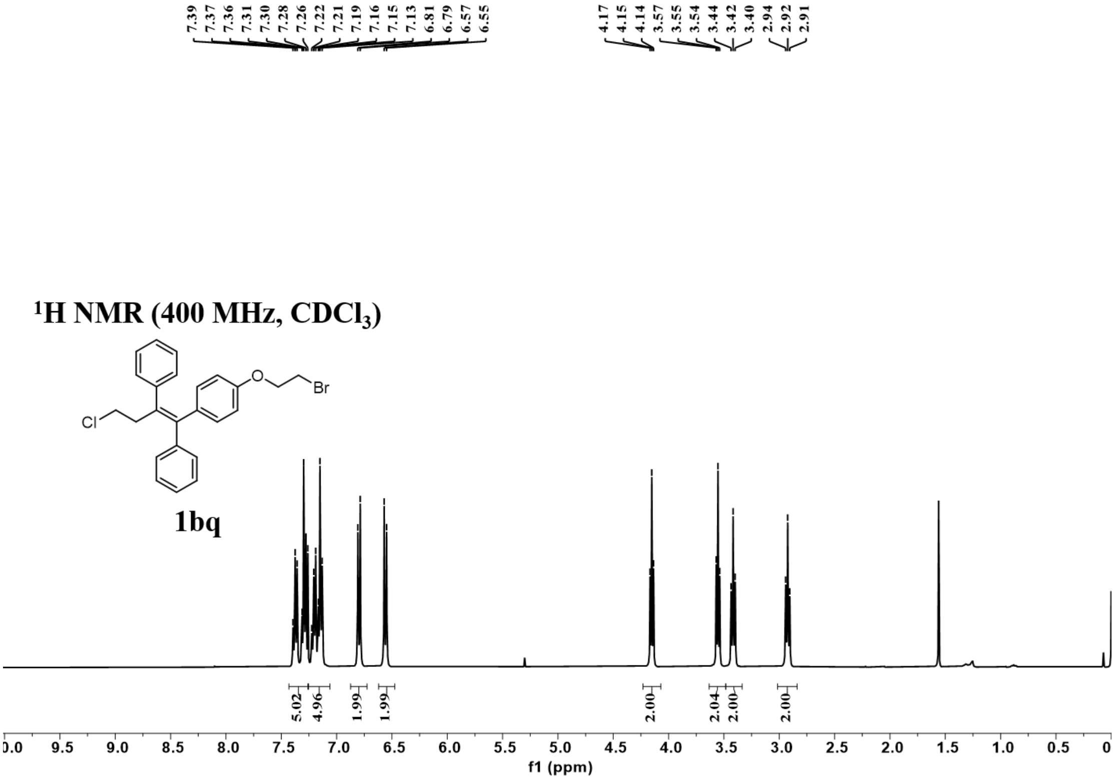

Supplementary Fig. 11. ¹H NMR of compound 1bq. The sample has been recorded in 400 MHz, $CDCl_3$ at 25 ºC

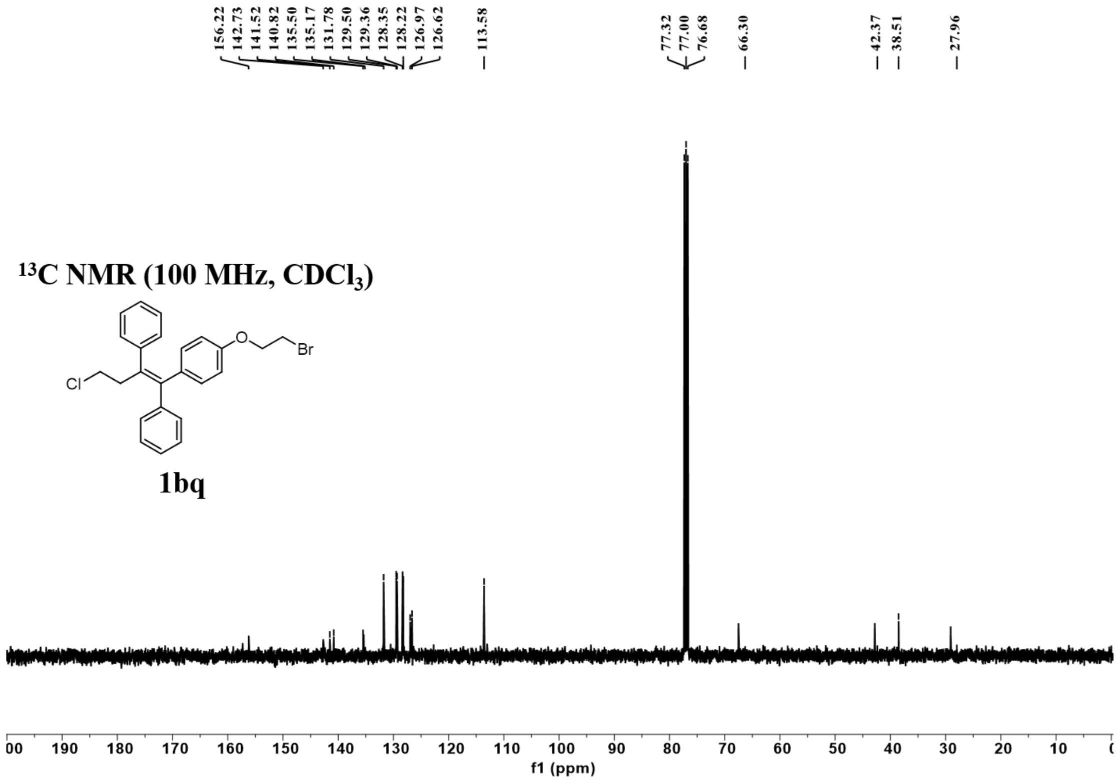

Supplementary Fig. 12. ^{13}C NMR of compound 1bq. The sample has been recorded in 100 MHz, $CDCl_3$ at 25 ºC

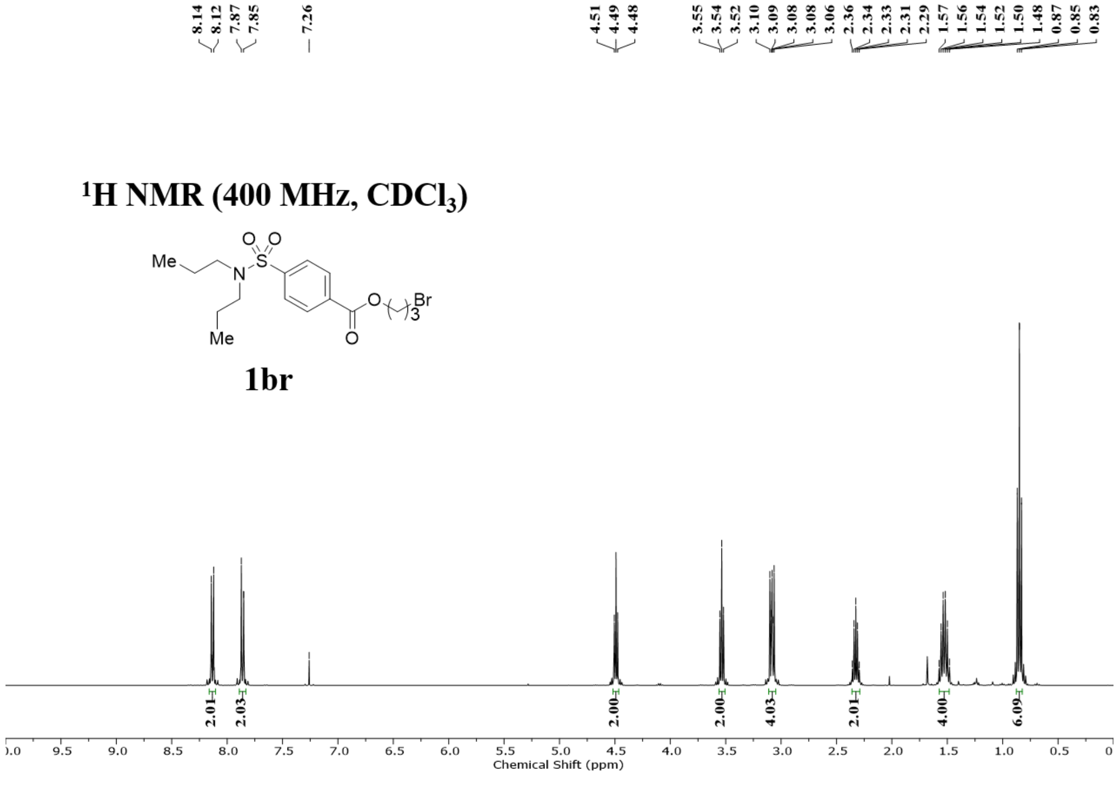

Supplementary Fig. 13. ^{1}H NMR of compound 1br. The sample has been recorded in 400 MHz, $CDCl_3$ at 25 ºC

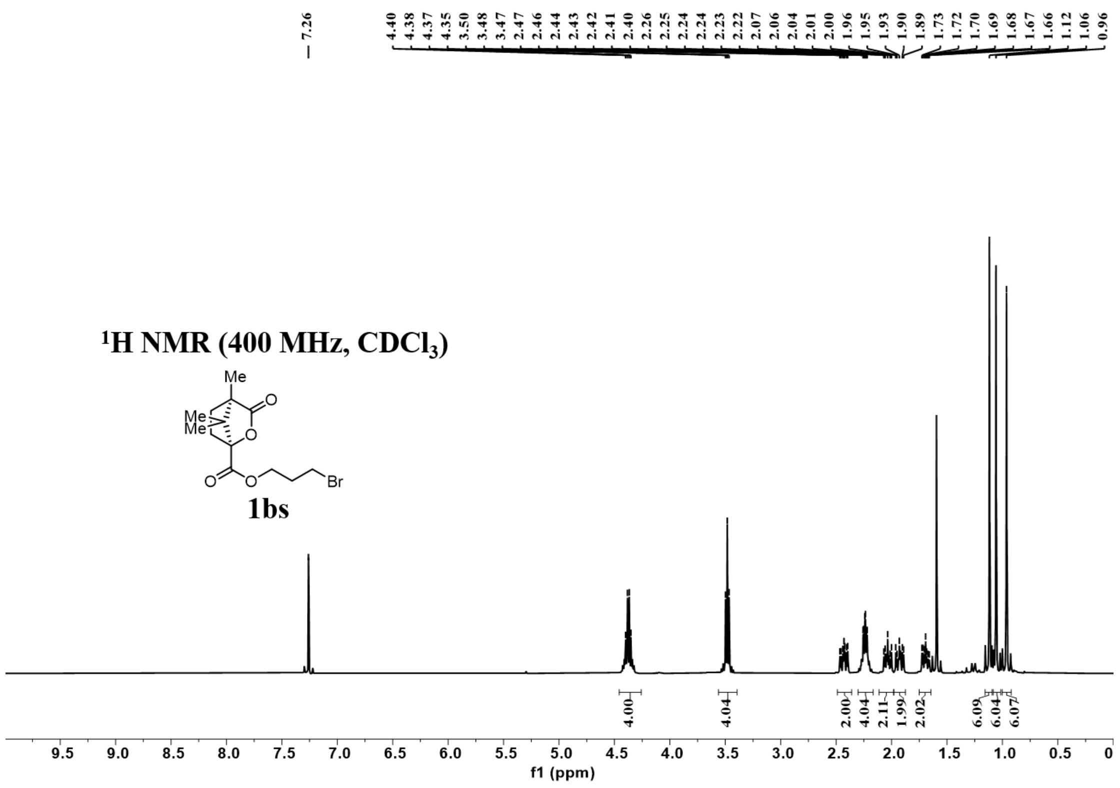

Supplementary Fig. 14. ^{1}H NMR of compound 1bs. The sample has been recorded in 400 MHz, $CDCl_3$ at 25 ºC

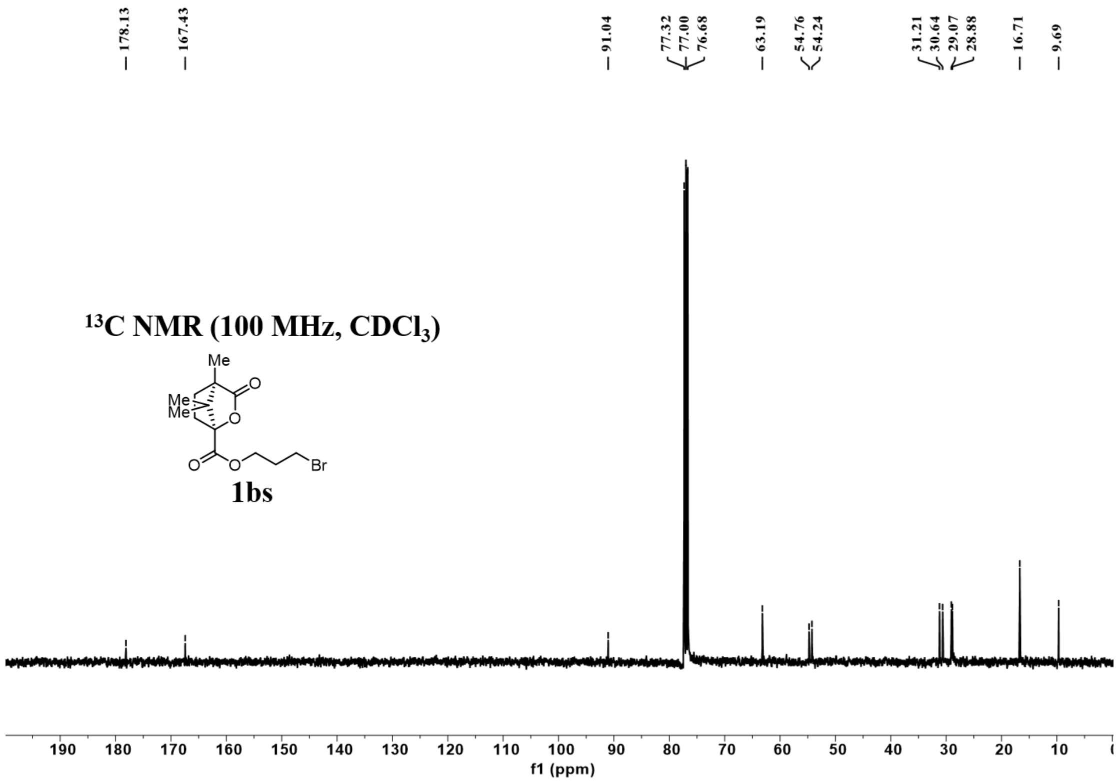

Supplementary Fig. 15. ^{13}C NMR of compound 1bs. The sample has been recorded in 100 MHz, $CDCl_3$ at 25 ºC

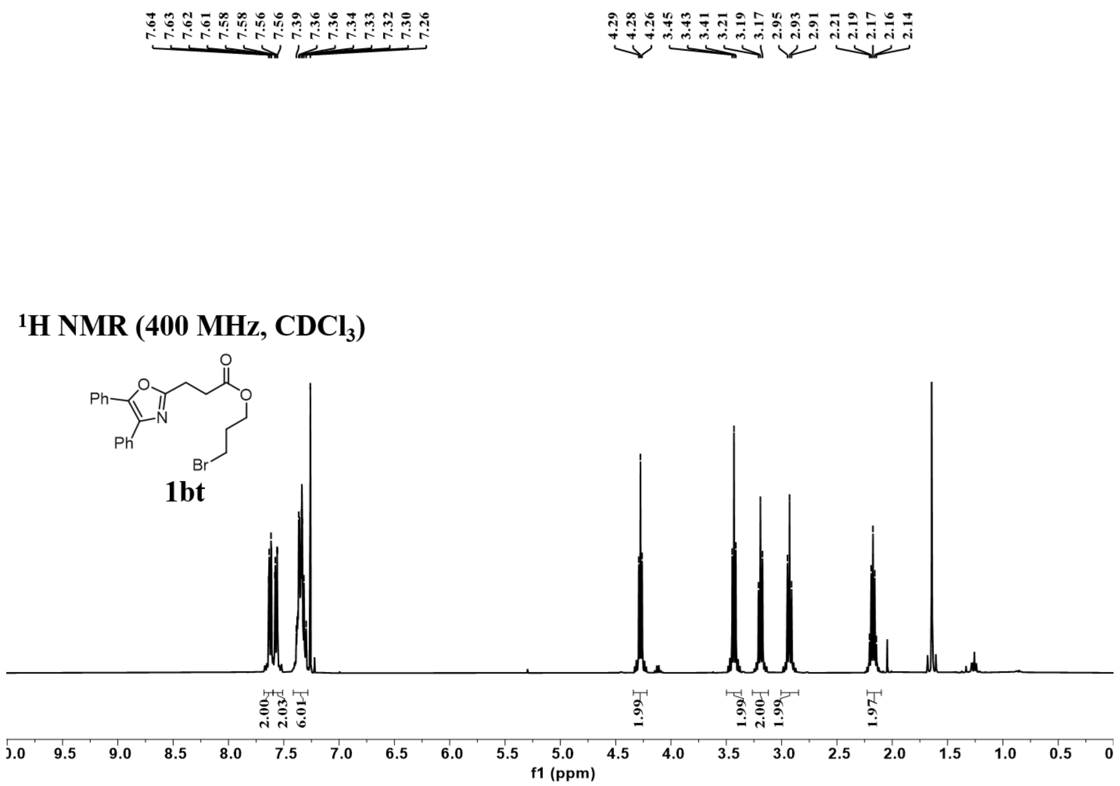

Supplementary Fig. 16. ^{1}H NMR of compound 1bt. The sample has been recorded in 400 MHz, $CDCl_3$ at 25 ºC

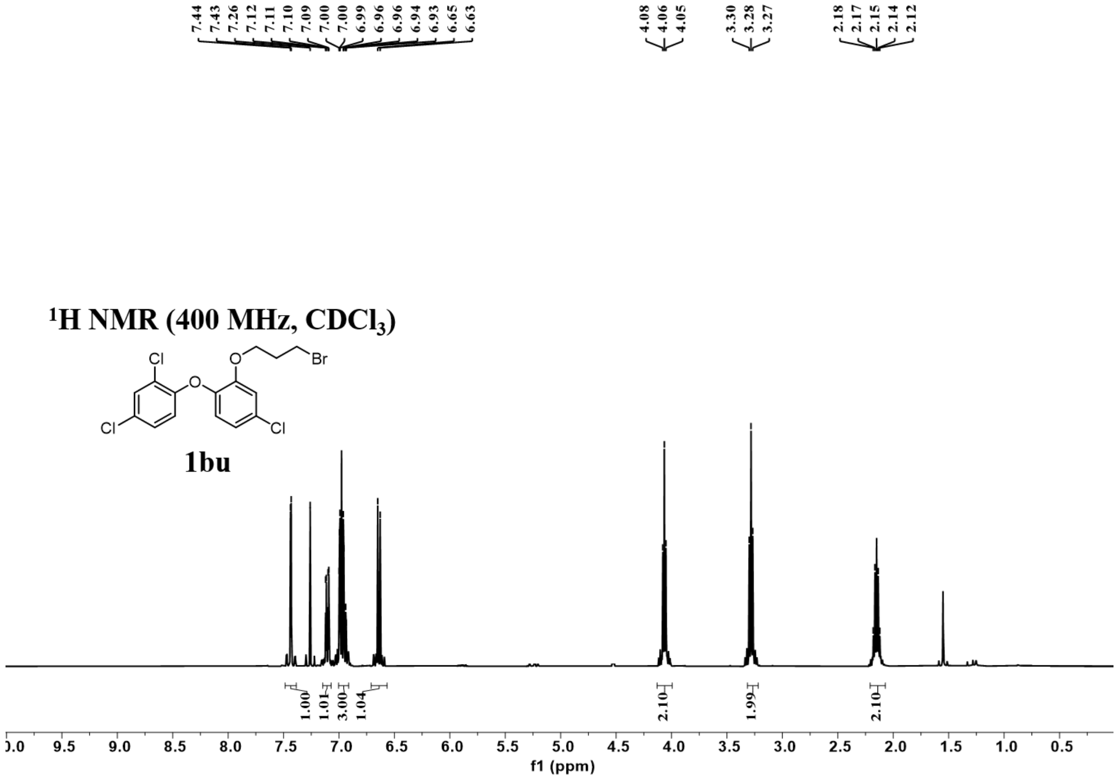

Supplementary Fig. 17. ^{1}H NMR of compound 1bu. The sample has been recorded in 400 MHz, $CDCl_3$ at 25 ºC

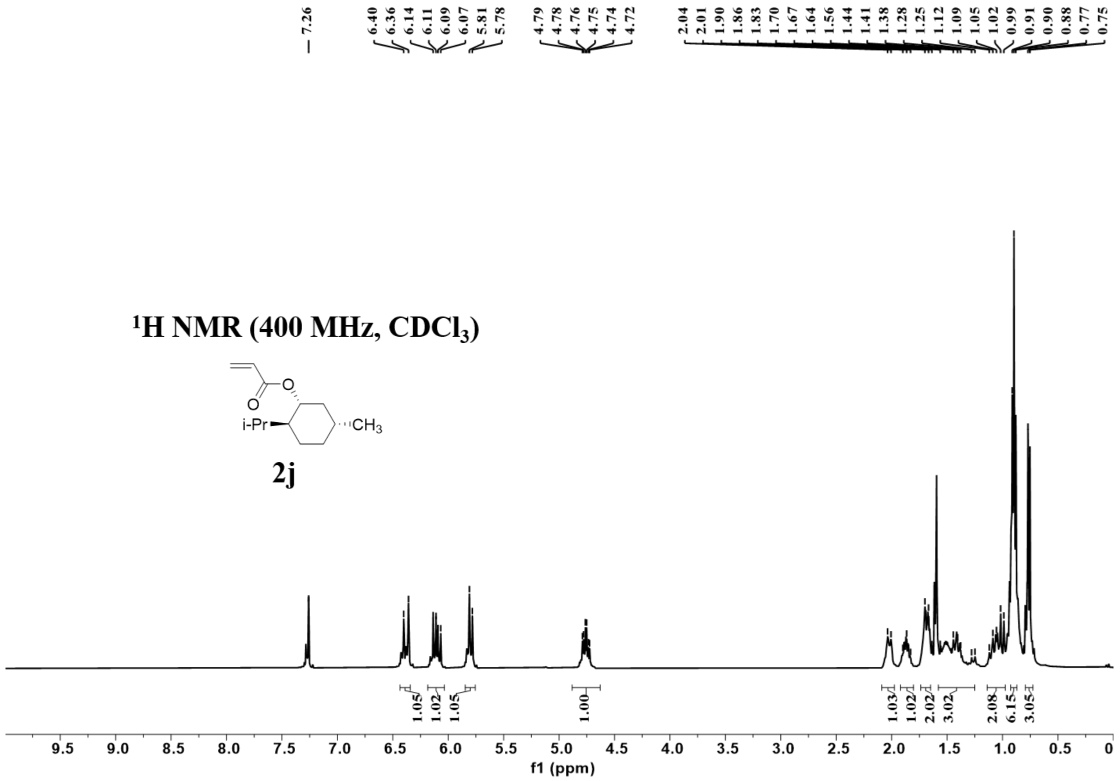

Supplementary Fig. 18. ^{1}H NMR of compound 2j. The sample has been recorded in 400 MHz, $CDCl_3$ at 25 ºC

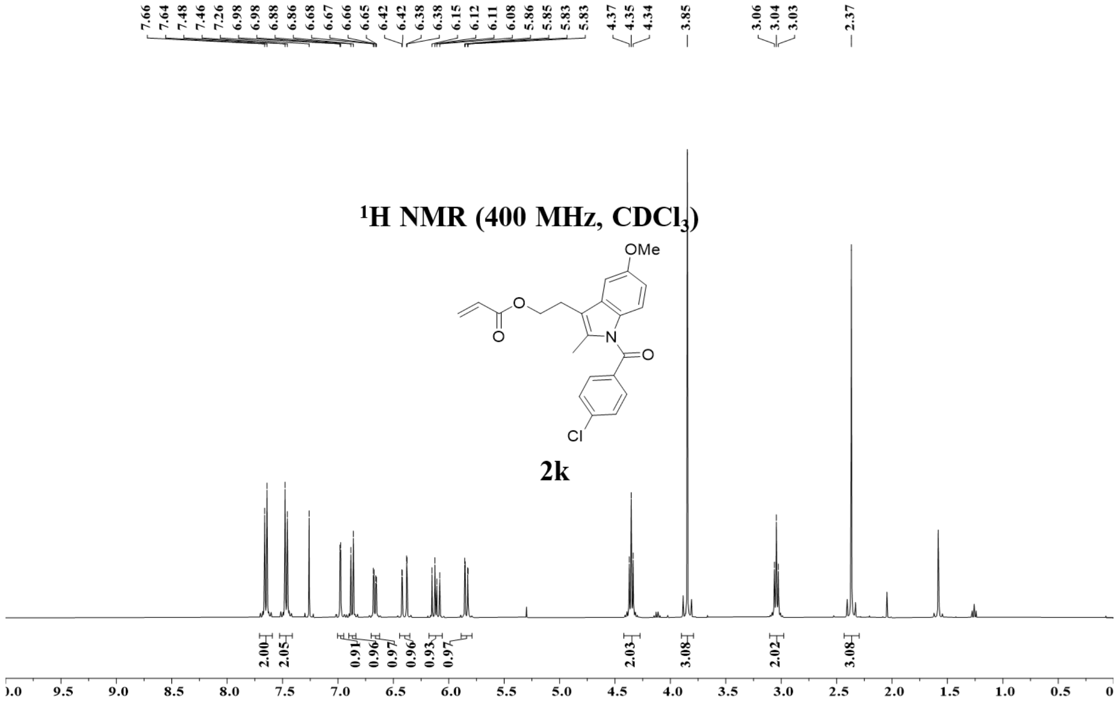

Supplementary Fig. 19. ^{1}H NMR of compound 2k. The sample has been recorded in 400 MHz, $CDCl_3$ at 25 ºC

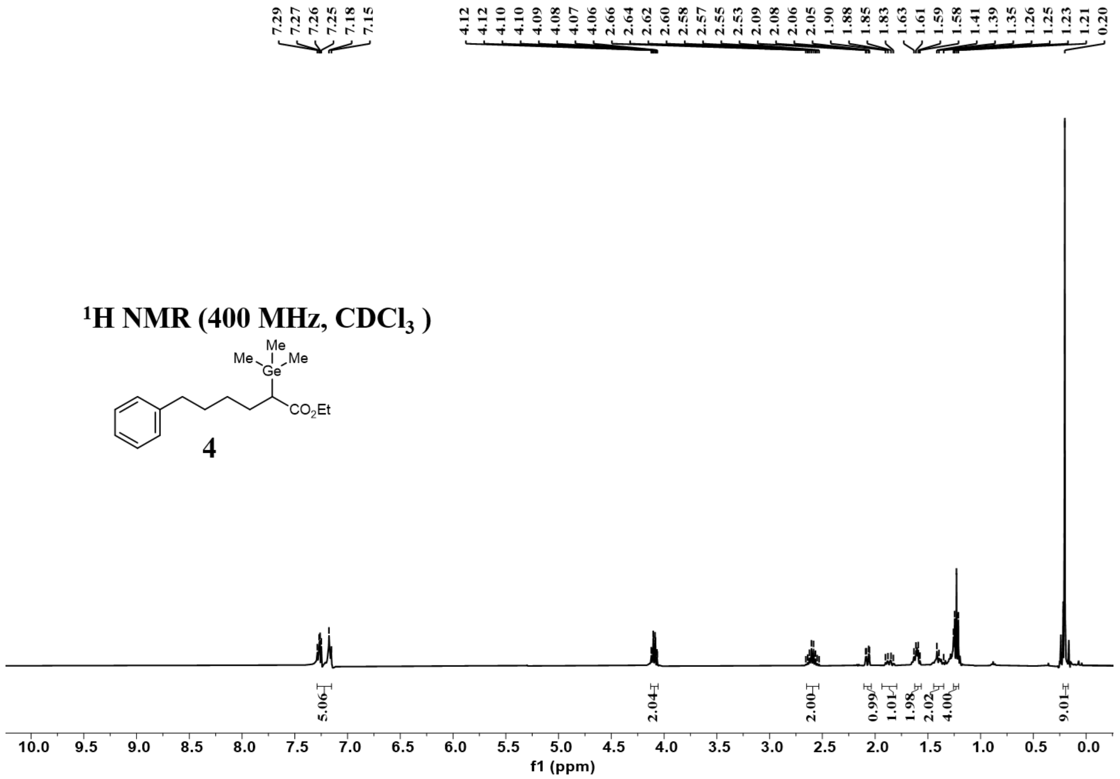

Supplementary Fig. 20. ^{1}H NMR of compound 4. The sample has been recorded in 400 MHz, $CDCl_3$ at 25 ºC

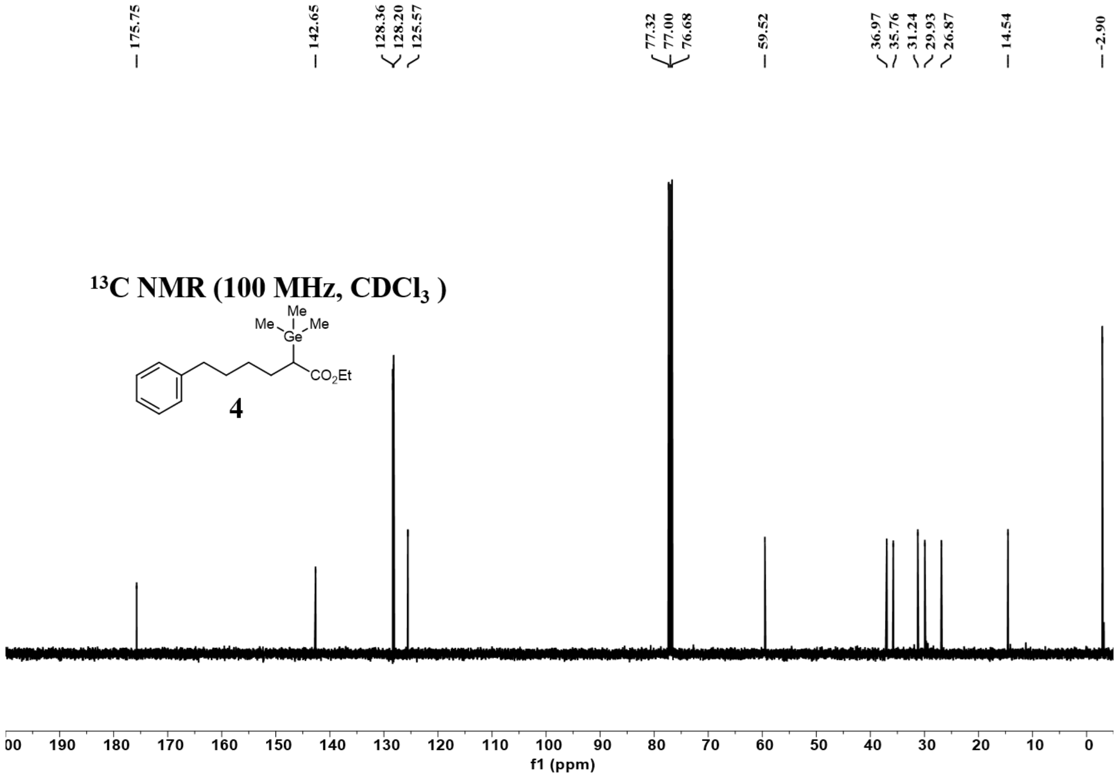

Supplementary Fig. 21. ^{13}C NMR of compound 4. The sample has been recorded in 100 MHz, $CDCl_3$ at 25 ºC

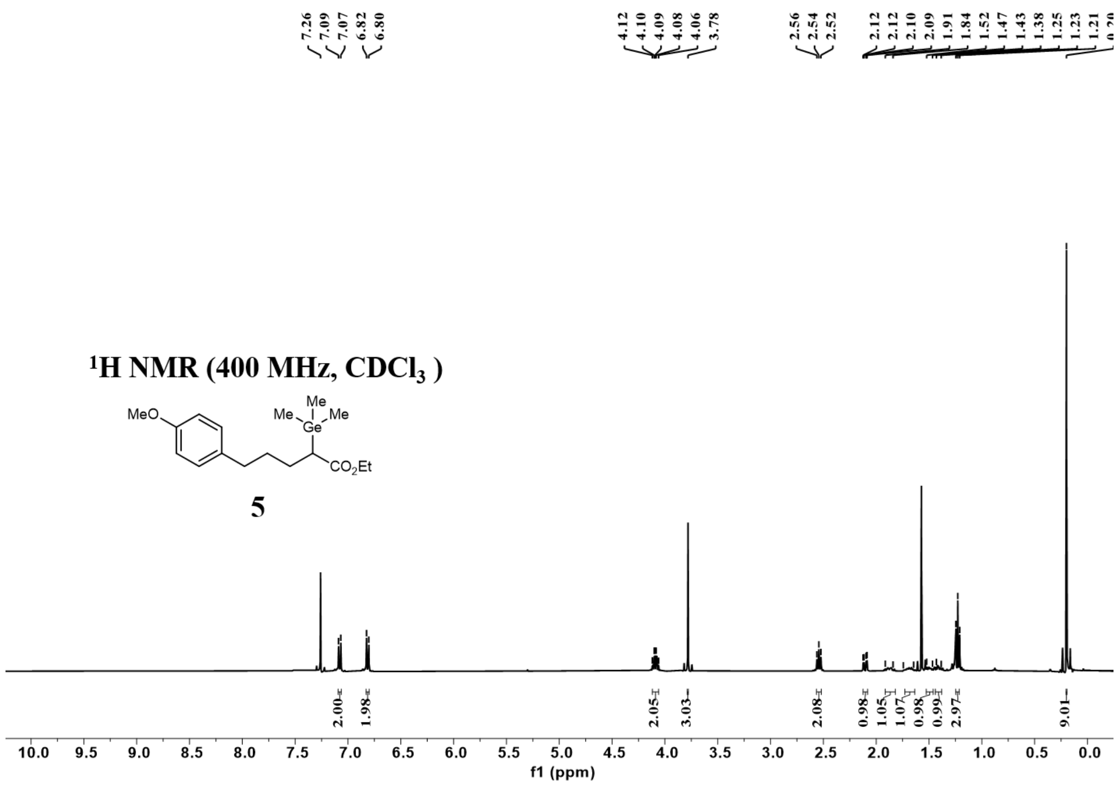

Supplementary Fig. 22. ^{1}H NMR of compound 5. The sample has been recorded in 400 MHz, $CDCl_3$ at 25 ºC

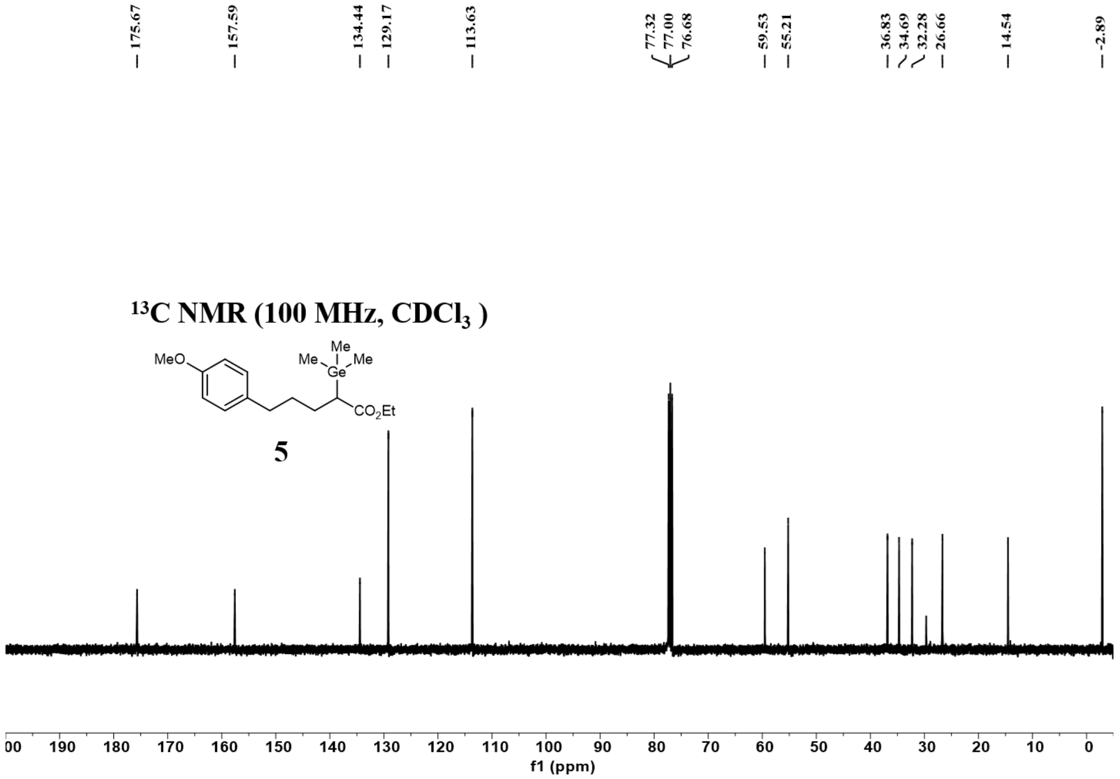

Supplementary Fig. 23. ^{13}C NMR of compound 5. The sample has been recorded in 100 MHz, $CDCl_3$ at 25 ºC

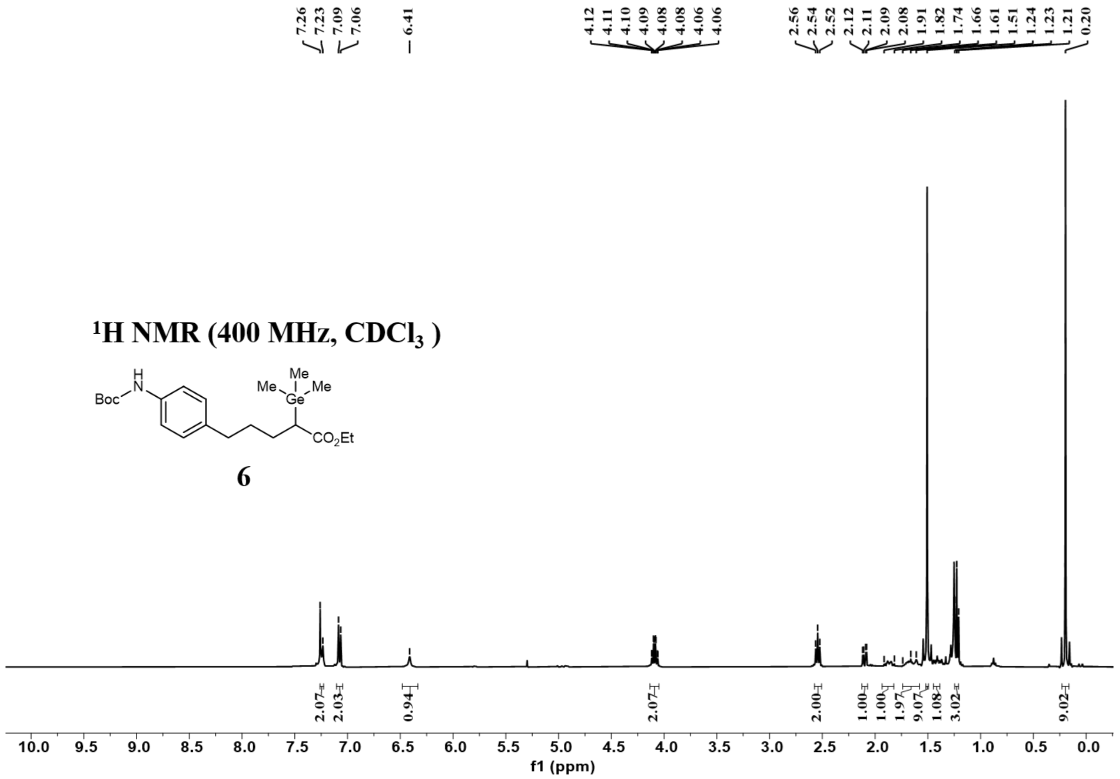

Supplementary Fig. 24. ^{1}H NMR of compound 6. The sample has been recorded in 400 MHz, $CDCl_3$ at 25 ºC

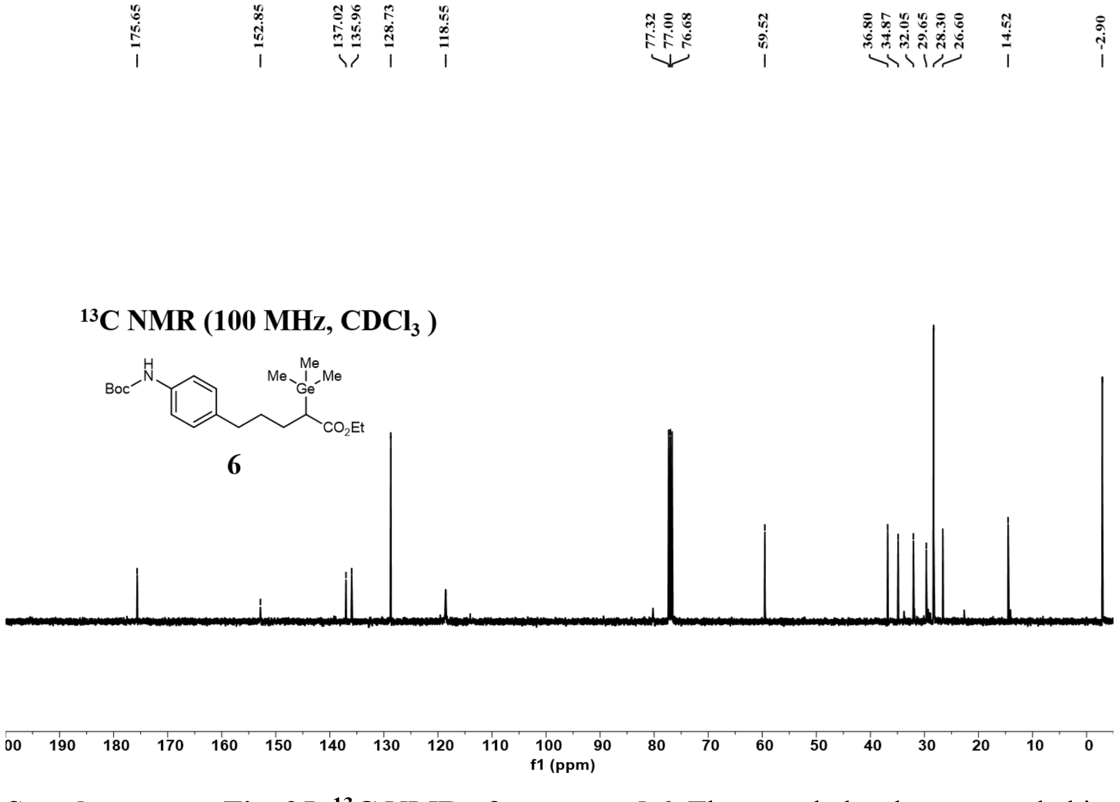

Supplementary Fig. 25. ^{13}C NMR of compound 6. The sample has been recorded in 100 MHz, $CDCl_3$ at 25 ºC

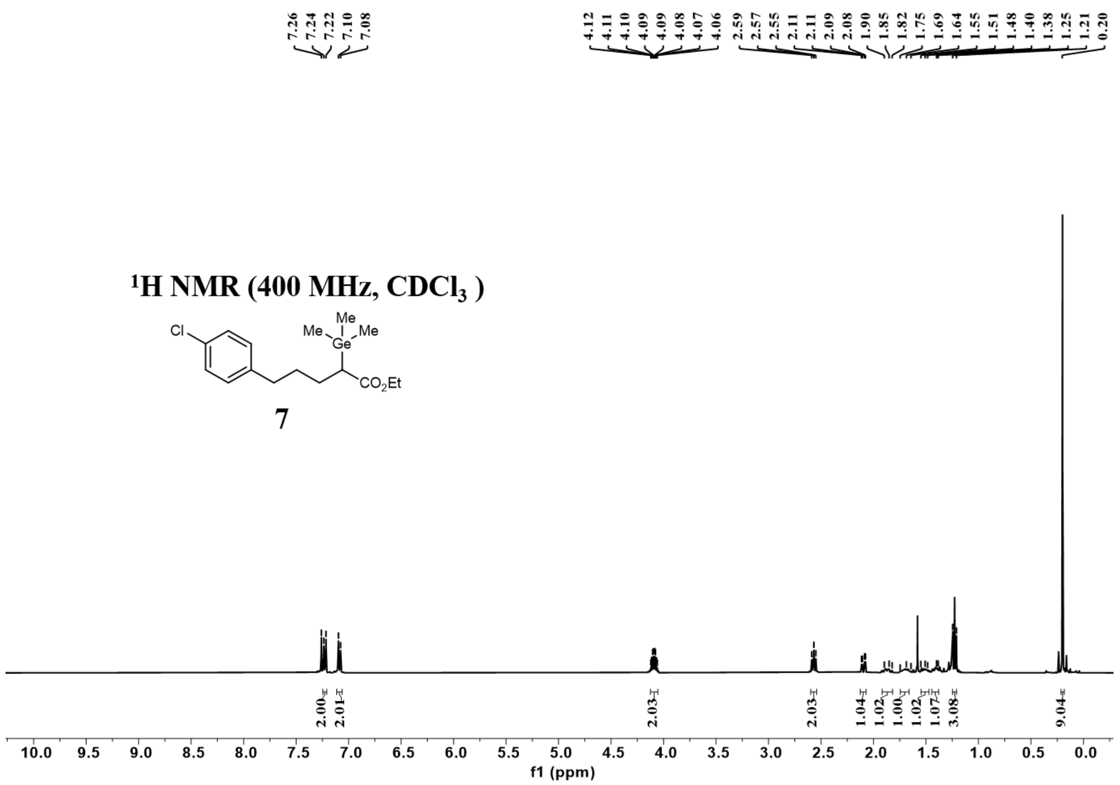

Supplementary Fig. 26. ^{1}H NMR of compound 7. The sample has been recorded in 400 MHz, $CDCl_3$ at 25 ºC

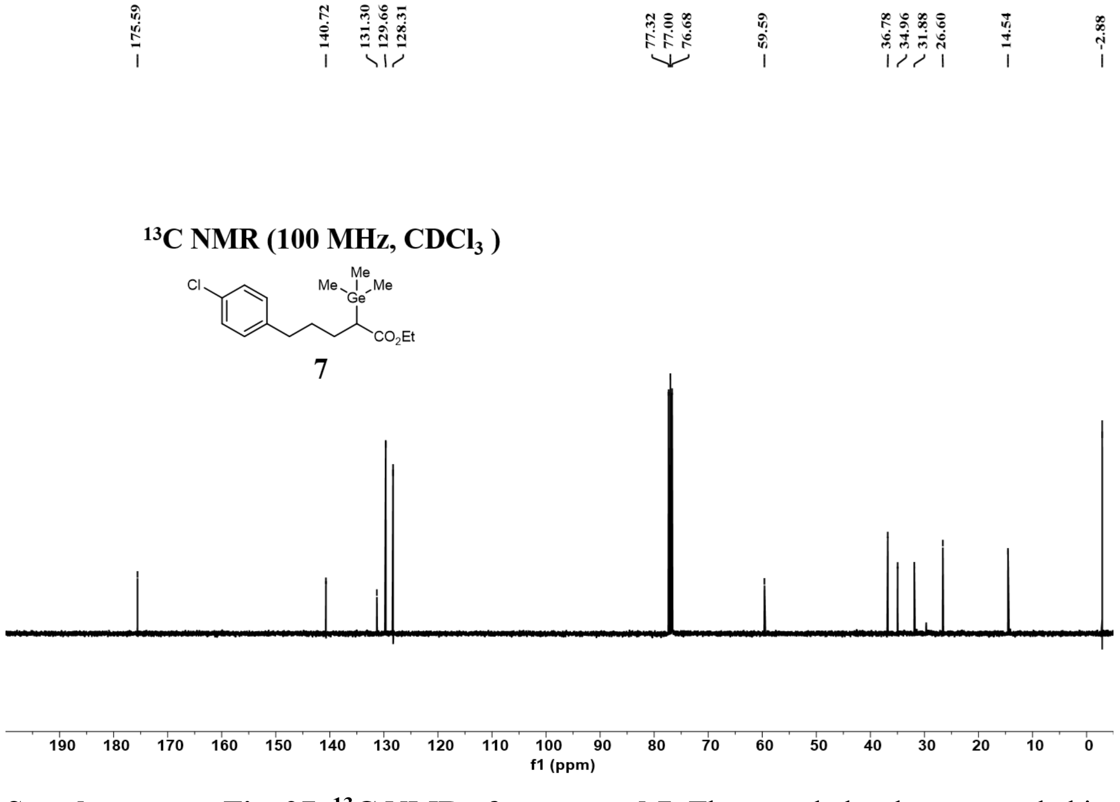

Supplementary Fig. 27. ^{13}C NMR of compound 7. The sample has been recorded in 100 MHz, $CDCl_3$ at 25 ºC

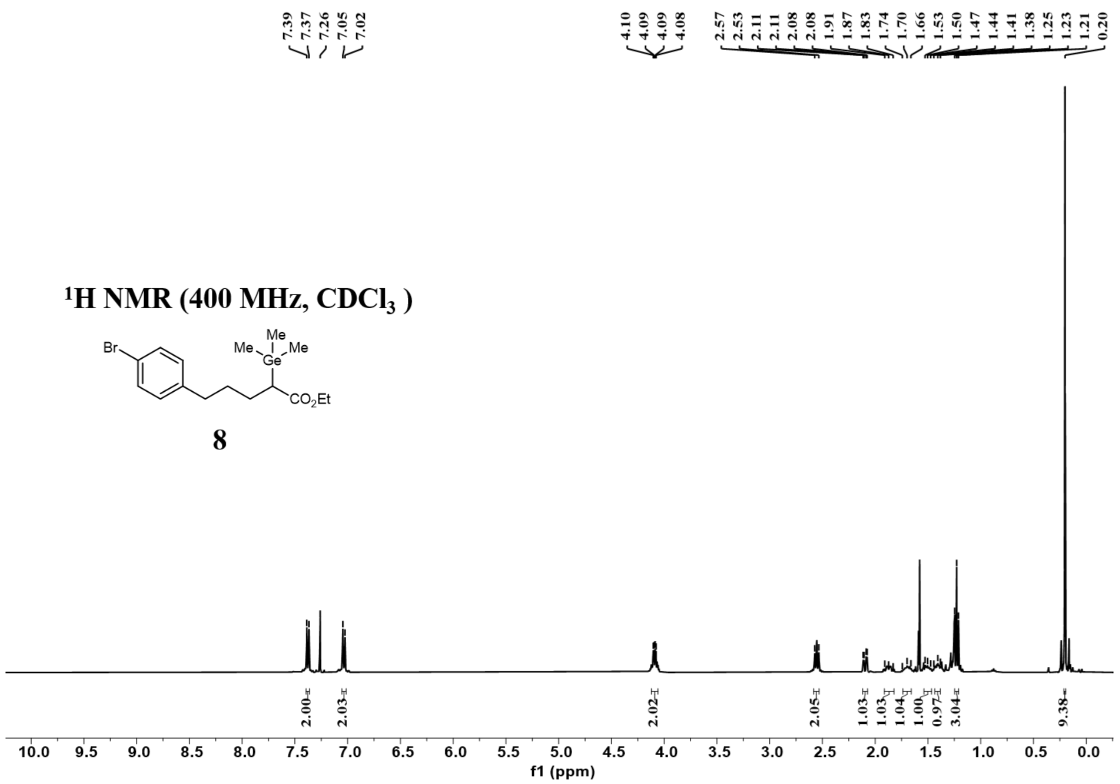

Supplementary Fig. 28. ^{1}H NMR of compound 8. The sample has been recorded in 400 MHz, $CDCl_3$ at 25 ºC

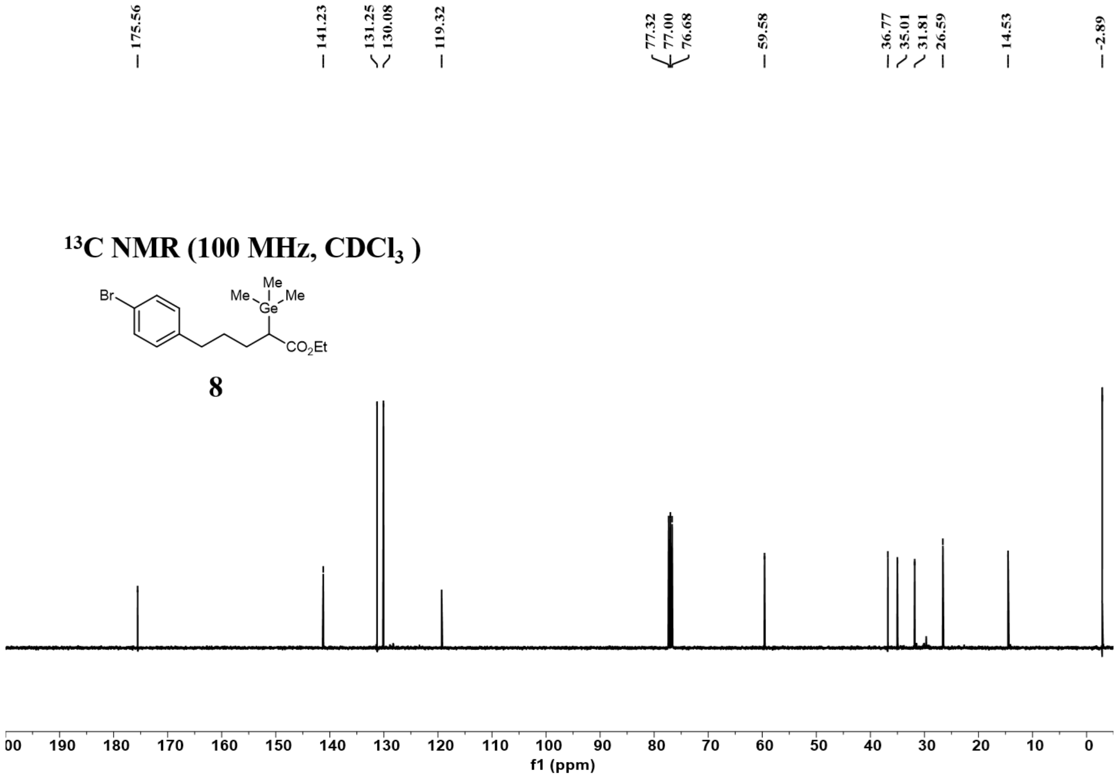

Supplementary Fig. 29. ^{13}C NMR of compound 8. The sample has been recorded in 100 MHz, $CDCl_3$ at 25 ºC

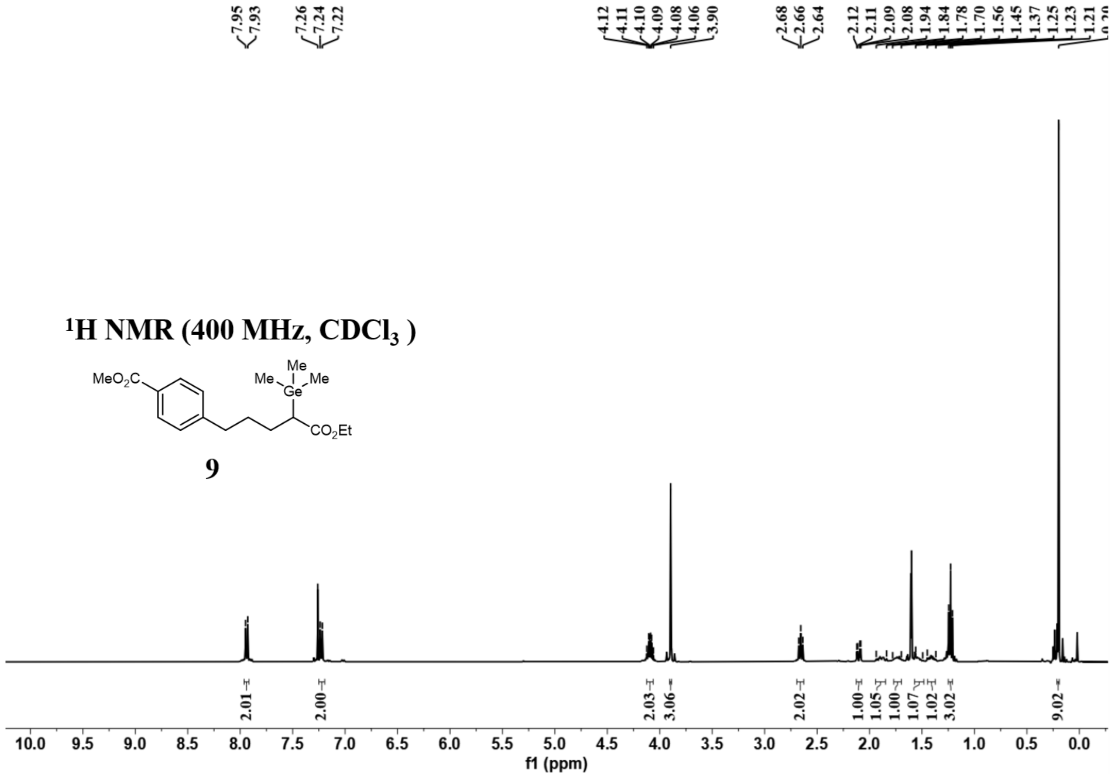

Supplementary Fig. 30. ^{1}H NMR of compound 9. The sample has been recorded in 400 MHz, $CDCl_3$ at 25 ºC

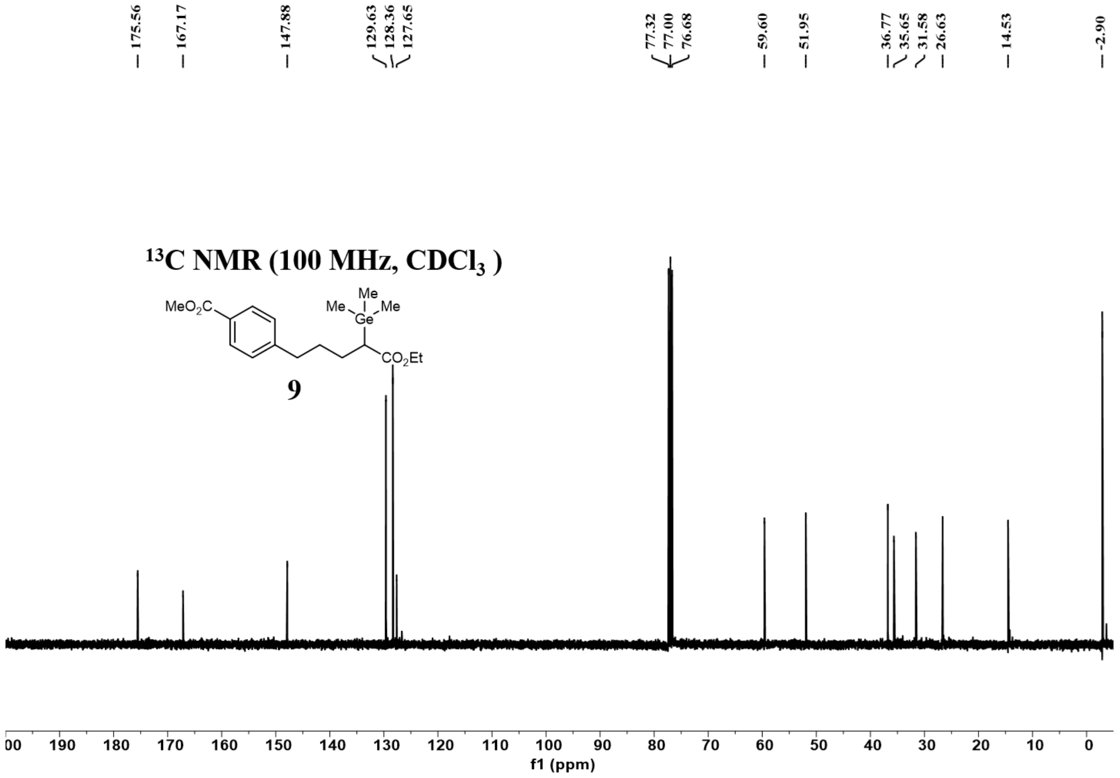

Supplementary Fig. 31. ^{13}C NMR of compound 9. The sample has been recorded in 100 MHz, $CDCl_3$ at 25 ºC

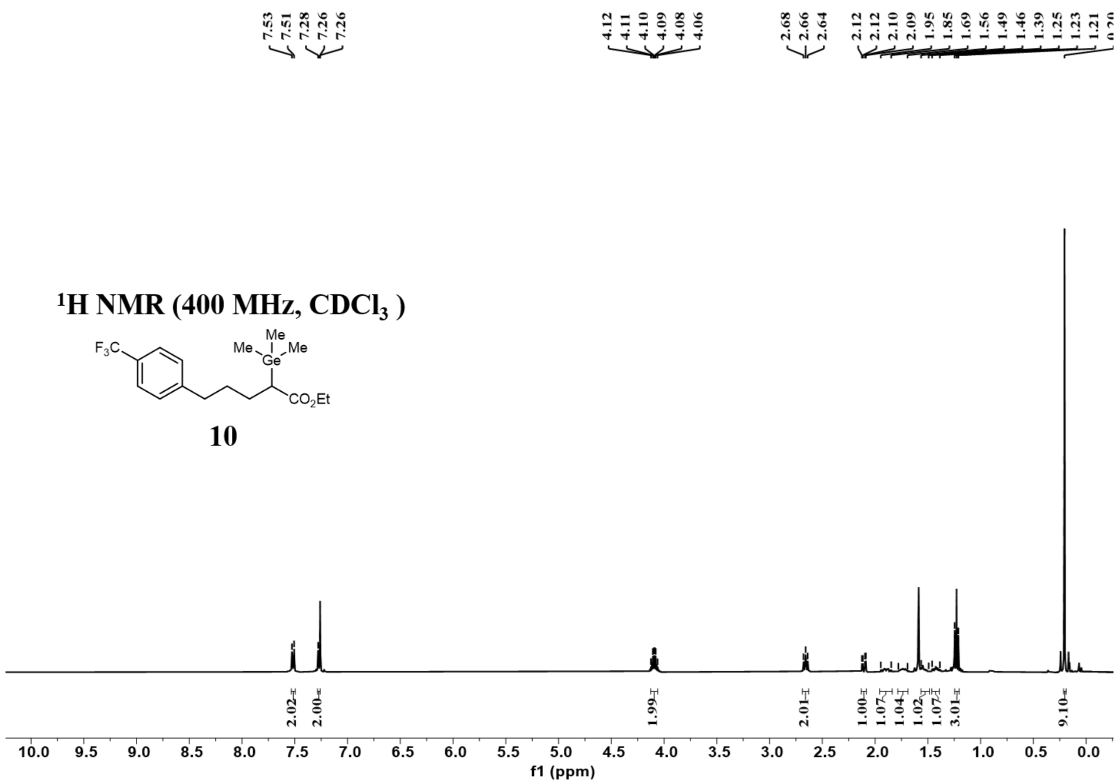

Supplementary Fig. 32. ^{1}H NMR of compound 10. The sample has been recorded in 400 MHz, $CDCl_3$ at 25 ºC

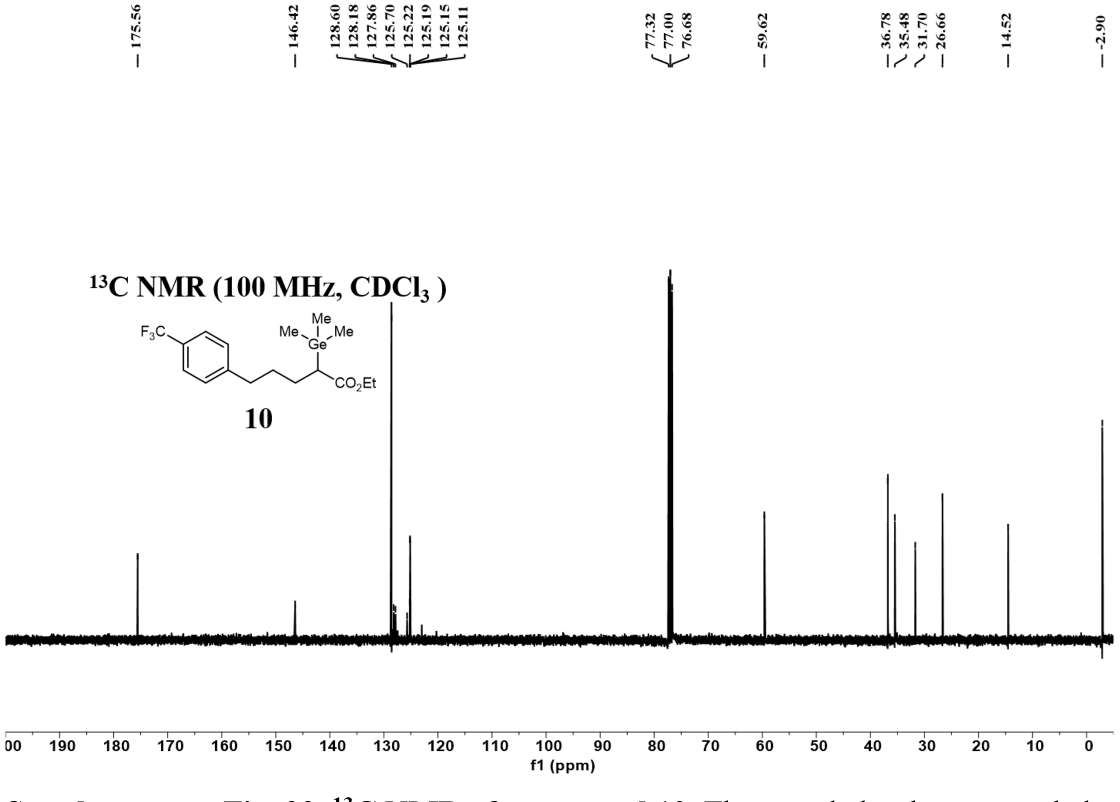

Supplementary Fig. 33. ^{13}C NMR of compound 10. The sample has been recorded in 100 MHz, $CDCl_3$ at 25 ºC

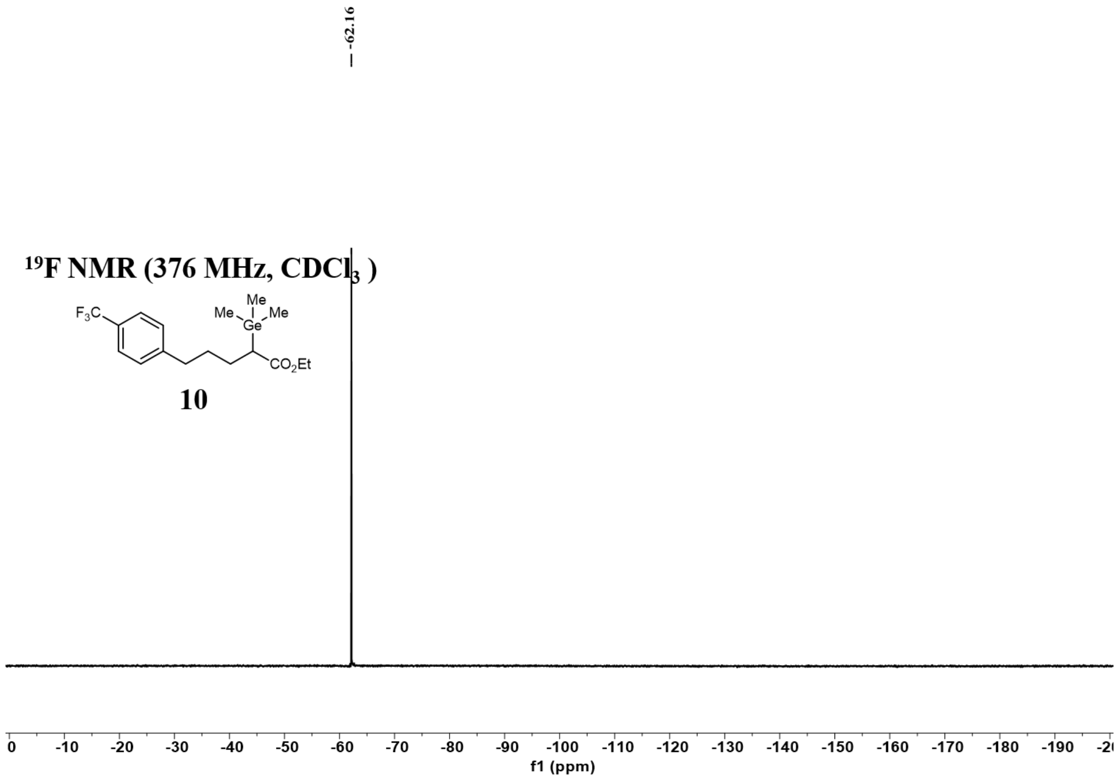

Supplementary Fig. 34. ^{19}F NMR of compound 10. The sample has been recorded in 376 MHz, $CDCl_3$ at 25 ºC

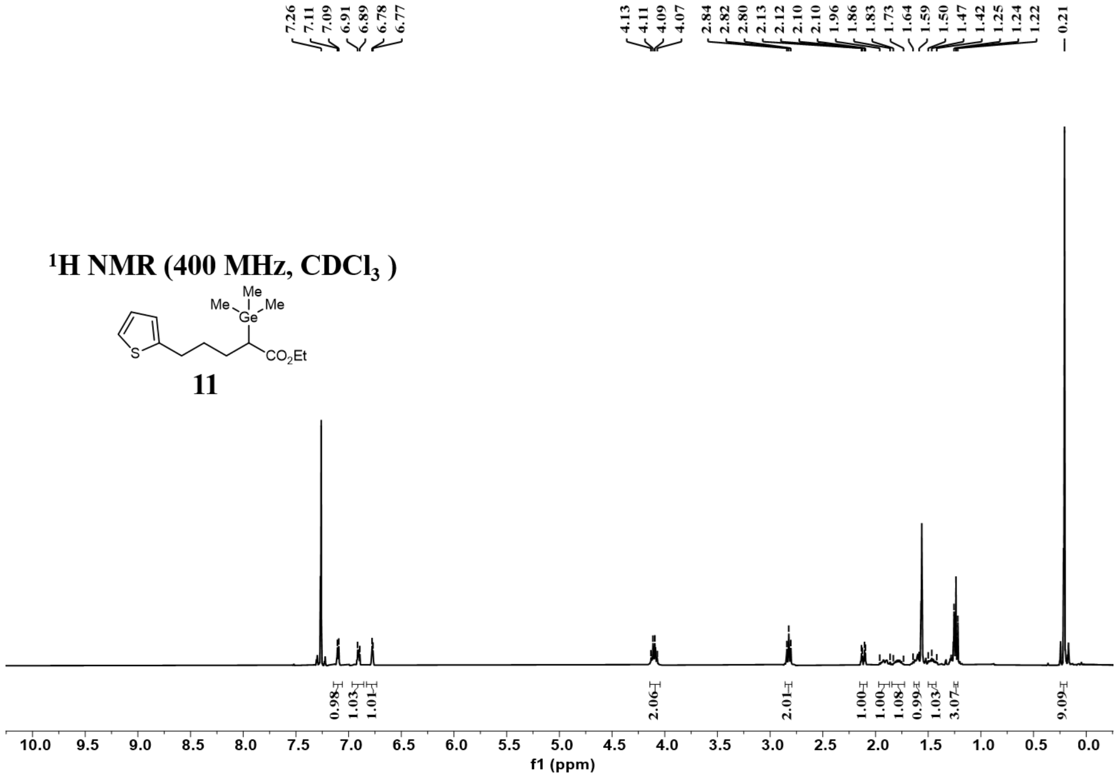

Supplementary Fig. 35. ^{1}H NMR of compound 11. The sample has been recorded in 400 MHz, $CDCl_3$ at 25 ºC

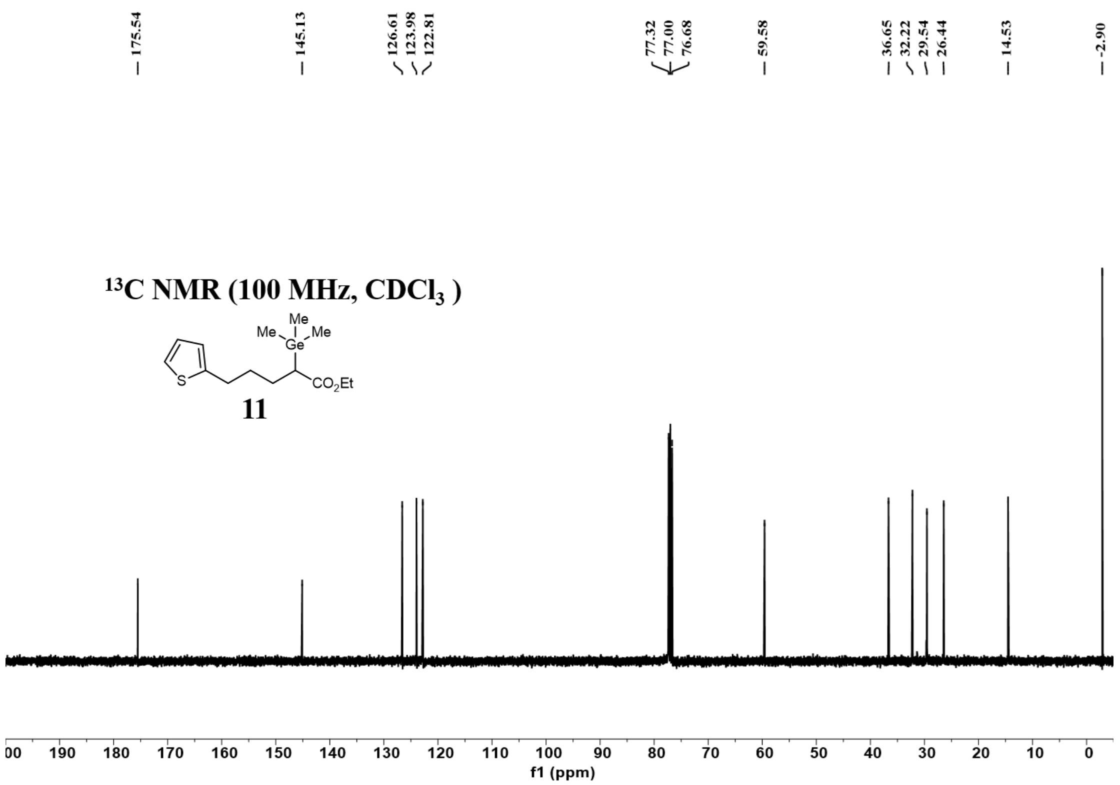

Supplementary Fig. 36. ^{13}C NMR of compound 11. The sample has been recorded in 100 MHz, $CDCl_3$ at 25 ºC

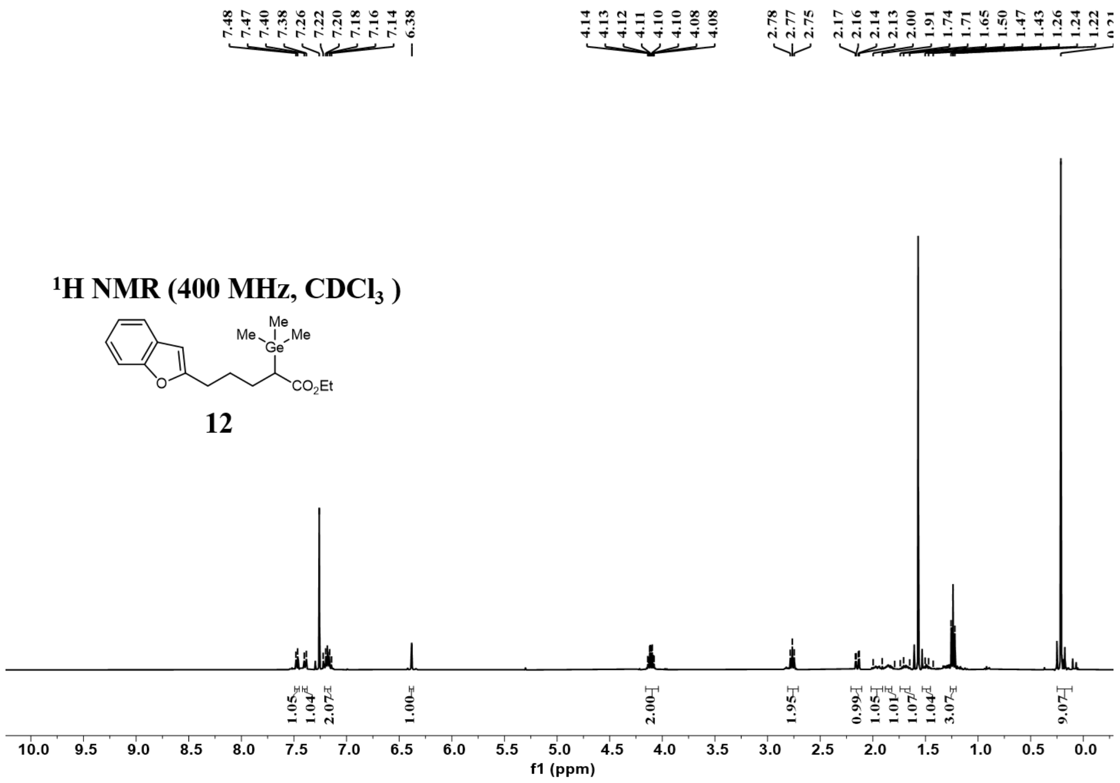

Supplementary Fig. 37. ^{1}H NMR of compound 12. The sample has been recorded in 400 MHz, $CDCl_3$ at 25 ºC

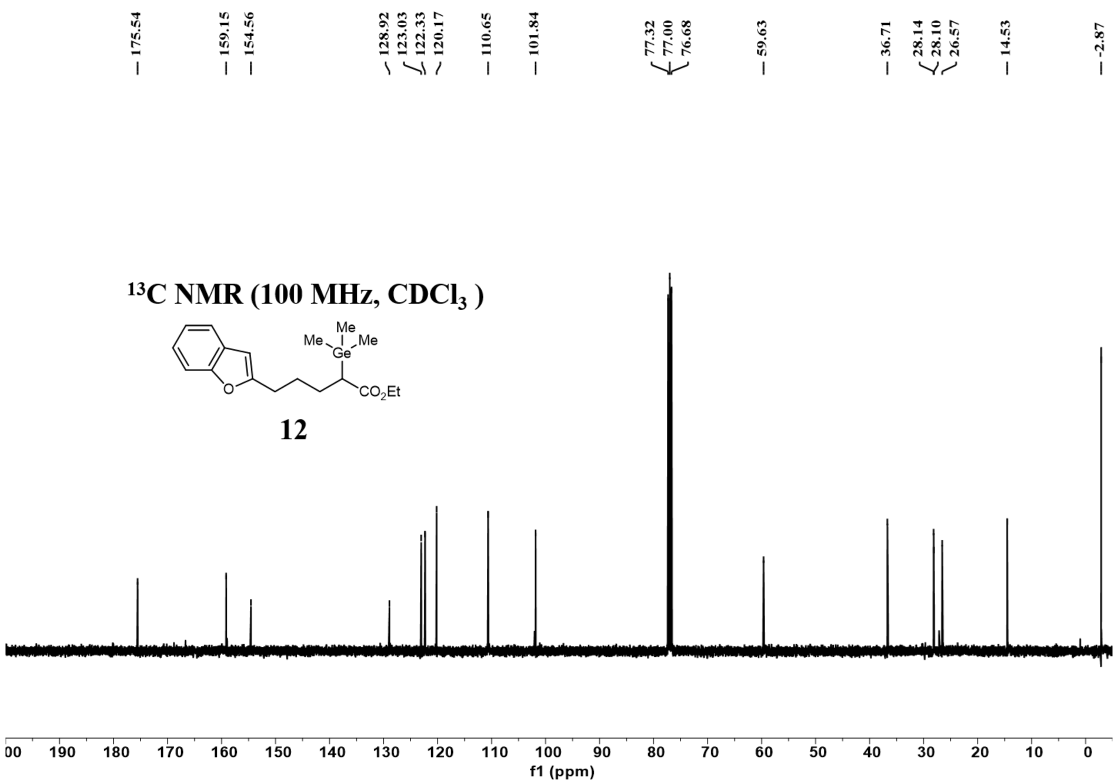

Supplementary Fig. 38. ^{13}C NMR of compound 12. The sample has been recorded in 100 MHz, $CDCl_3$ at 25 ºC

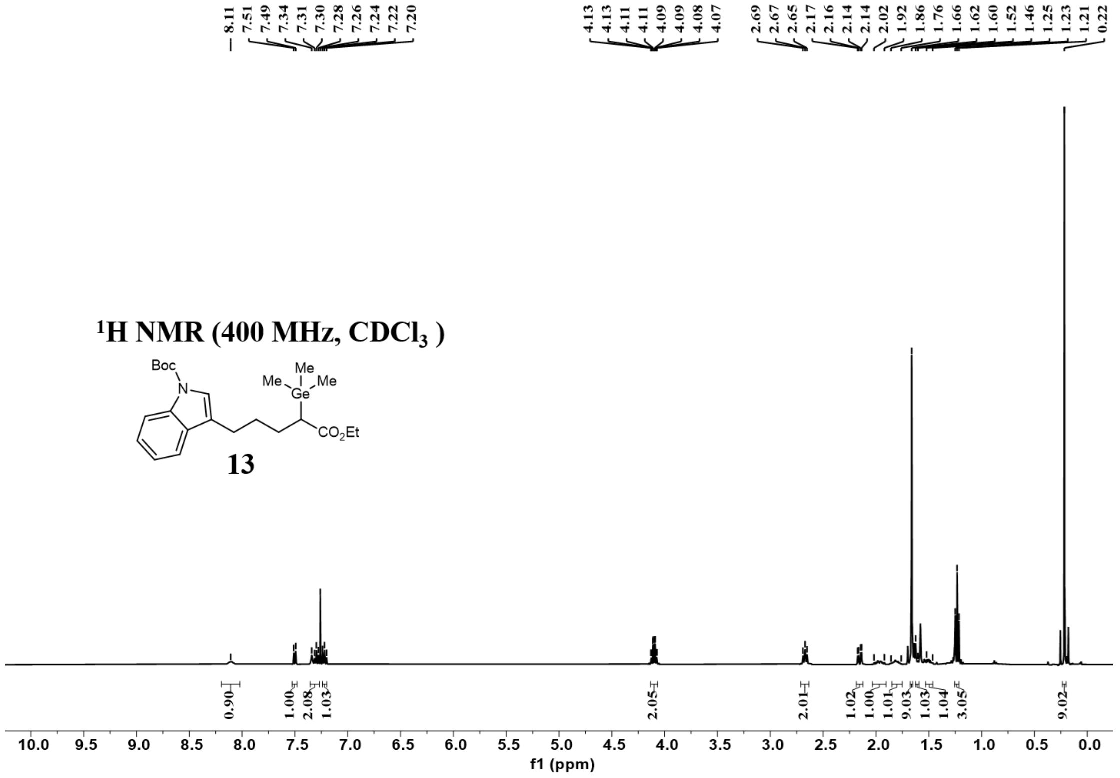

Supplementary Fig. 39. ^{1}H NMR of compound 13. The sample has been recorded in 400 MHz, $CDCl_3$ at 25 ºC

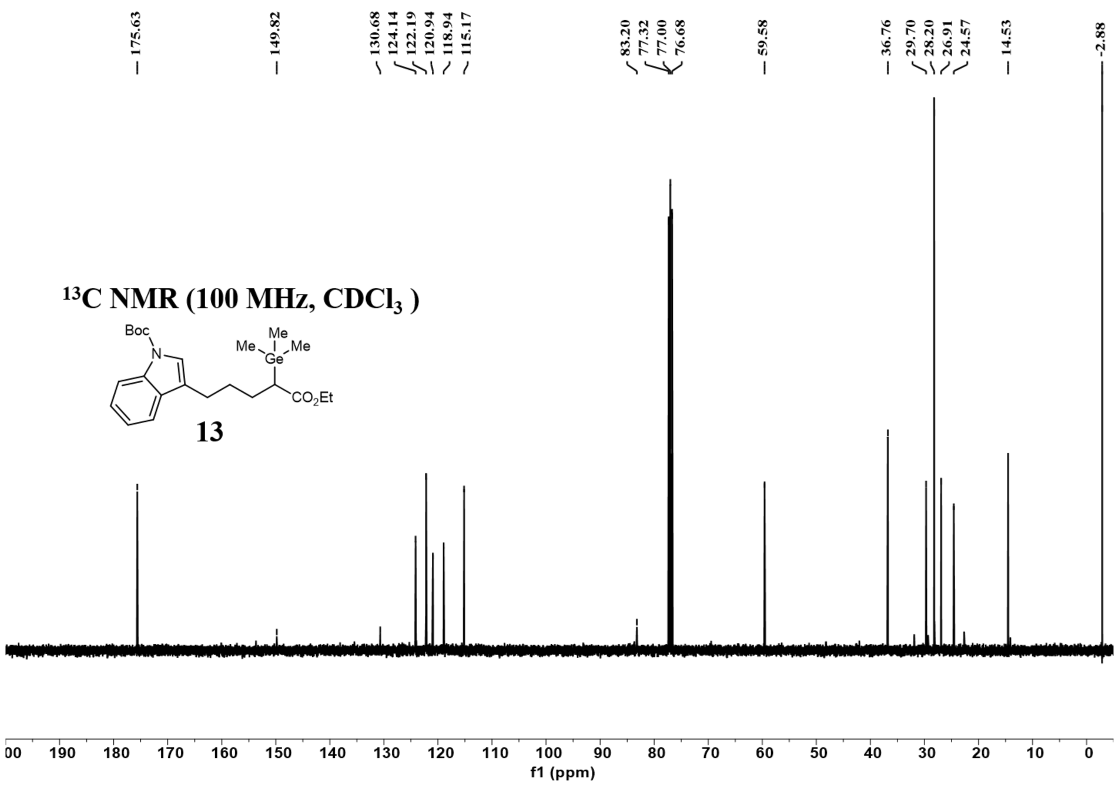

Supplementary Fig. 40. ^{13}C NMR of compound 13. The sample has been recorded in 100 MHz, $CDCl_3$ at 25 ºC

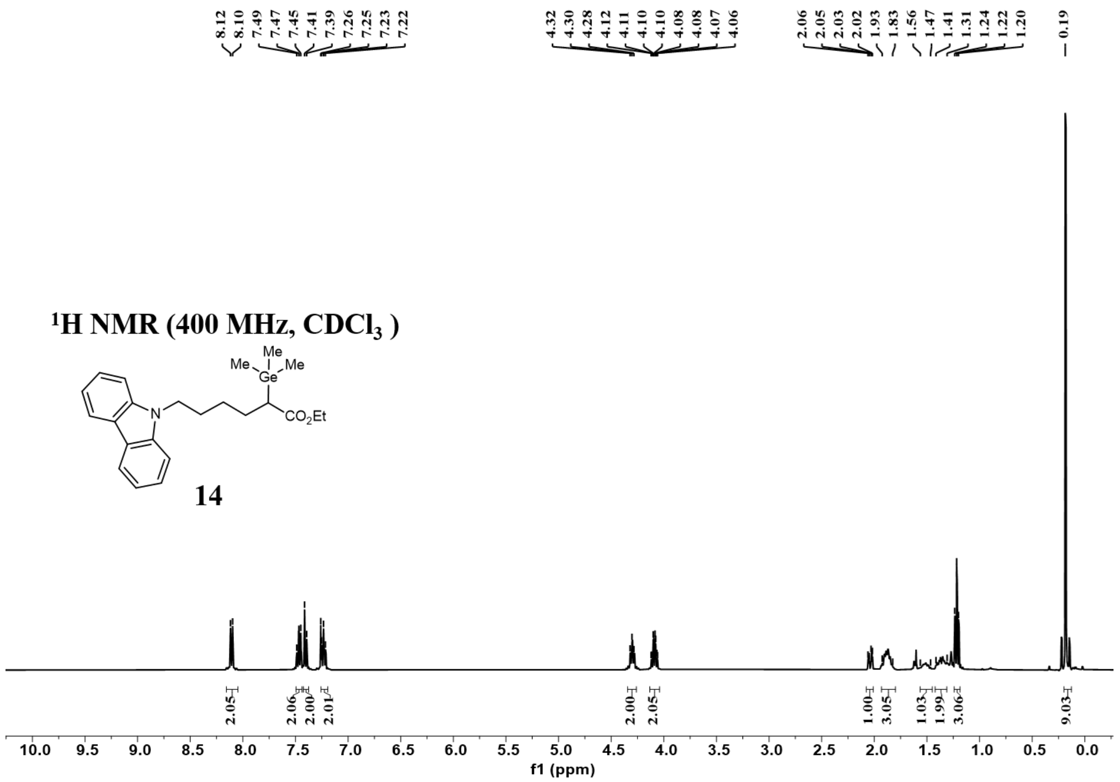

Supplementary Fig. 41. ^{1}H NMR of compound 14. The sample has been recorded in 400 MHz, $CDCl_3$ at 25 ºC

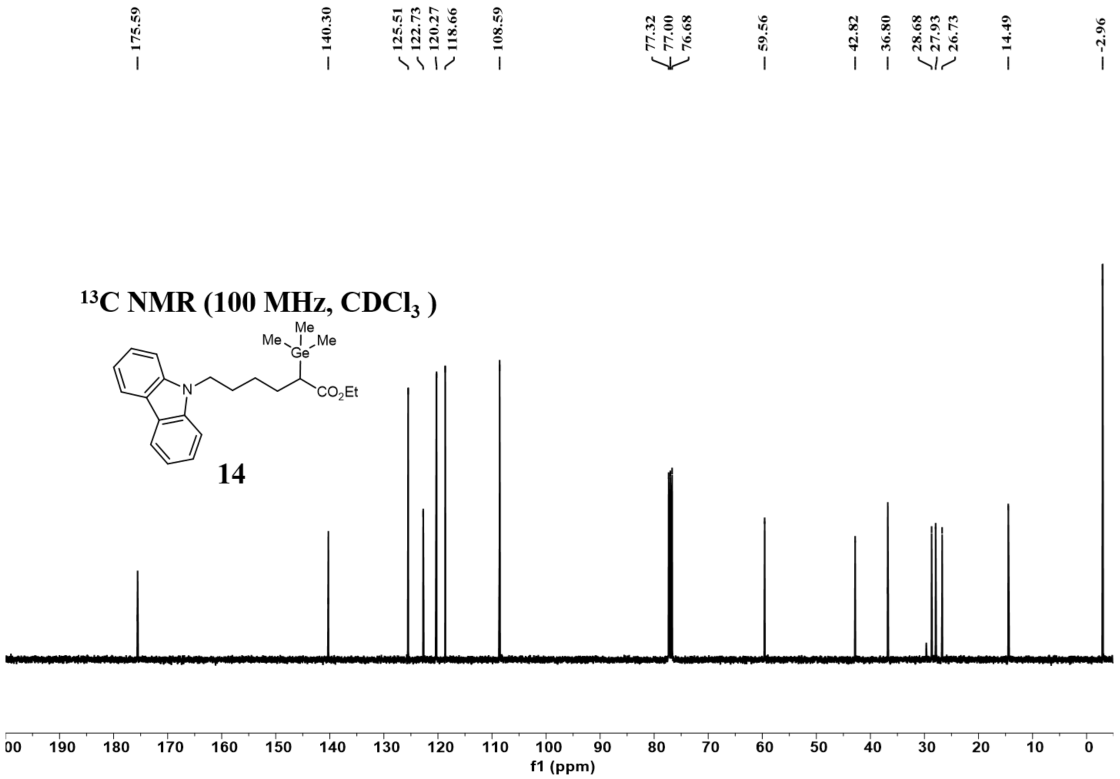

Supplementary Fig. 42. ^{13}C NMR of compound 14. The sample has been recorded in 100 MHz, $CDCl_3$ at 25 ºC

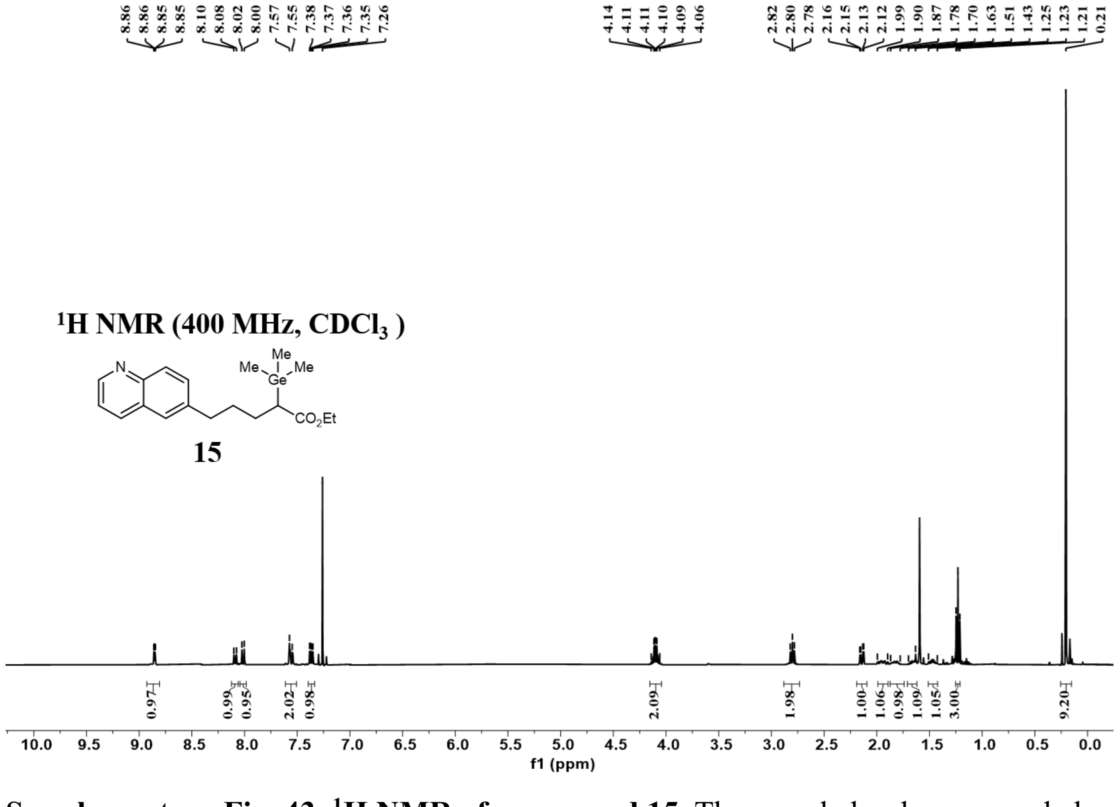

Supplementary Fig. 43. ^{1}H NMR of compound 15. The sample has been recorded in 400 MHz, $CDCl_3$ at 25 ºC

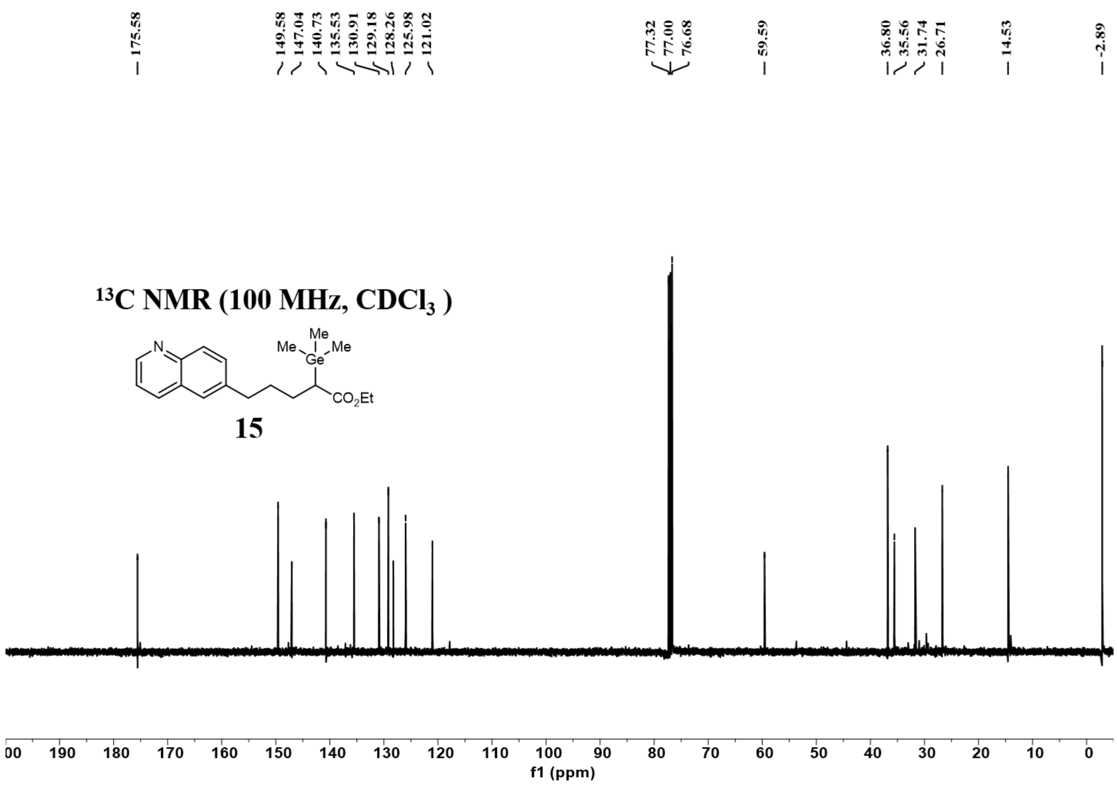

Supplementary Fig. 44. ^{13}C NMR of compound 15. The sample has been recorded in 100 MHz, $CDCl_3$ at 25 ºC

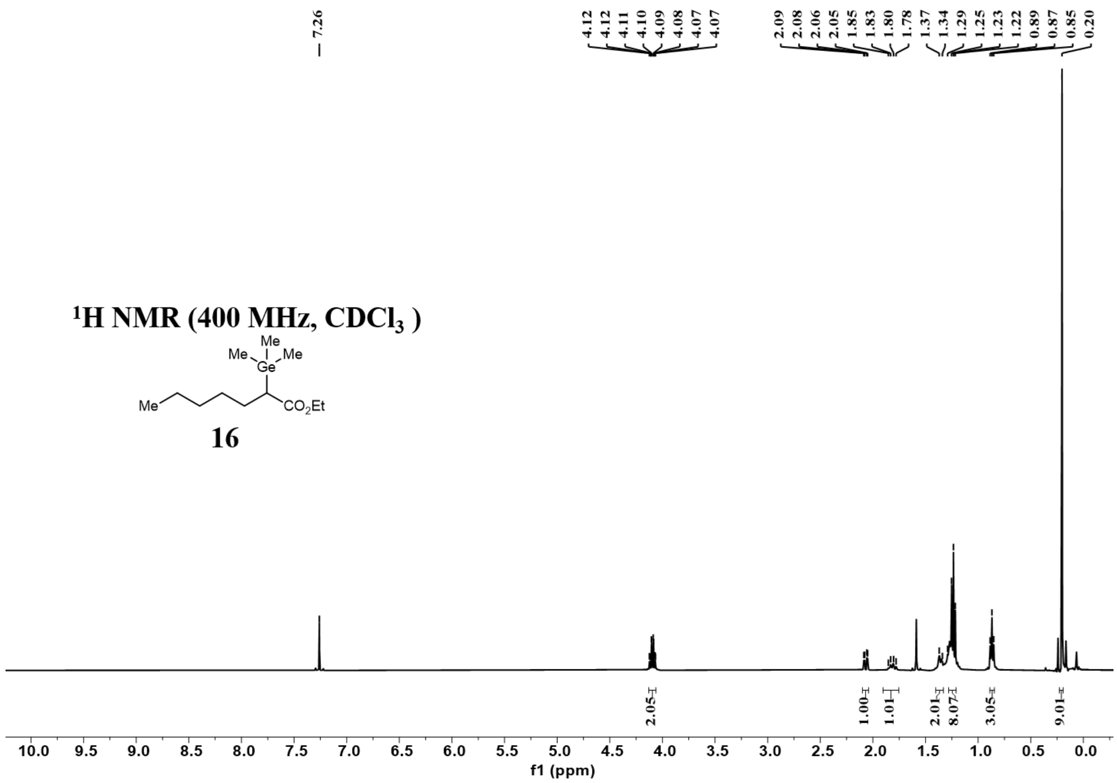

Supplementary Fig. 45. ^{1}H NMR of compound 16. The sample has been recorded in 400 MHz, $CDCl_3$ at 25 ºC

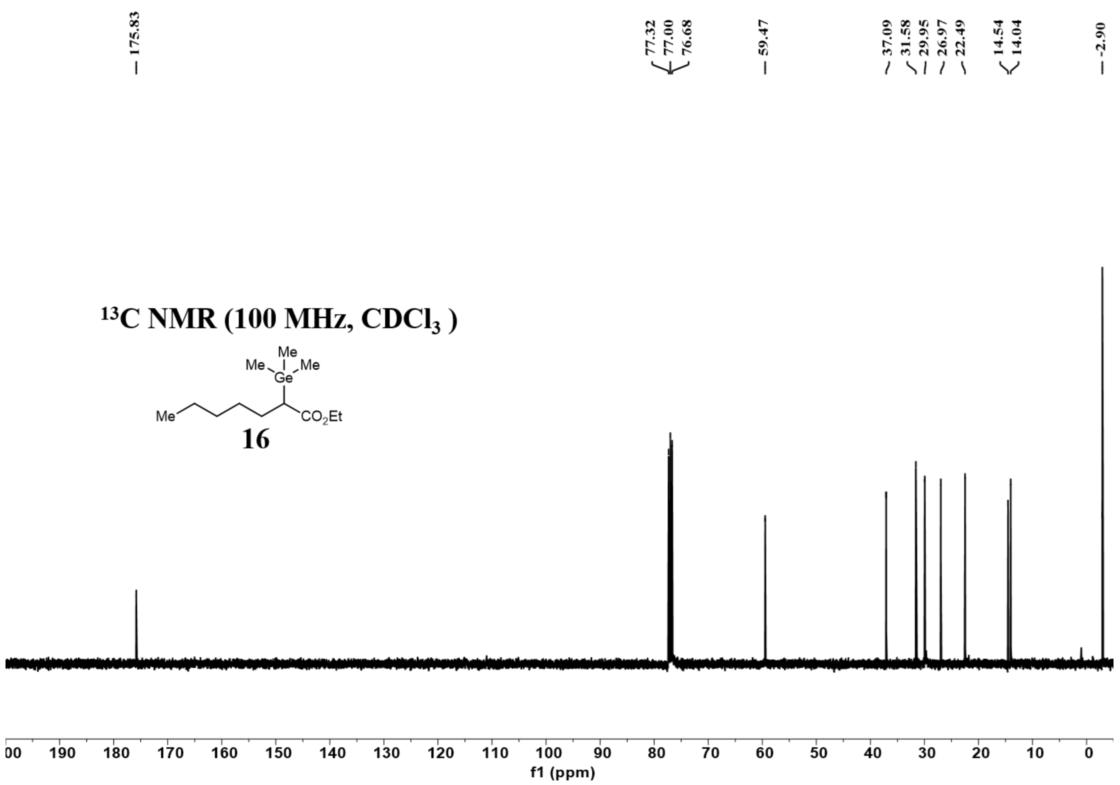

Supplementary Fig. 46. ^{13}C NMR of compound 16. The sample has been recorded in 100 MHz, $CDCl_3$ at 25 ºC

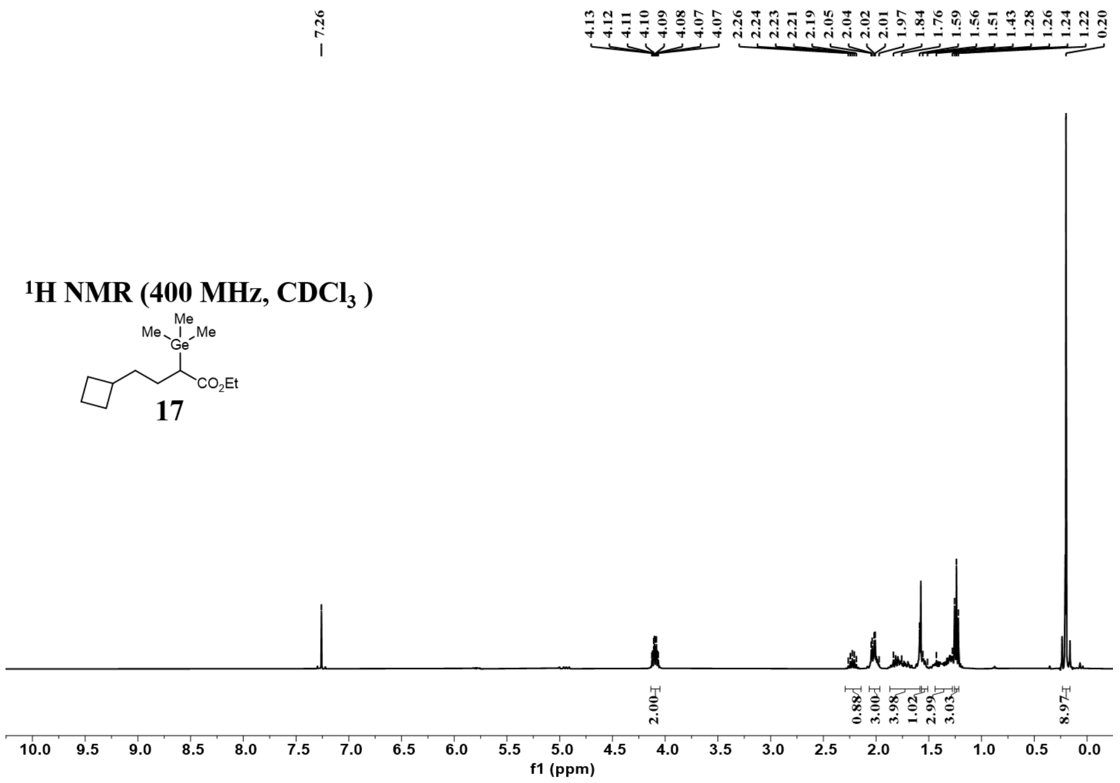

Supplementary Fig. 47. ^{1}H NMR of compound 17. The sample has been recorded in 400 MHz, $CDCl_3$ at 25 ºC

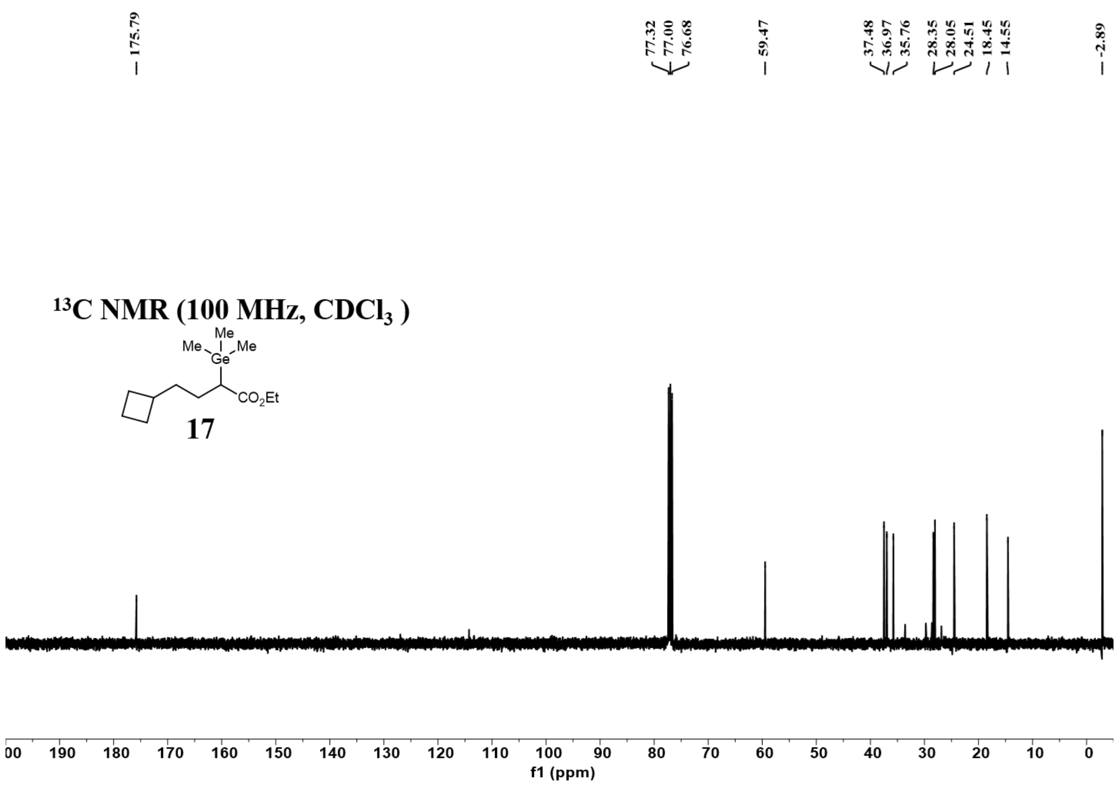

Supplementary Fig. 48. ^{13}C NMR of compound 17. The sample has been recorded in 100 MHz, $CDCl_3$ at 25 ºC

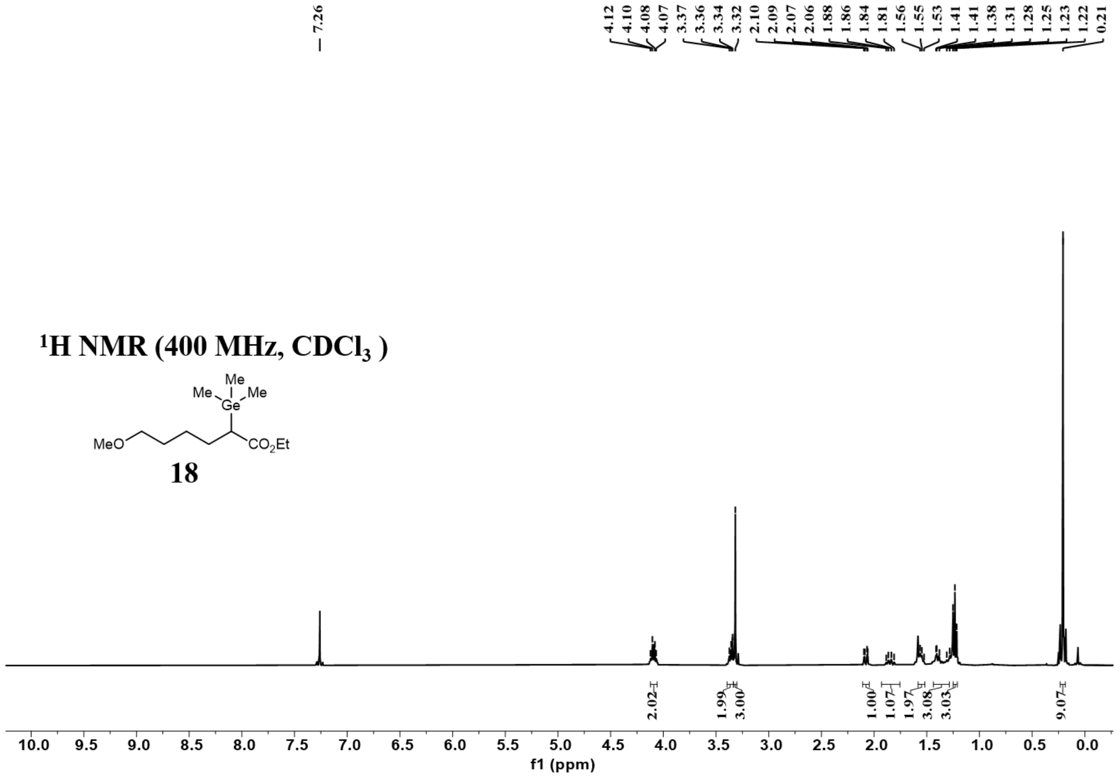

Supplementary Fig. 49. ^{1}H NMR of compound 18. The sample has been recorded in 400 MHz, $CDCl_3$ at 25 ºC

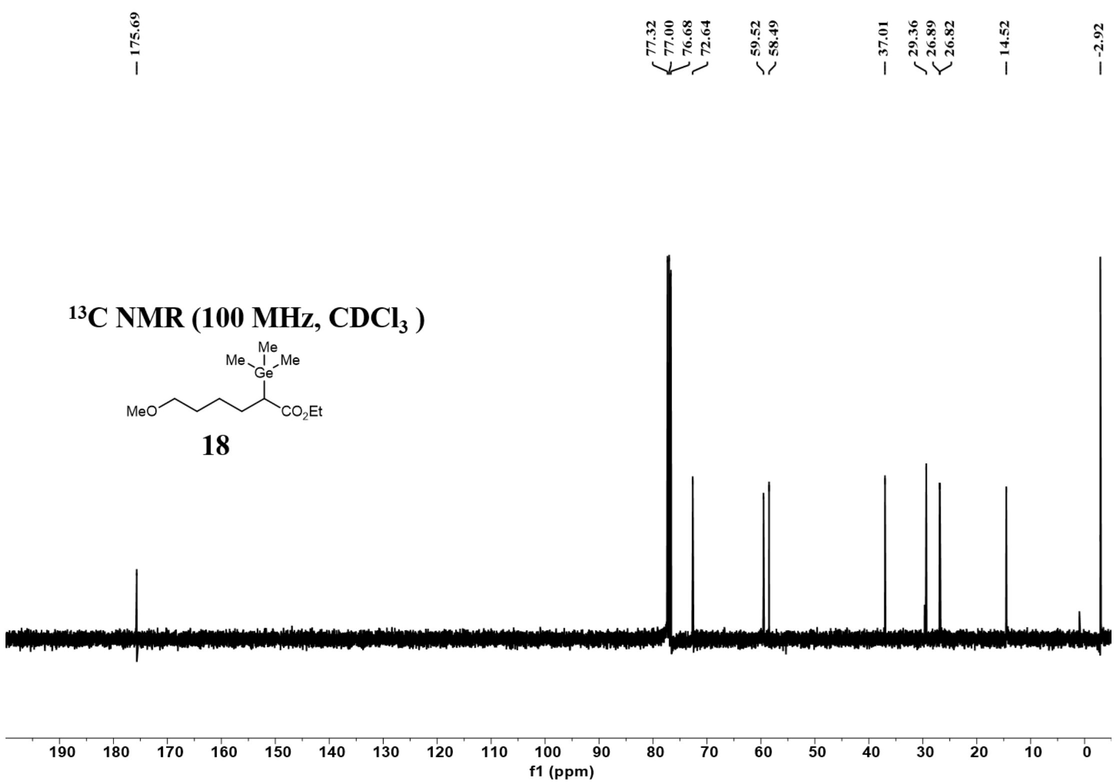

Supplementary Fig. 50. ^{13}C NMR of compound 18. The sample has been recorded in 100 MHz, $CDCl_3$ at 25 ºC

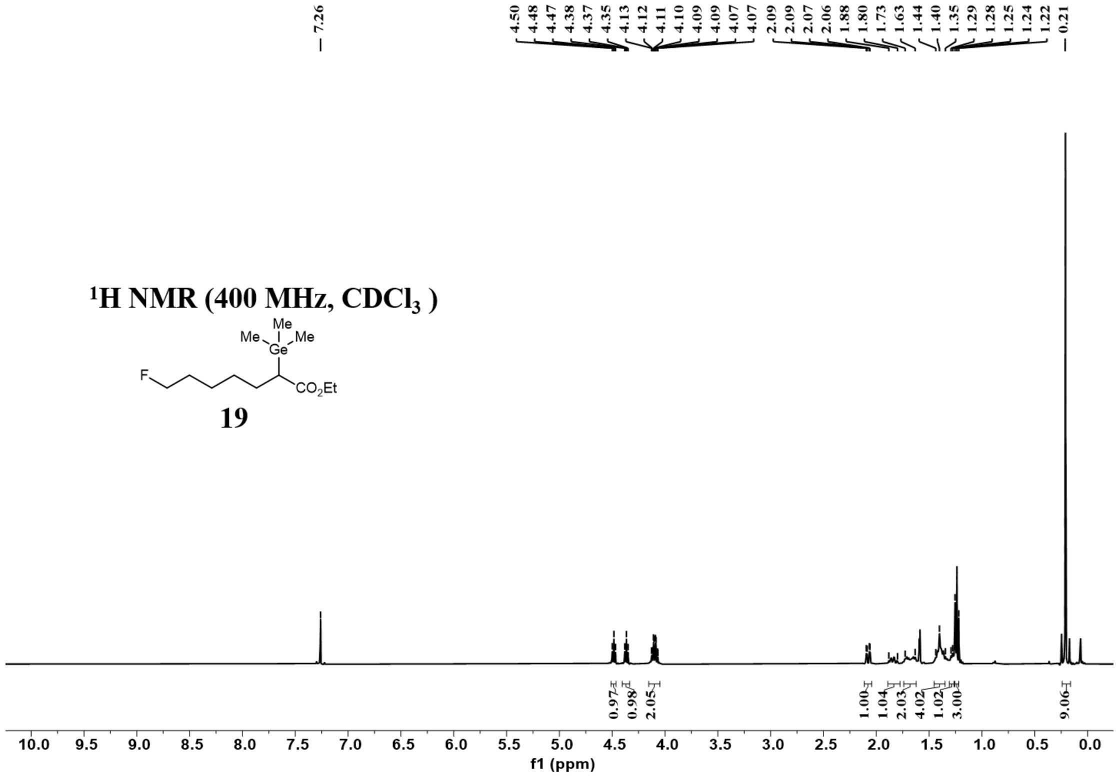

Supplementary Fig. 51. ^{1}H NMR of compound 19. The sample has been recorded in 400 MHz, $CDCl_3$ at 25 ºC

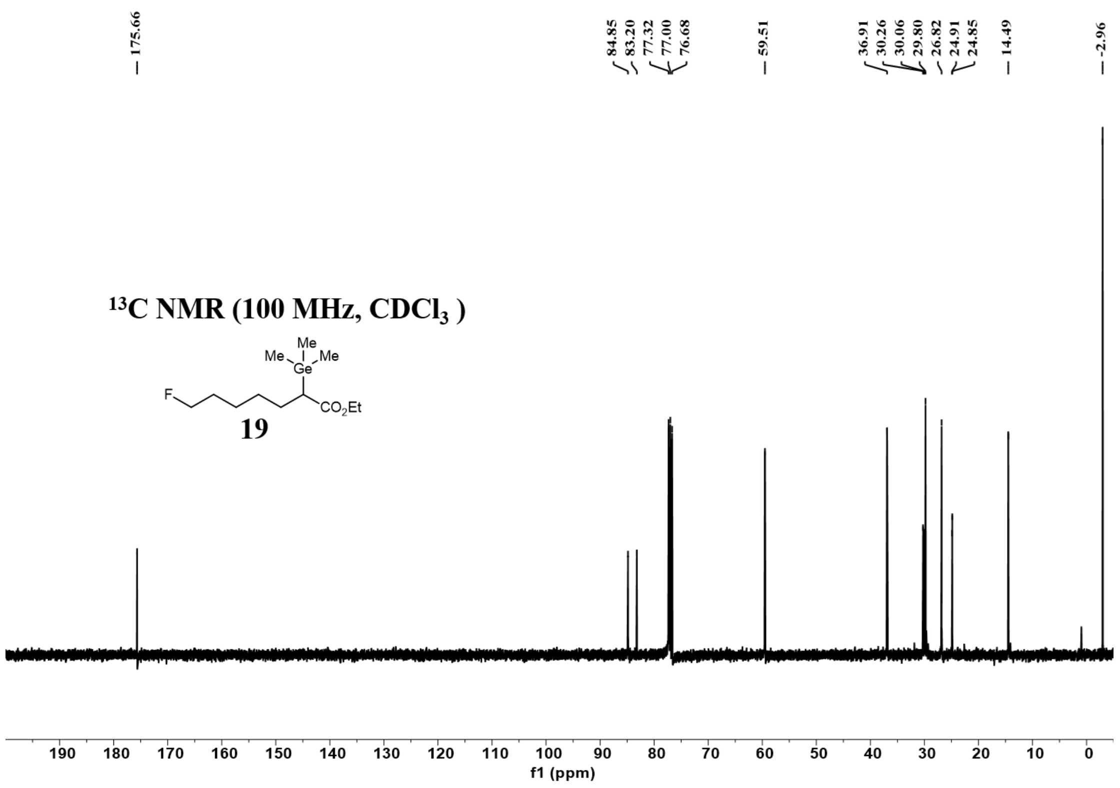

Supplementary Fig. 52. ^{13}C NMR of compound 19. The sample has been recorded in 100 MHz, CDCl3 at 25 ºC

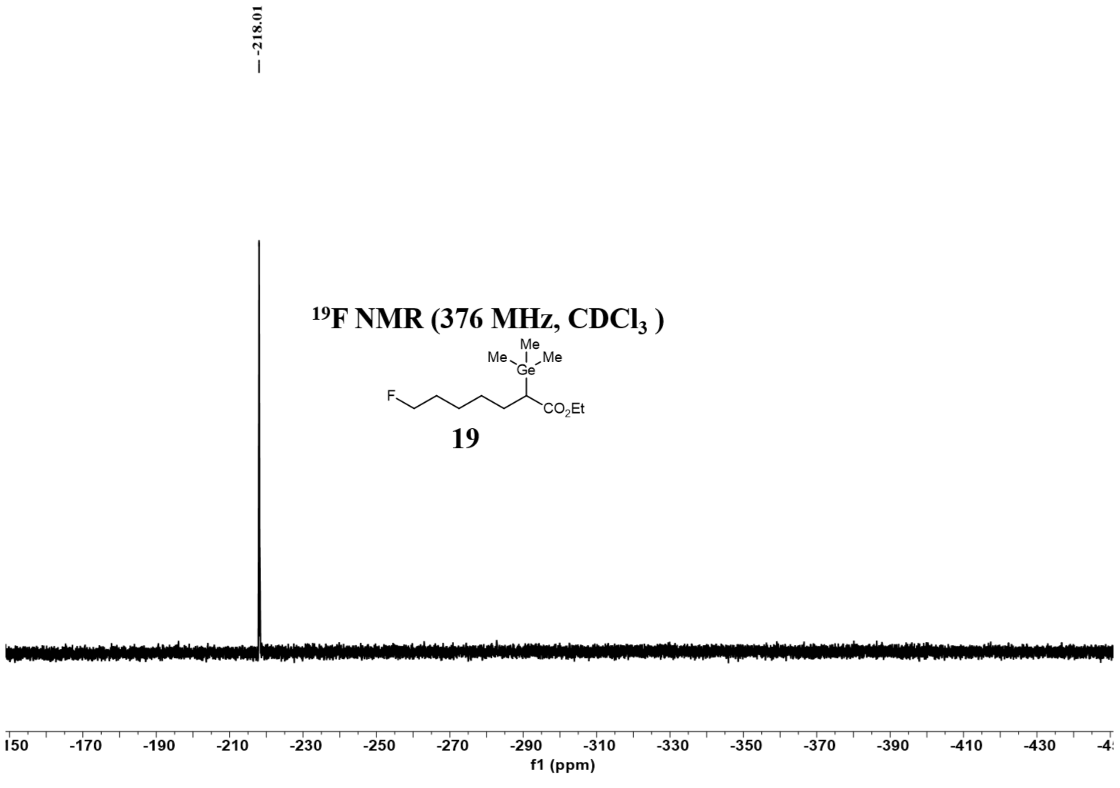

Supplementary Fig. 53. ^{19}F NMR of compound 19. The sample has been recorded in 376 MHz, CDCl3 at 25 ºC

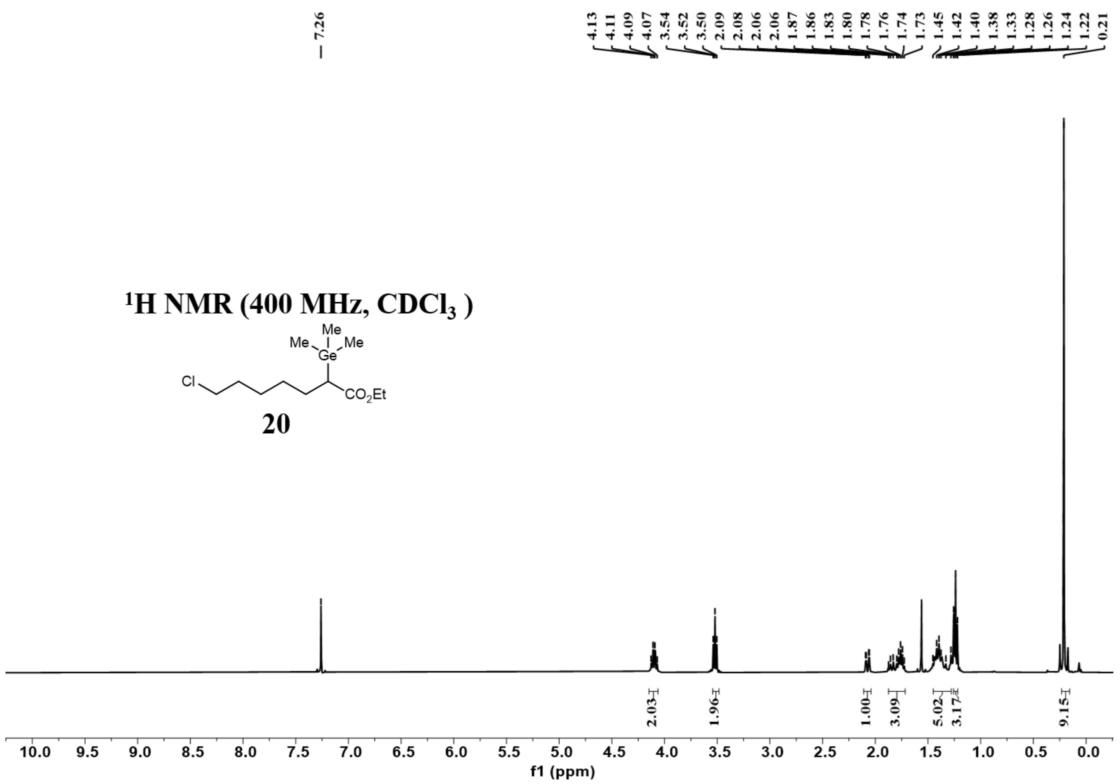

Supplementary Fig. 54. ^{1}H NMR of compound 20. The sample has been recorded in 400 MHz, $CDCl_3$ at 25 ºC

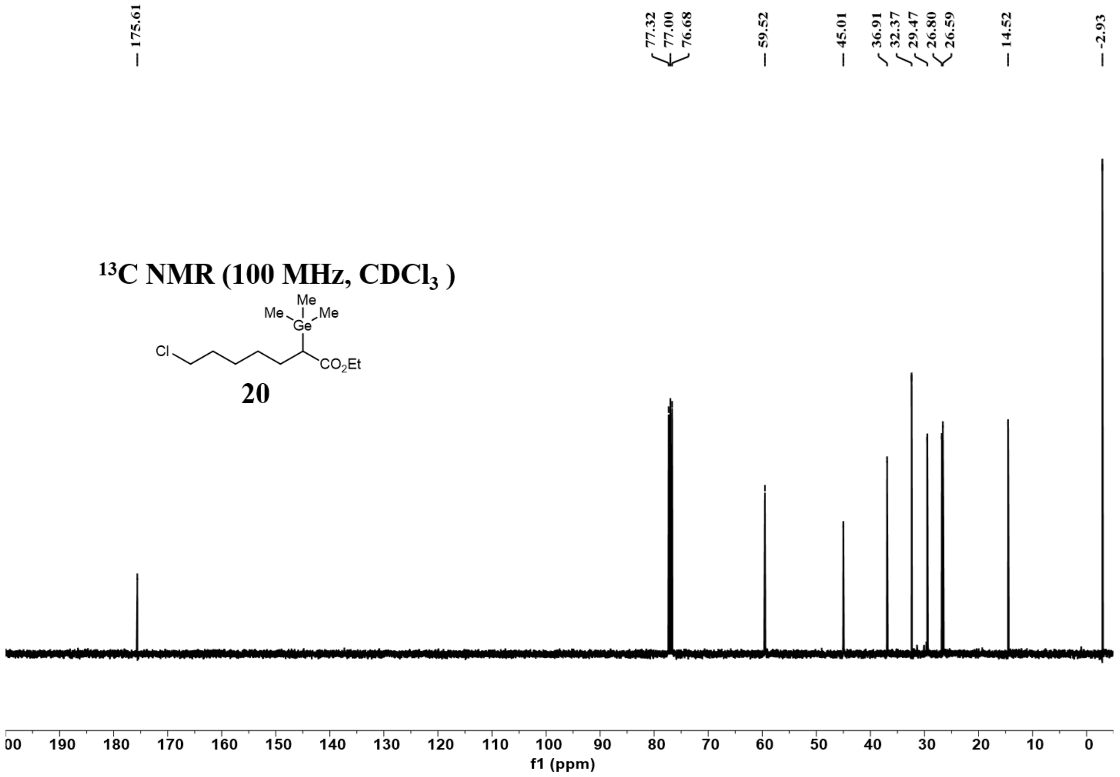

Supplementary Fig. 55. ^{13}C NMR of compound 20. The sample has been recorded in 100 MHz, $CDCl_3$ at 25 ºC

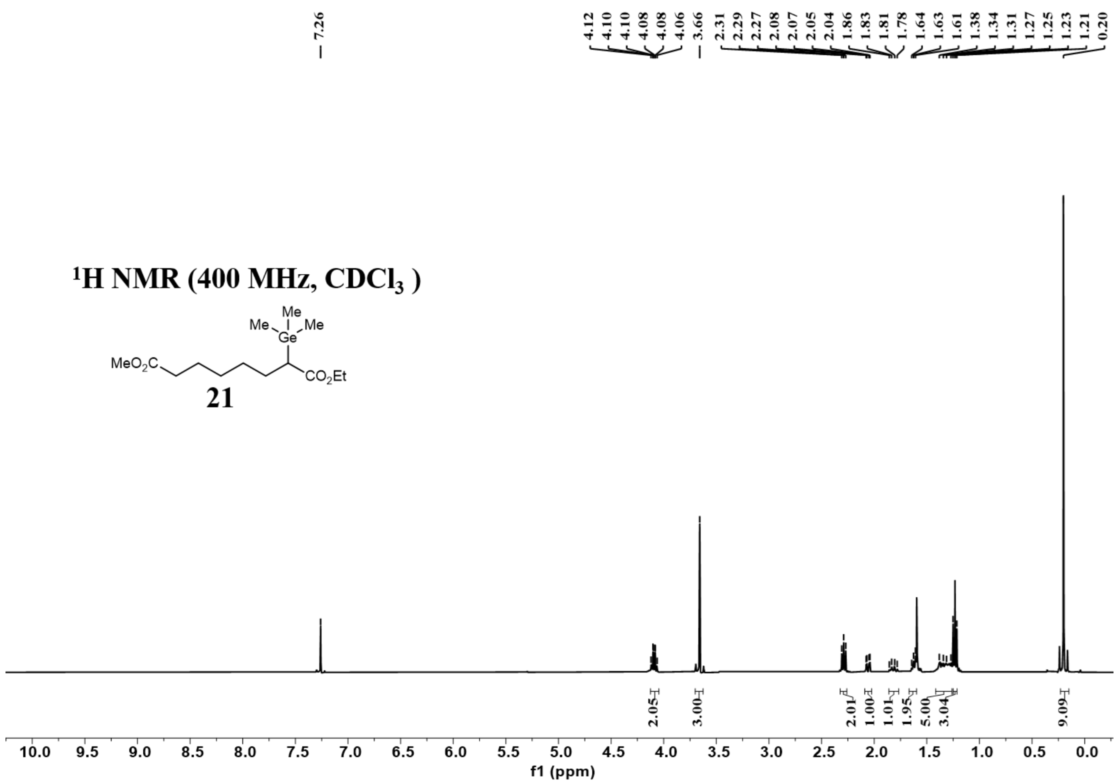

Supplementary Fig. 56. ^{1}H NMR of compound 21. The sample has been recorded in 400 MHz, $CDCl_3$ at 25 ºC

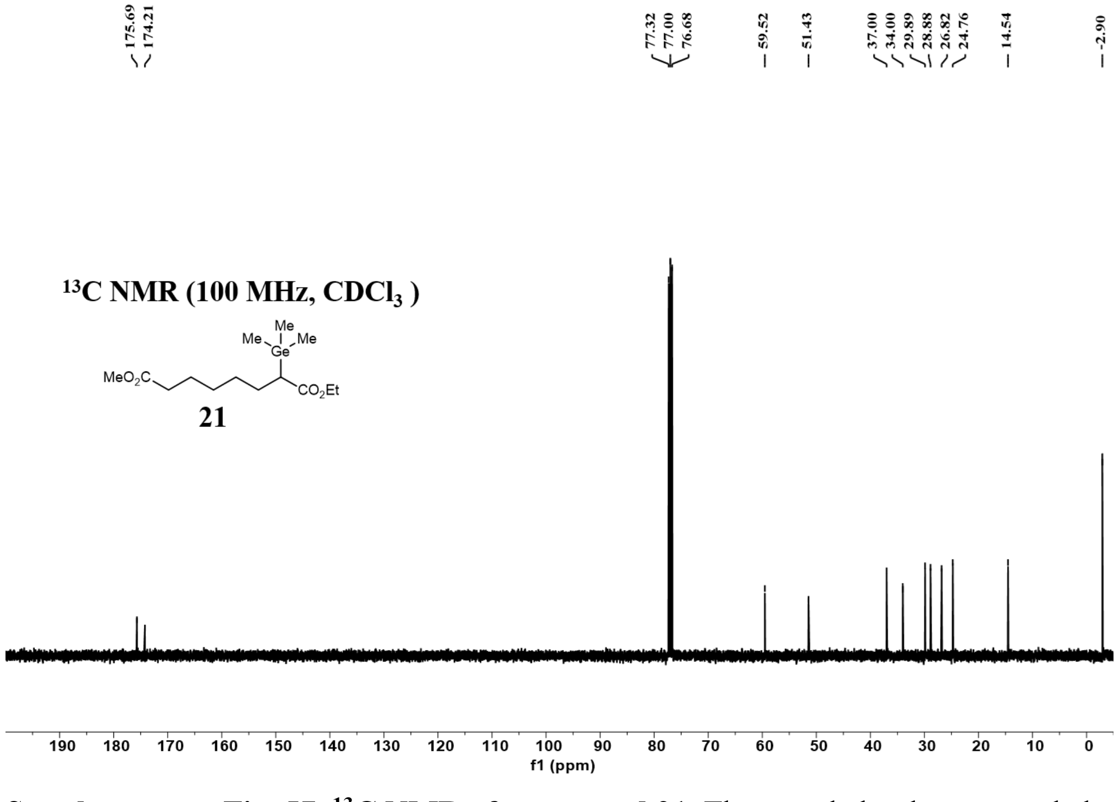

Supplementary Fig. 57. ^{13}C NMR of compound 21. The sample has been recorded in 100 MHz, $CDCl_3$ at 25 ºC

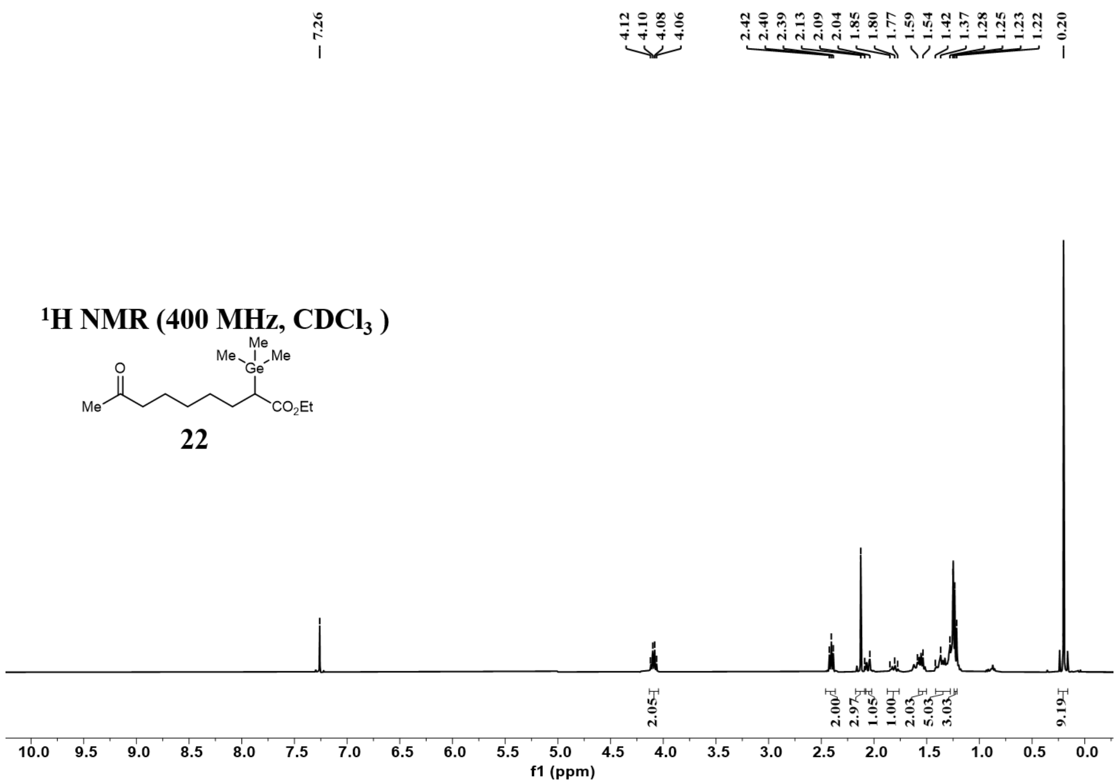

Supplementary Fig. 58. ^{1}H NMR of compound 22. The sample has been recorded in 400 MHz, $CDCl_3$ at 25 ºC

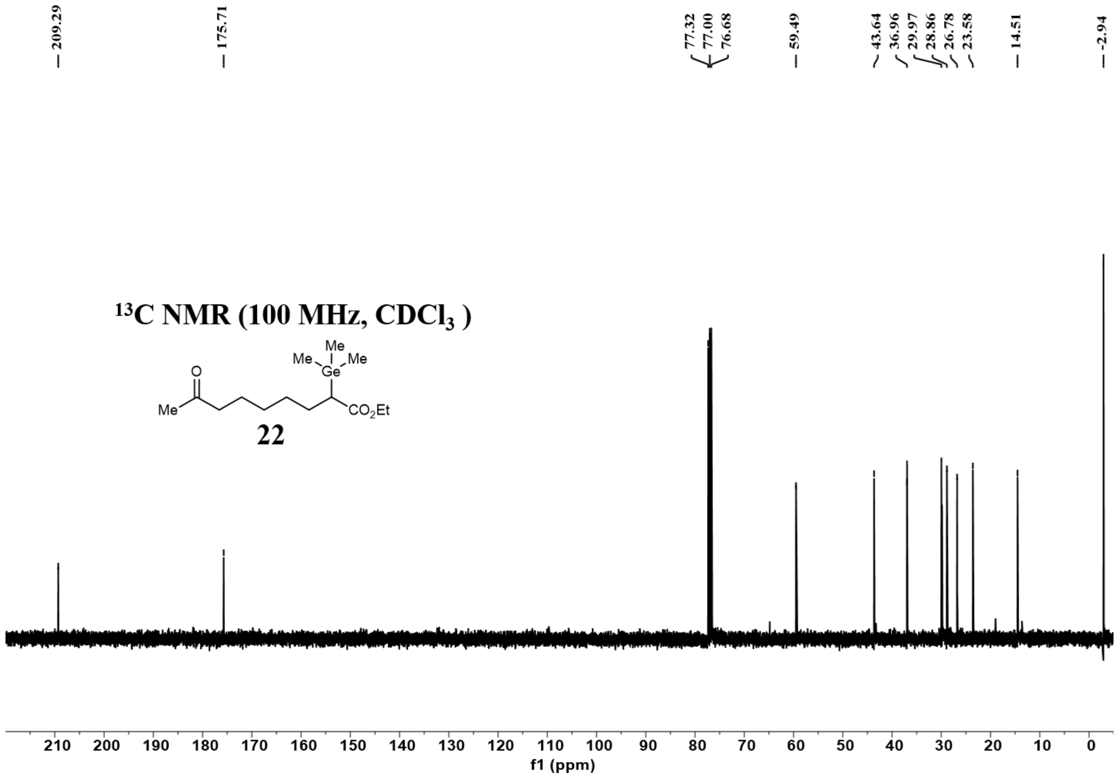

Supplementary Fig. 59. ^{13}C NMR of compound 22. The sample has been recorded in 100 MHz, $CDCl_3$ at 25 ºC

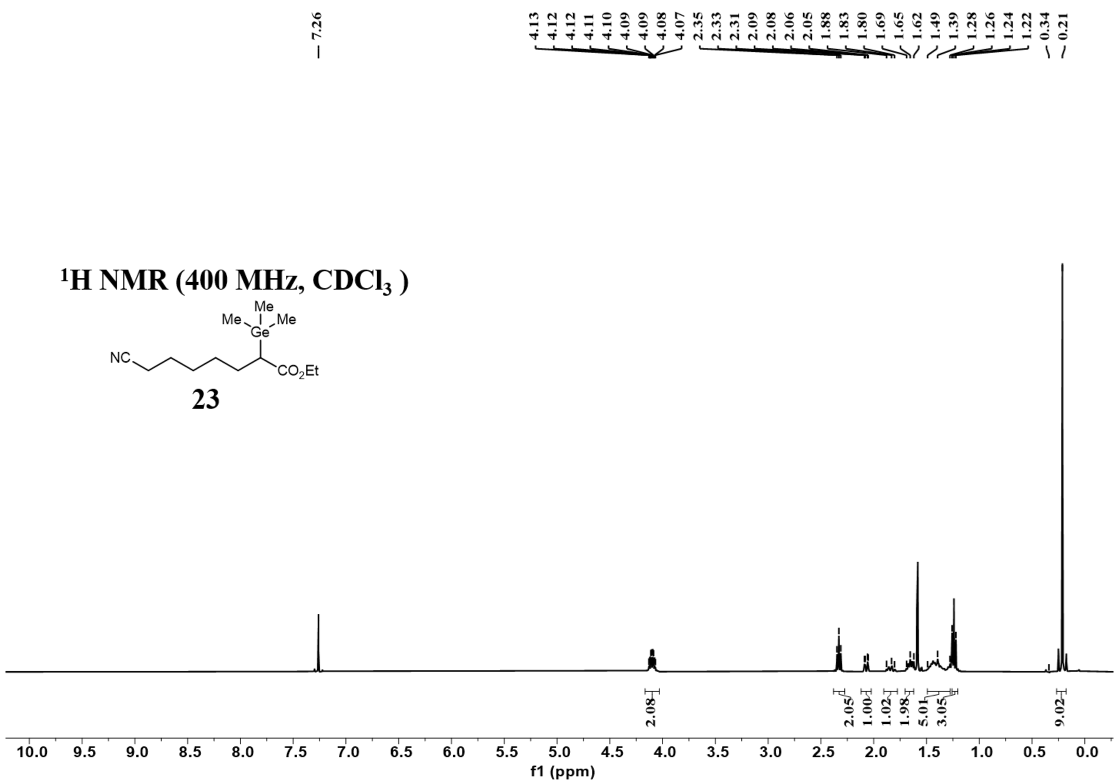

Supplementary Fig. 60. ^{1}H NMR of compound 23. The sample has been recorded in 400 MHz, $CDCl_3$ at 25 ºC

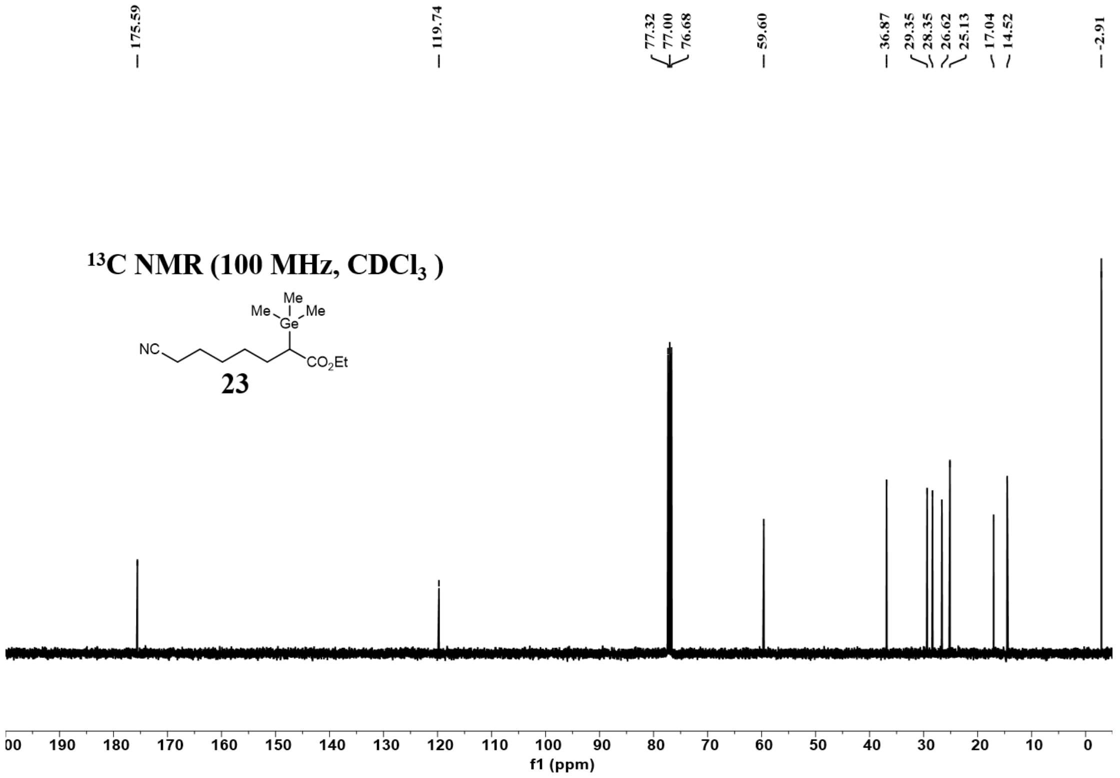

Supplementary Fig. 61. ^{13}C NMR of compound 23. The sample has been recorded in 100 MHz, $CDCl_3$ at 25 ºC

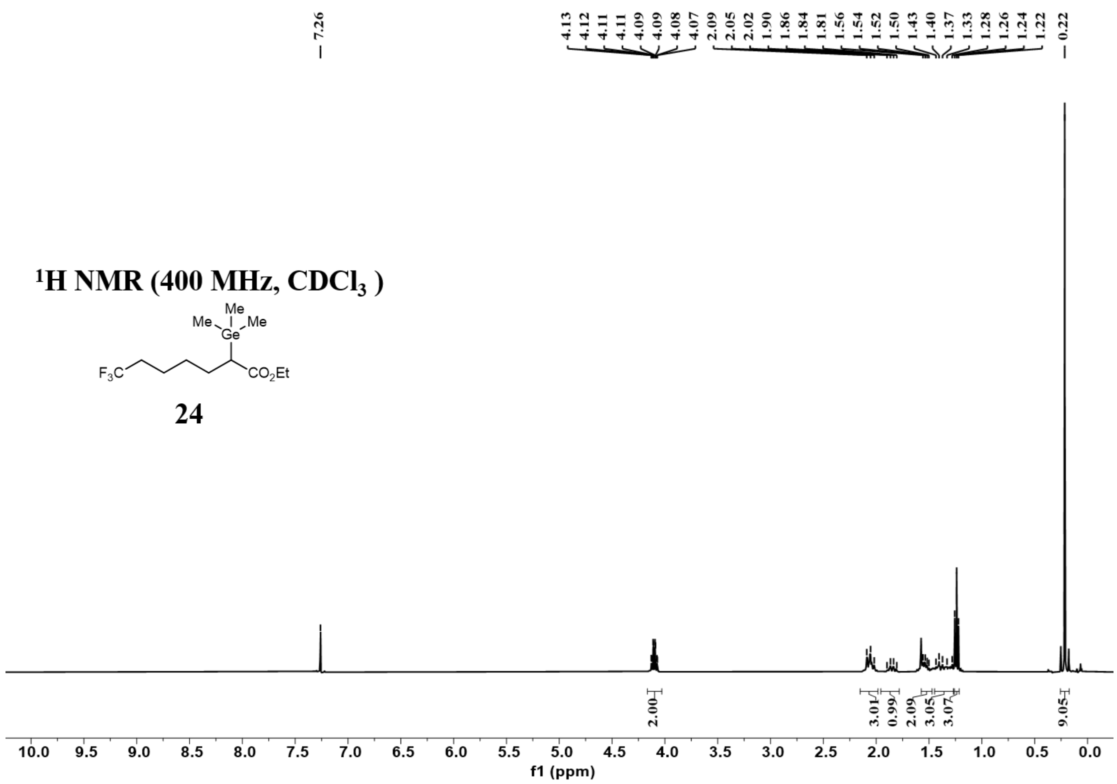

Supplementary Fig. 62. ^{1}H NMR of compound 24. The sample has been recorded in 400 MHz, $CDCl_3$ at 25 ºC

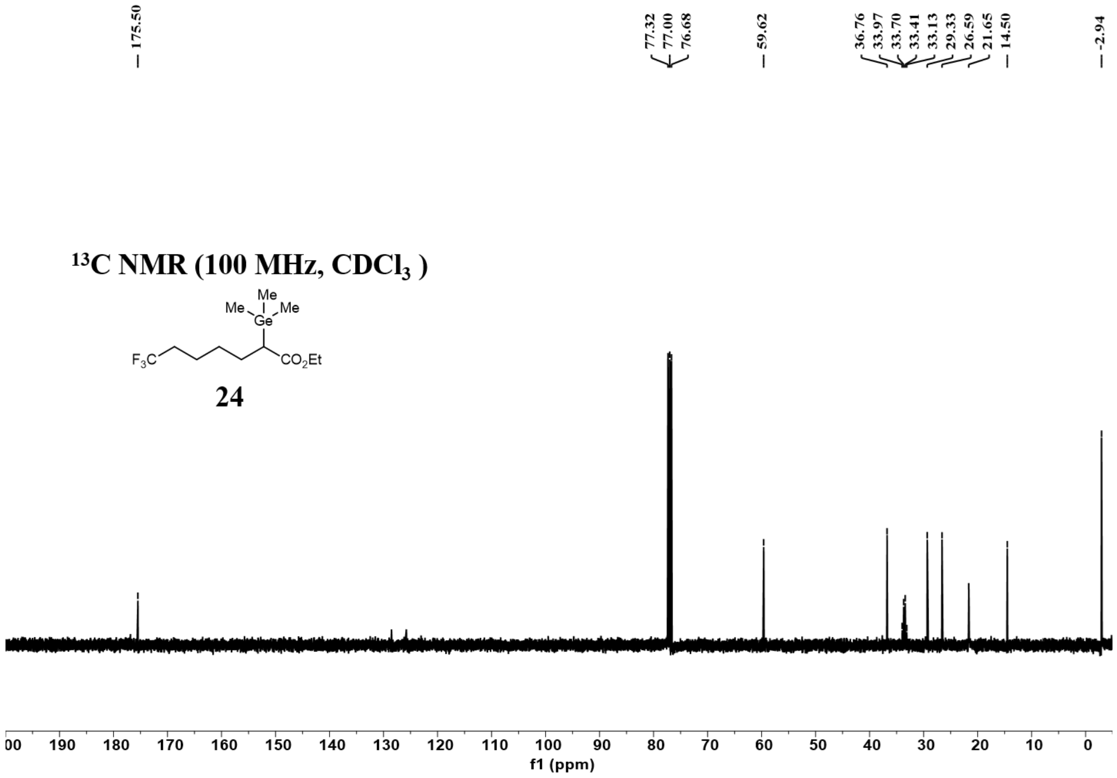

Supplementary Fig. 63. ^{13}C NMR of compound 24. The sample has been recorded in 100 MHz, $CDCl_3$ at 25 ºC

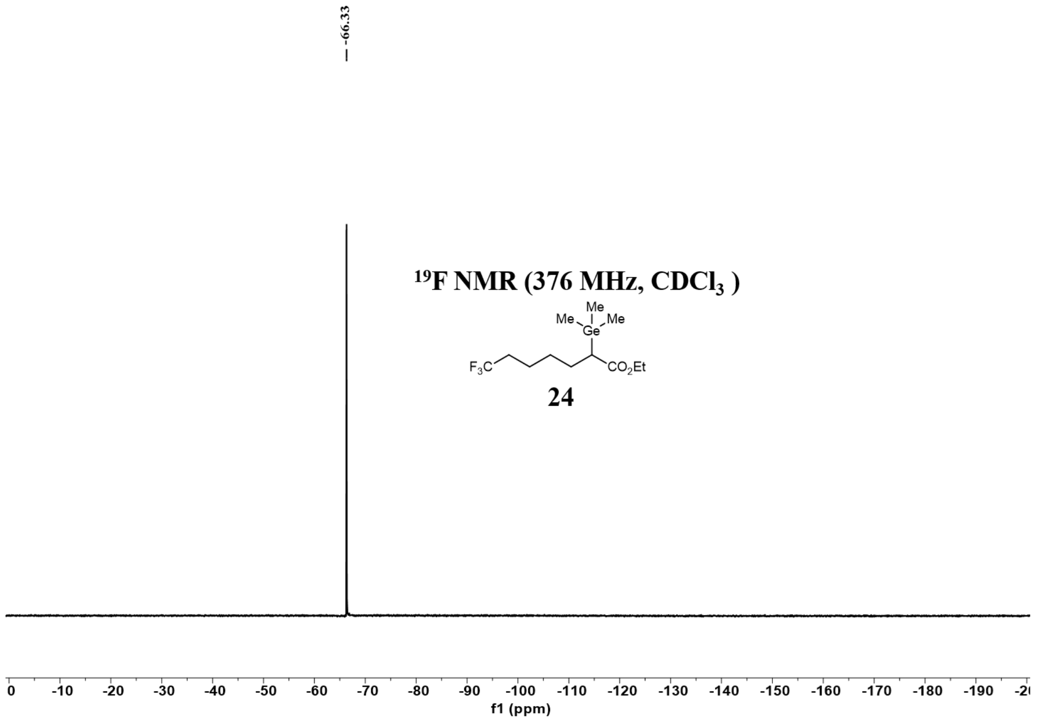

Supplementary Fig. 64. **^{19}F NMR of compound 24.** The sample has been recorded in 376 MHz, $CDCl_3$ at 25 ºC

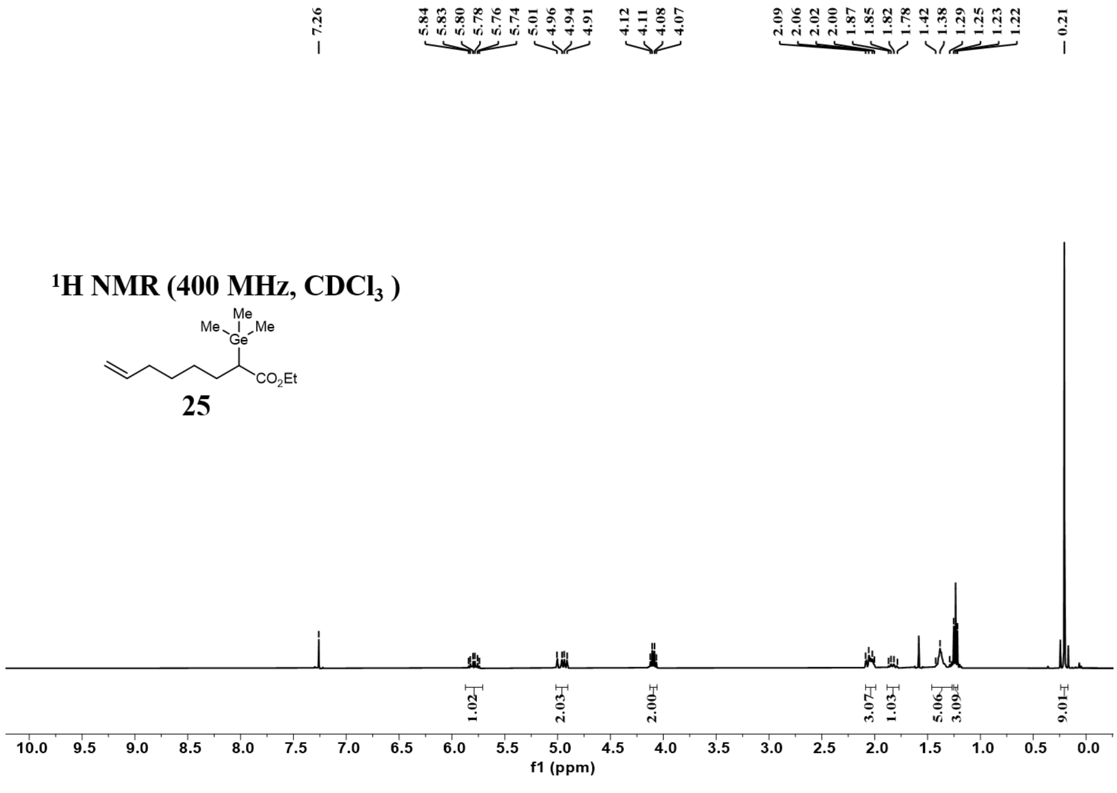

Supplementary Fig. 65. ^{1}H NMR of compound 25. The sample has been recorded in 400 MHz, $CDCl_3$ at 25 ºC

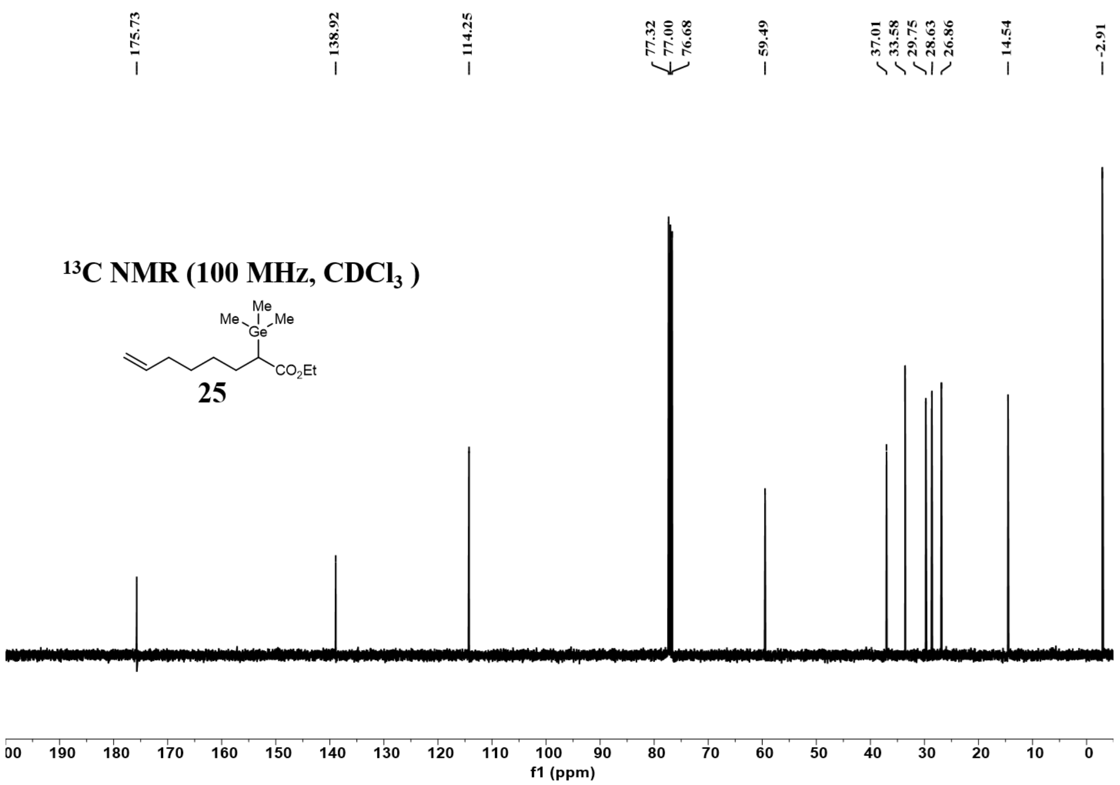

Supplementary Fig. 66. ^{13}C NMR of compound 25. The sample has been recorded in 100 MHz, $CDCl_3$ at 25 ºC.

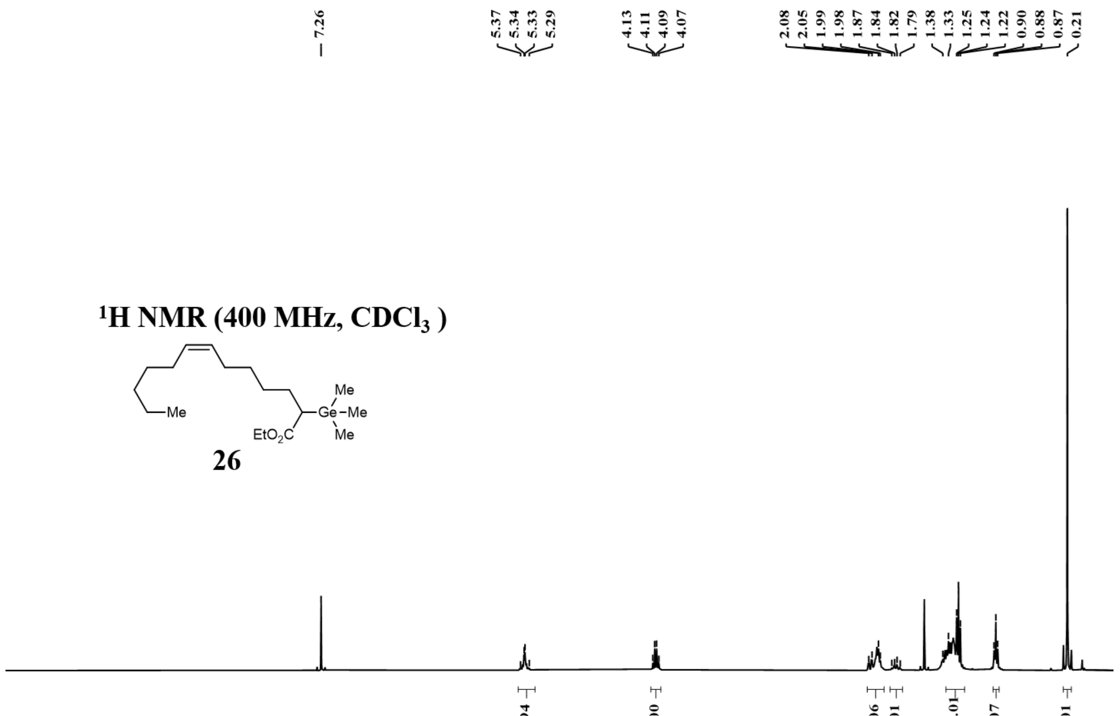

Supplementary Fig. 67. ^{1}H NMR of compound 26. The sample has been recorded in 400 MHz, $CDCl_3$ at 25 ºC

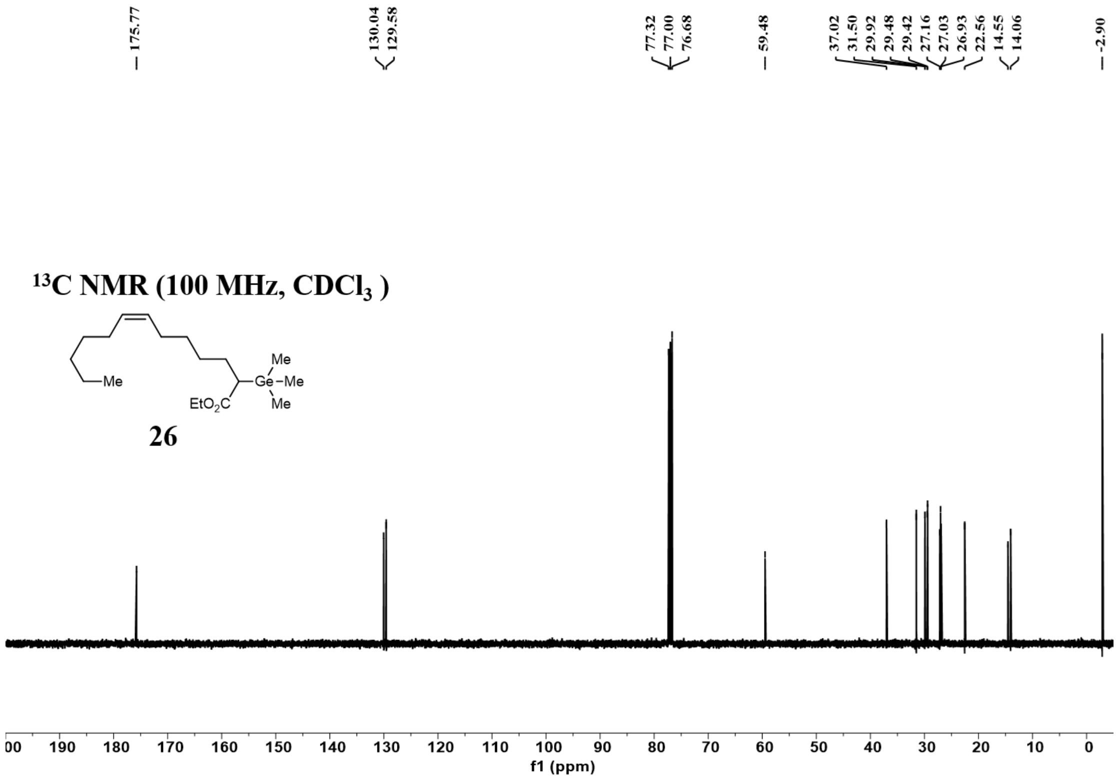

Supplementary Fig. 68. ^{13}C NMR of compound 26. The sample has been recorded in 100 MHz, $CDCl_3$ at 25 ºC.

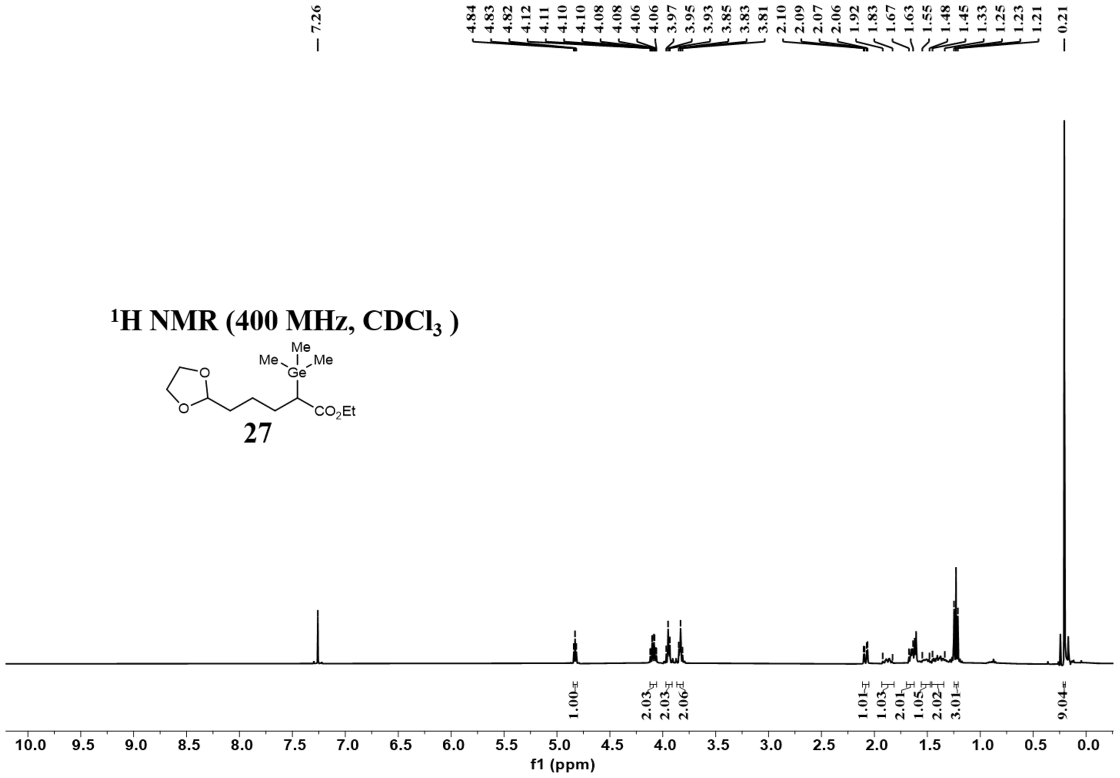

Supplementary Fig. 69. ^{1}H NMR of compound 27. The sample has been recorded in 400 MHz, $CDCl_3$ at 25 ºC

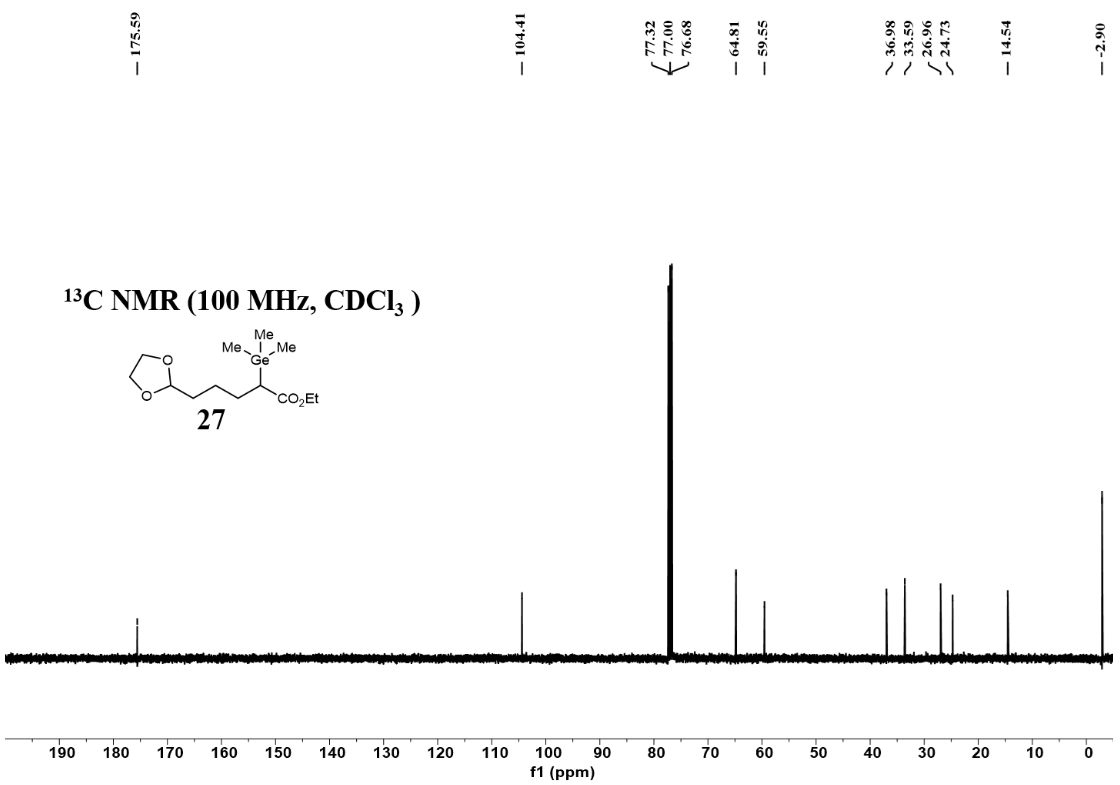

Supplementary Fig. 70. ^{13}C NMR of compound 27. The sample has been recorded in 100 MHz, $CDCl_3$ at 25 ºC.

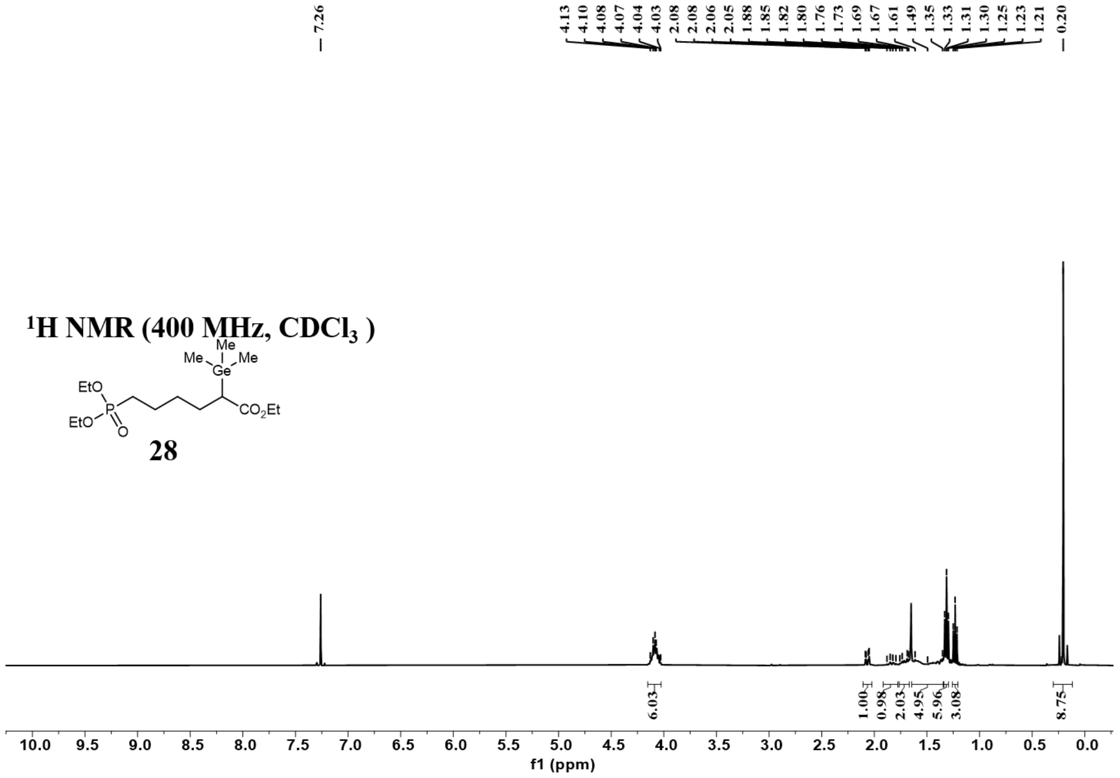

Supplementary Fig. 71. ^{1}H NMR of compound 28. The sample has been recorded in 400 MHz, $CDCl_3$ at 25 ºC

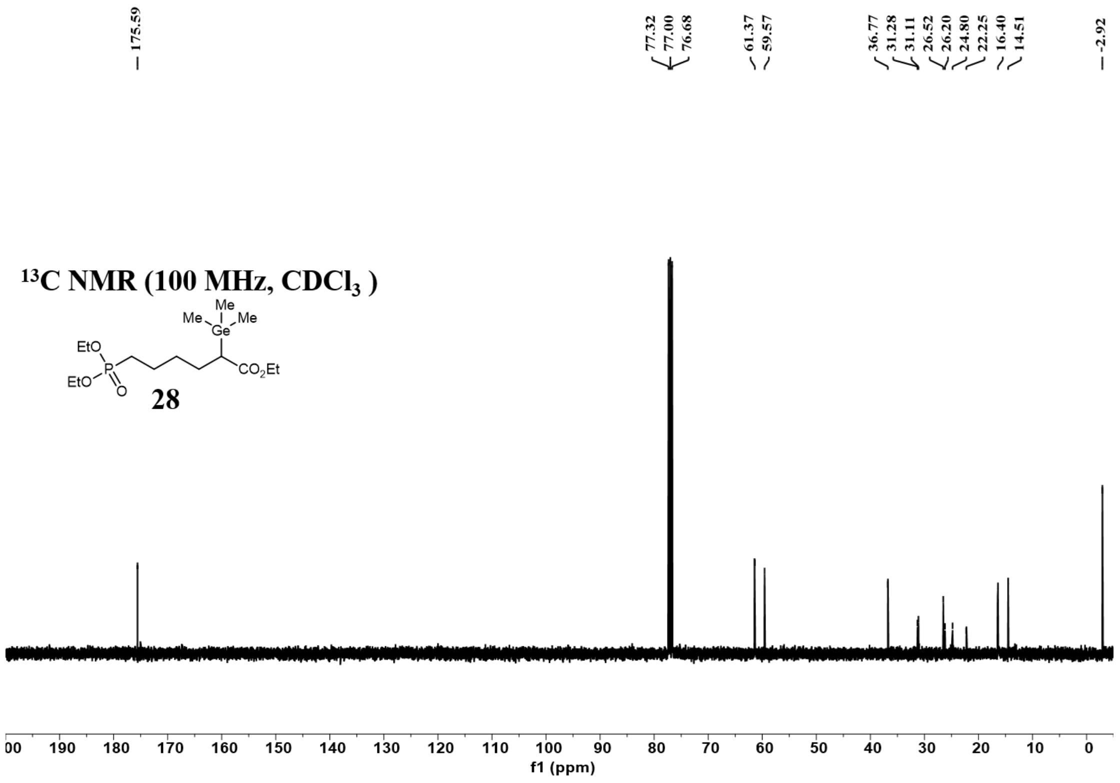

Supplementary Fig. 72. ^{13}C NMR of compound 28. The sample has been recorded in 100 MHz, $CDCl_3$ at 25 ºC.

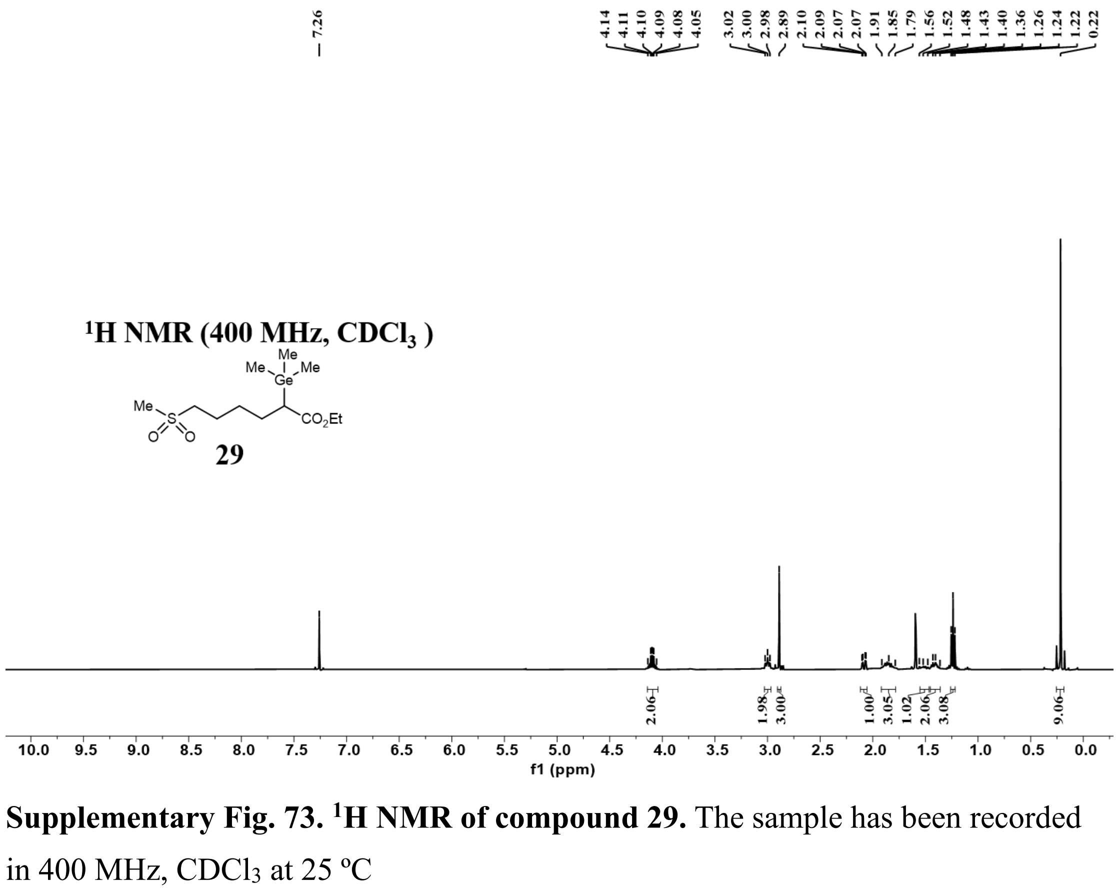

Supplementary Fig. 73. ^{1}H NMR of compound 29. The sample has been recorded in 400 MHz, $CDCl_3$ at 25 ºC

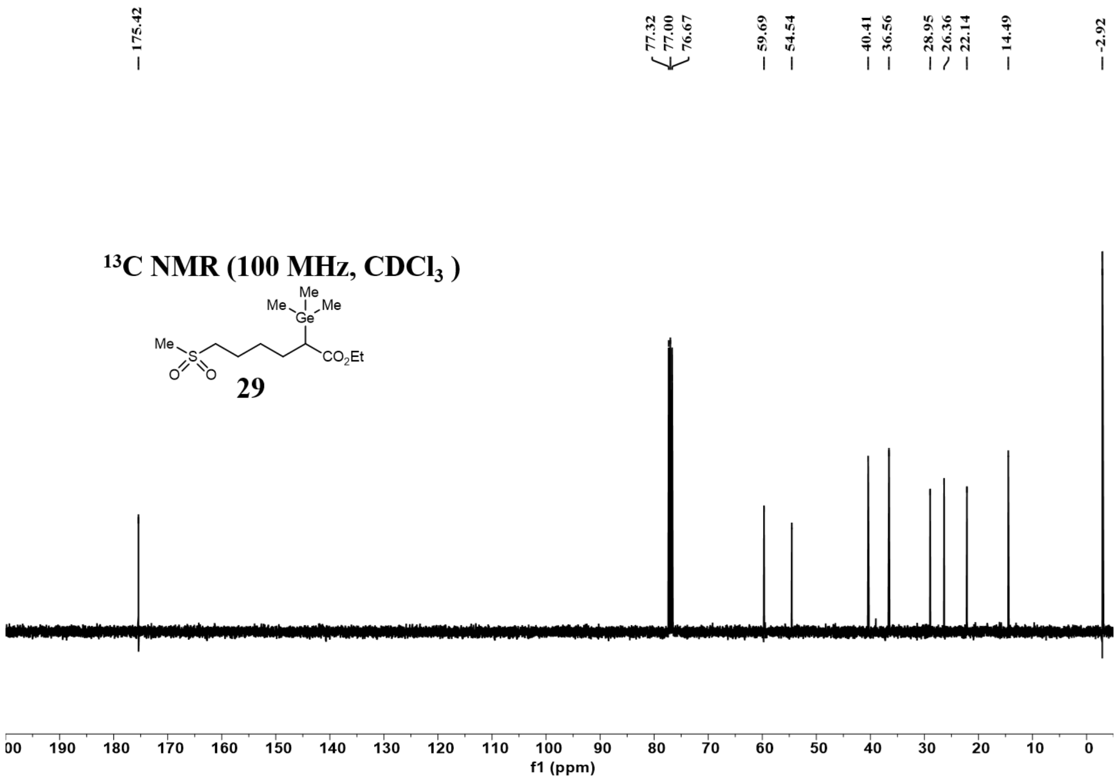

Supplementary Fig. 74. ^{13}C NMR of compound 29. The sample has been recorded in 100 MHz, $CDCl_3$ at 25 ºC

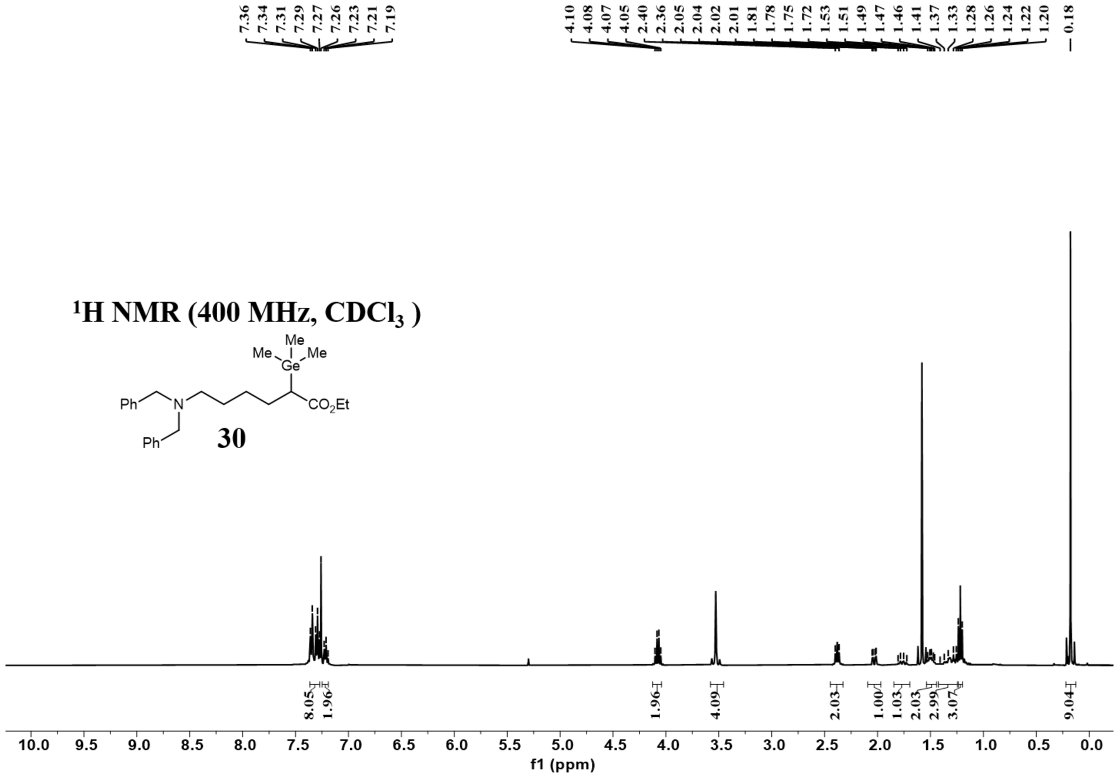

Supplementary Fig. 75. ^{1}H NMR of compound 30. The sample has been recorded in 400 MHz, $CDCl_3$ at 25 ºC

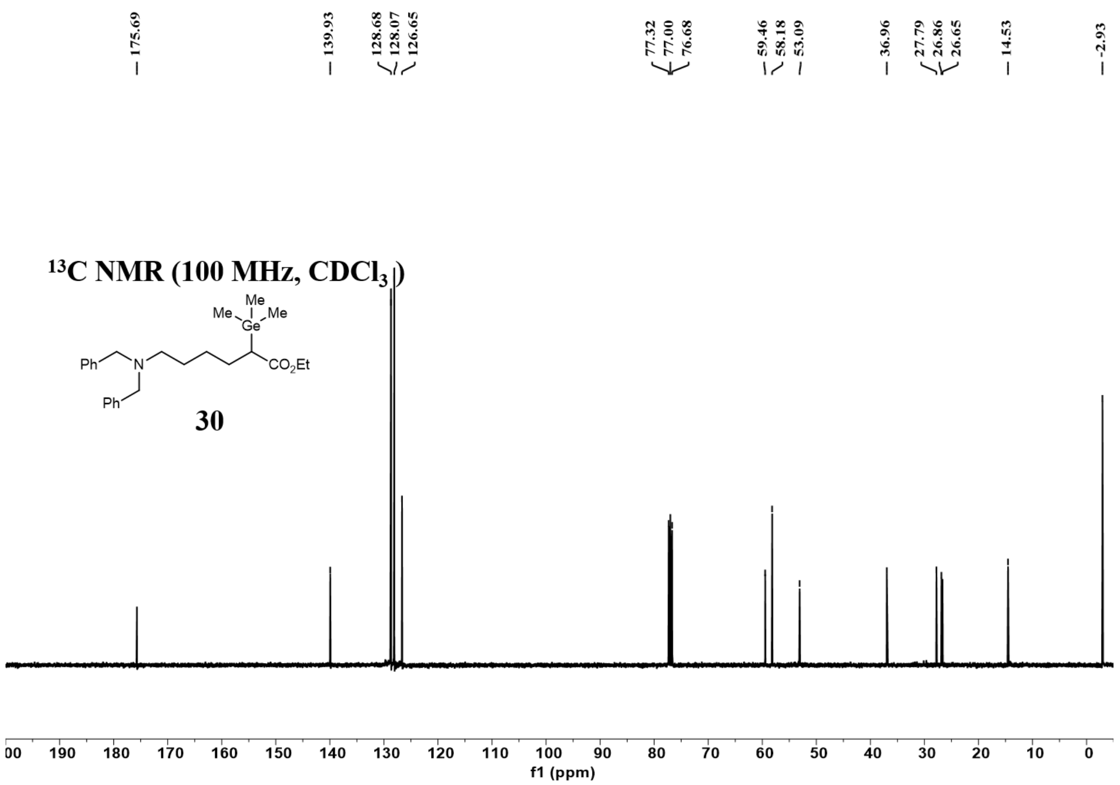

Supplementary Fig. 76. ^{13}C NMR of compound 30. The sample has been recorded in 100 MHz, $CDCl_3$ at 25 ºC

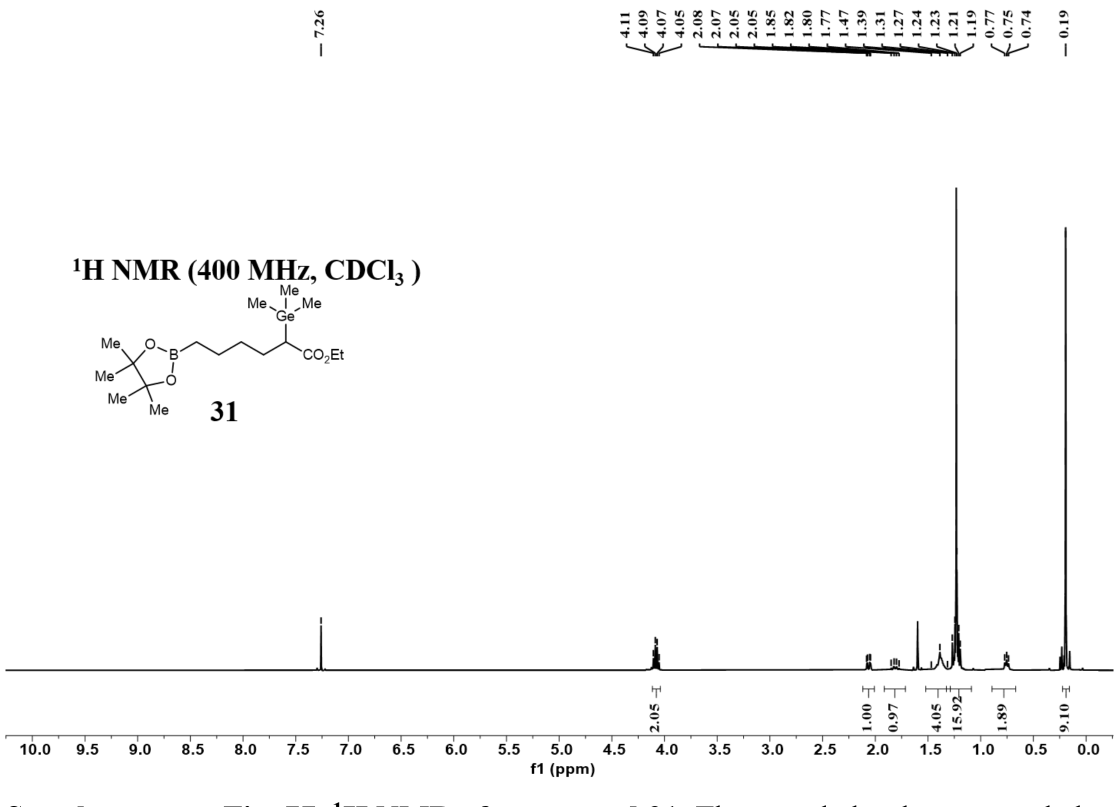

Supplementary Fig. 77. ^{1}H NMR of compound 31. The sample has been recorded in 400 MHz, $CDCl_3$ at 25 ºC

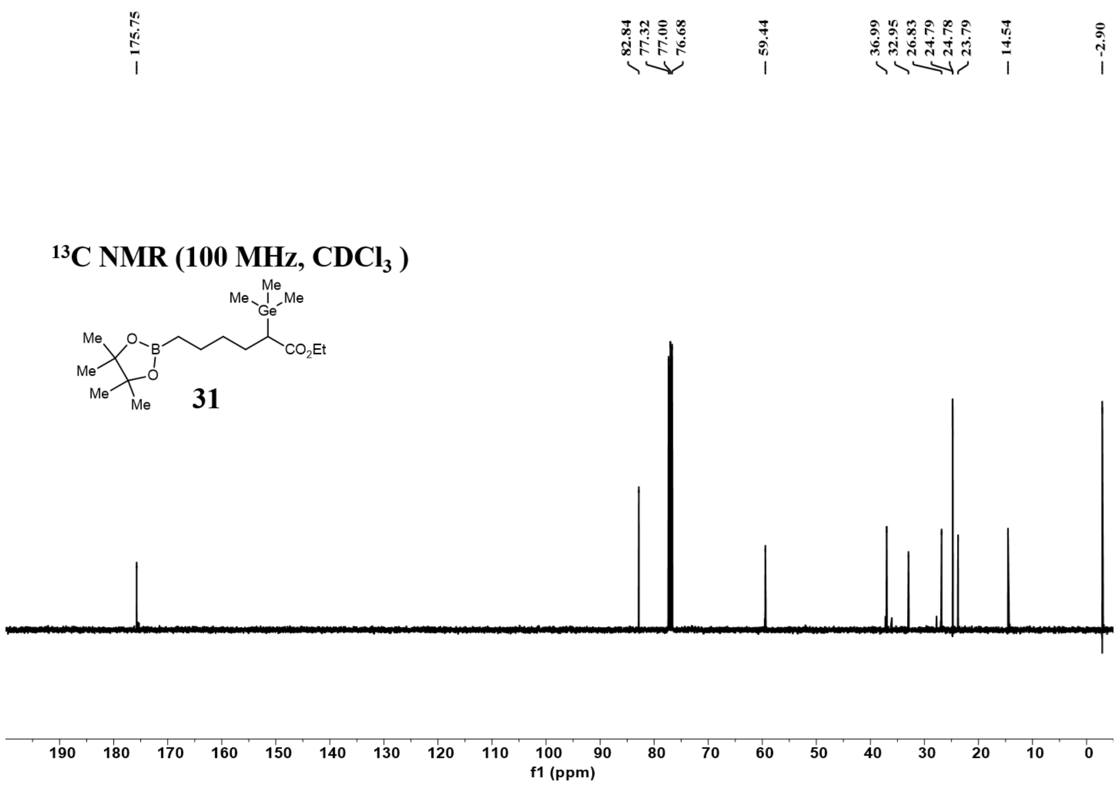

Supplementary Fig. 78. ^{13}C NMR of compound 31. The sample has been recorded in 100 MHz, $CDCl_3$ at 25 ºC

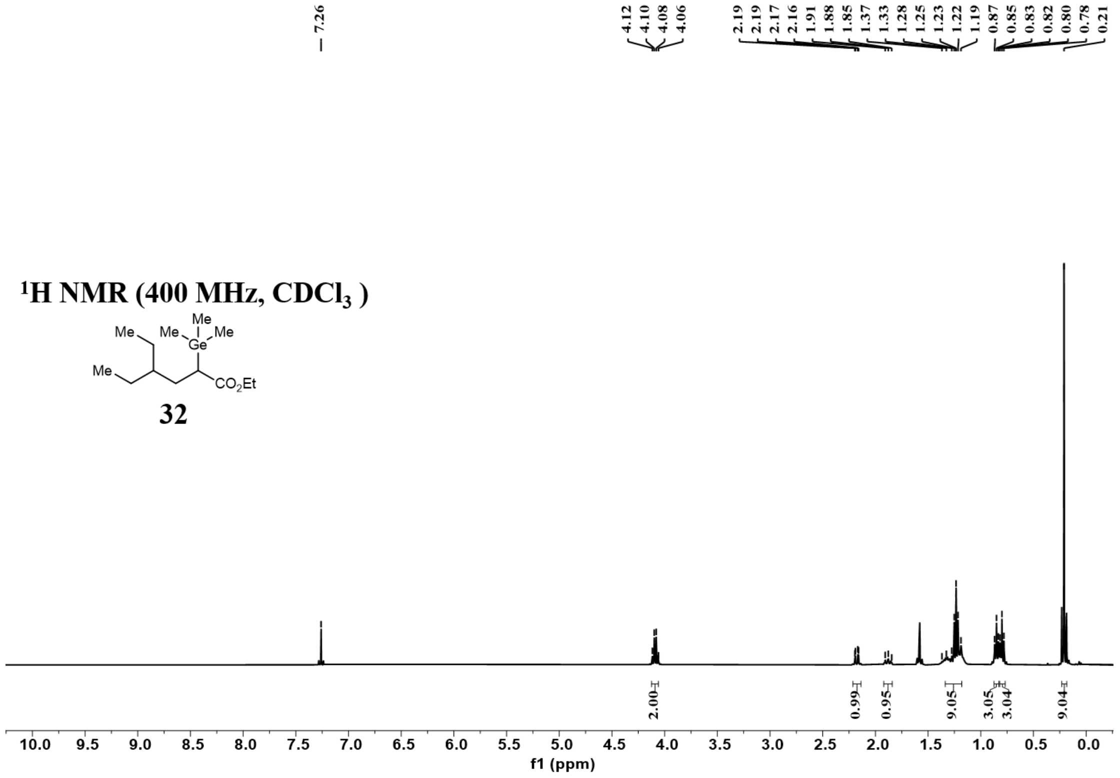

Supplementary Fig. 79. ^{1}H NMR of compound 32. The sample has been recorded in 400 MHz, $CDCl_3$ at 25 ºC

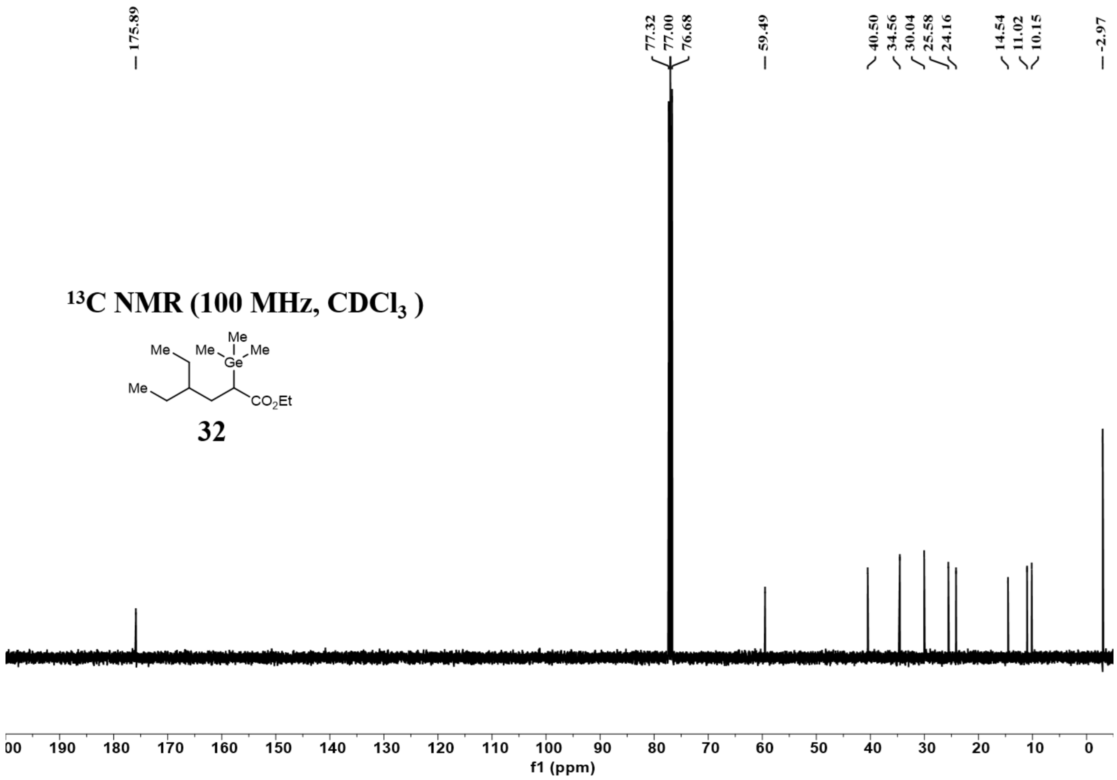

Supplementary Fig. 80. ^{13}C NMR of compound 32. The sample has been recorded in 100 MHz, $CDCl_3$ at 25 ºC

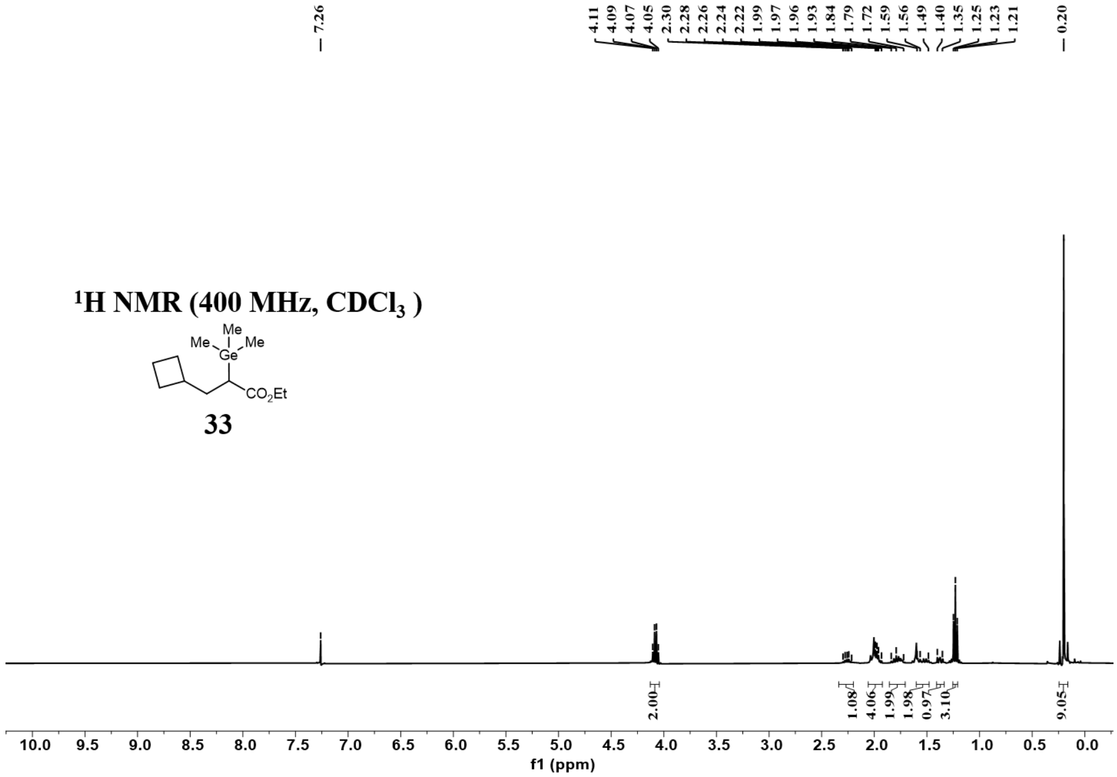

Supplementary Fig. 81. ^{1}H NMR of compound 33. The sample has been recorded in 400 MHz, $CDCl_3$ at 25 ºC

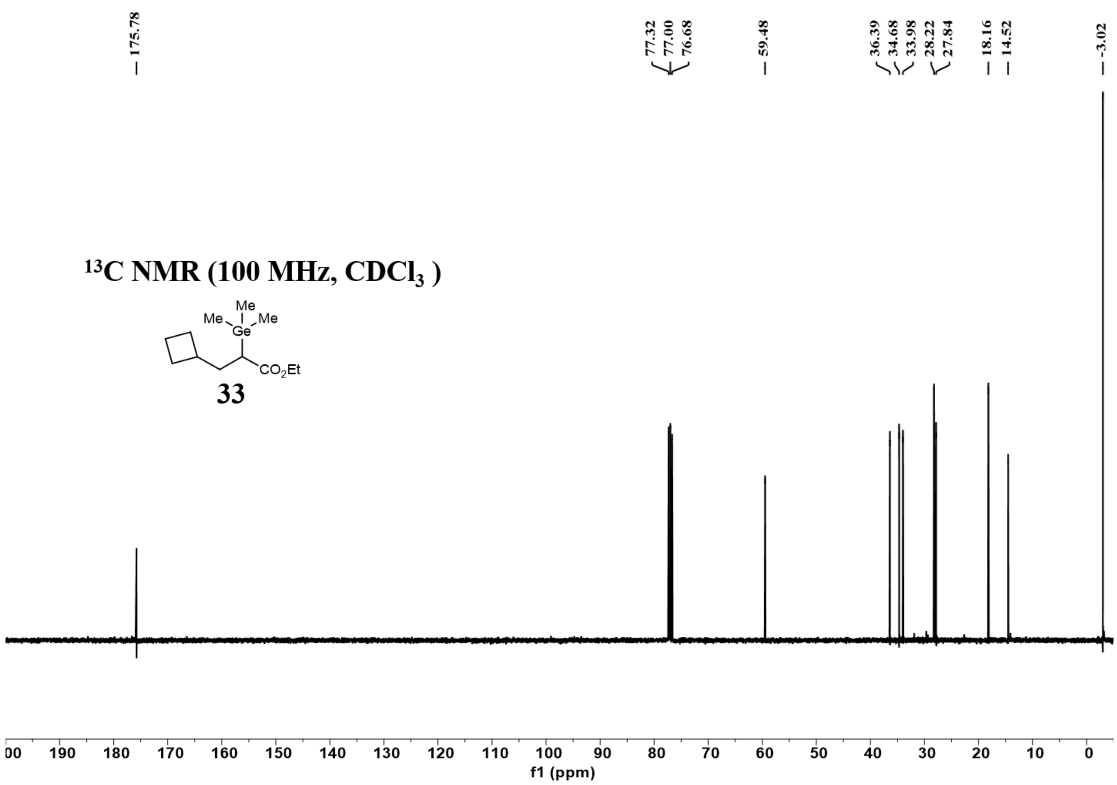

Supplementary Fig. 82. ^{13}C NMR of compound 33. The sample has been recorded in 100 MHz, $CDCl_3$ at 25 ºC

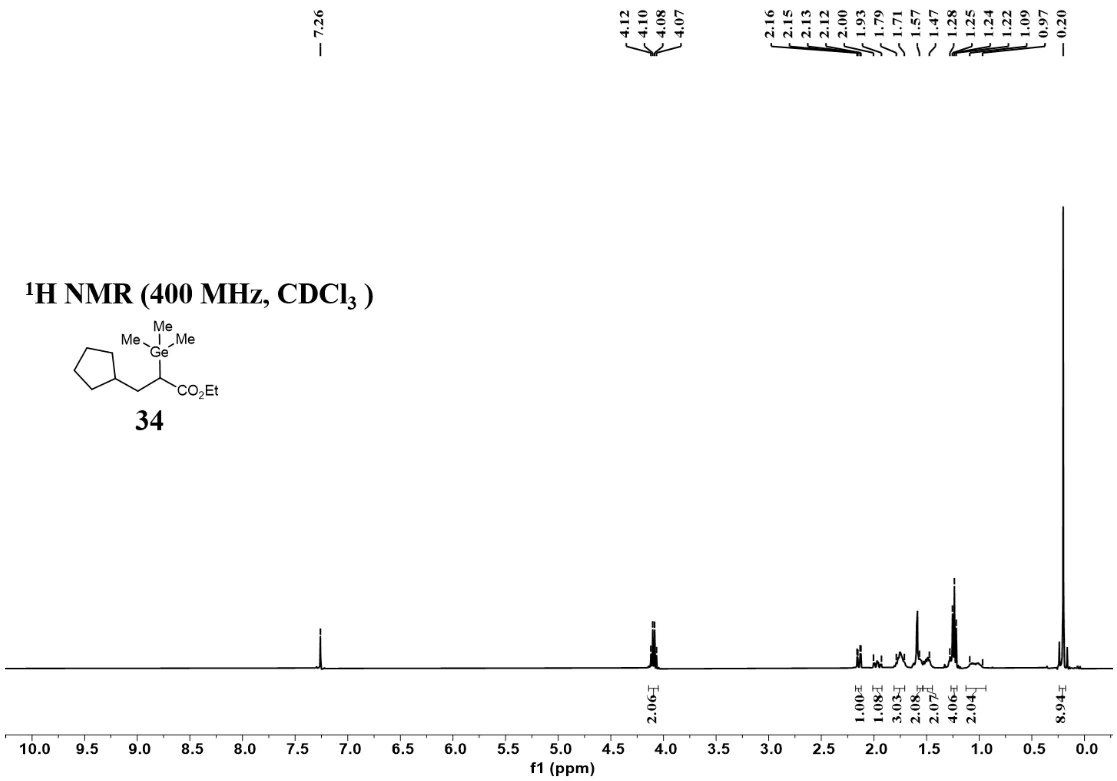

Supplementary Fig. 83. ^{1}H NMR of compound 34. The sample has been recorded in 400 MHz, $CDCl_3$ at 25 ºC

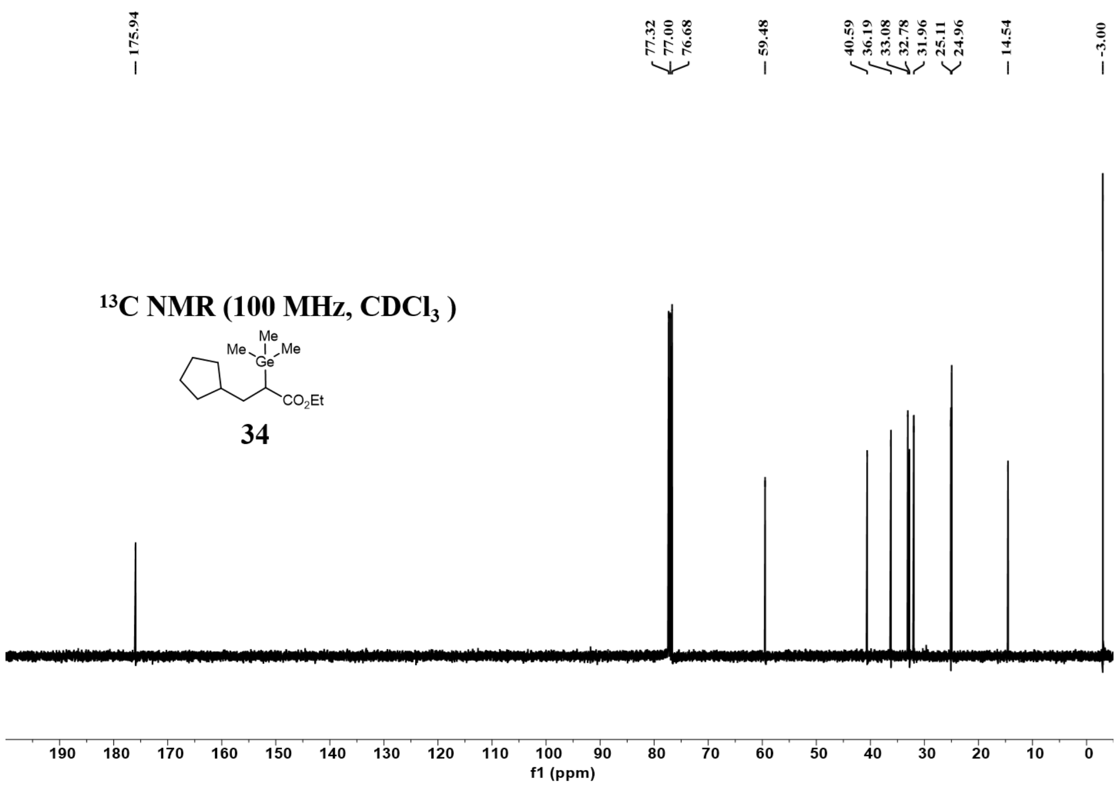

Supplementary Fig. 84. ^{13}C NMR of compound 34. The sample has been recorded in 100 MHz, $CDCl_3$ at 25 ºC

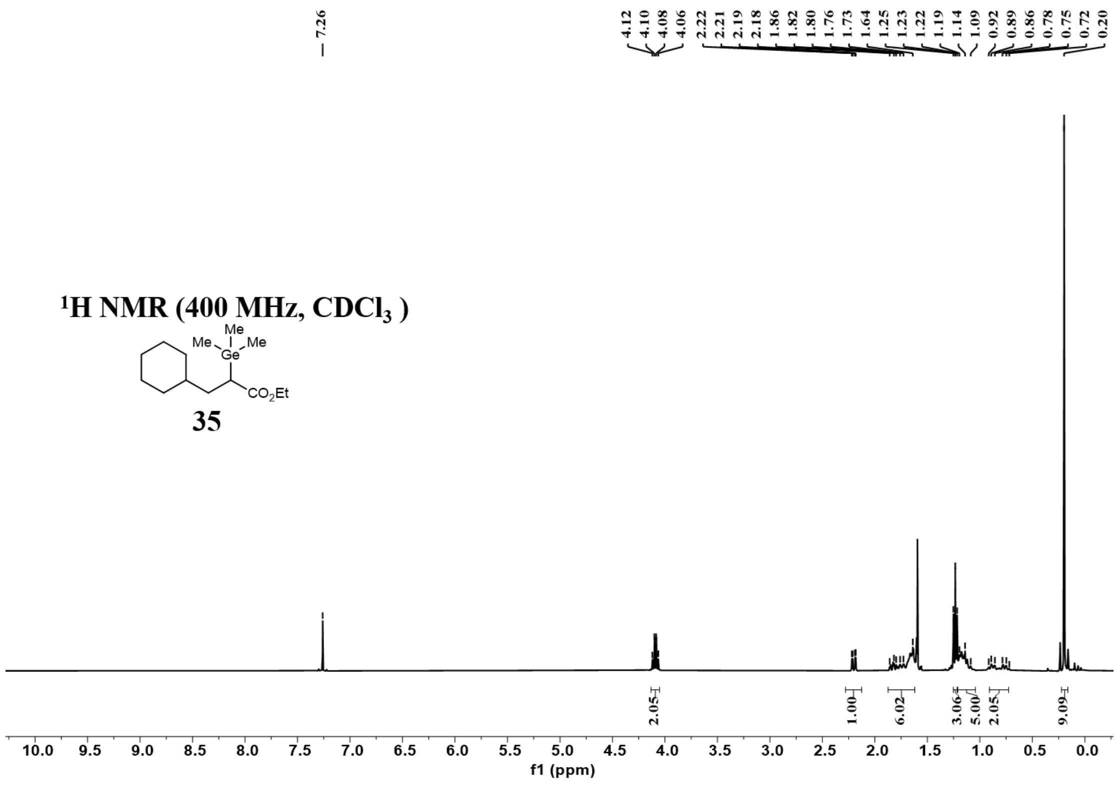

Supplementary Fig. 85. ^{1}H NMR of compound 35. The sample has been recorded in 400 MHz, $CDCl_3$ at 25 ºC

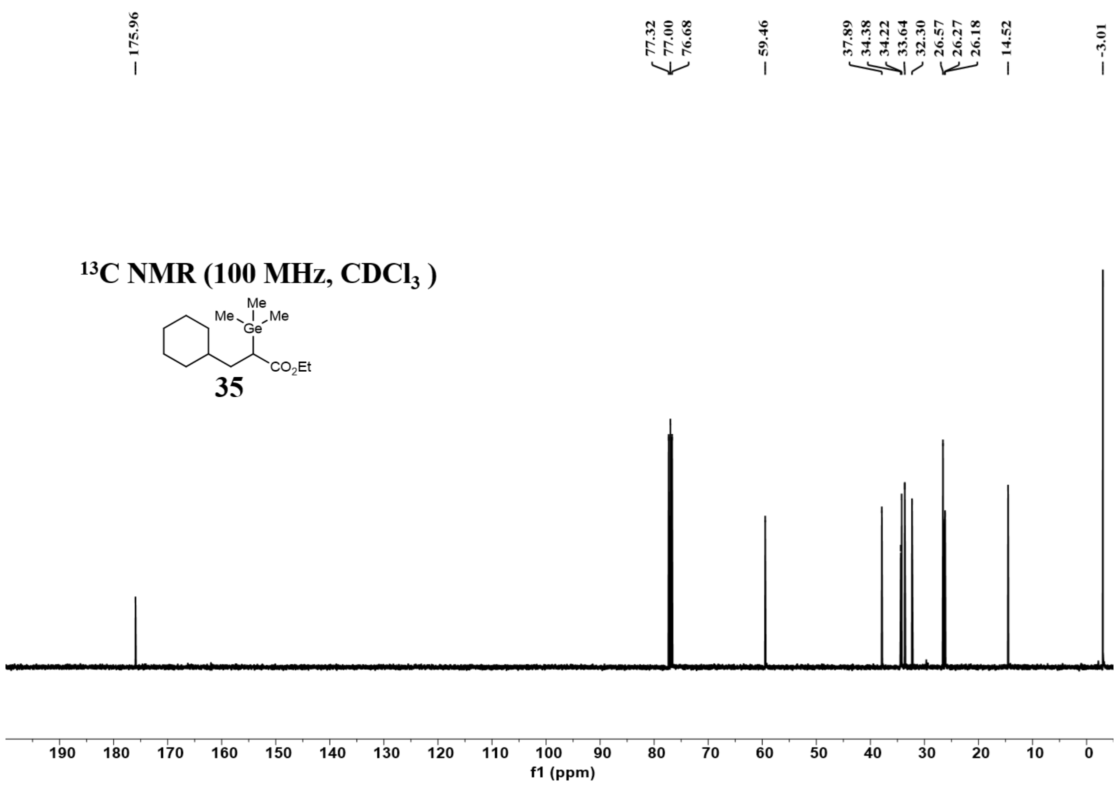

Supplementary Fig. 86. ^{13}C NMR of compound 35. The sample has been recorded in 100 MHz, $CDCl_3$ at 25 ºC

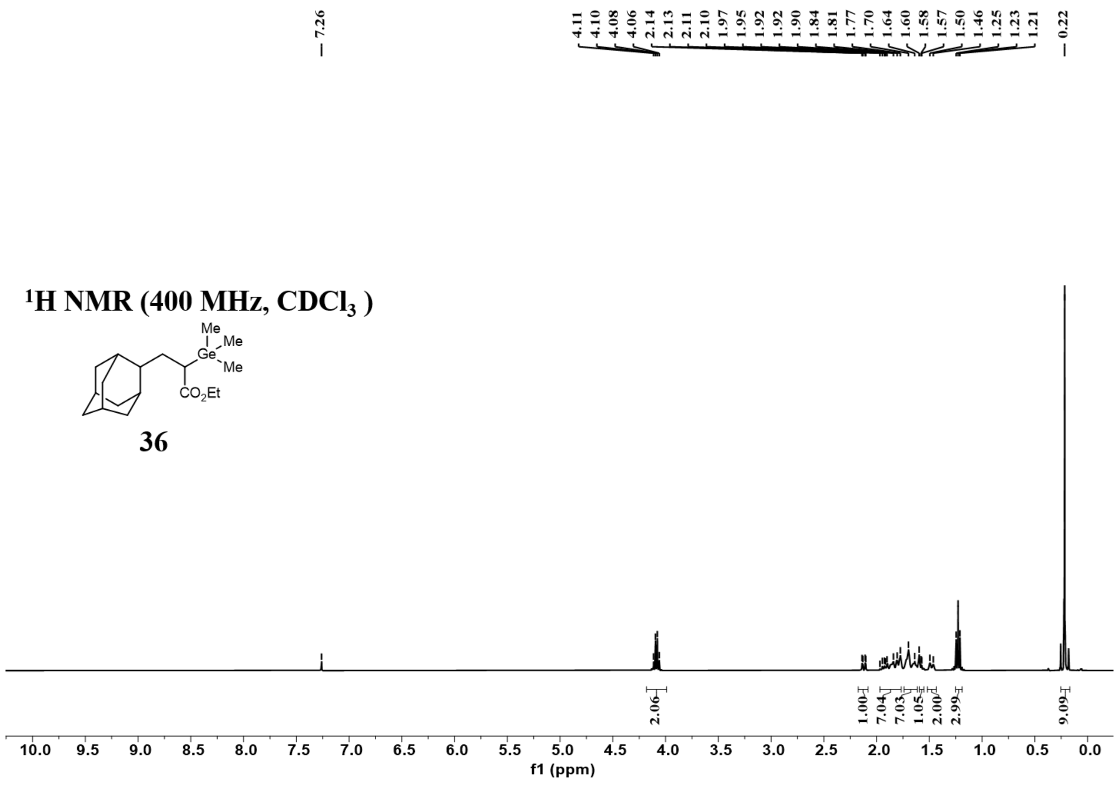

Supplementary Fig. 87. ^{1}H NMR of compound 36. The sample has been recorded in 400 MHz, $CDCl_3$ at 25 ºC

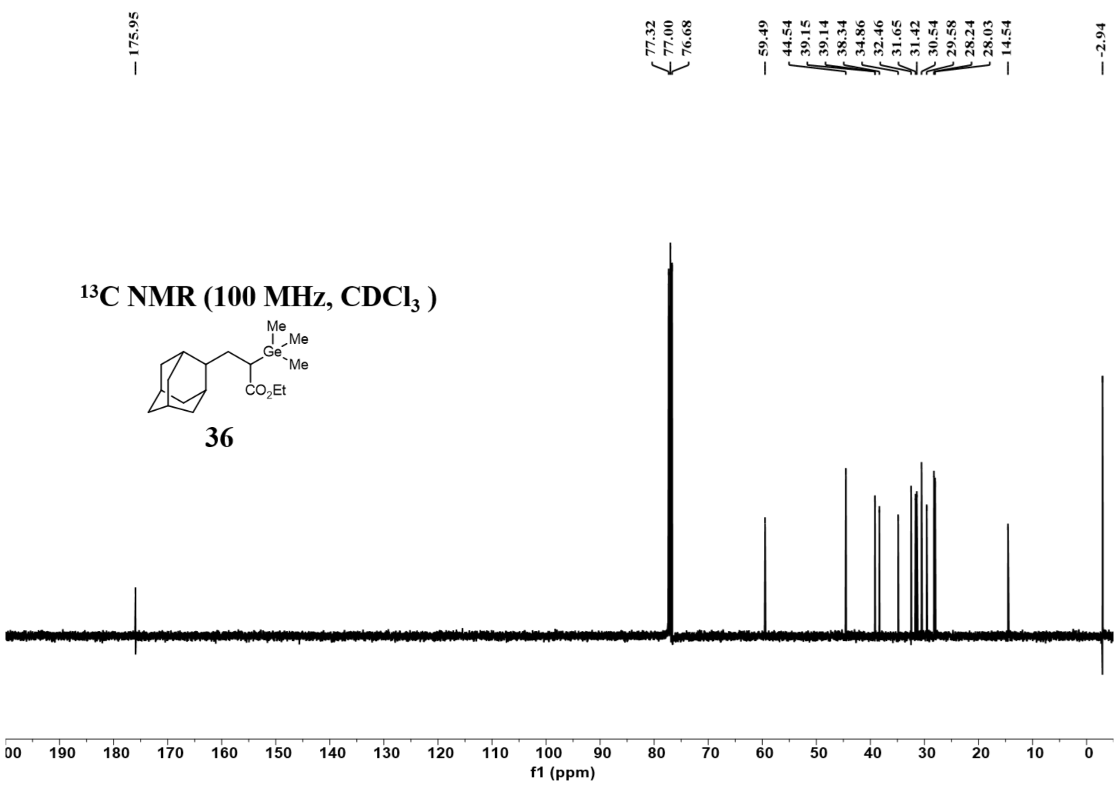

Supplementary Fig. 88. ^{13}C NMR of compound 36. The sample has been recorded in 100 MHz, $CDCl_3$ at 25 ºC

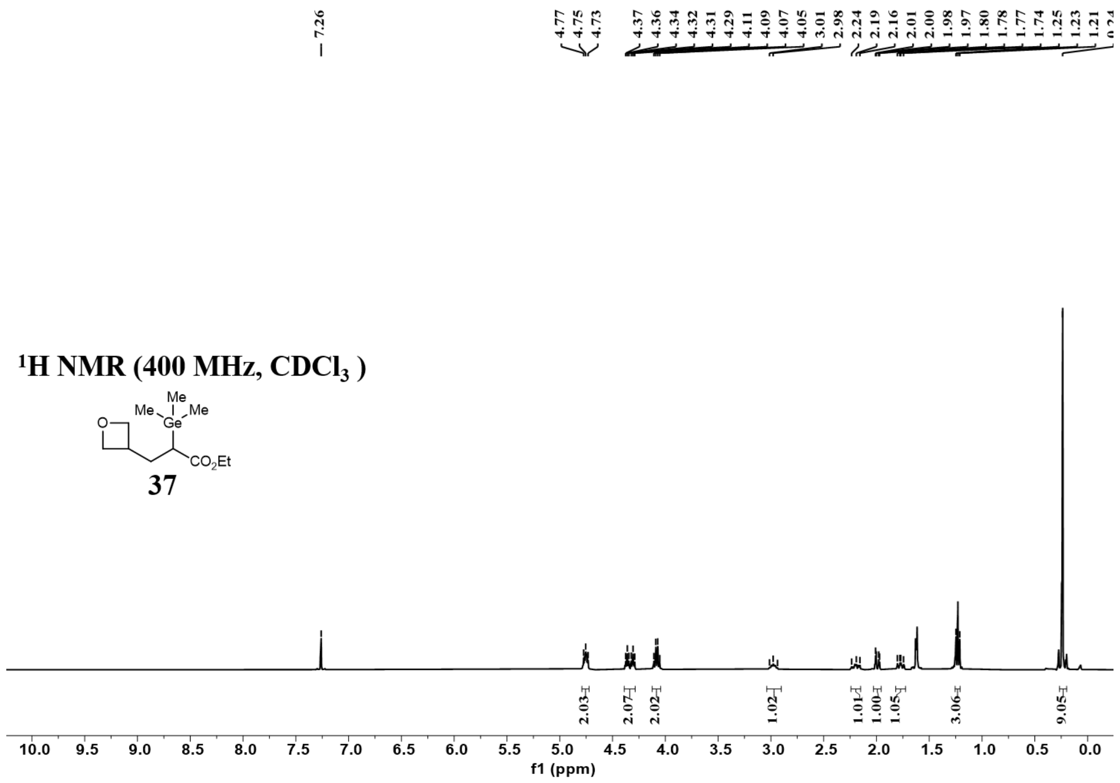

Supplementary Fig. 89. ^{1}H NMR of compound 37. The sample has been recorded in 400 MHz, $CDCl_3$ at 25 ºC

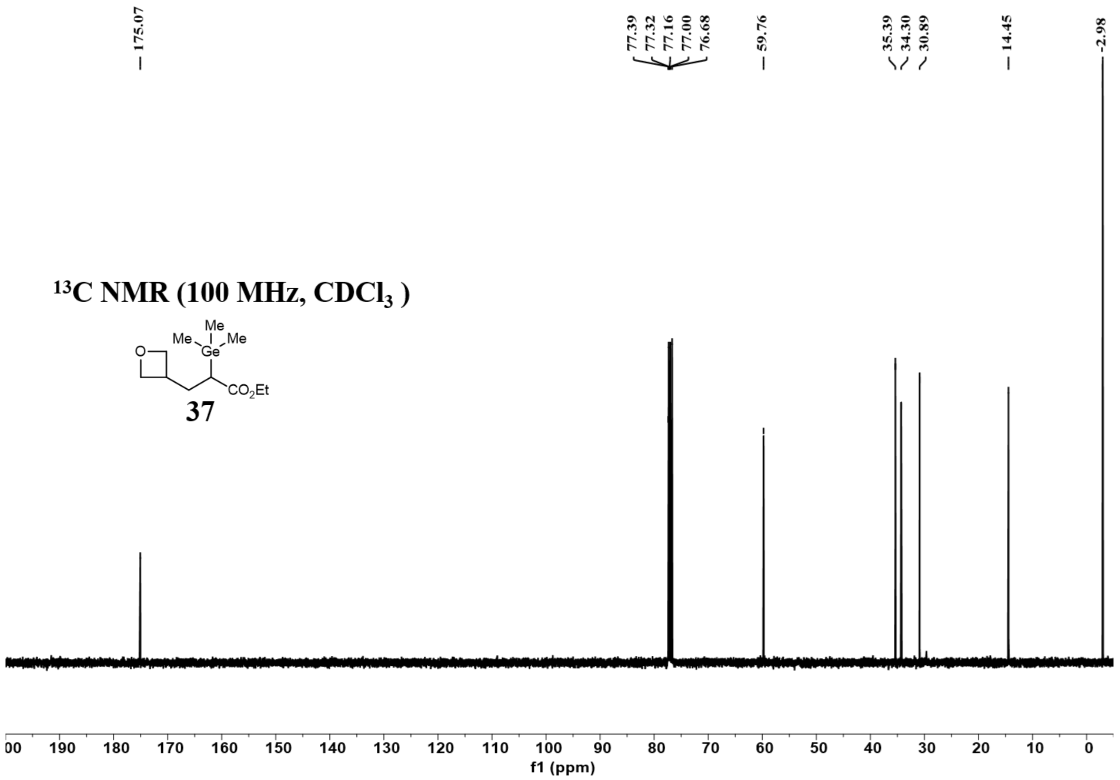

Supplementary Fig. 90. ^{13}C NMR of compound 37. The sample has been recorded in 100 MHz, $CDCl_3$ at 25 ºC

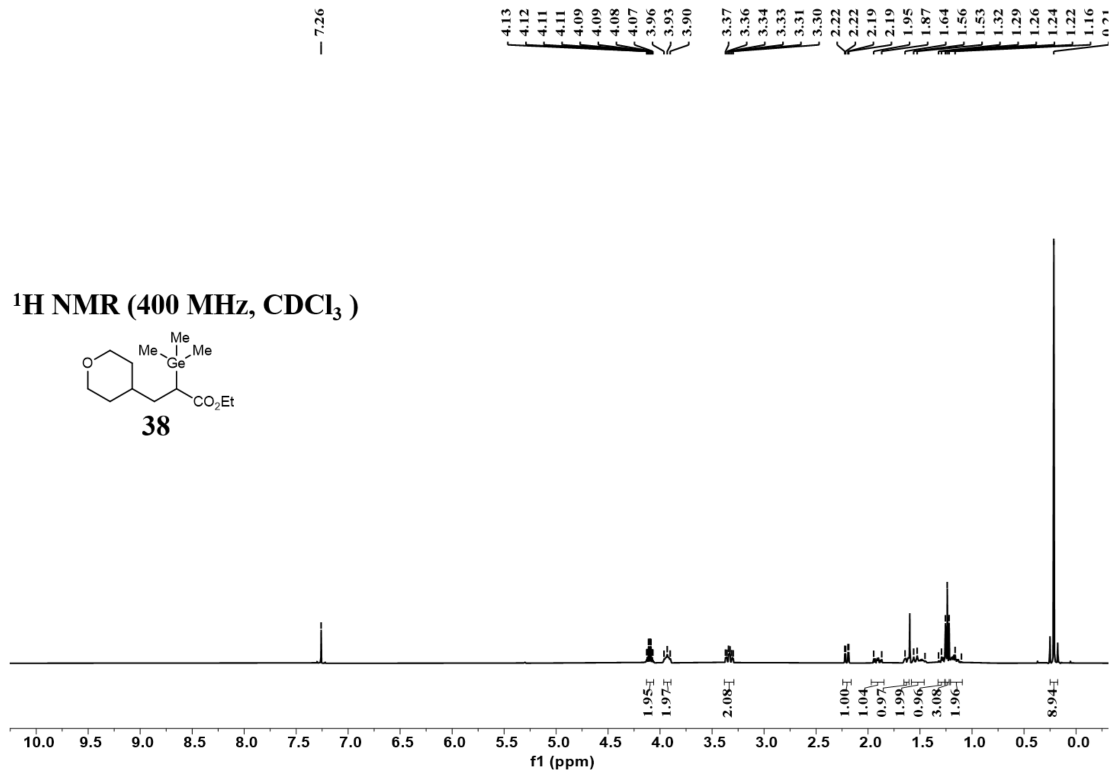

Supplementary Fig. 91. ^{1}H NMR of compound 38. The sample has been recorded in 400 MHz, $CDCl_3$ at 25 ºC.

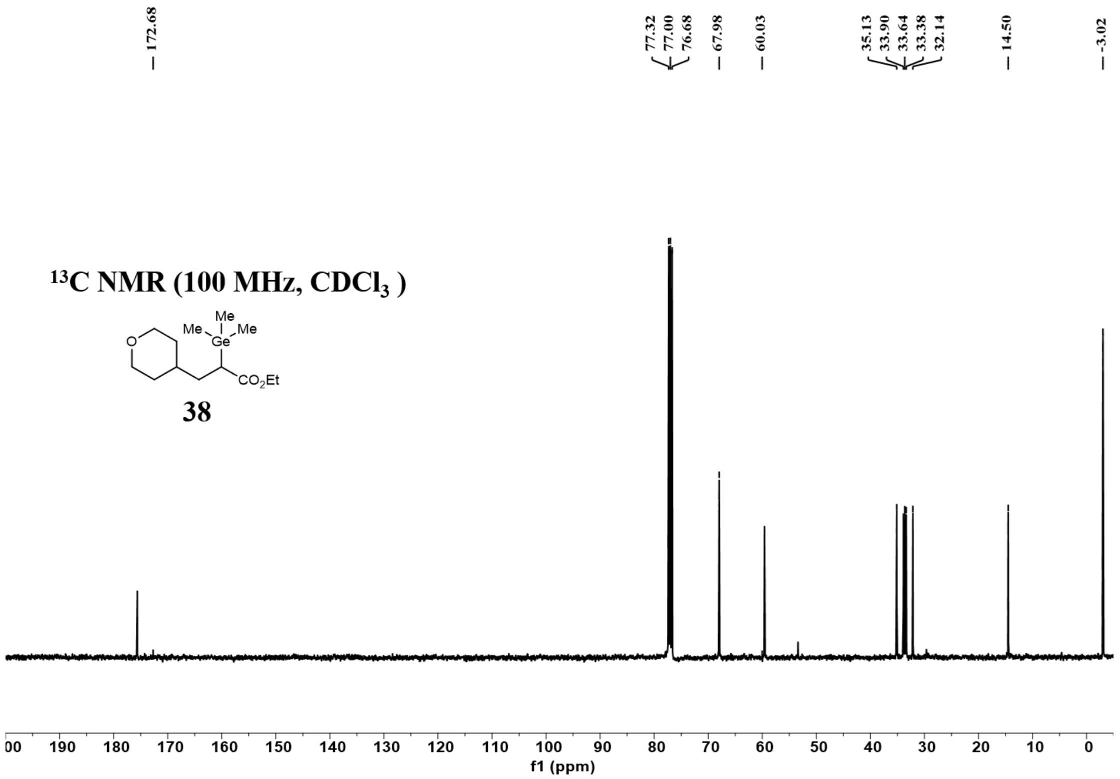

Supplementary Fig. 92. ^{13}C NMR of compound 38. The sample has been recorded in 100 MHz, $CDCl_3$ at 25 ºC

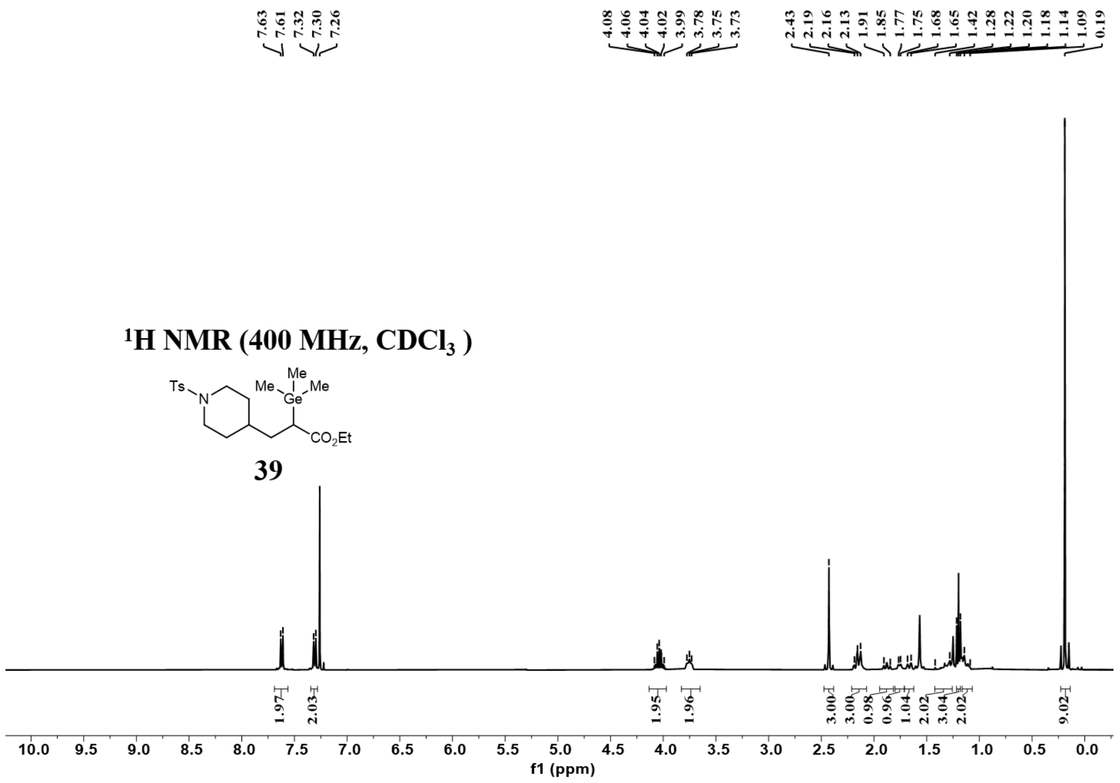

Supplementary Fig. 93. ^{1}H NMR of compound 39. The sample has been recorded in 400 MHz, $CDCl_3$ at 25 ºC

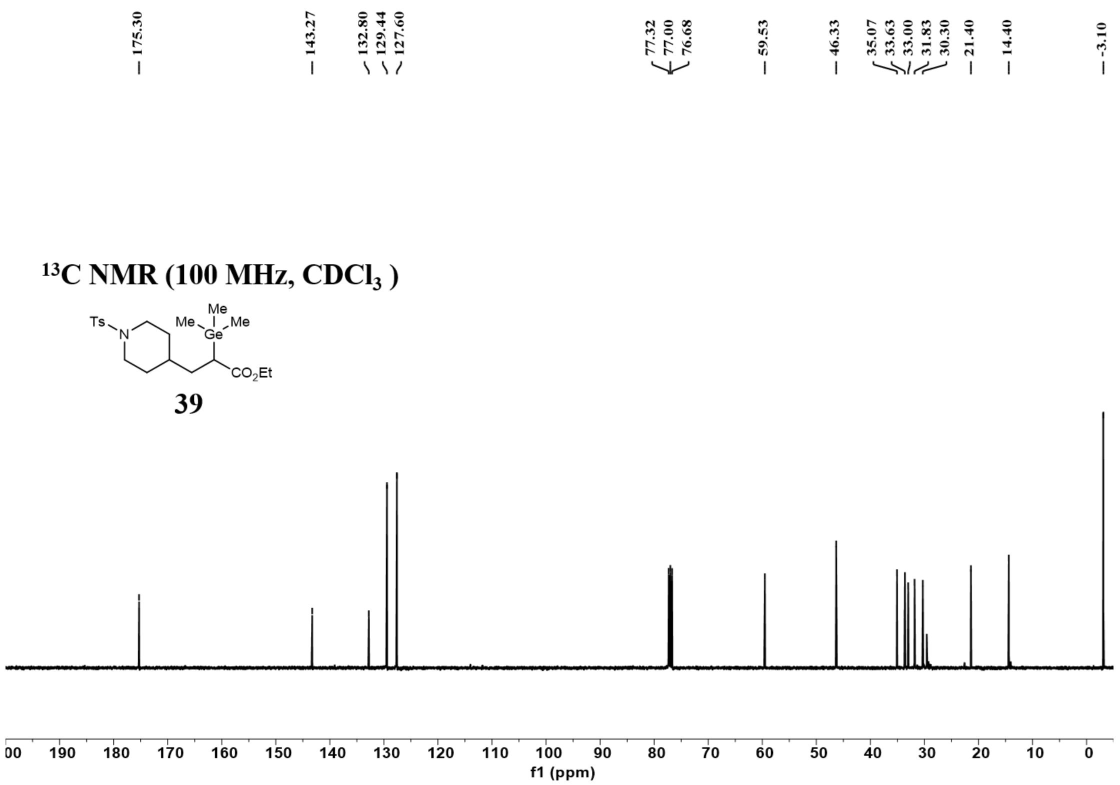

Supplementary Fig. 94. ^{13}C NMR of compound 39. The sample has been recorded in 100 MHz, $CDCl_3$ at 25 ºC

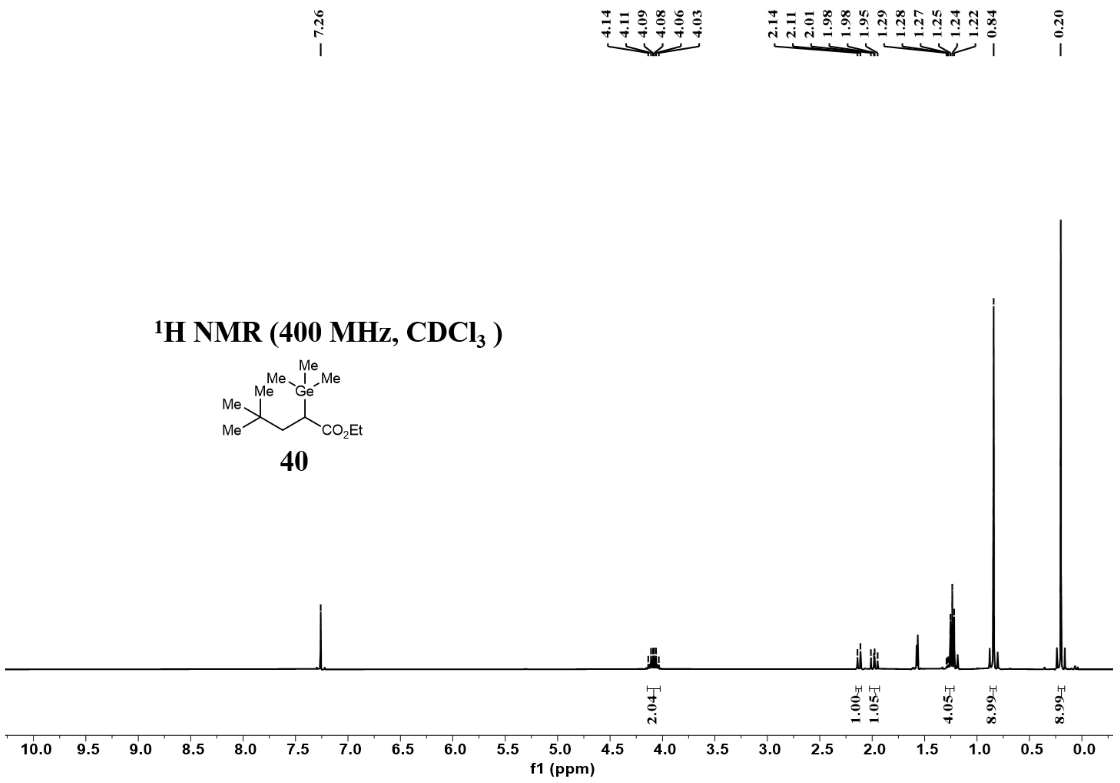

Supplementary Fig. 95. ^{1}H NMR of compound 40. The sample has been recorded in 400 MHz, $CDCl_3$ at 25 ºC

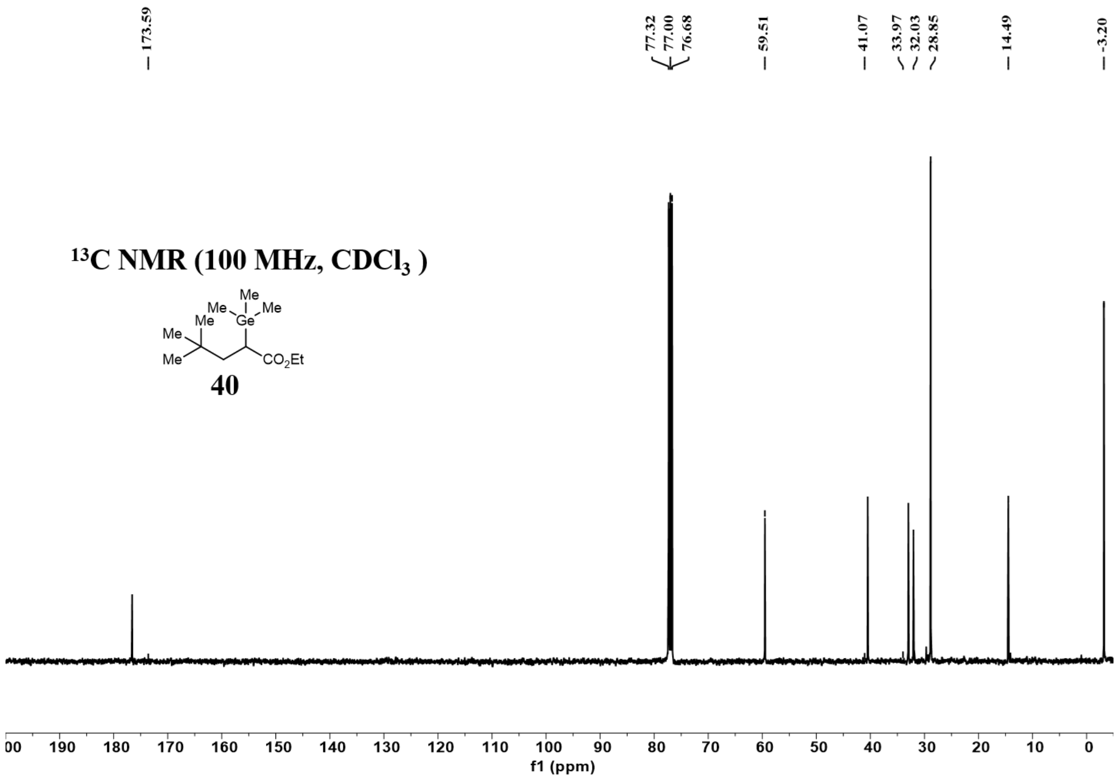

Supplementary Fig. 96. ^{13}C NMR of compound 40. The sample has been recorded in 100 MHz, $CDCl_3$ at 25 ºC

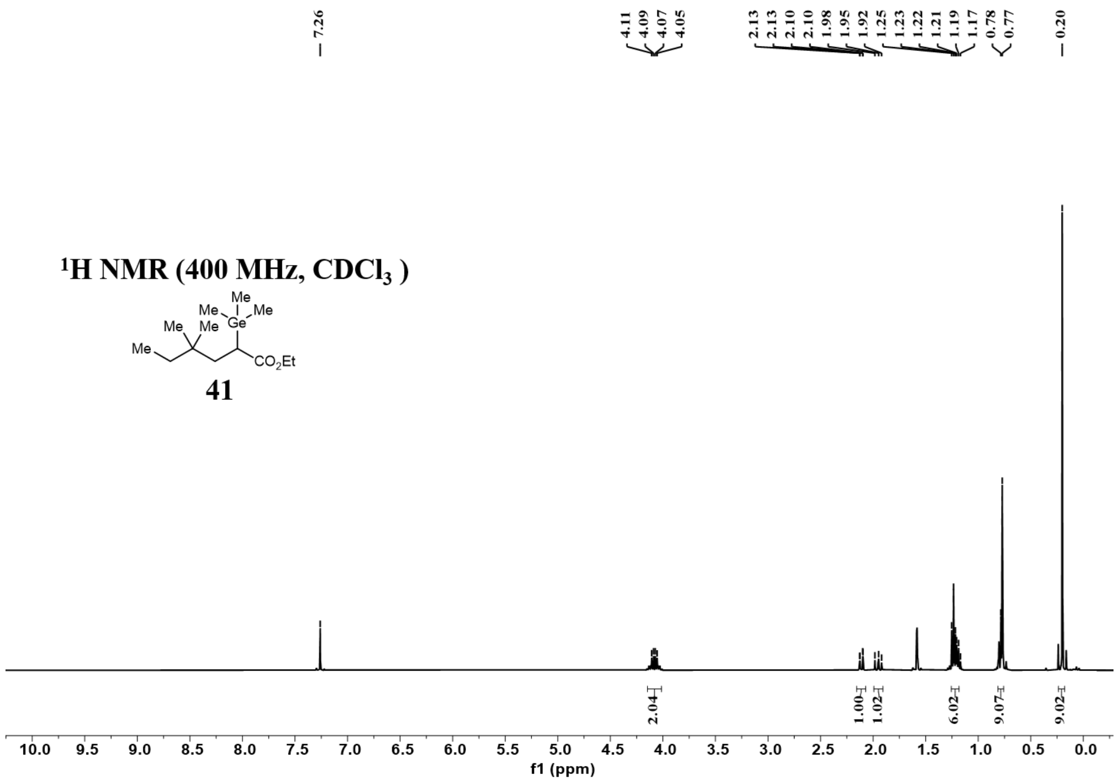

Supplementary Fig. 97. ^{1}H NMR of compound 41. The sample has been recorded in 400 MHz, $CDCl_3$ at 25 ºC

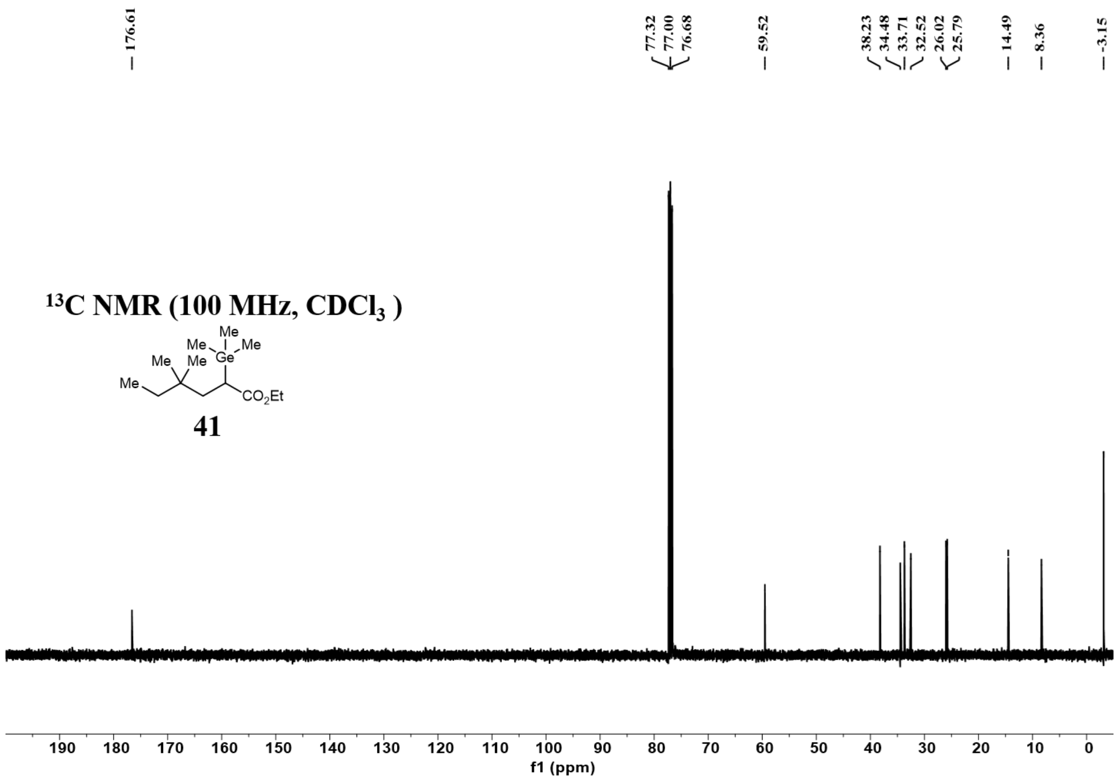

Supplementary Fig. 98. ^{13}C NMR of compound 41. The sample has been recorded in 100 MHz, $CDCl_3$ at 25 ºC.

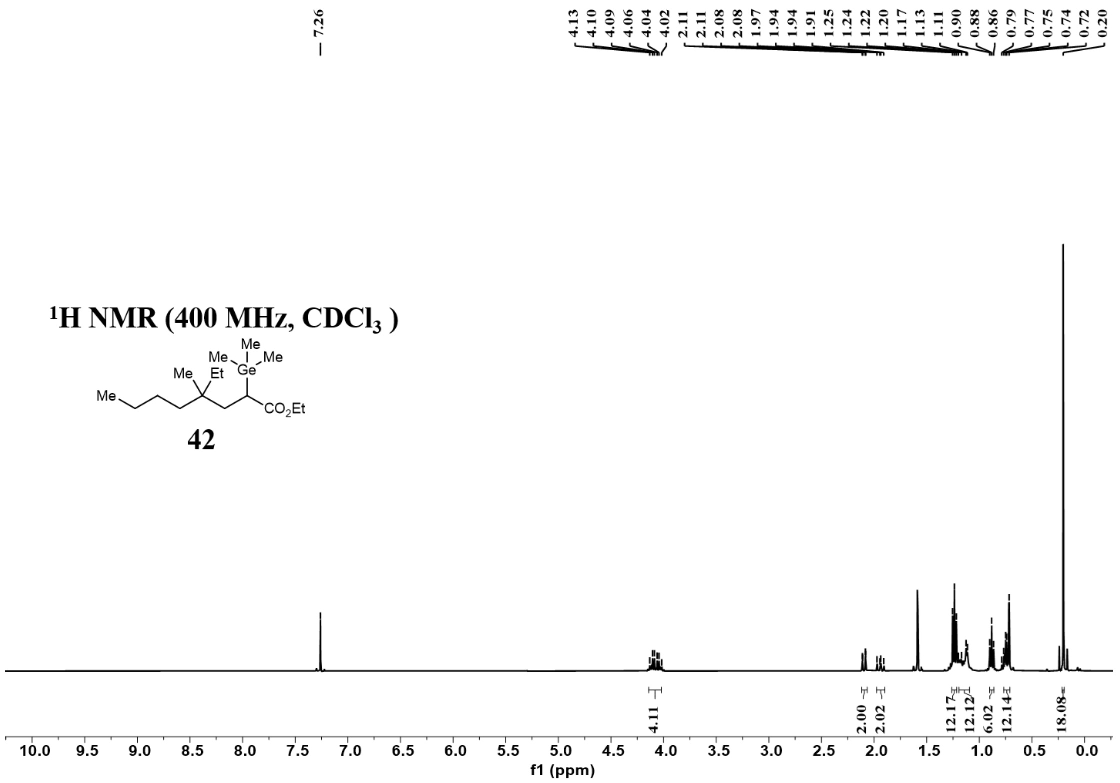

Supplementary Fig. 99. ^{1}H NMR of compound 42. The sample has been recorded in 400 MHz, $CDCl_3$ at 25 ºC

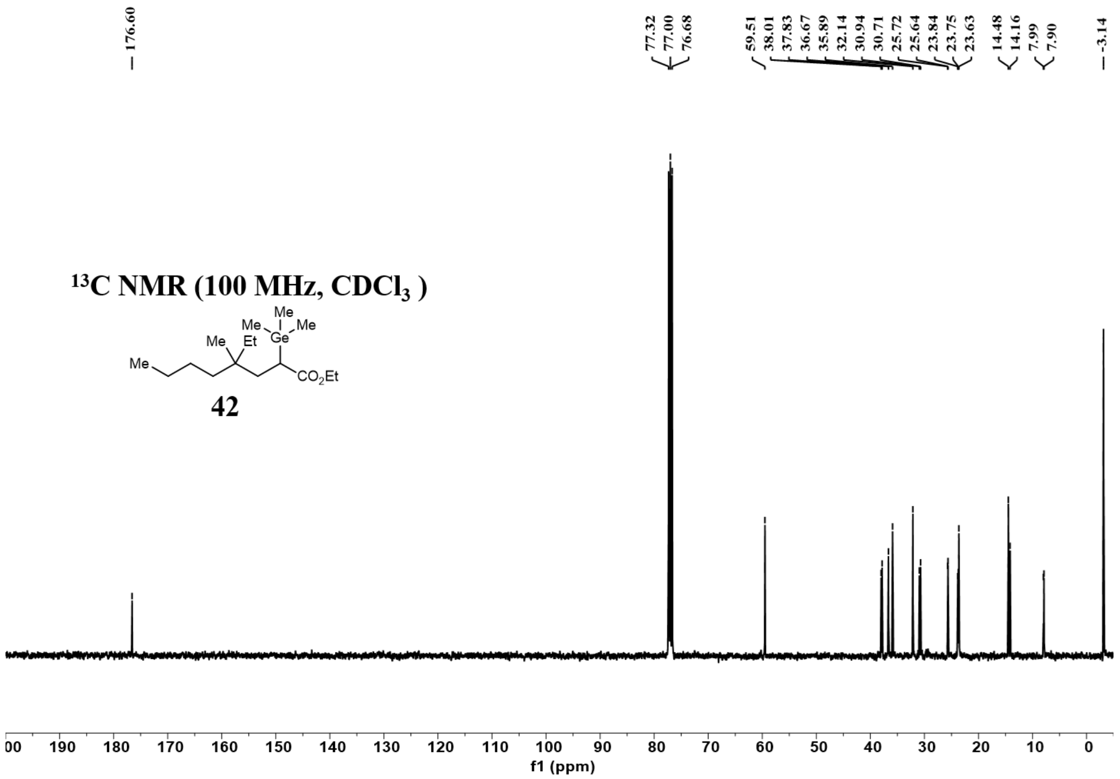

Supplementary Fig. 100. ^{13}C NMR of compound 42. The sample has been recorded in 100 MHz, $CDCl_3$ at 25 ºC.

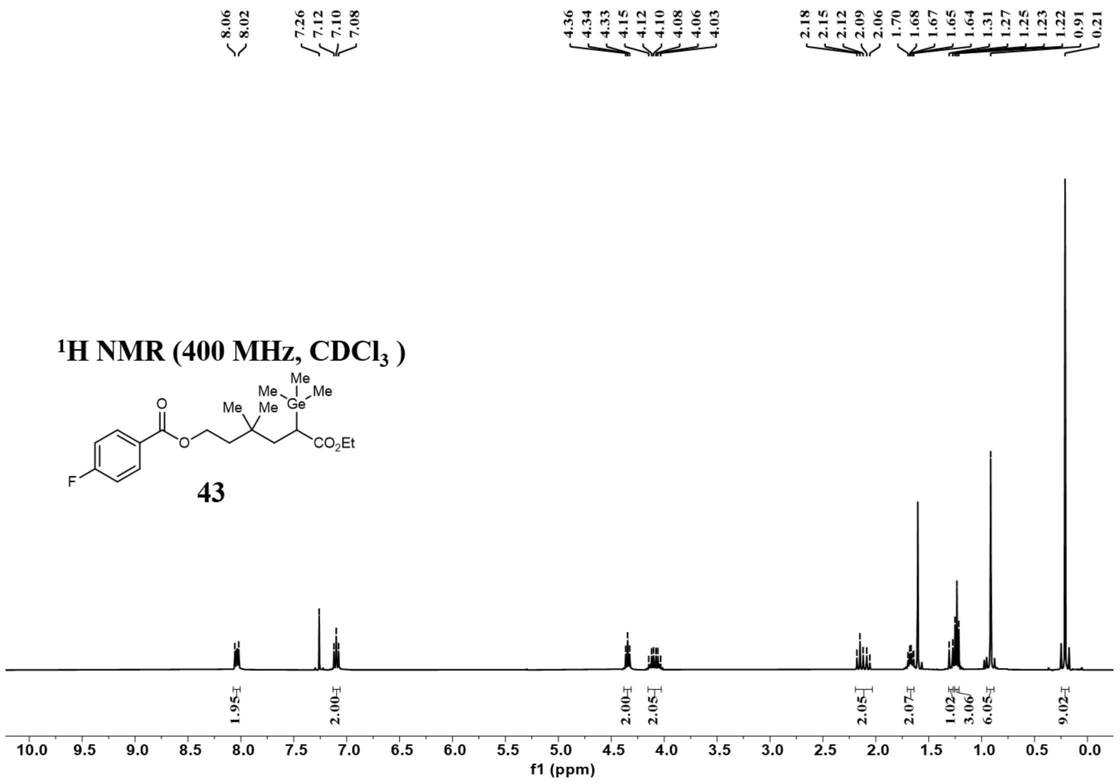

Supplementary Fig. 101. ^{1}H NMR of compound 43. The sample has been recorded in 400 MHz, $CDCl_3$ at 25 ºC

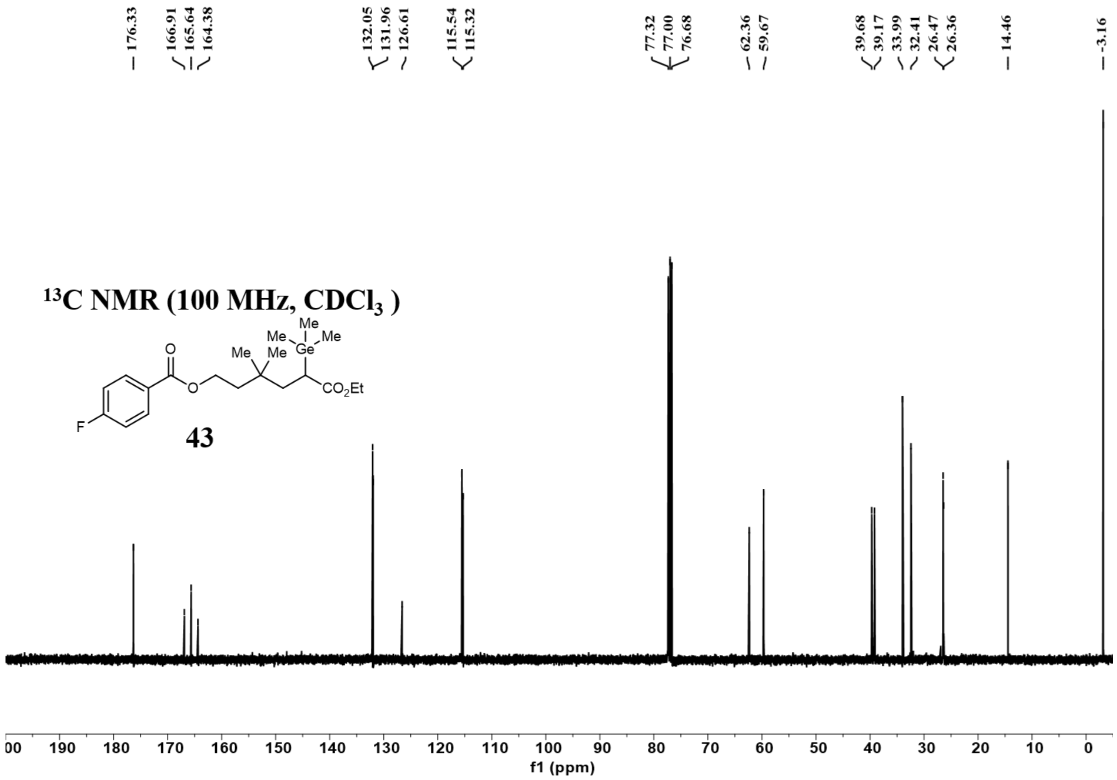

Supplementary Fig. 102. ^{13}C NMR of compound 43. The sample has been recorded in 100 MHz, $CDCl_3$ at 25 ºC.

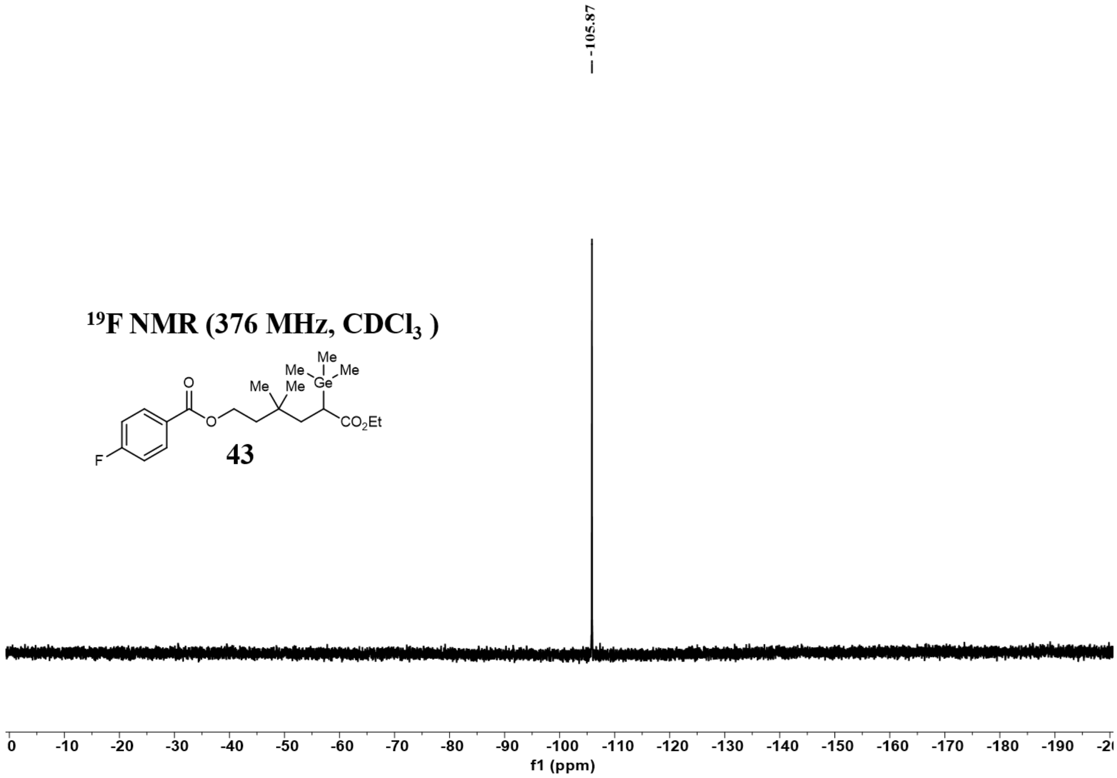

Supplementary Fig. 103. ^{19}F NMR of compound 43. The sample has been recorded in 376 MHz, $CDCl_3$ at 25 ºC

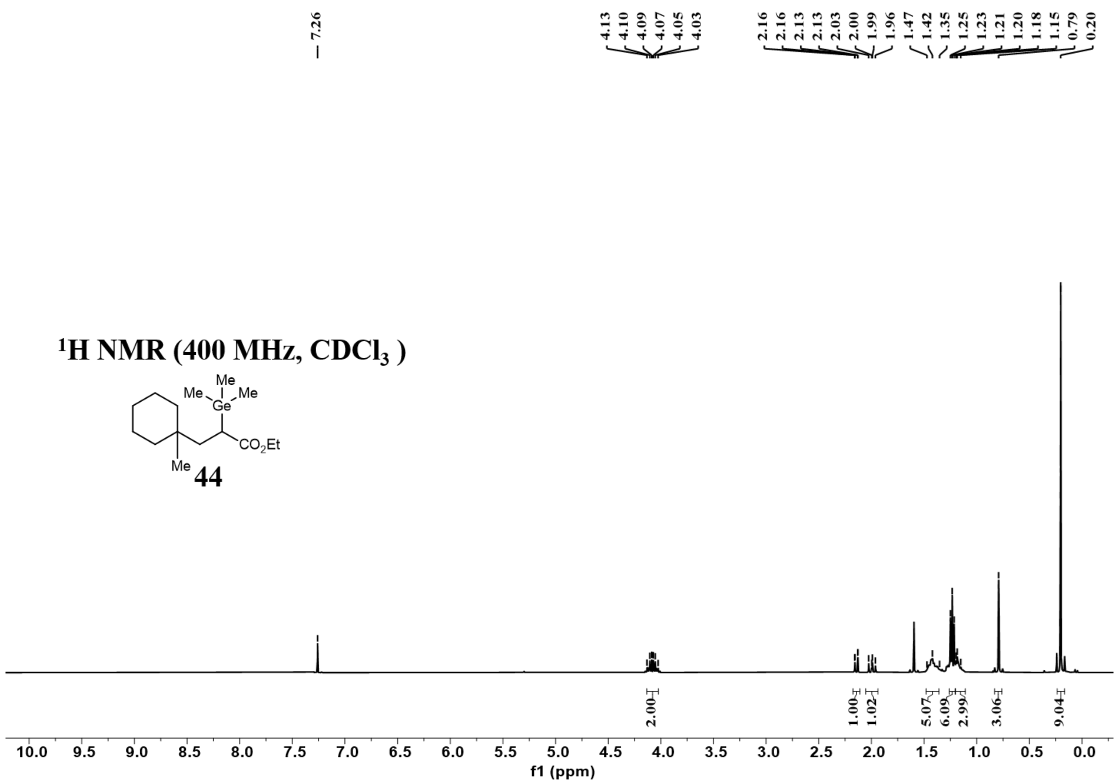

Supplementary Fig. 104. ^{1}H NMR of compound 44. The sample has been recorded in 400 MHz, $CDCl_3$ at 25 ºC

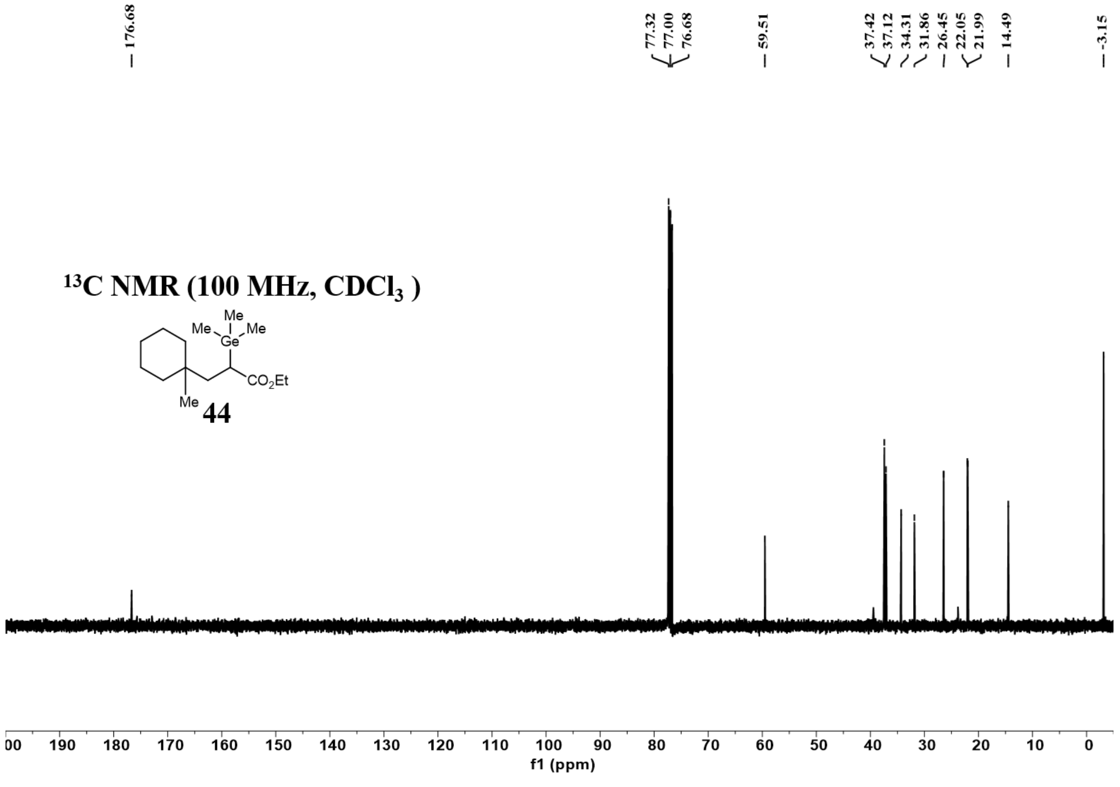

Supplementary Fig. 105. ^{13}C NMR of compound 44. The sample has been recorded in 100 MHz, $CDCl_3$ at 25 ºC.

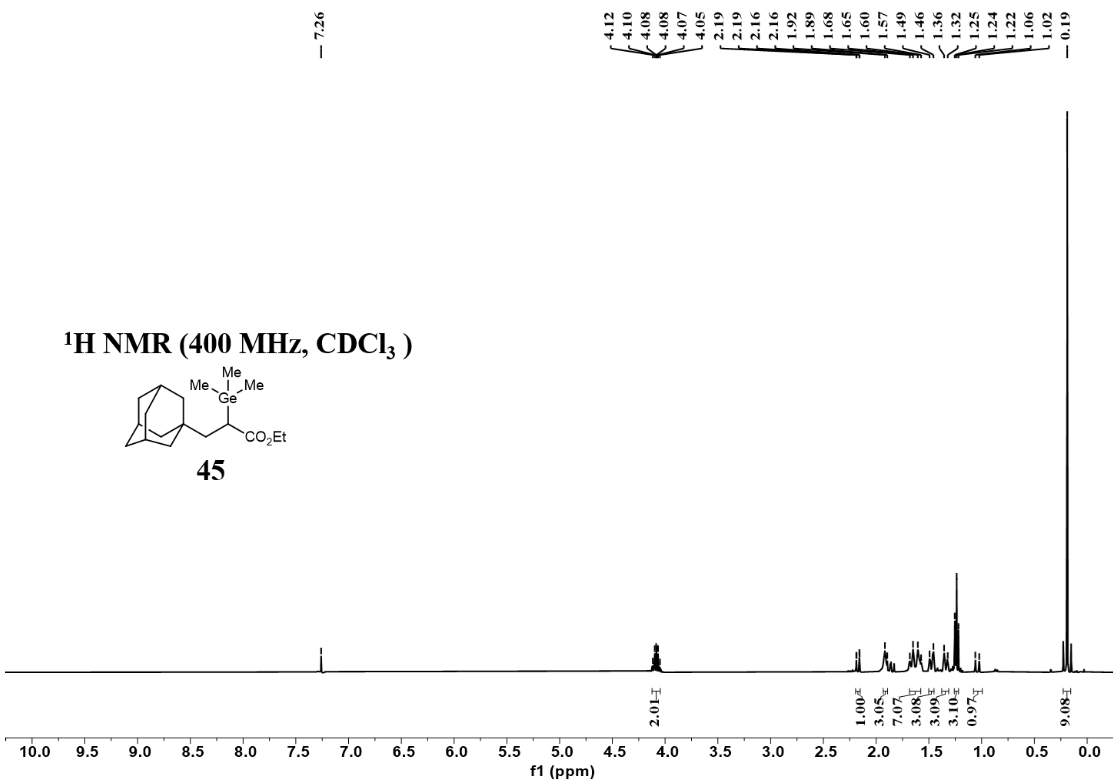

Supplementary Fig. 106. ^{1}H NMR of compound 45. The sample has been recorded in 400 MHz, $CDCl_3$ at 25 ºC

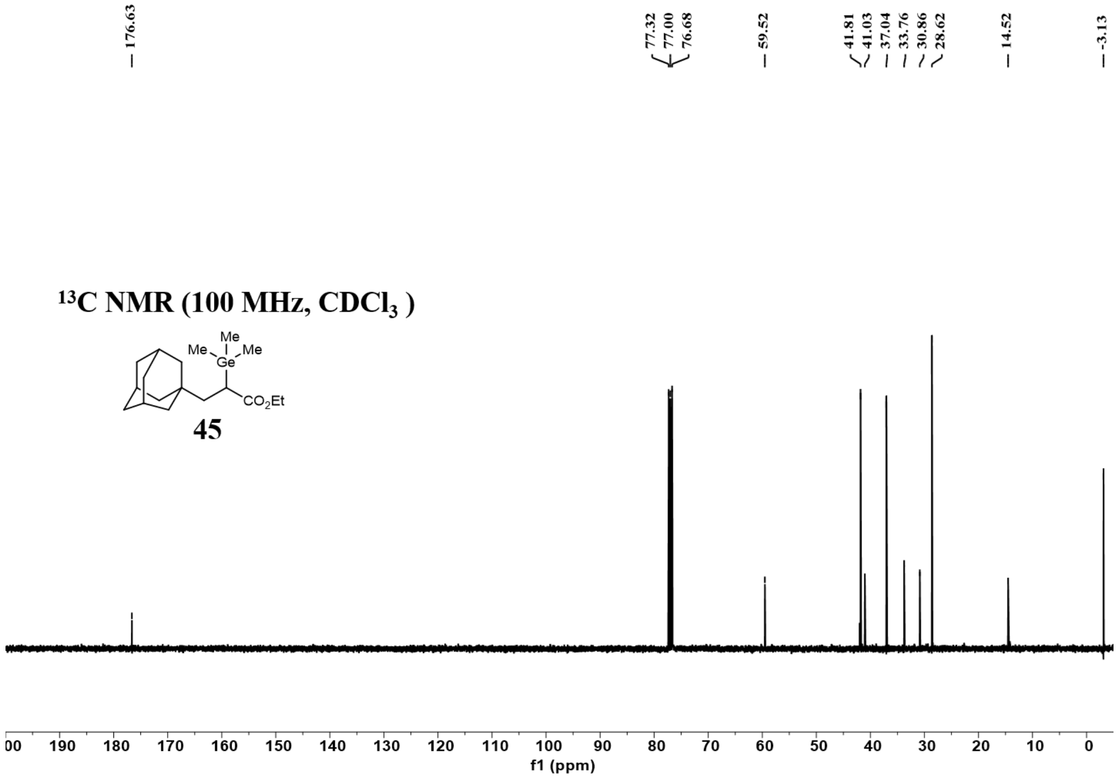

Supplementary Fig. 107. ^{13}C NMR of compound 45. The sample has been recorded in 100 MHz, $CDCl_3$ at 25 ºC.

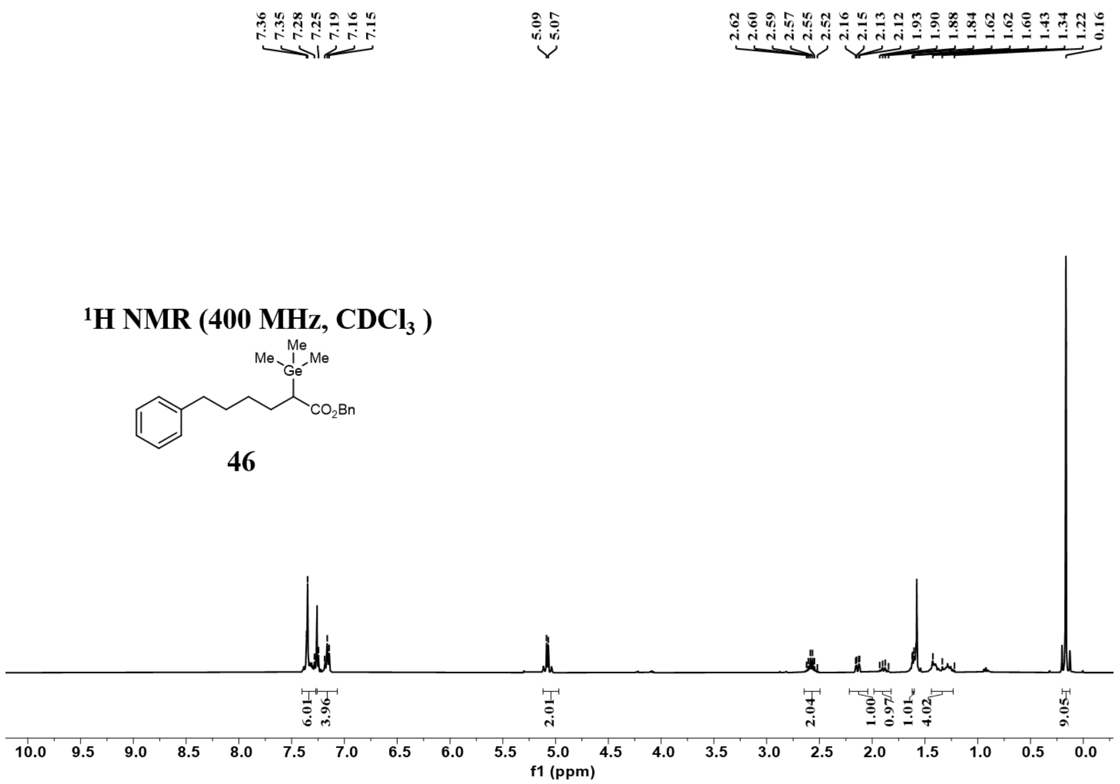

Supplementary Fig. 108. ^{1}H NMR of compound 46. The sample has been recorded in 400 MHz, $CDCl_3$ at 25 ºC

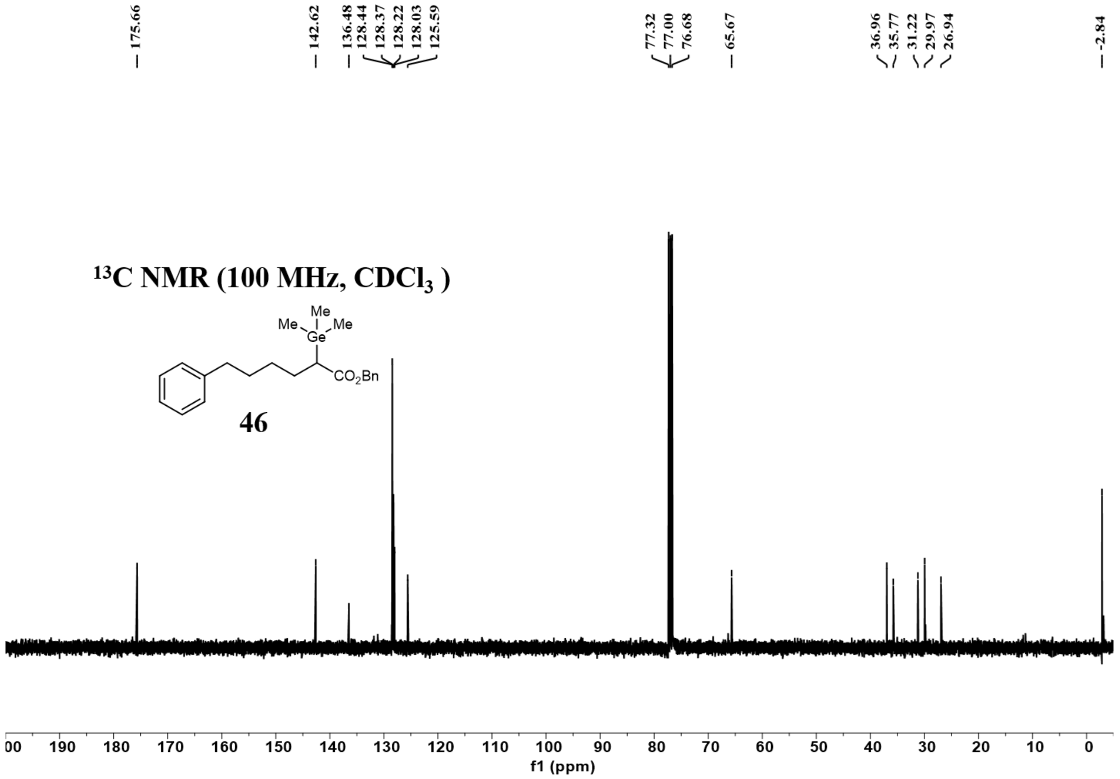

Supplementary Fig. 109. ^{13}C NMR of compound 46. The sample has been recorded in 100 MHz, $CDCl_3$ at 25 ºC.

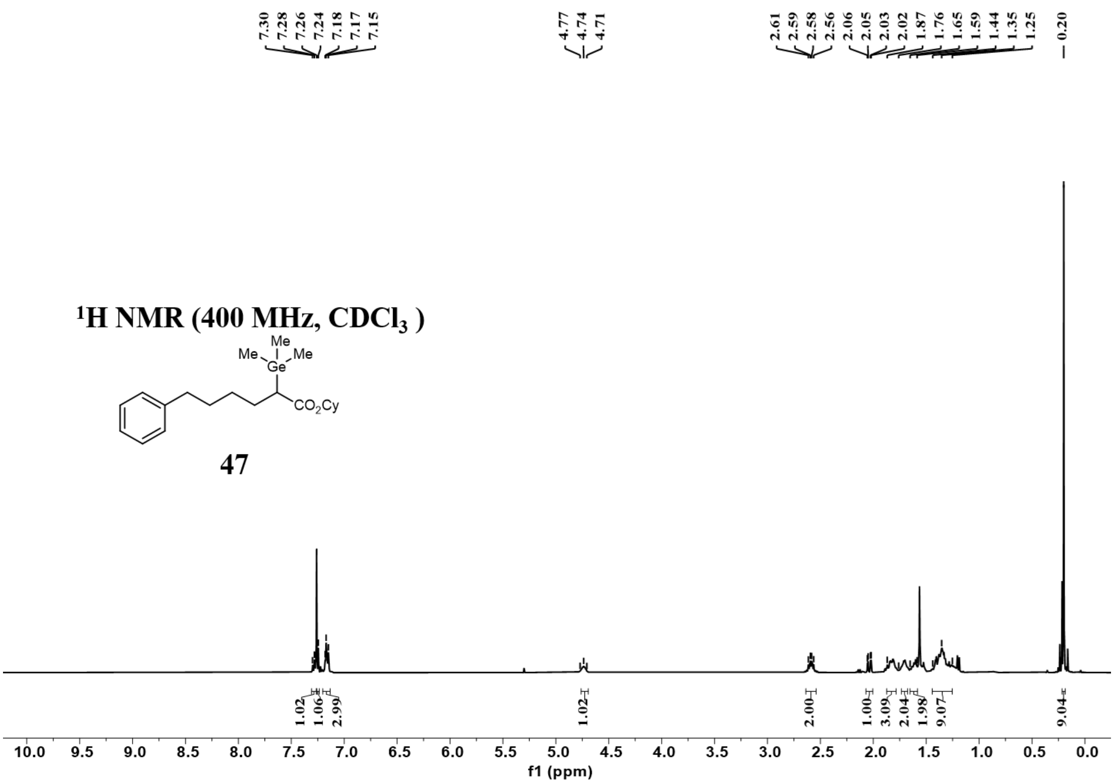

Supplementary Fig. 110. ^{1}H NMR of compound 47. The sample has been recorded in 400 MHz, $CDCl_3$ at 25 ºC

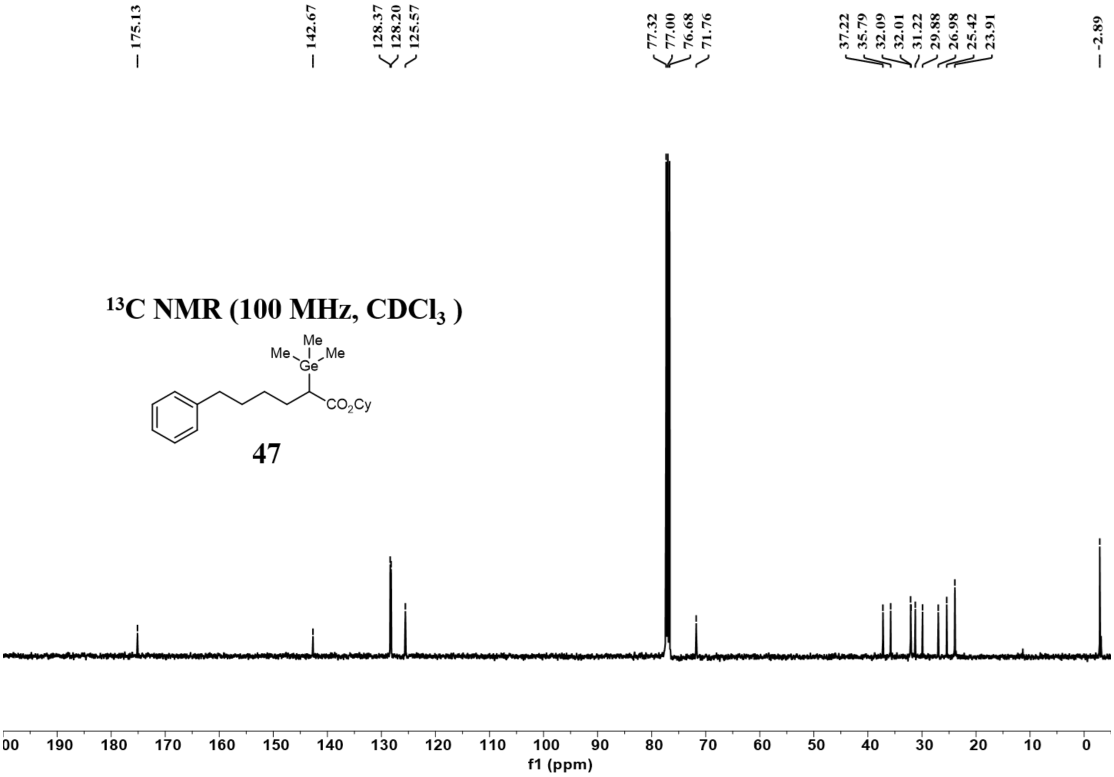

Supplementary Fig. 111. ^{13}C NMR of compound 47. The sample has been recorded in 100 MHz, $CDCl_3$ at 25 ºC.

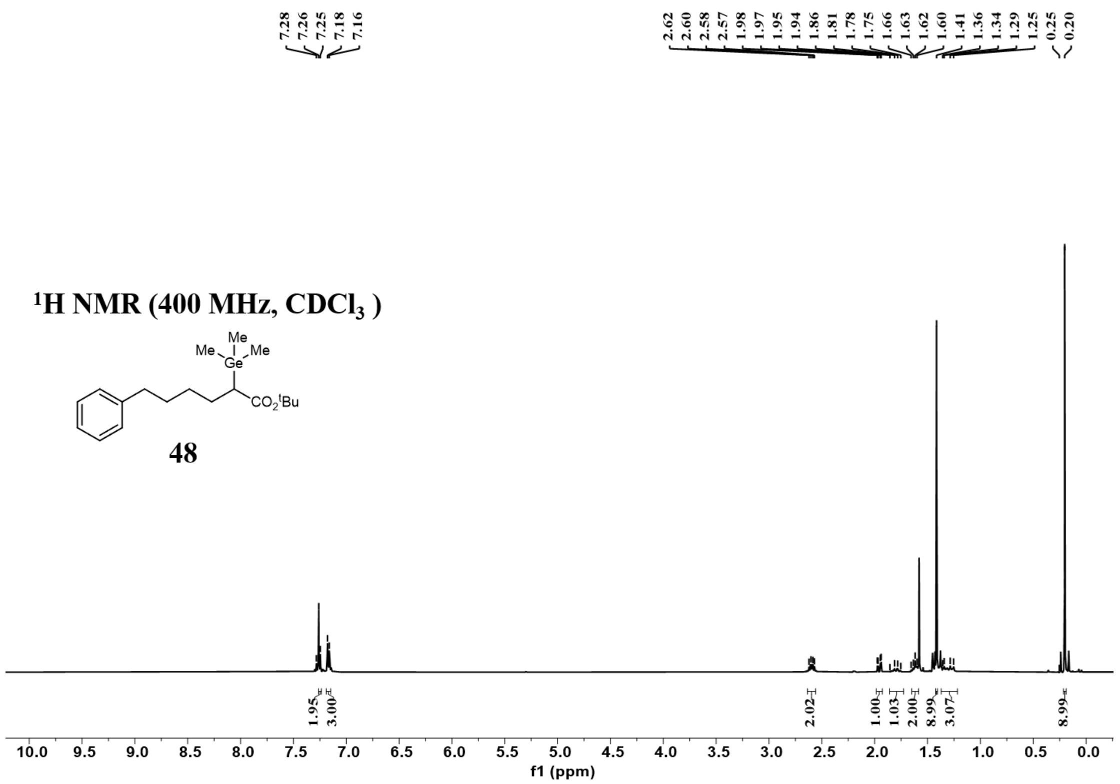

Supplementary Fig. 112. ^{1}H NMR of compound 48. The sample has been recorded in 400 MHz, $CDCl_3$ at 25 ºC

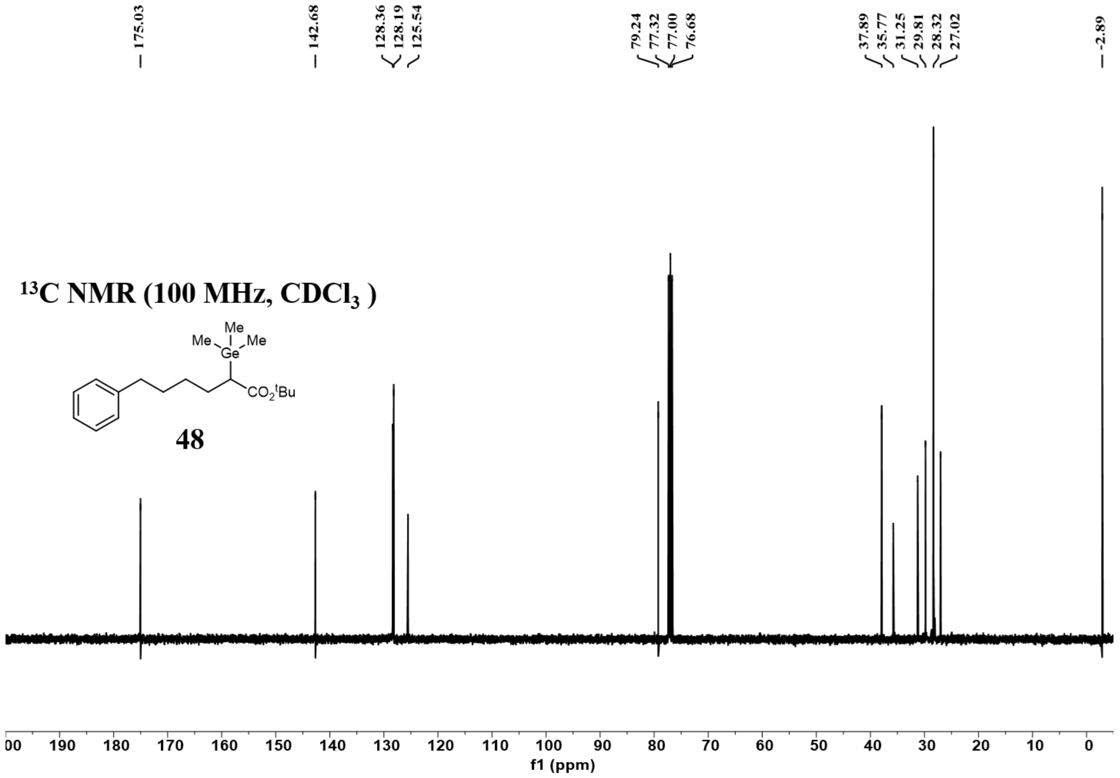

Supplementary Fig. 113. ^{13}C NMR of compound 48. The sample has been recorded in 100 MHz, $CDCl_3$ at 25 ºC.

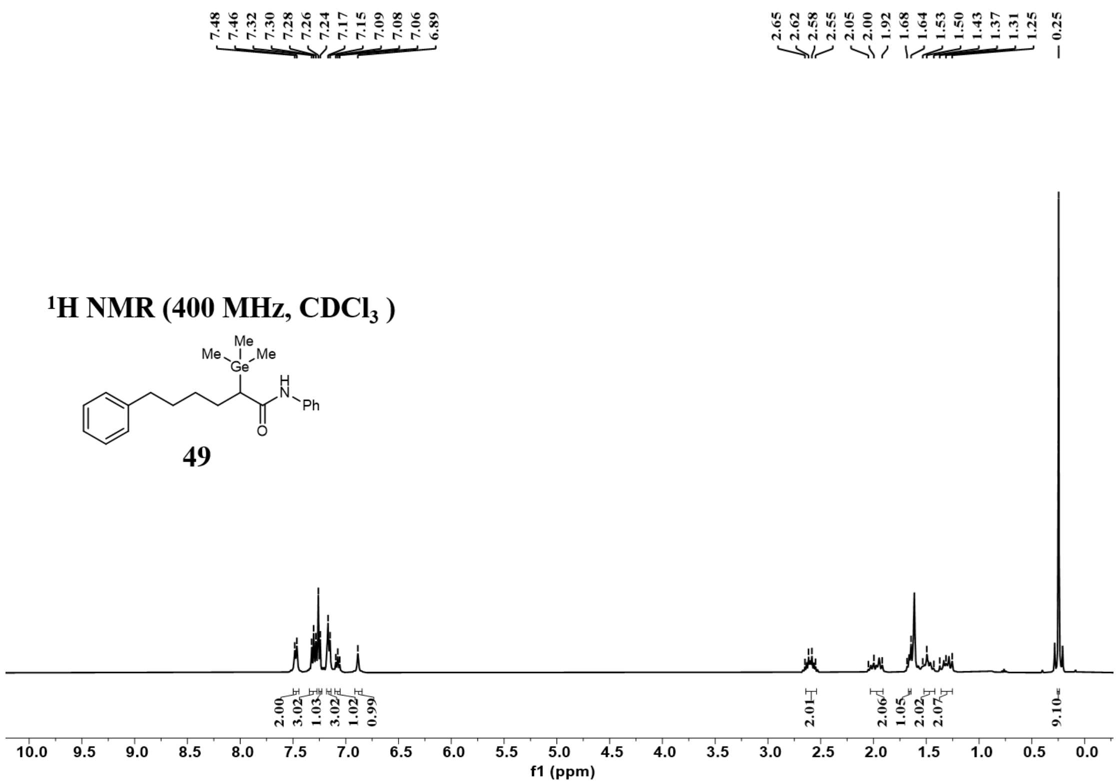

Supplementary Fig. 114. ^{1}H NMR of compound 49. The sample has been recorded in 400 MHz, $CDCl_3$ at 25 ºC

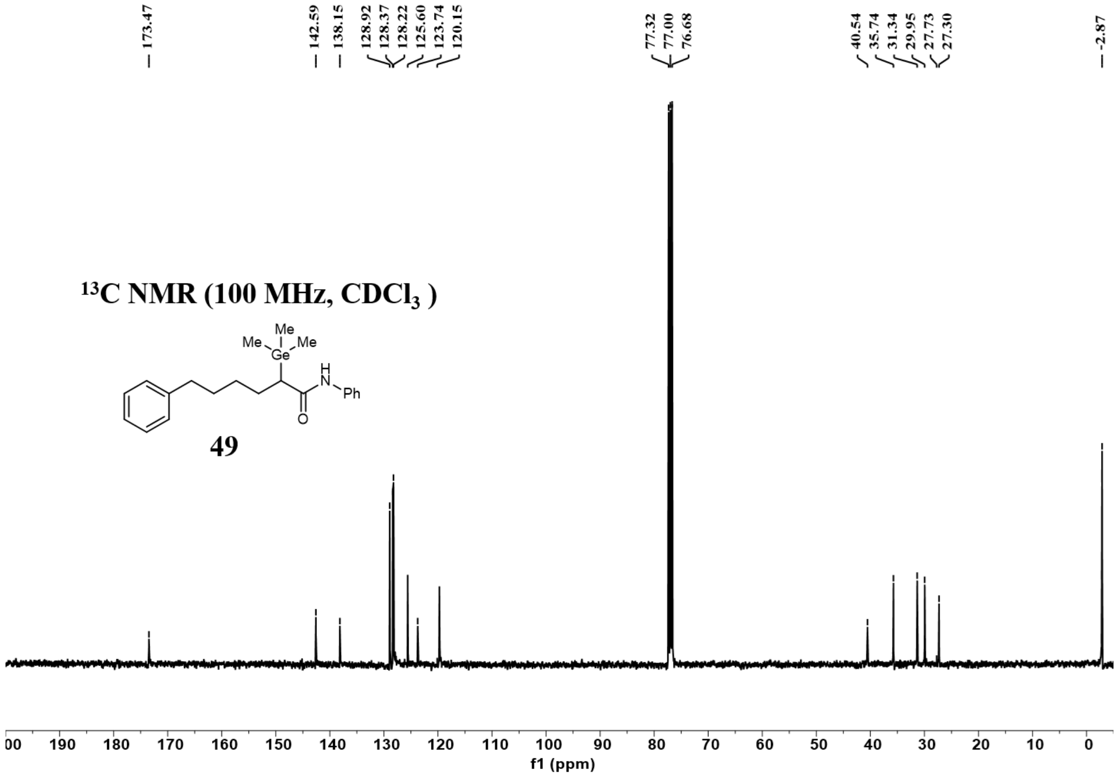

Supplementary Fig. 115. ^{13}C NMR of compound 49. The sample has been recorded in 100 MHz, $CDCl_3$ at 25 ºC.

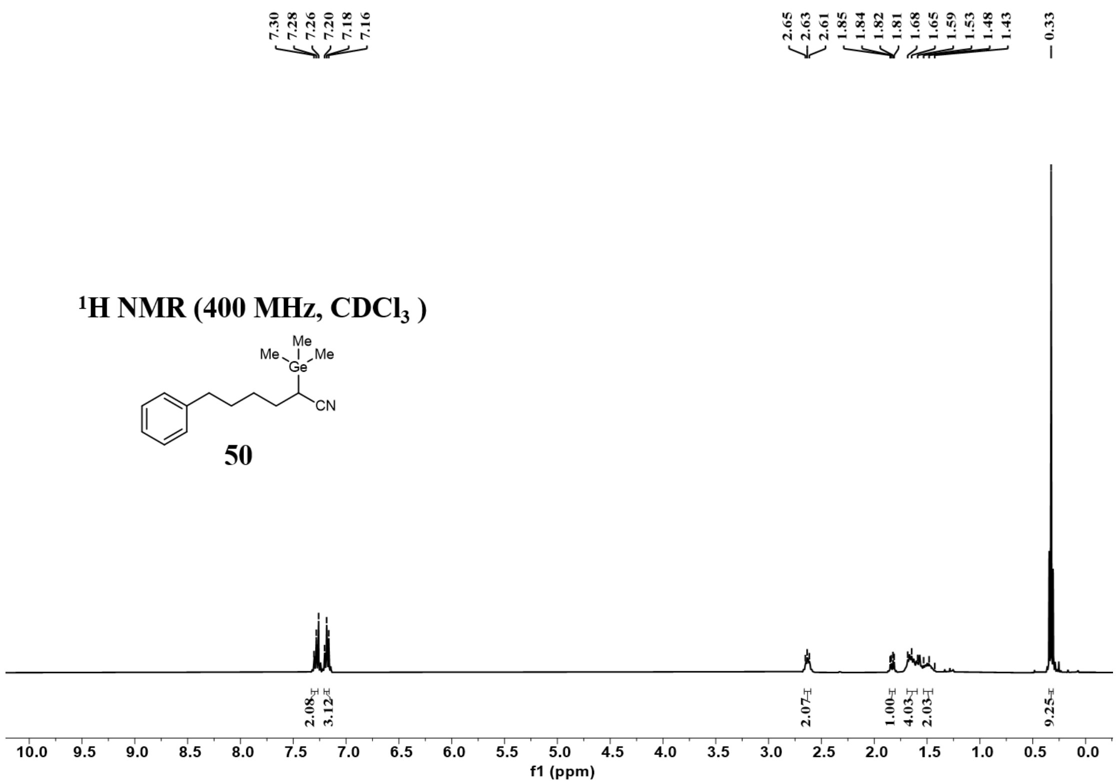

Supplementary Fig. 116. **^{1}H NMR of compound 50.** The sample has been recorded in 400 MHz, $CDCl_3$ at 25 ºC

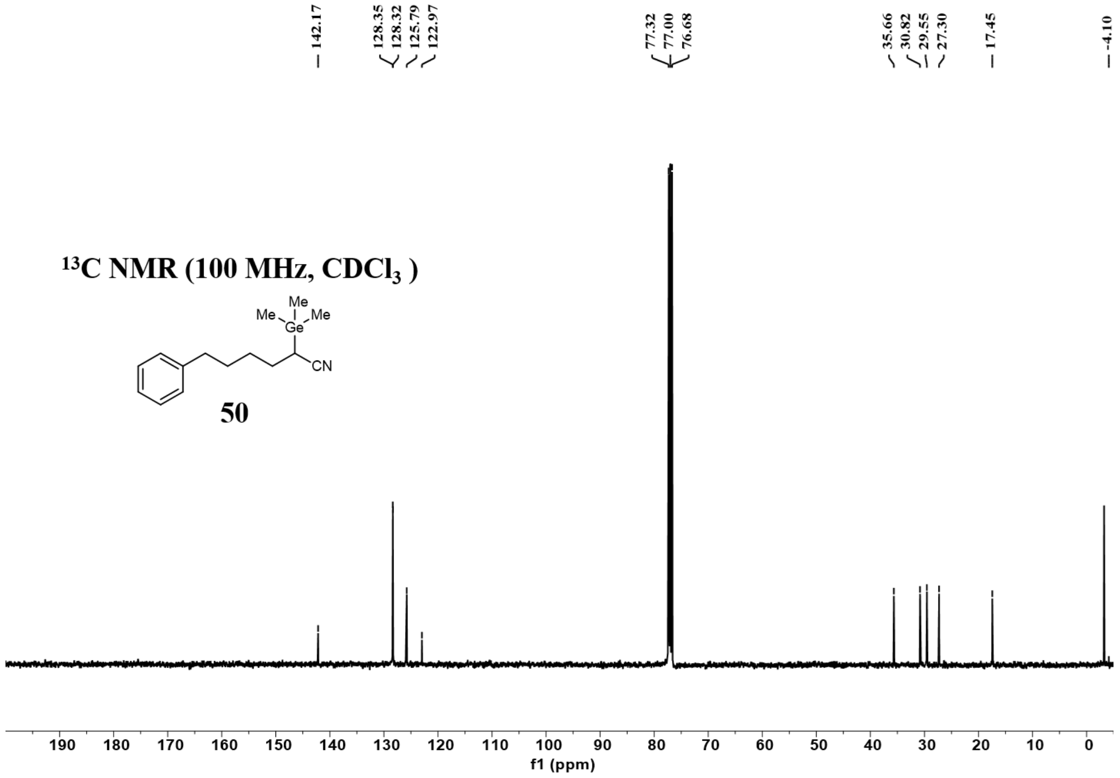

Supplementary Fig. 117. ^{13}C NMR of compound 50. The sample has been recorded in 100 MHz, $CDCl_3$ at 25 ºC.

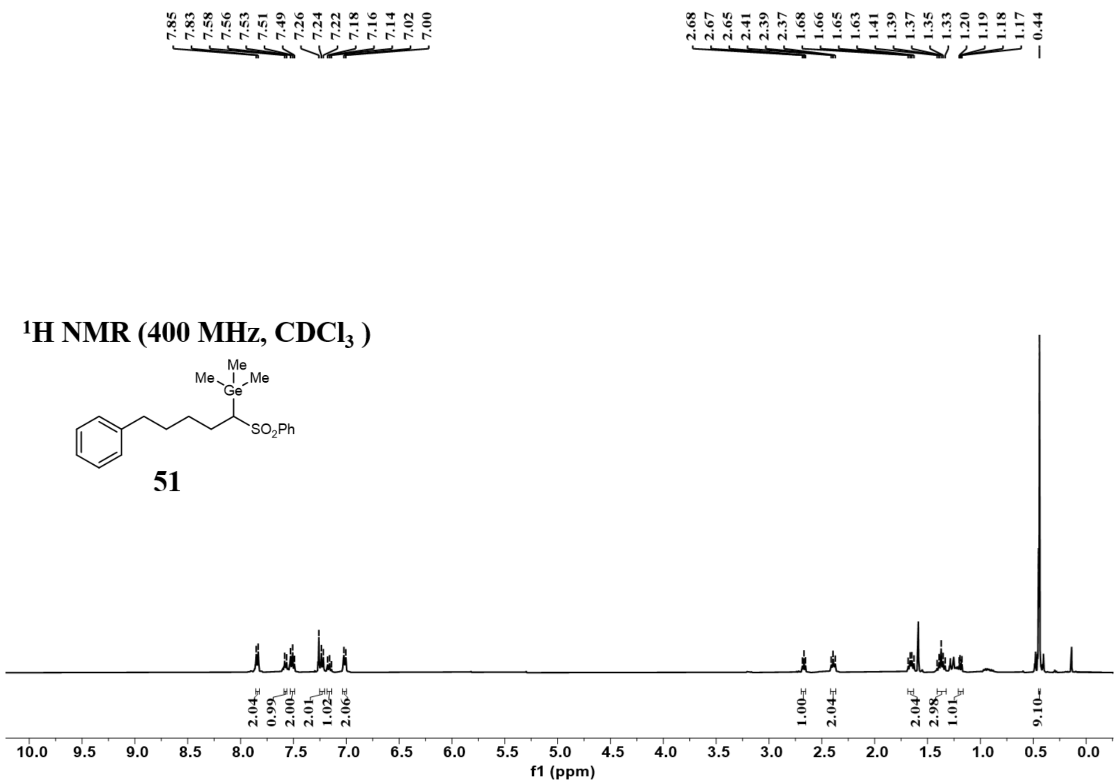

Supplementary Fig. 118. ^{1}H NMR of compound 51. The sample has been recorded in 400 MHz, $CDCl_3$ at 25 ºC

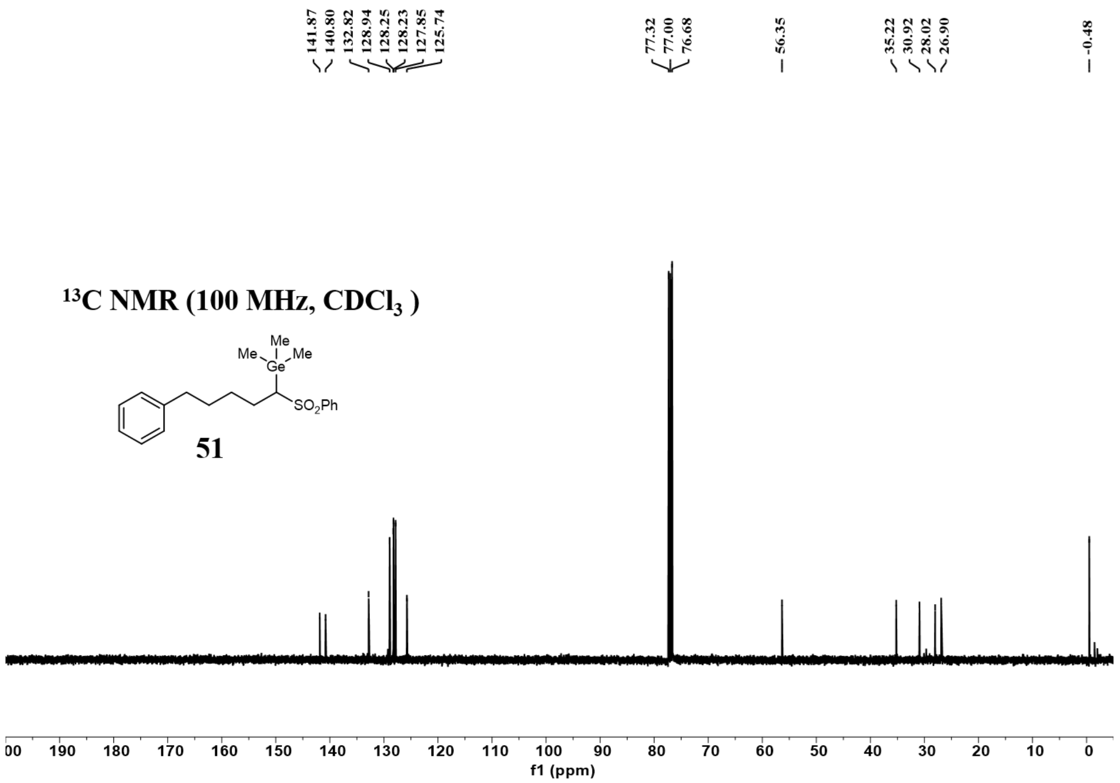

Supplementary Fig. 119. ^{13}C NMR of compound 51. The sample has been recorded in 100 MHz, $CDCl_3$ at 25 ºC.

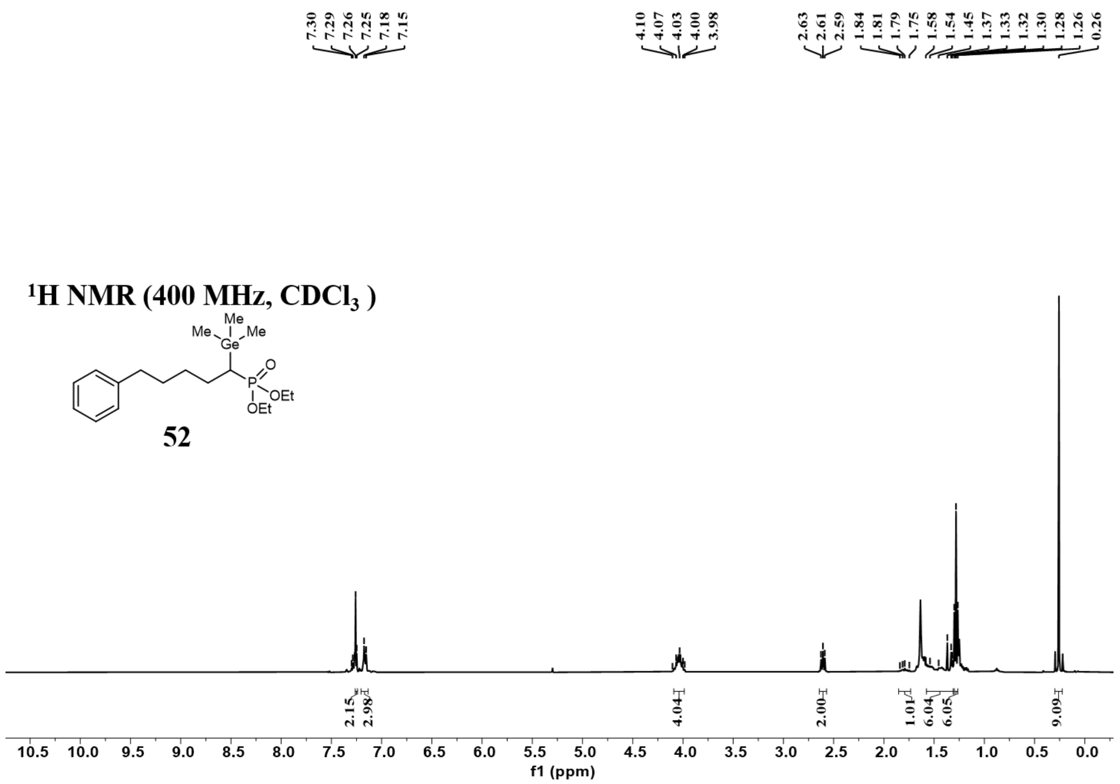

Supplementary Fig. 120. ^{1}H NMR of compound 52. The sample has been recorded in 400 MHz, $CDCl_3$ at 25 ºC

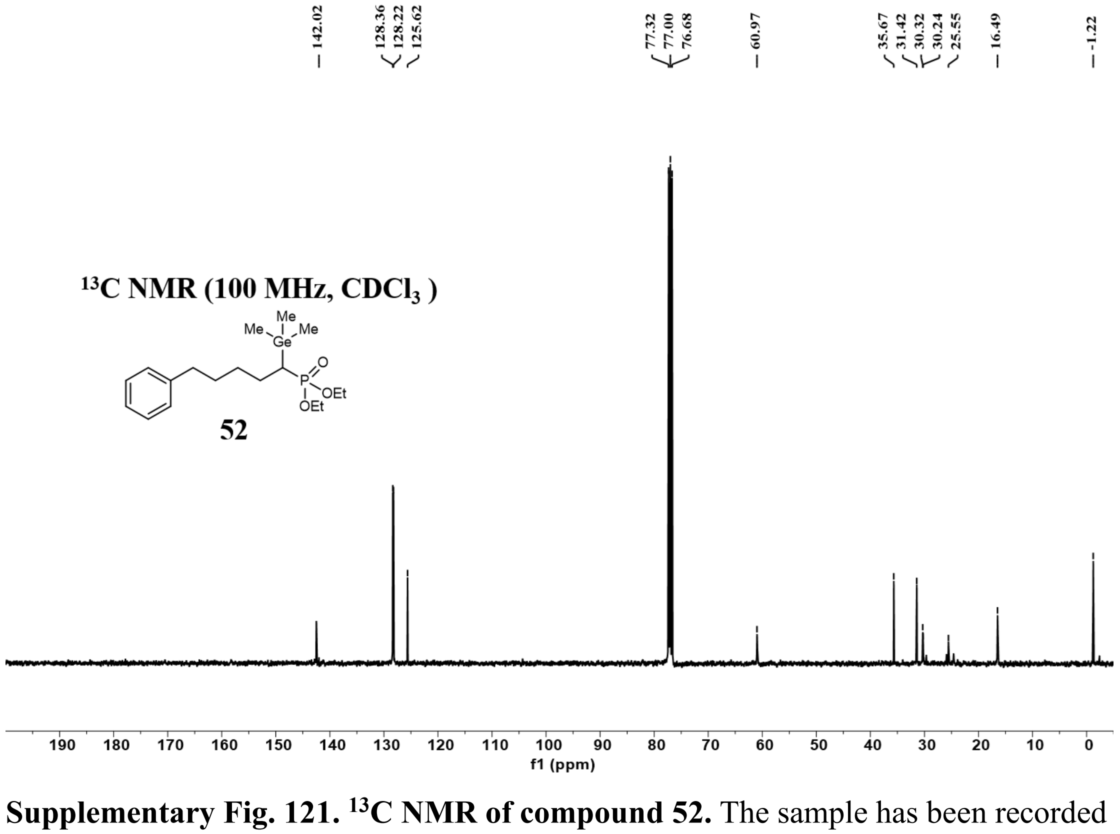

Supplementary Fig. 121. ^{13}C NMR of compound 52. The sample has been recorded in 100 MHz, $CDCl_3$ at 25 ºC.

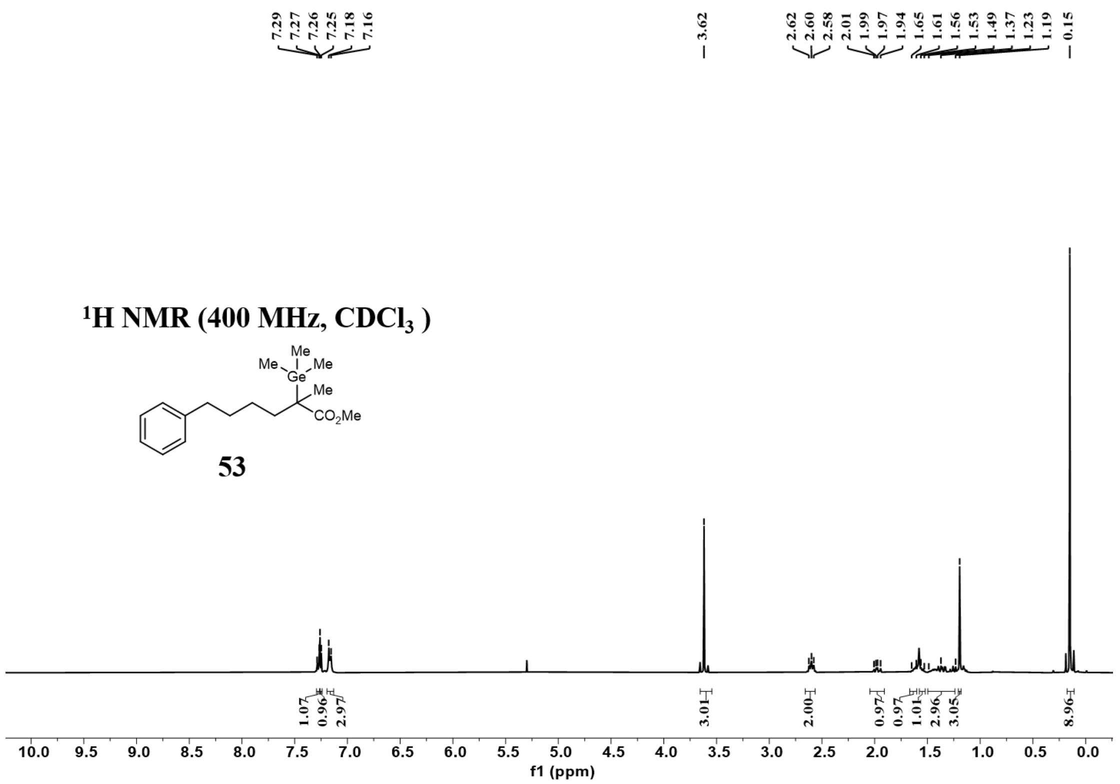

Supplementary Fig. 122. ^{1}H NMR of compound 53. The sample has been recorded in 400 MHz, $CDCl_3$ at 25 ºC

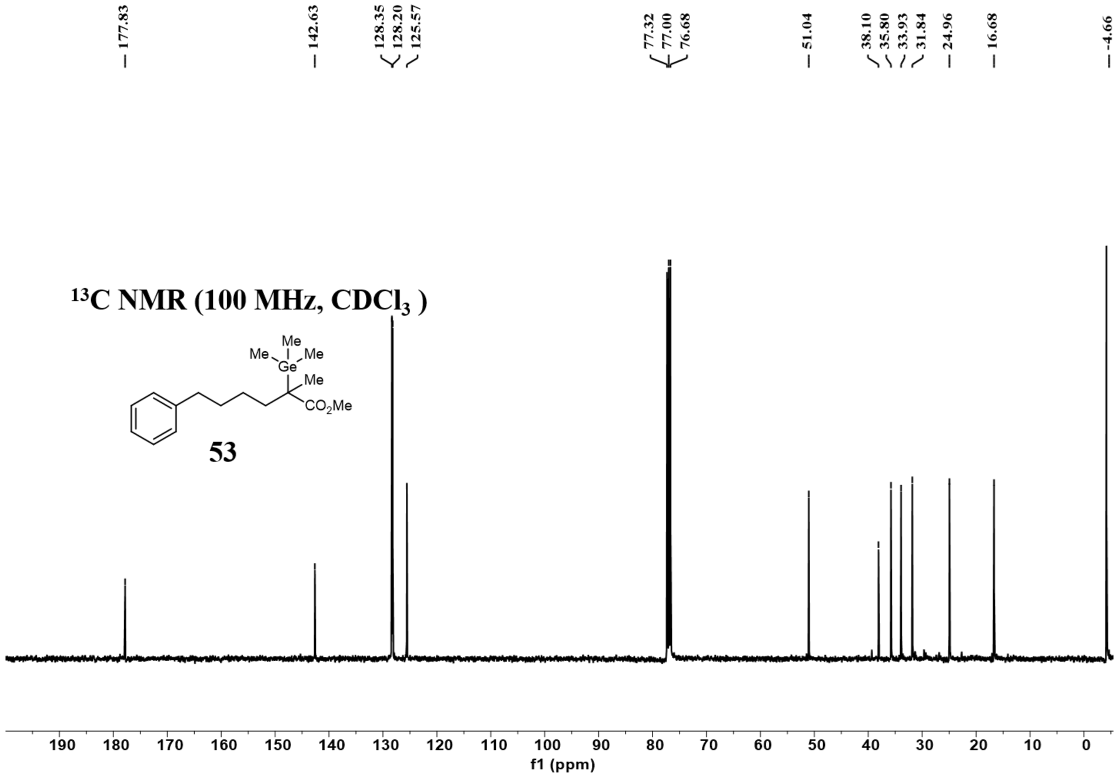

Supplementary Fig. 123. ^{13}C NMR of compound 53. The sample has been recorded in 100 MHz, $CDCl_3$ at 25 ºC.

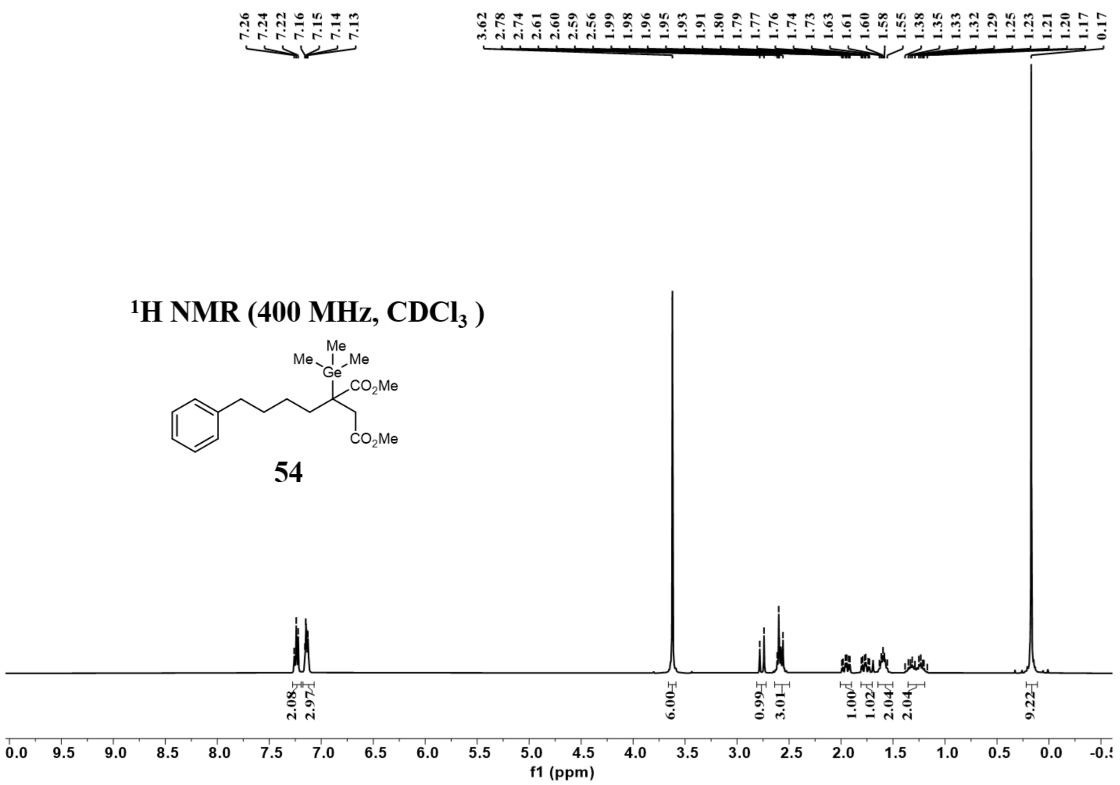

Supplementary Fig. 124. ^{1}H NMR of compound 54. The sample has been recorded in 400 MHz, $CDCl_3$ at 25 ºC

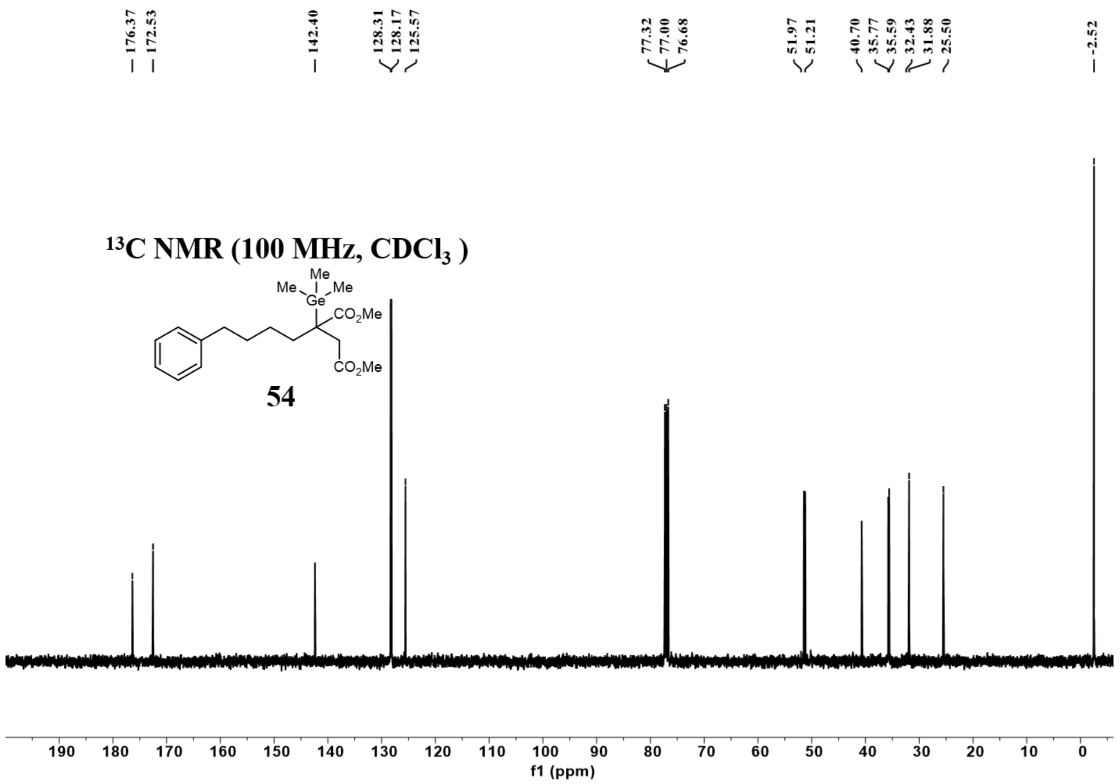

Supplementary Fig. 125. ^{13}C NMR of compound 54. The sample has been recorded in 100 MHz, $CDCl_3$ at 25 ºC.

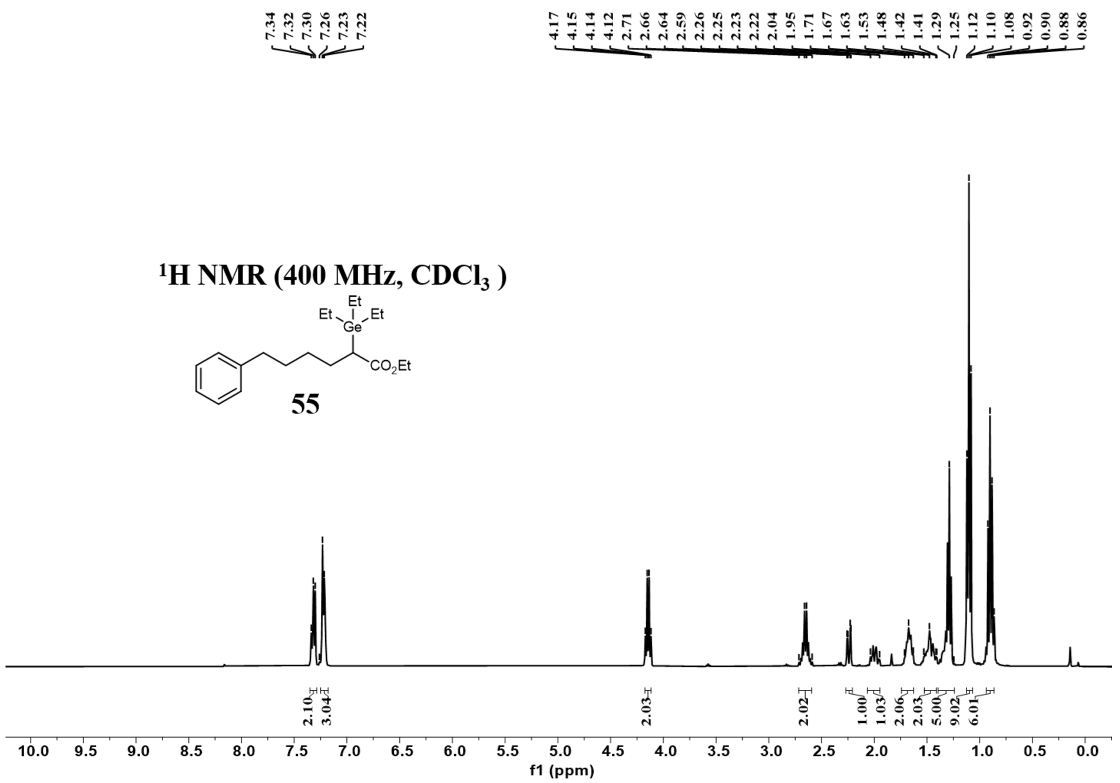

Supplementary Fig. 126. ^{1}H NMR of compound 55. The sample has been recorded in 400 MHz, $CDCl_3$ at 25 ºC

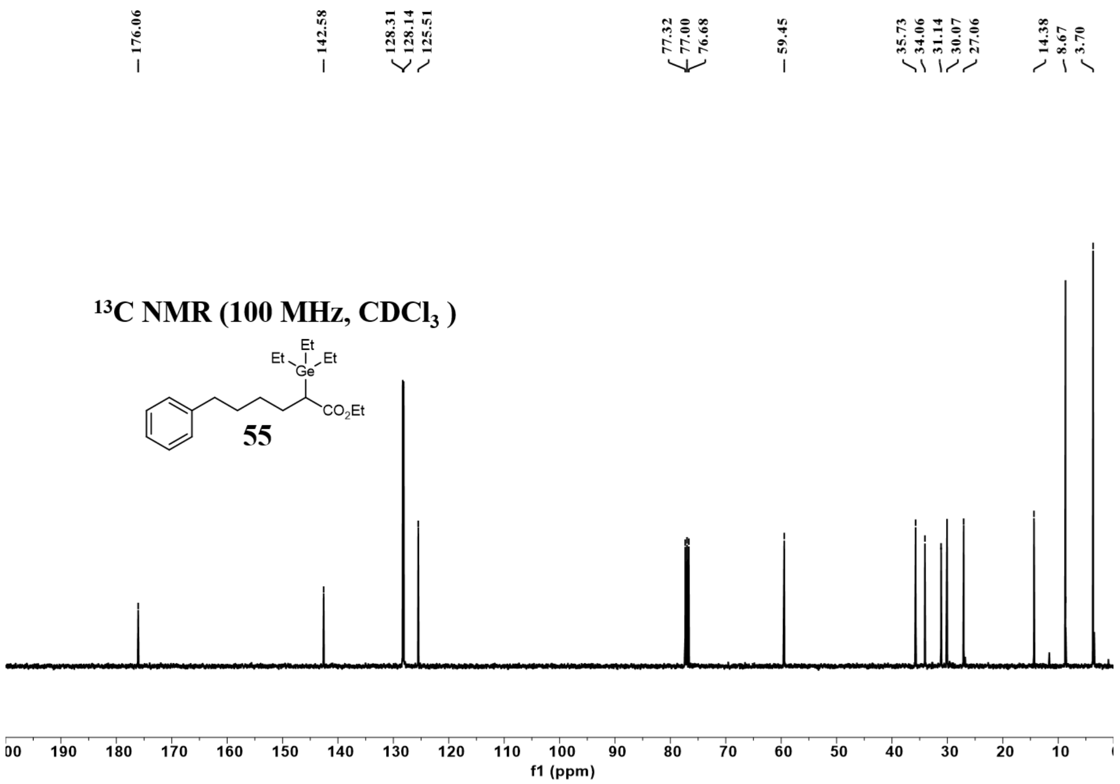

Supplementary Fig. 127. ^{13}C NMR of compound 55. The sample has been recorded in 100 MHz, $CDCl_3$ at 25 ºC.

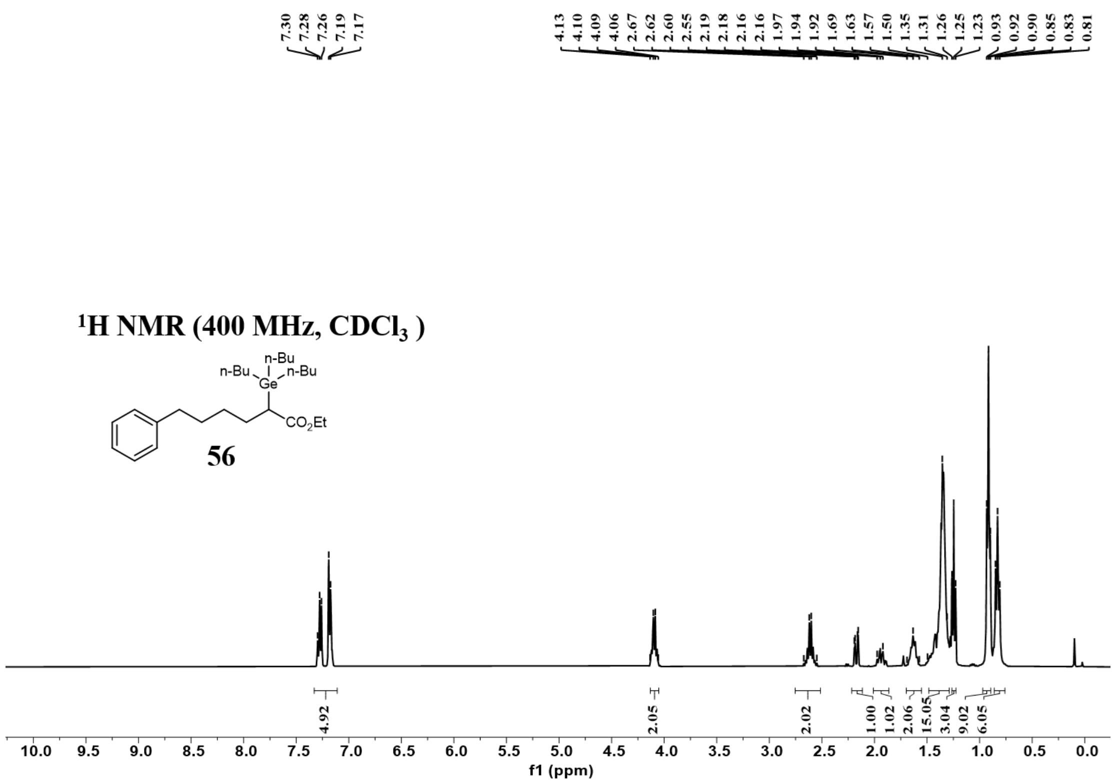

Supplementary Fig. 128. ^{1}H NMR of compound 56. The sample has been recorded in 400 MHz, $CDCl_3$ at 25 ºC

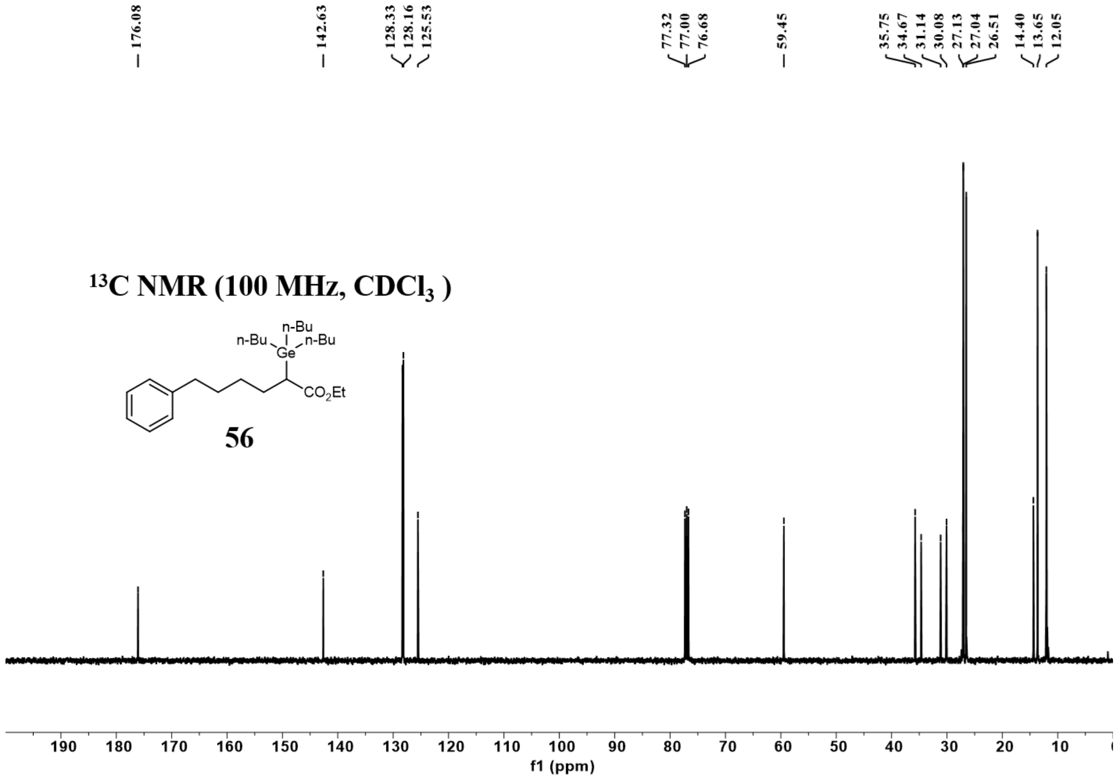

Supplementary Fig. 129. ^{13}C NMR of compound 56. The sample has been recorded in 100 MHz, $CDCl_3$ at 25 ºC.

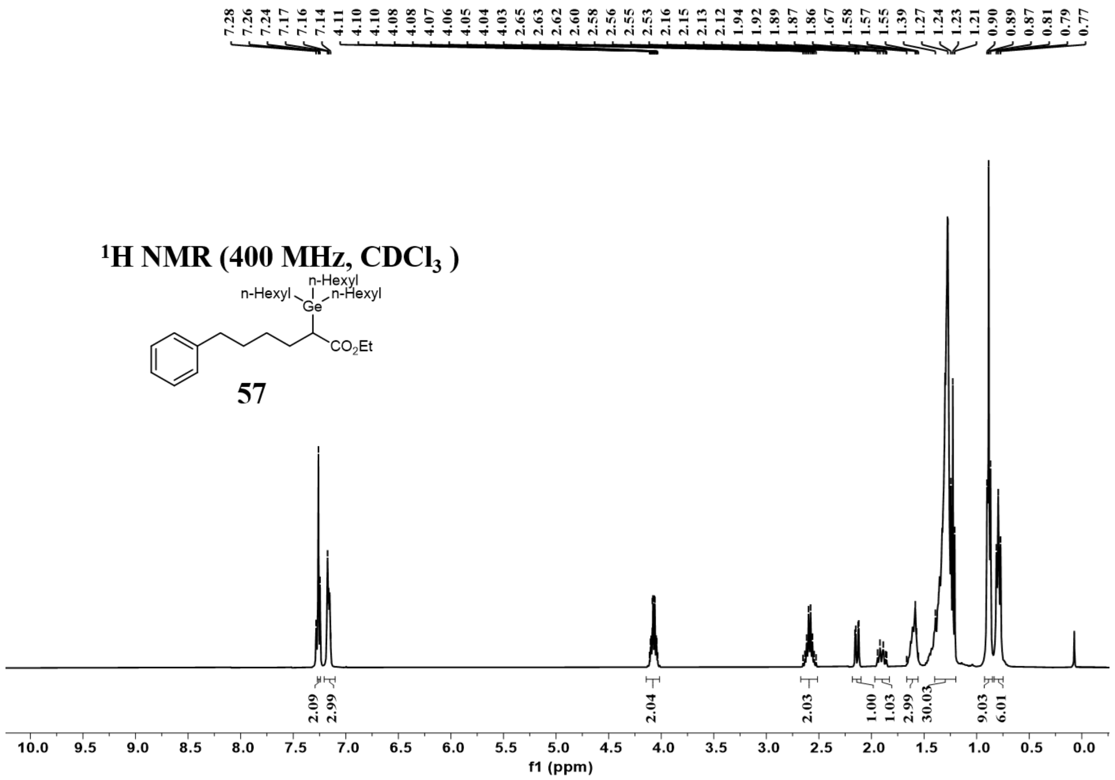

Supplementary Fig. 130. ^{1}H NMR of compound 57. The sample has been recorded in 400 MHz, $CDCl_3$ at 25 ºC

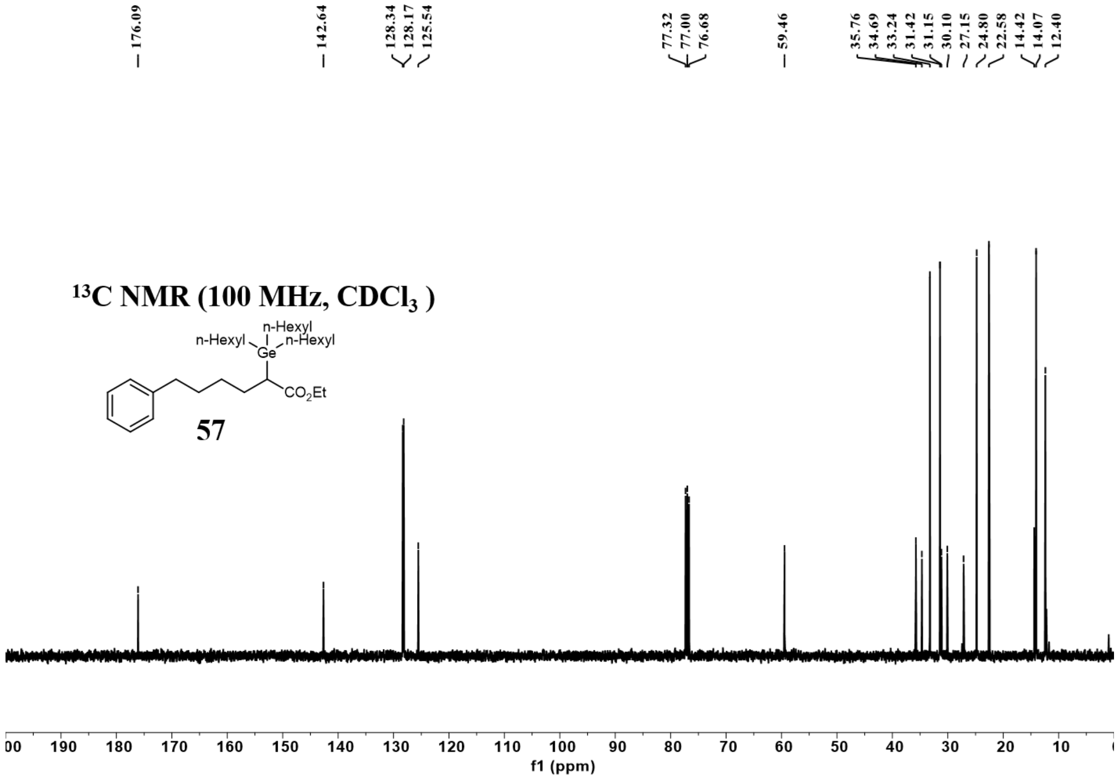

Supplementary Fig. 131. ^{13}C NMR of compound 57. The sample has been recorded in 100 MHz, $CDCl_3$ at 25 ºC.

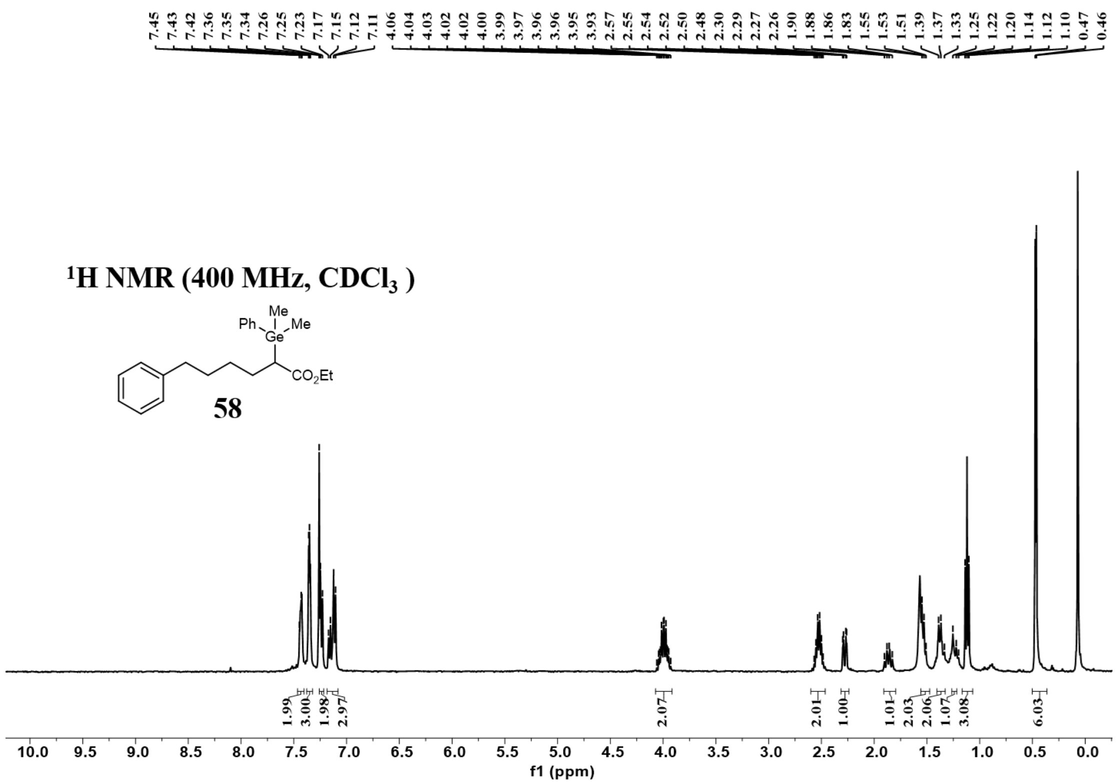

Supplementary Fig. 132. ^{1}H NMR of compound 58. The sample has been recorded in 400 MHz, $CDCl_3$ at 25 ºC

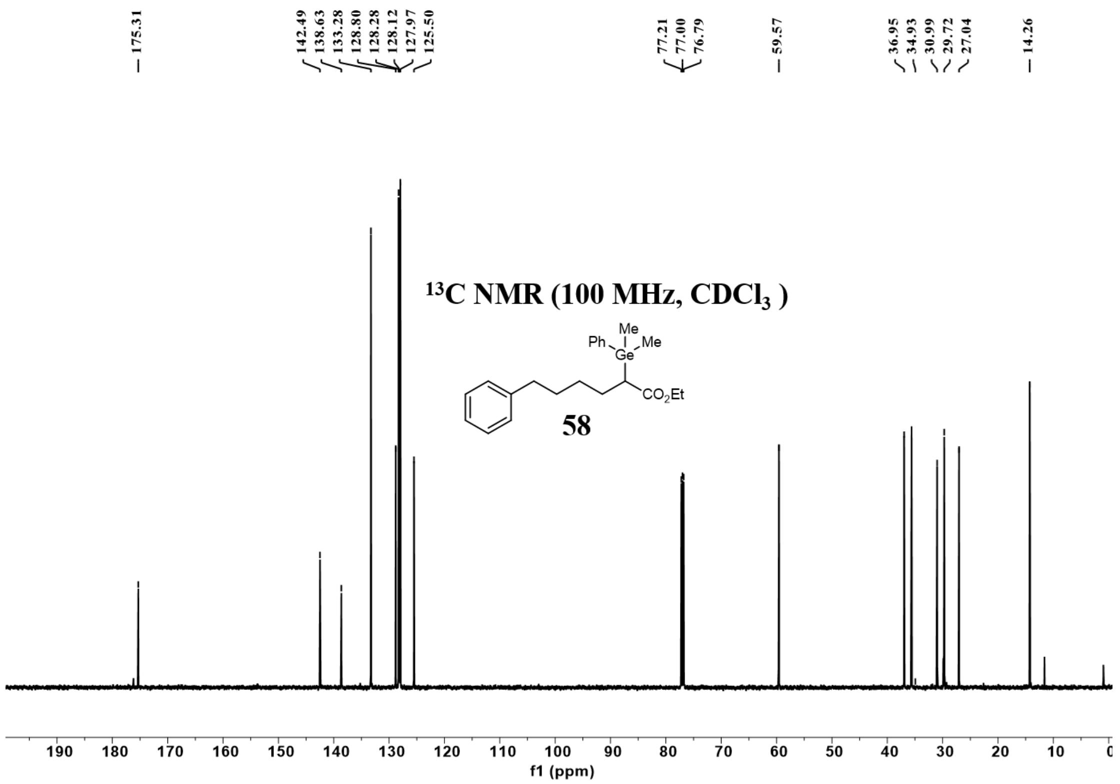

Supplementary Fig. 133. ^{13}C NMR of compound 58. The sample has been recorded in 100 MHz, $CDCl_3$ at 25 ºC.

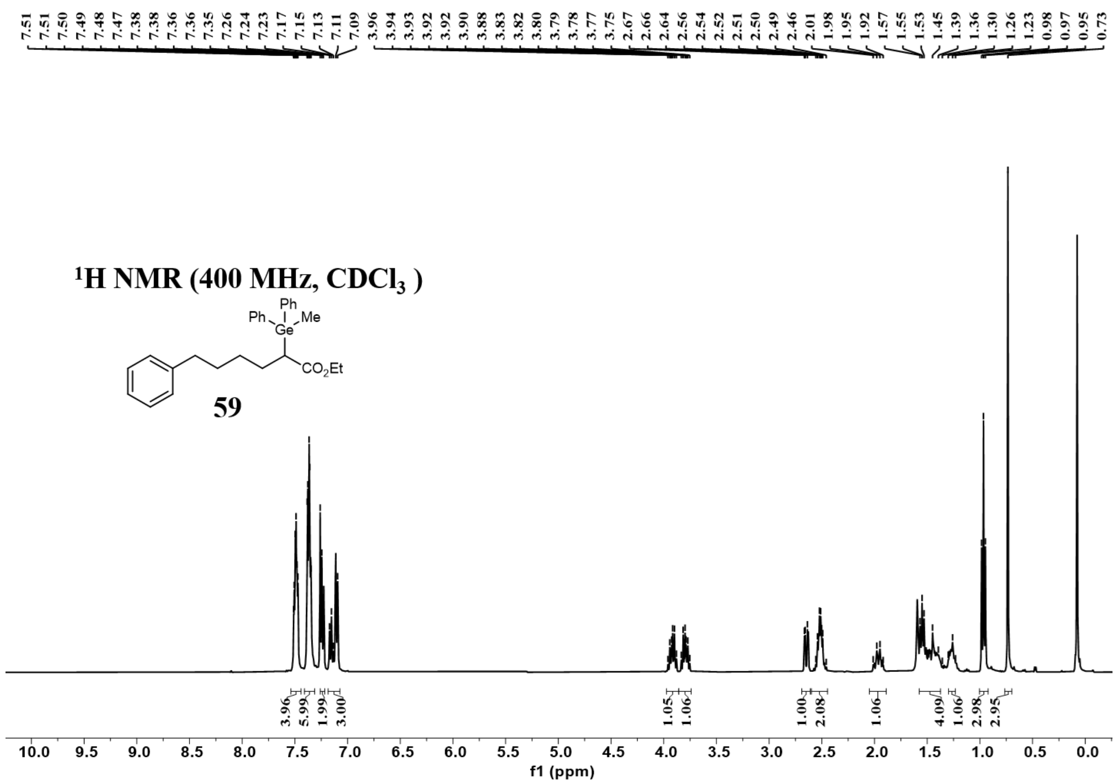

Supplementary Fig. 134. ^{1}H NMR of compound 59. The sample has been recorded in 400 MHz, $CDCl_3$ at 25 ºC

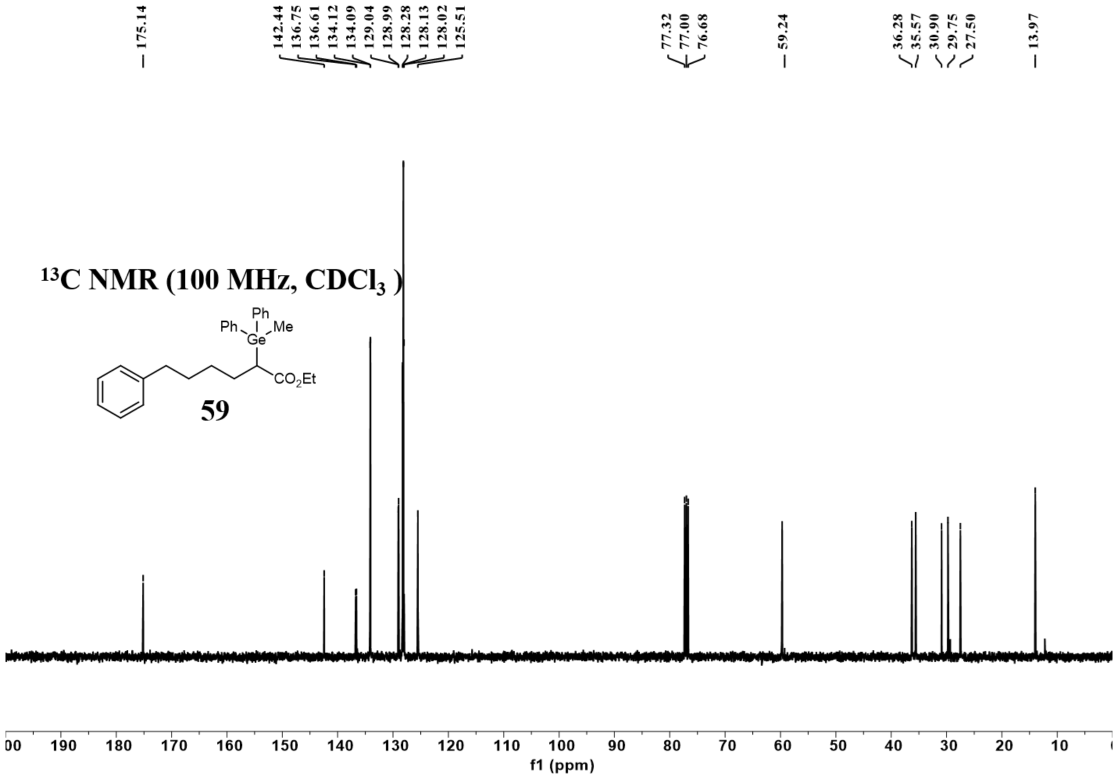

Supplementary Fig. 135. ^{13}C NMR of compound 59. The sample has been recorded in 100 MHz, $CDCl_3$ at 25 ºC.

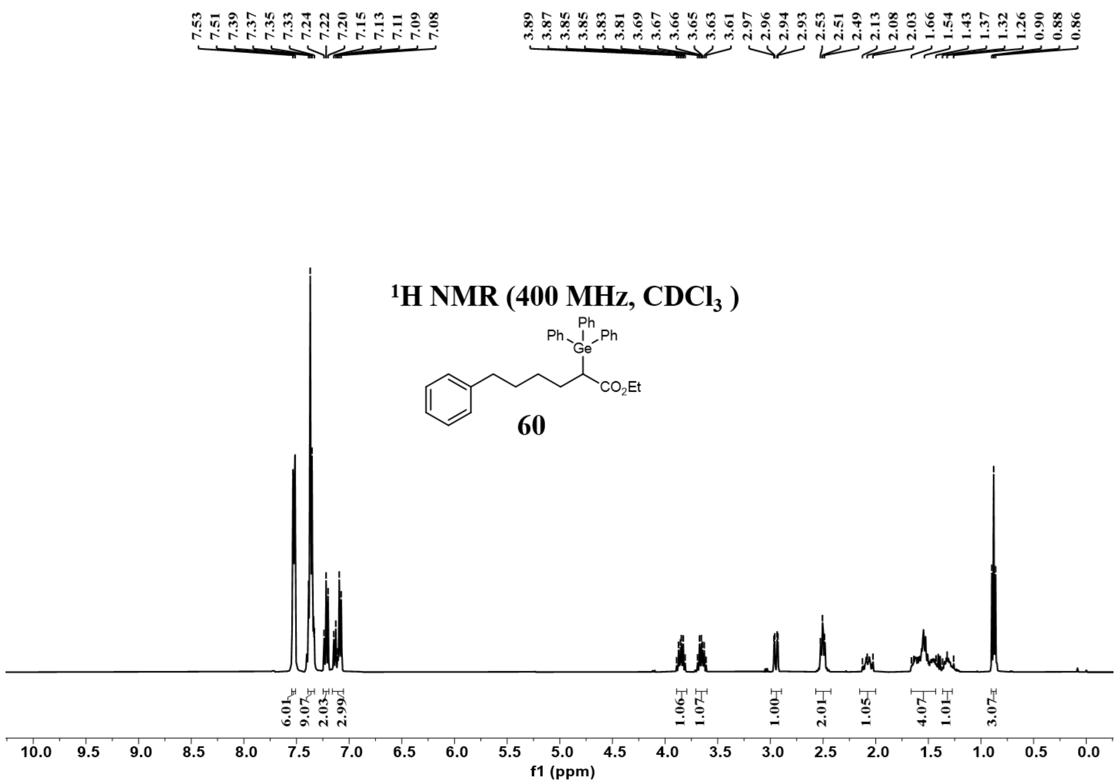

Supplementary Fig. 136. ^{1}H NMR of compound 60. The sample has been recorded in 400 MHz, $CDCl_3$ at 25 ºC

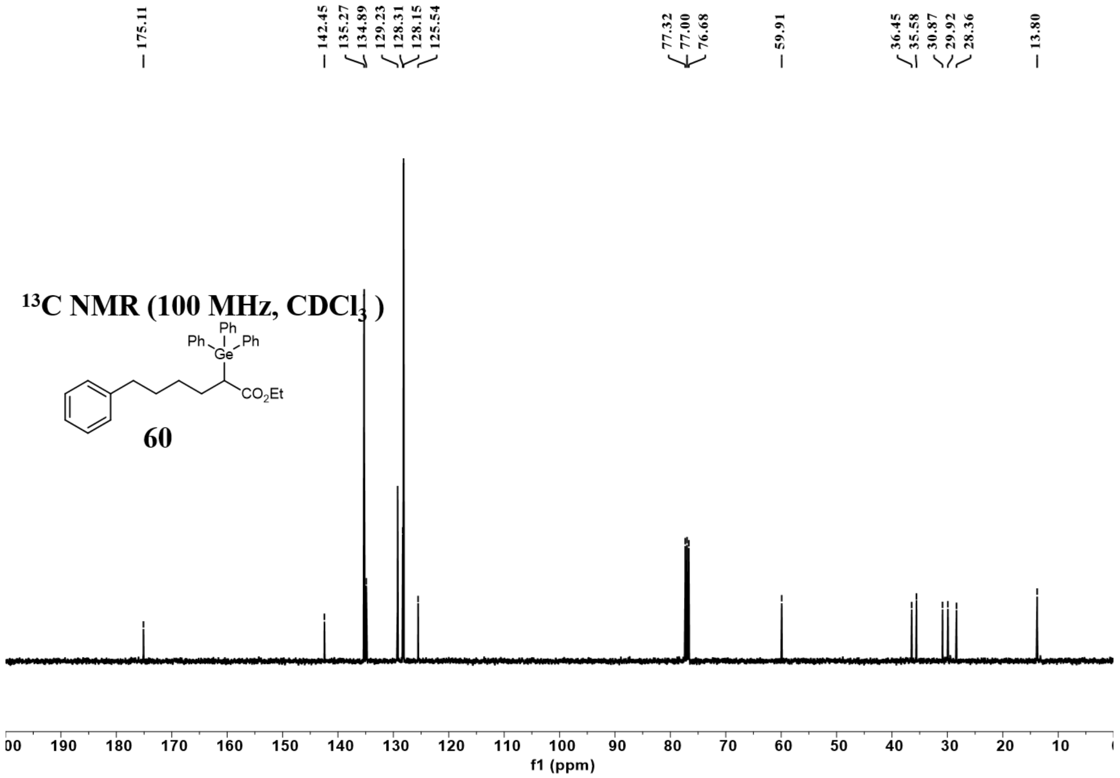

Supplementary Fig. 137. ^{13}C NMR of compound 60. The sample has been recorded in 100 MHz, $CDCl_3$ at 25 ºC.

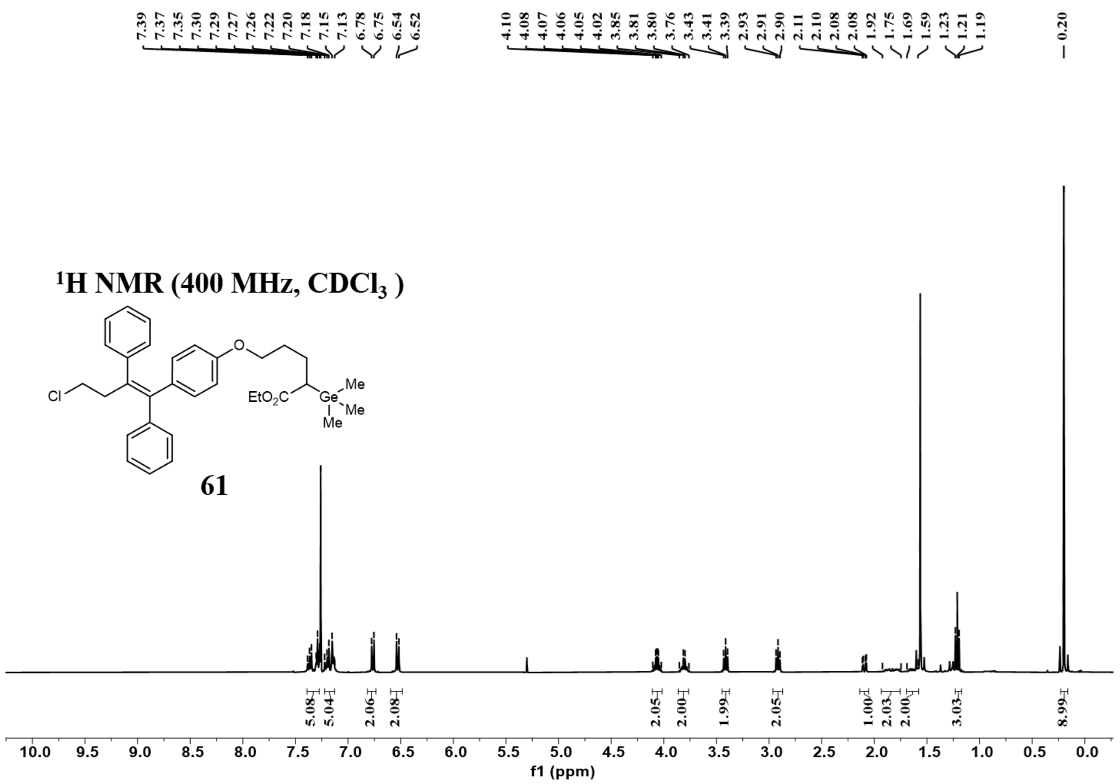

Supplementary Fig. 138. ^{1}H NMR of compound 61. The sample has been recorded in 400 MHz, $CDCl_3$ at 25 ºC

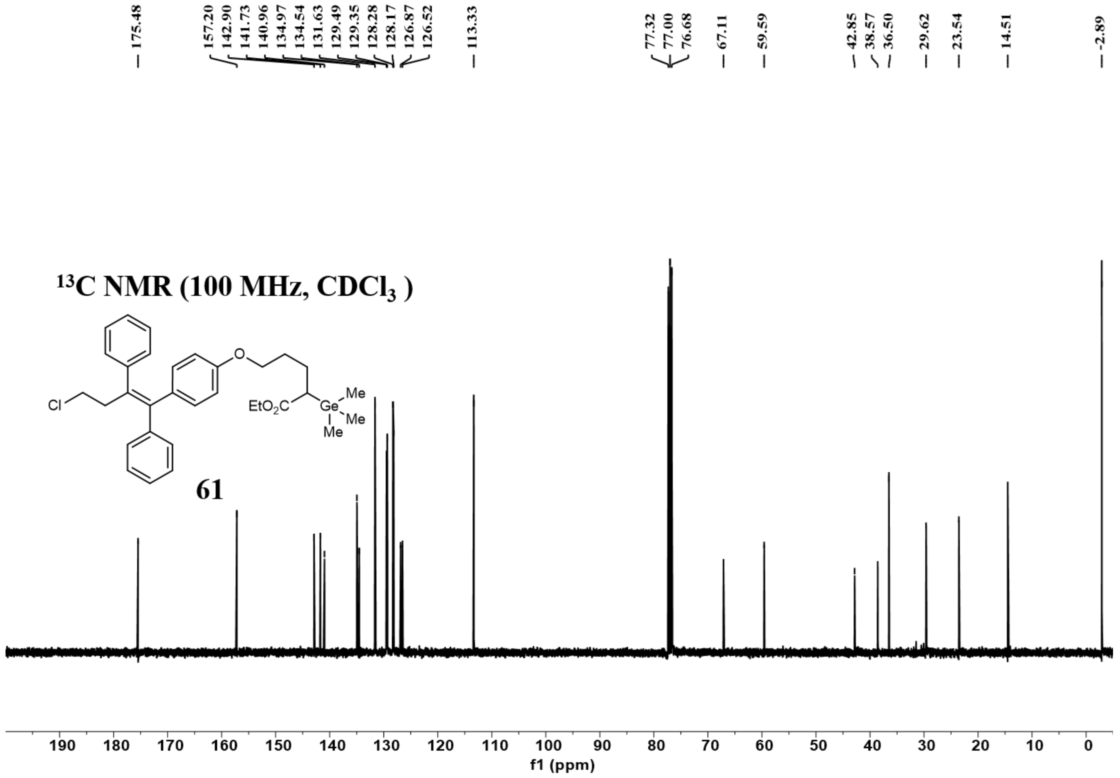

Supplementary Fig. 139. ^{13}C NMR of compound 61. The sample has been recorded in 100 MHz, $CDCl_3$ at 25 ºC.

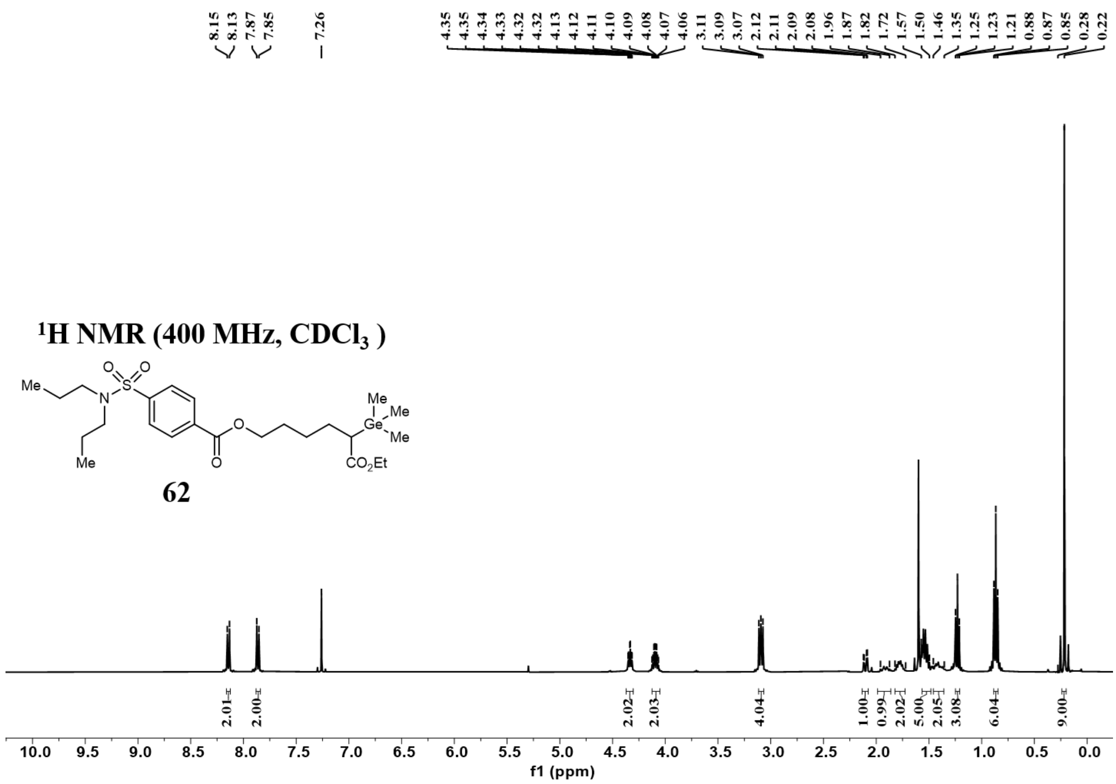

Supplementary Fig. 140. ^{1}H NMR of compound 62. The sample has been recorded in 400 MHz, $CDCl_3$ at 25 ºC

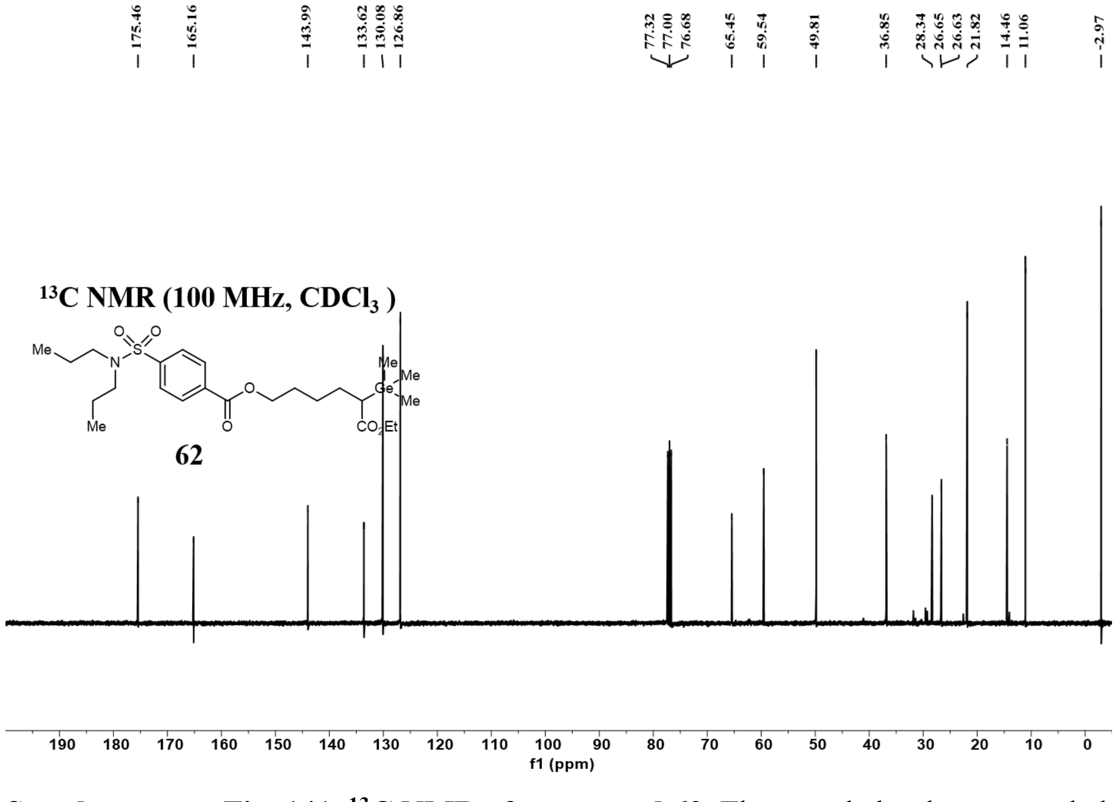

Supplementary Fig. 141. ^{13}C NMR of compound 62. The sample has been recorded in 100 MHz, $CDCl_3$ at 25 ºC.

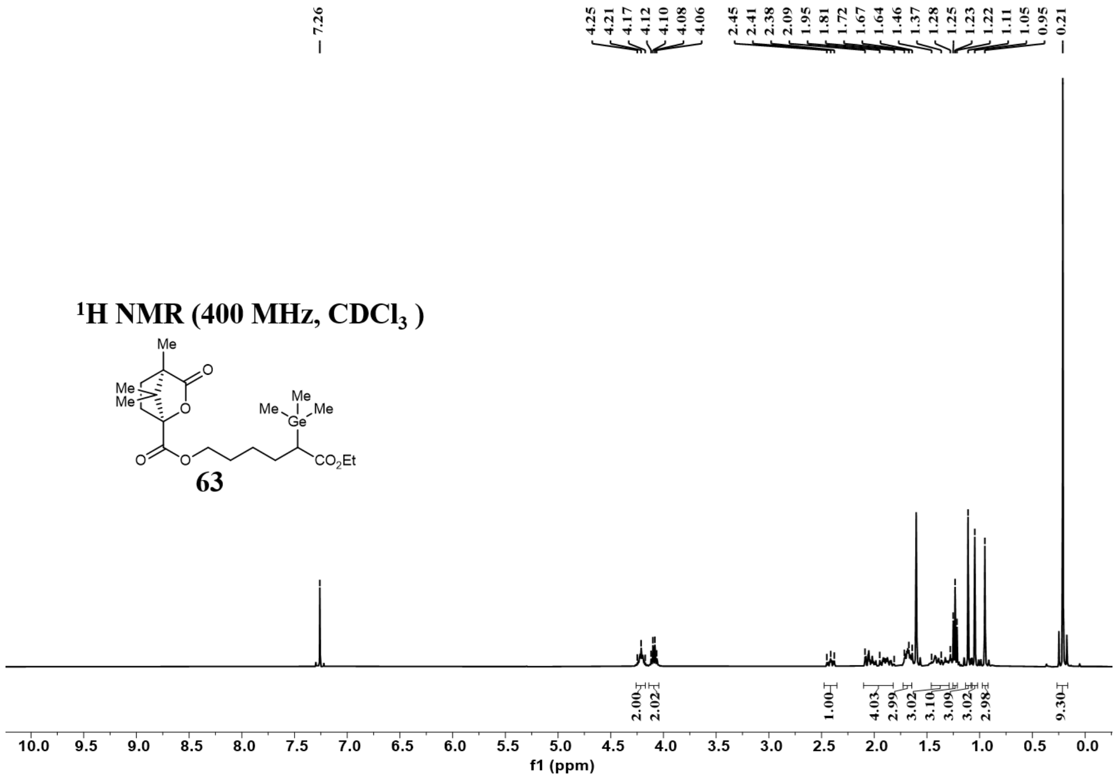

Supplementary Fig. 142. ^{1}H NMR of compound 63. The sample has been recorded in 400 MHz, $CDCl_3$ at 25 ºC

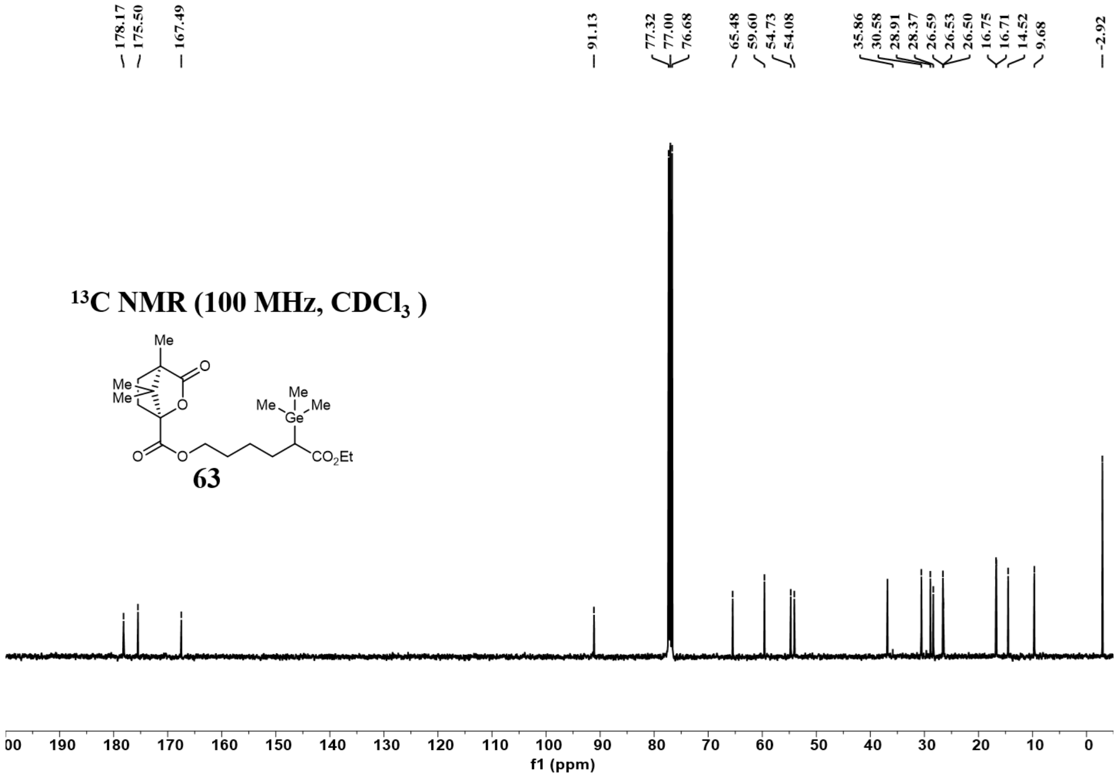

Supplementary Fig. 143. ^{13}C NMR of compound 63. The sample has been recorded in 100 MHz, $CDCl_3$ at 25 ºC.

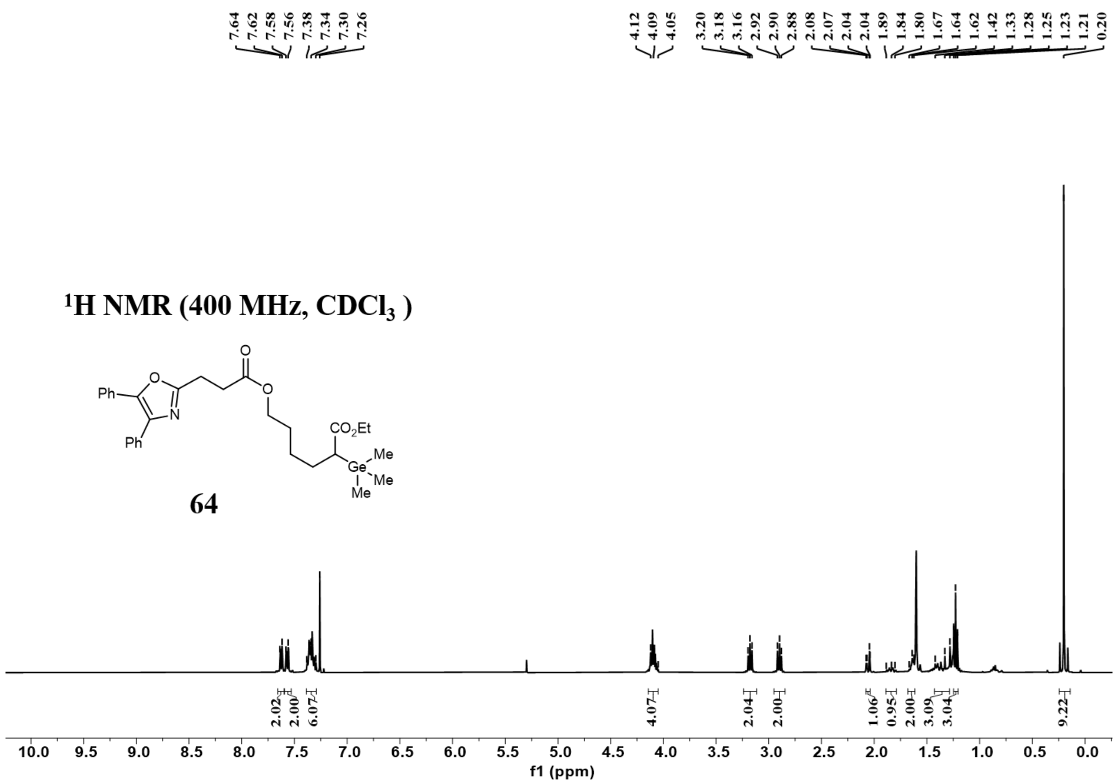

Supplementary Fig. 144. ^{1}H NMR of compound 64. The sample has been recorded in 400 MHz, $CDCl_3$ at 25 ºC

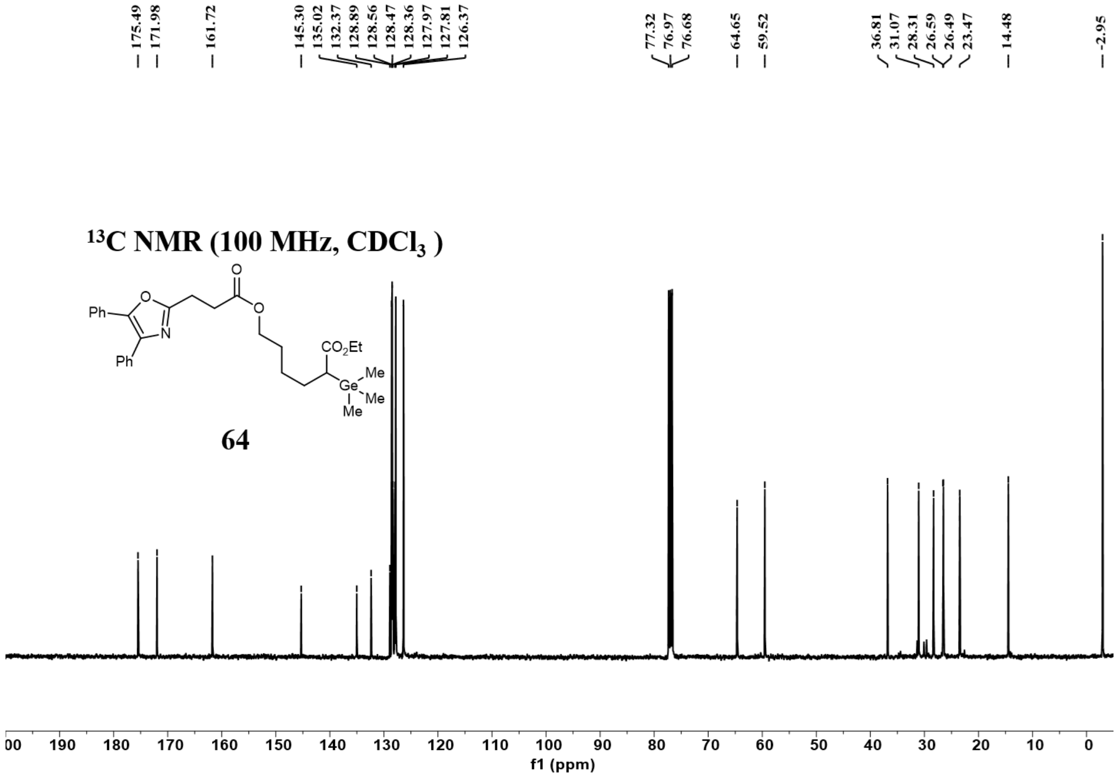

Supplementary Fig. 145. ^{13}C NMR of compound 64. The sample has been recorded in 100 MHz, $CDCl_3$ at 25 ºC.

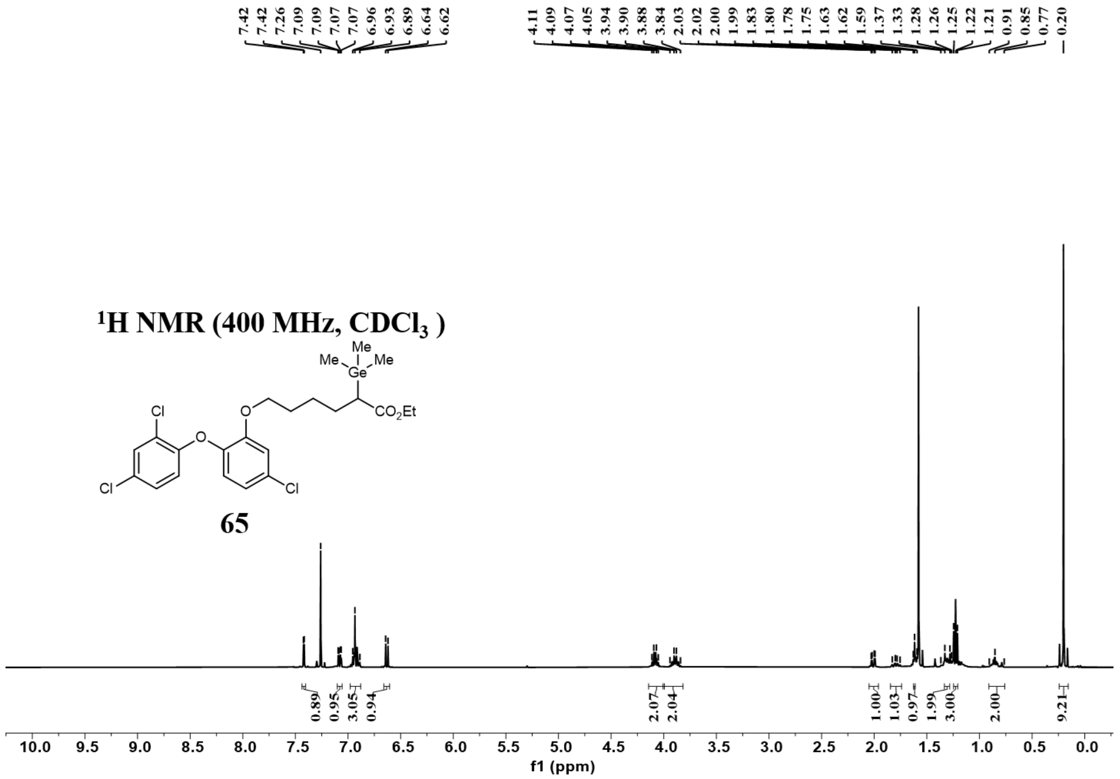

Supplementary Fig. 146. ^{1}H NMR of compound 65. The sample has been recorded in 400 MHz, $CDCl_3$ at 25 ºC

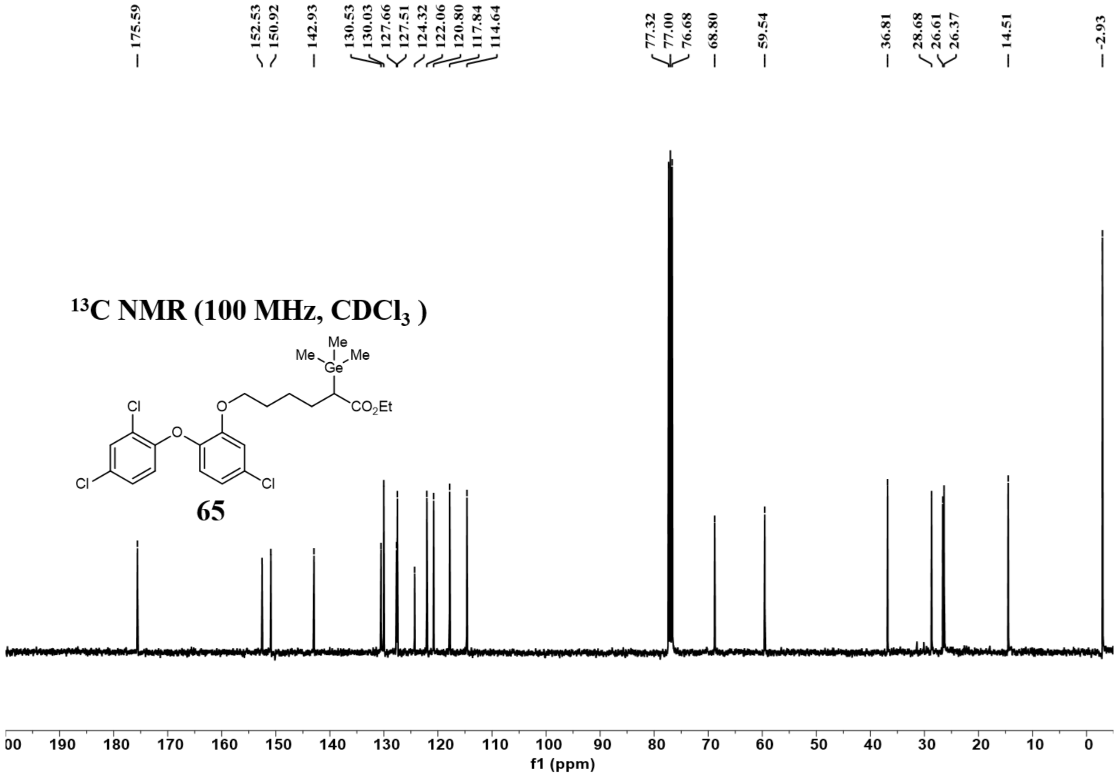

Supplementary Fig. 147. ^{13}C NMR of compound 65. The sample has been recorded in 100 MHz, $CDCl_3$ at 25 ºC.

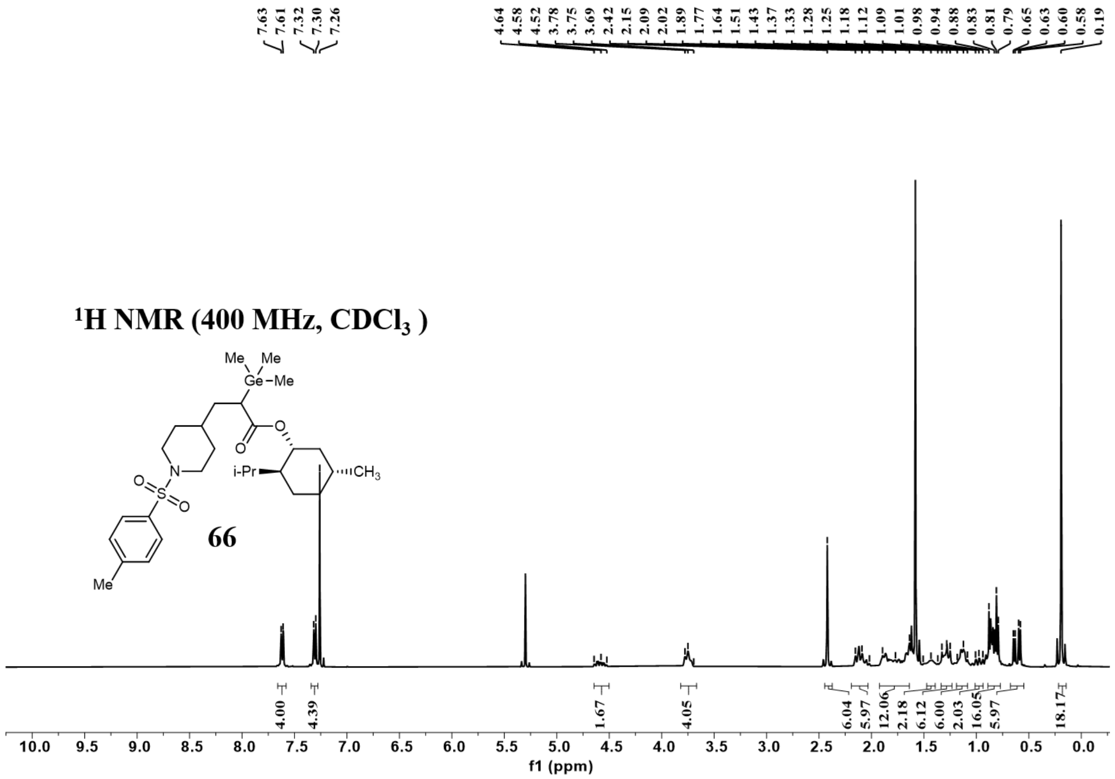

Supplementary Fig. 148. ^{1}H NMR of compound 66. The sample has been recorded in 400 MHz, $CDCl_3$ at 25 ºC

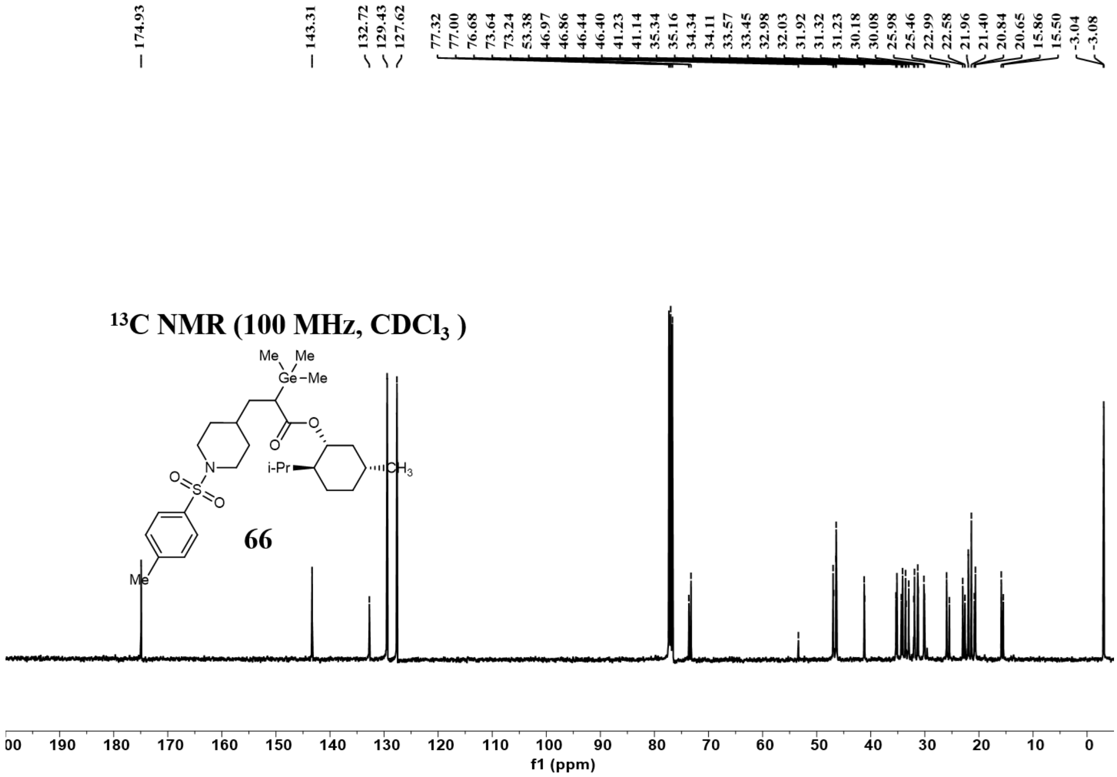

Supplementary Fig. 149. ^{13}C NMR of compound 66. The sample has been recorded in 100 MHz, $CDCl_3$ at 25 ºC.

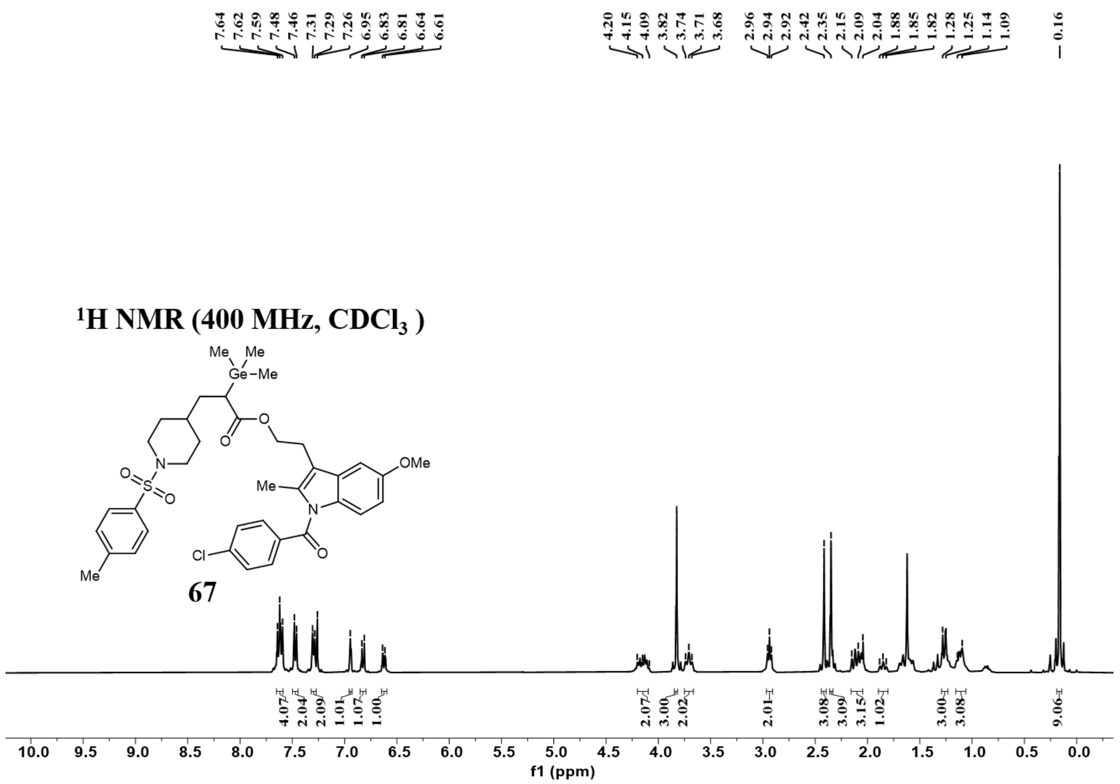

Supplementary Fig. 150. ^{1}H NMR of compound 67. The sample has been recorded in 400 MHz, $CDCl_3$ at 25 ºC

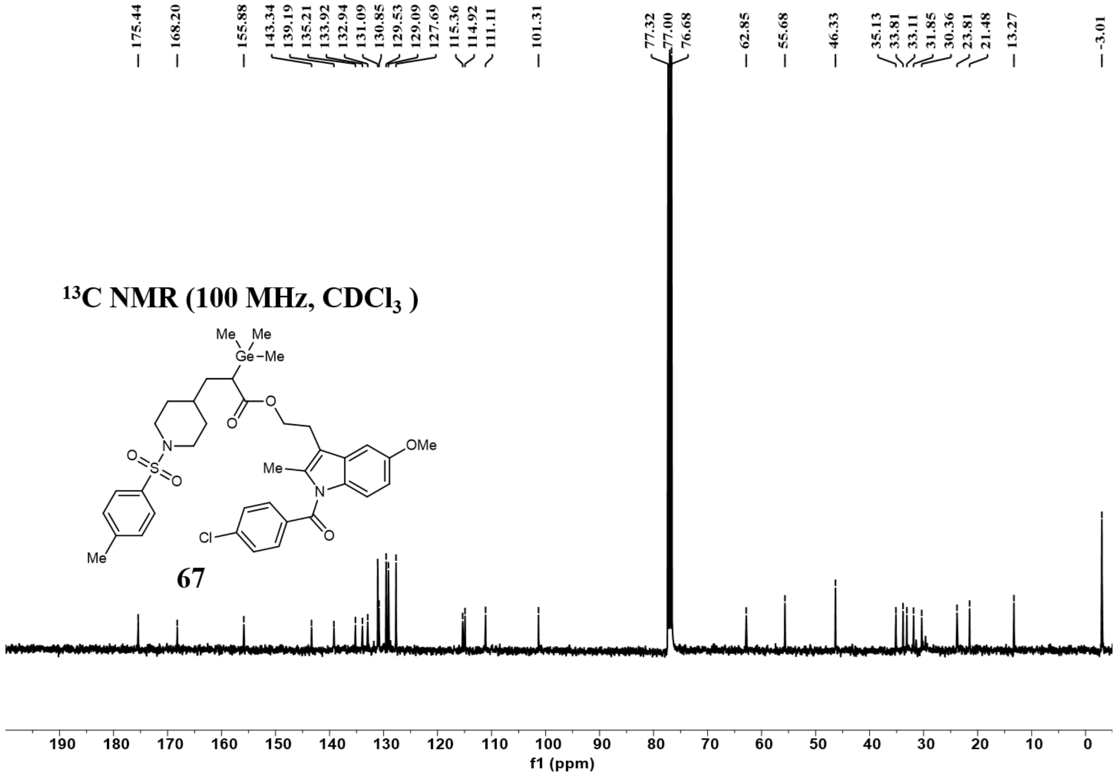

Supplementary Fig. 151. ^{13}C NMR of compound 67. The sample has been recorded in 100 MHz, $CDCl_3$ at 25 ºC.

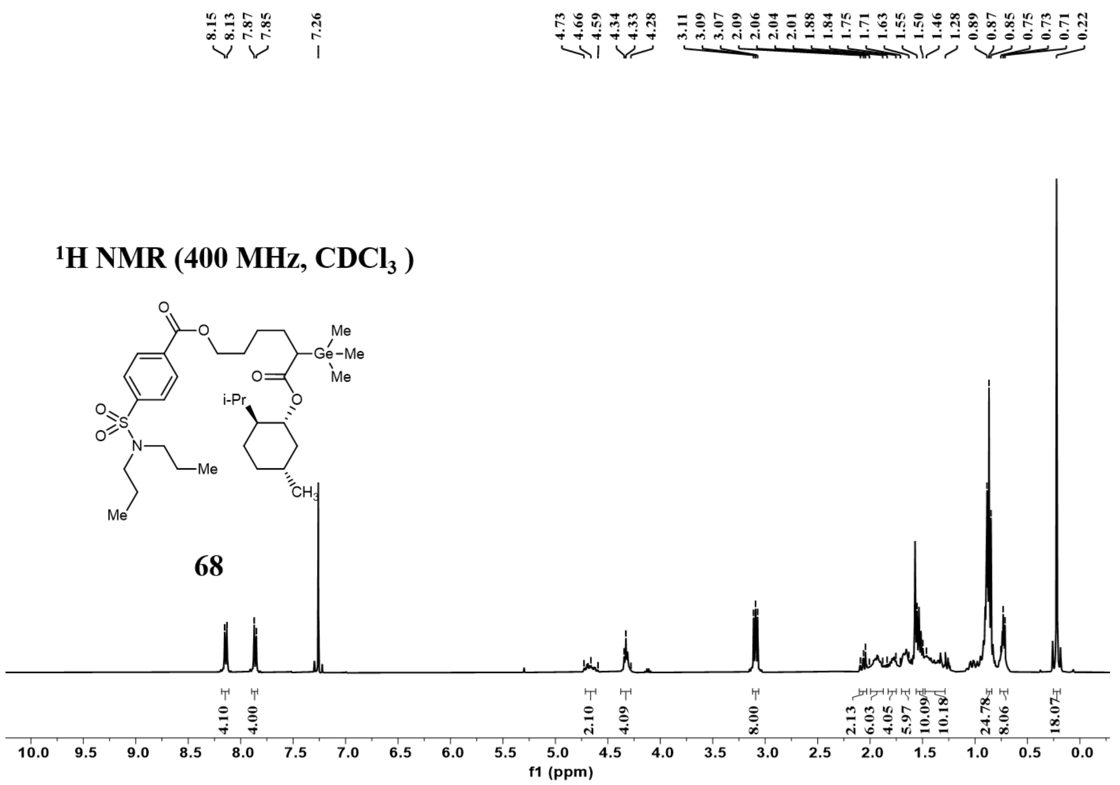

Supplementary Fig. 152. ^{1}H NMR of compound 68. The sample has been recorded in 400 MHz, $CDCl_3$ at 25 ºC

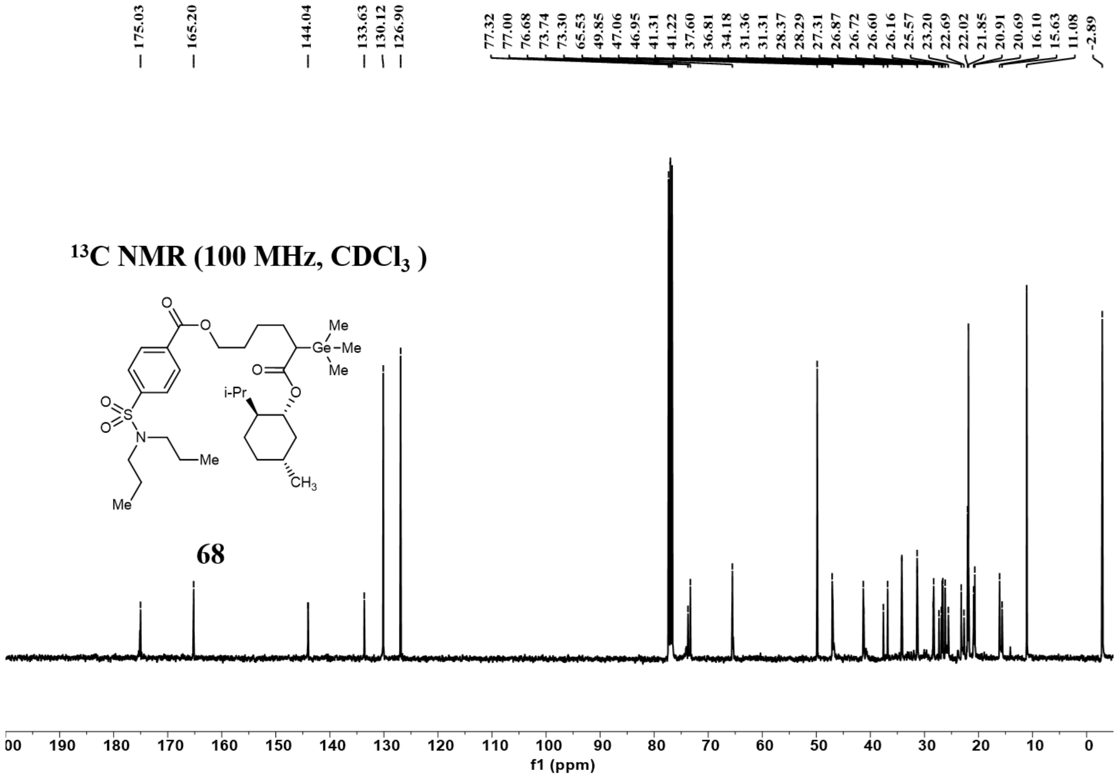

Supplementary Fig. 153. ^{13}C NMR of compound 68. The sample has been recorded in 100 MHz, $CDCl_3$ at 25 ºC.

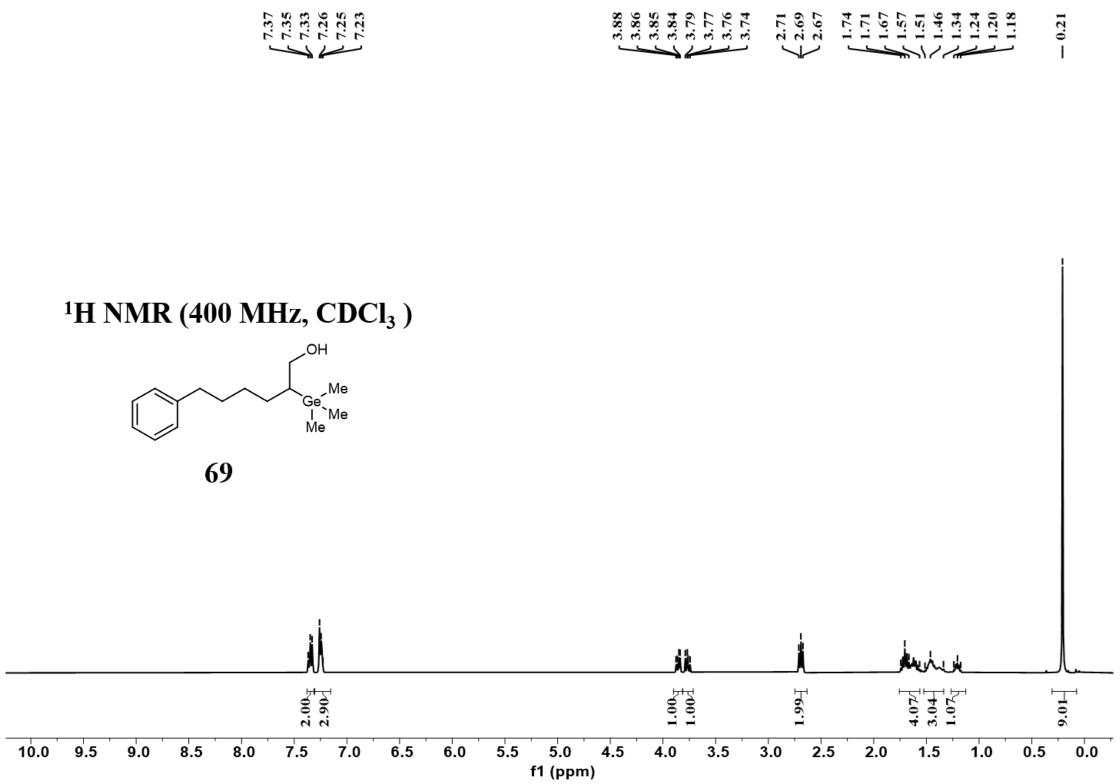

Supplementary Fig. 154. ^{1}H NMR of compound 69. The sample has been recorded in 400 MHz, $CDCl_3$ at 25 ºC

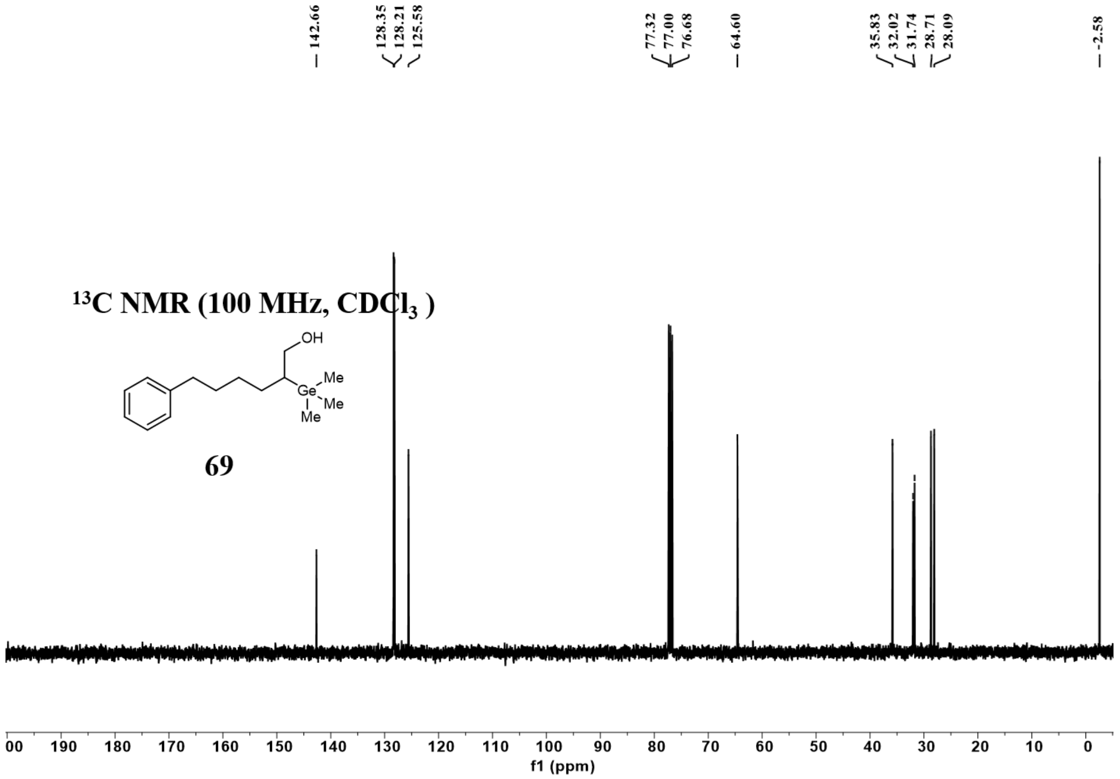

Supplementary Fig. 155. ^{13}C NMR of compound 69. The sample has been recorded in 100 MHz, $CDCl_3$ at 25 ºC.

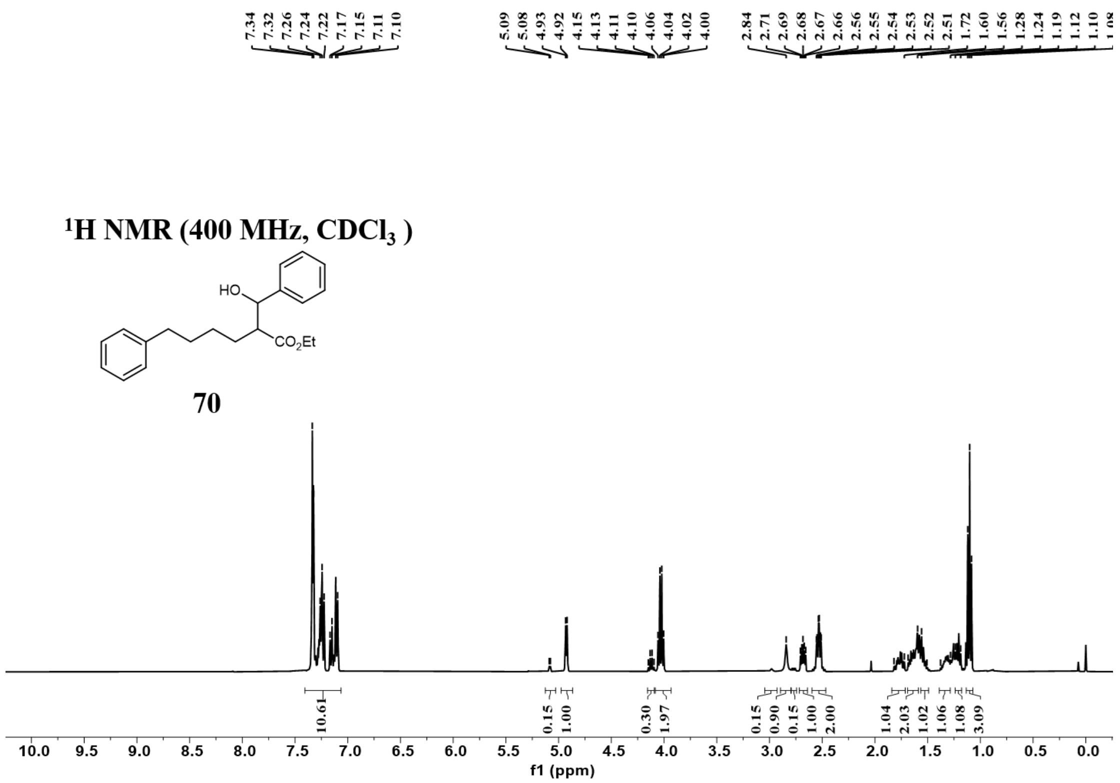

Supplementary Fig. 156. ¹H NMR of compound 70. The sample has been recorded in 400 MHz, $CDCl_3$ at 25 ºC

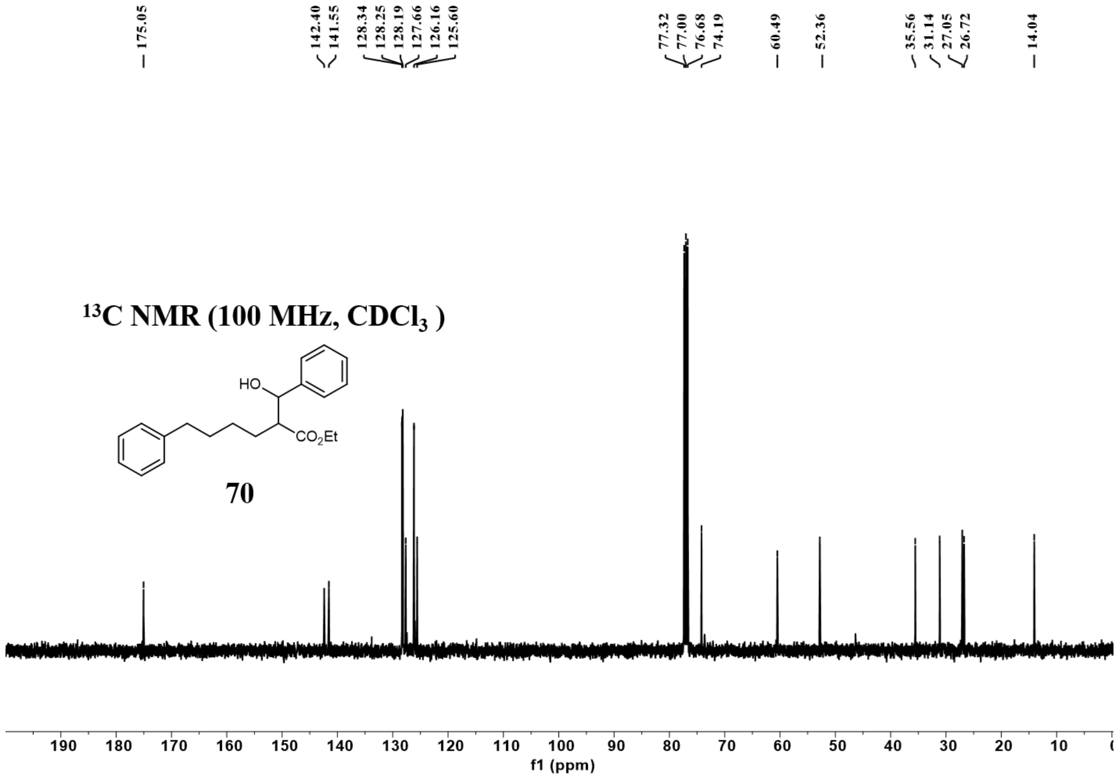

Supplementary Fig. 157. ¹³C NMR of compound 70. The sample has been recorded in 100 MHz, $CDCl_3$ at 25 ºC.

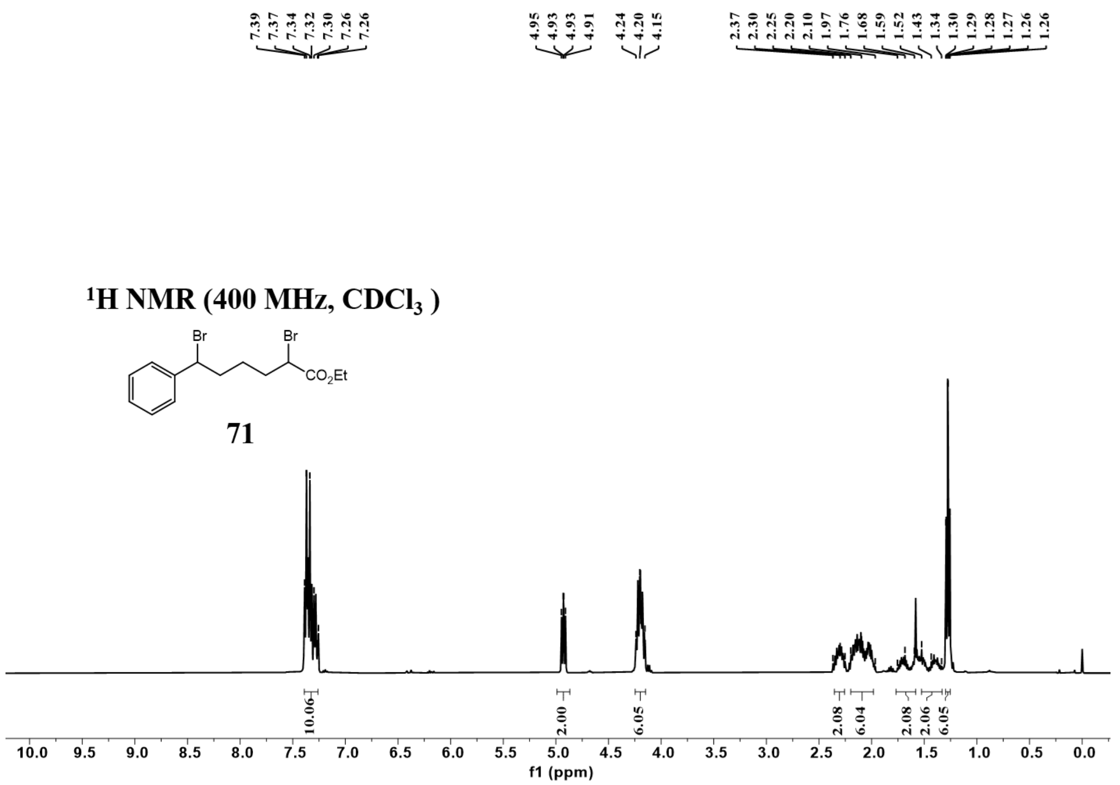

Supplementary Fig. 158. ^{1}H NMR of compound 71. The sample has been recorded in 400 MHz, $CDCl_3$ at 25 ºC

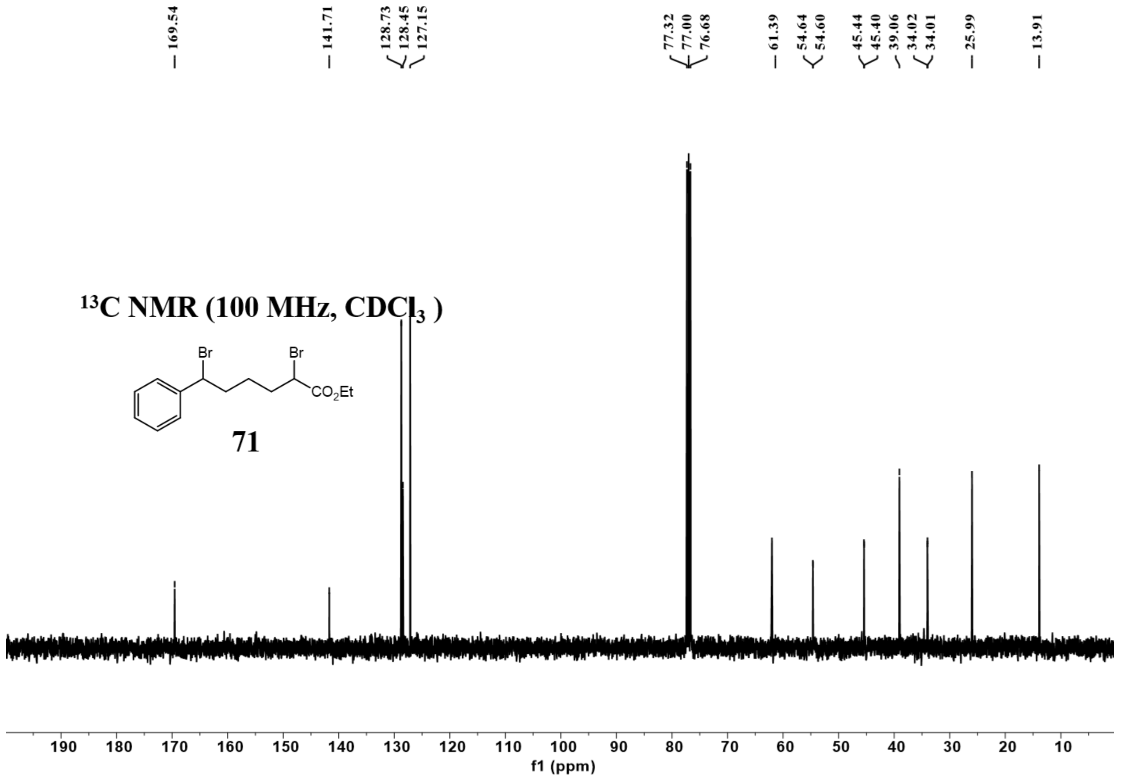

Supplementary Fig. 159. ^{13}C NMR of compound 71. The sample has been recorded in 100 MHz, $CDCl_3$ at 25 ºC.

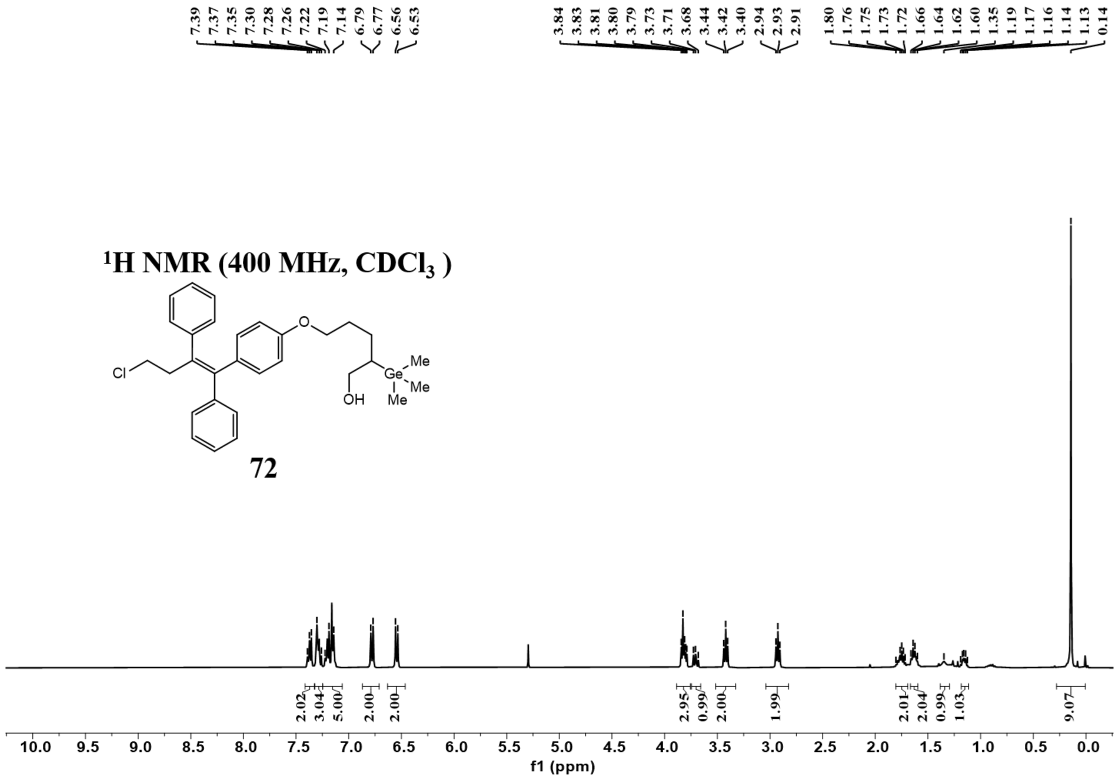

Supplementary Fig. 160. ^{1}H NMR of compound 72. The sample has been recorded in 400 MHz, $CDCl_3$ at 25 ºC

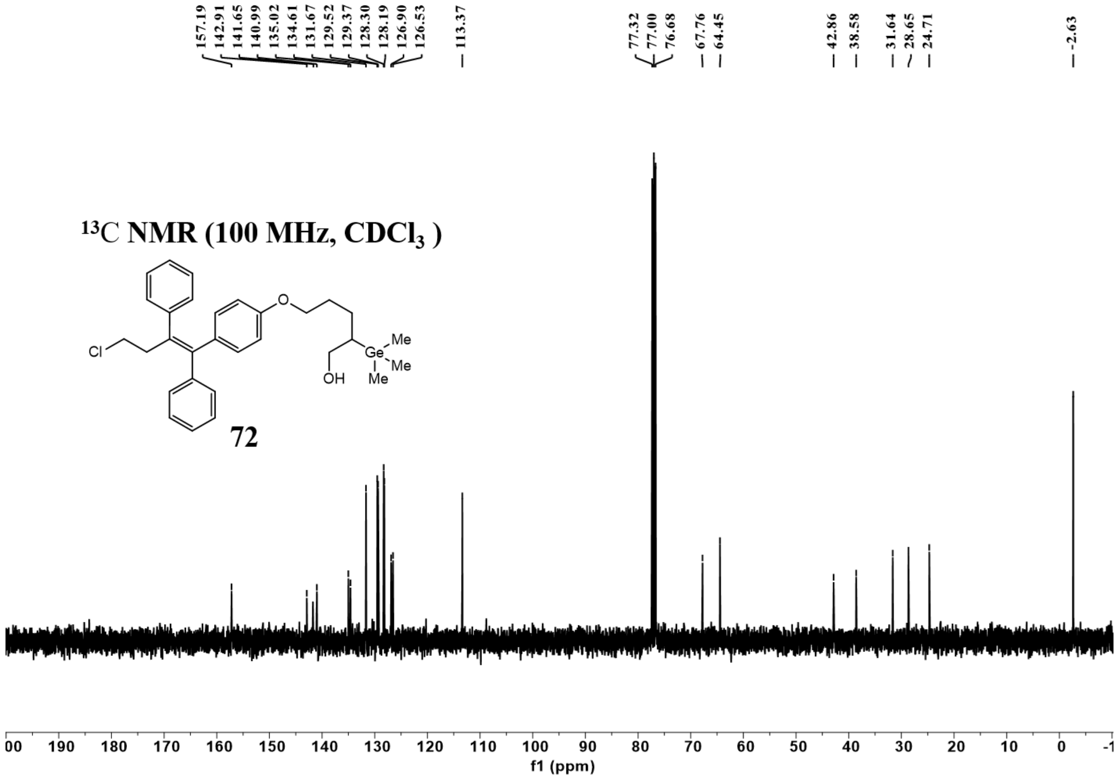

Supplementary Fig. 160. ^{13}C NMR of compound 72. The sample has been recorded in 400 MHz, $CDCl_3$ at 25 ºC

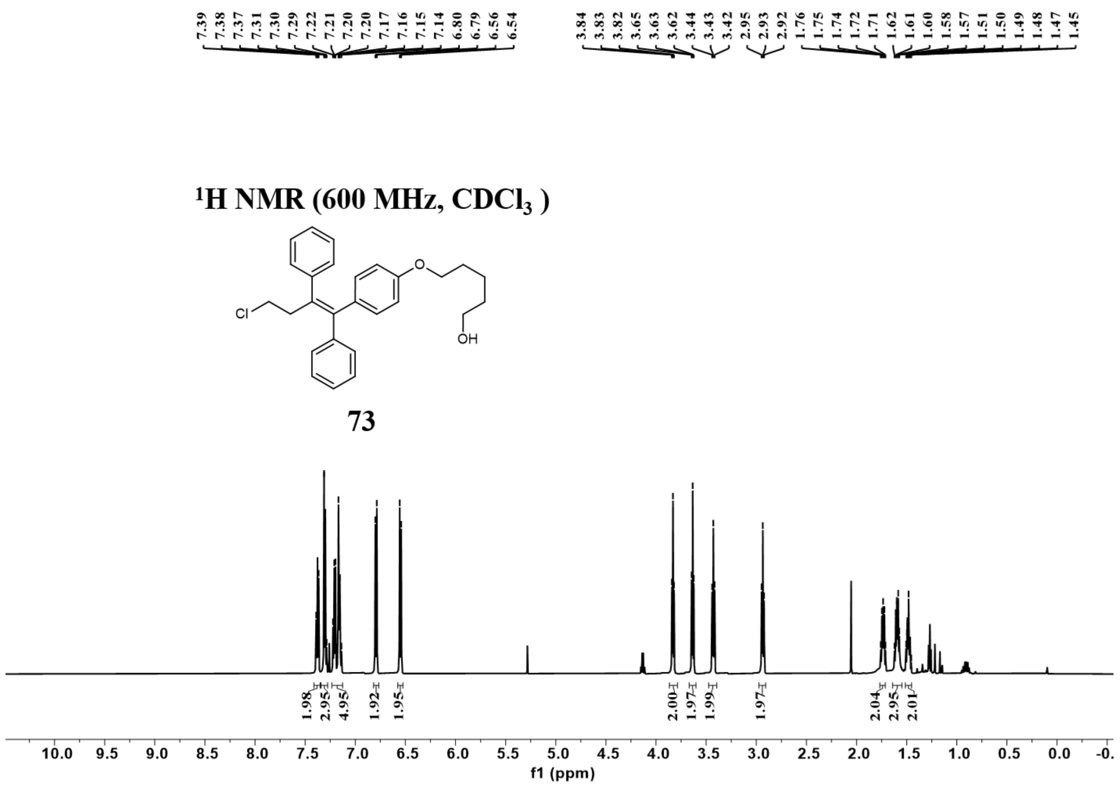

Supplementary Fig. 161. ^{1}H NMR of compound 73. The sample has been recorded in 600 MHz, $CDCl_3$ at 25 ºC

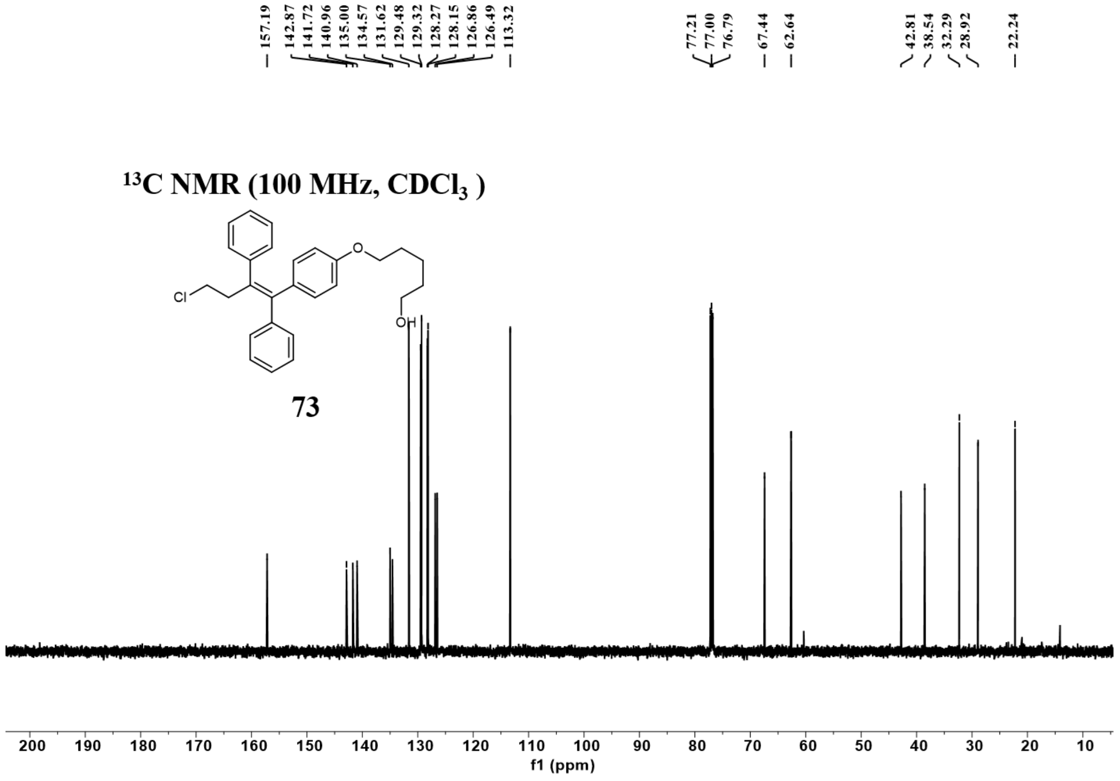

Supplementary Fig. 162. ^{13}C NMR of compound 73. The sample has been recorded in 400 MHz, $CDCl_3$ at 25 ºC

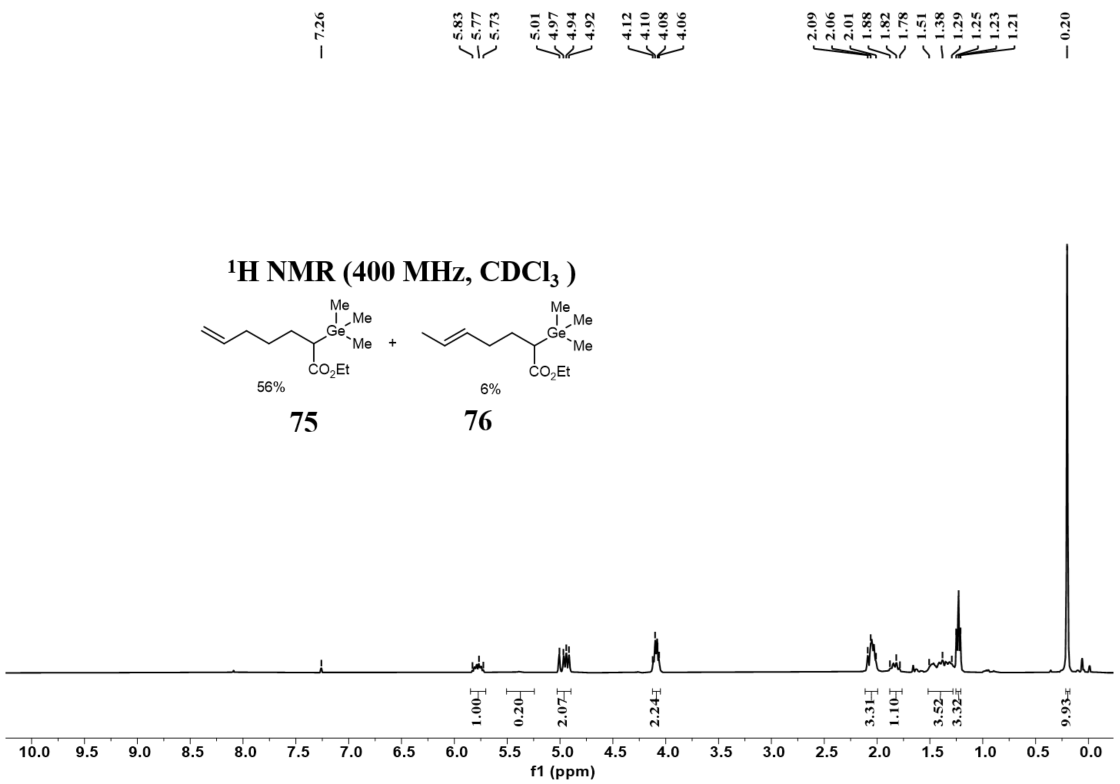

Supplementary Fig. 163. ^{1}H NMR of compound 75, 76. The sample has been recorded in 400 MHz, $CDCl_3$ at 25 ºC

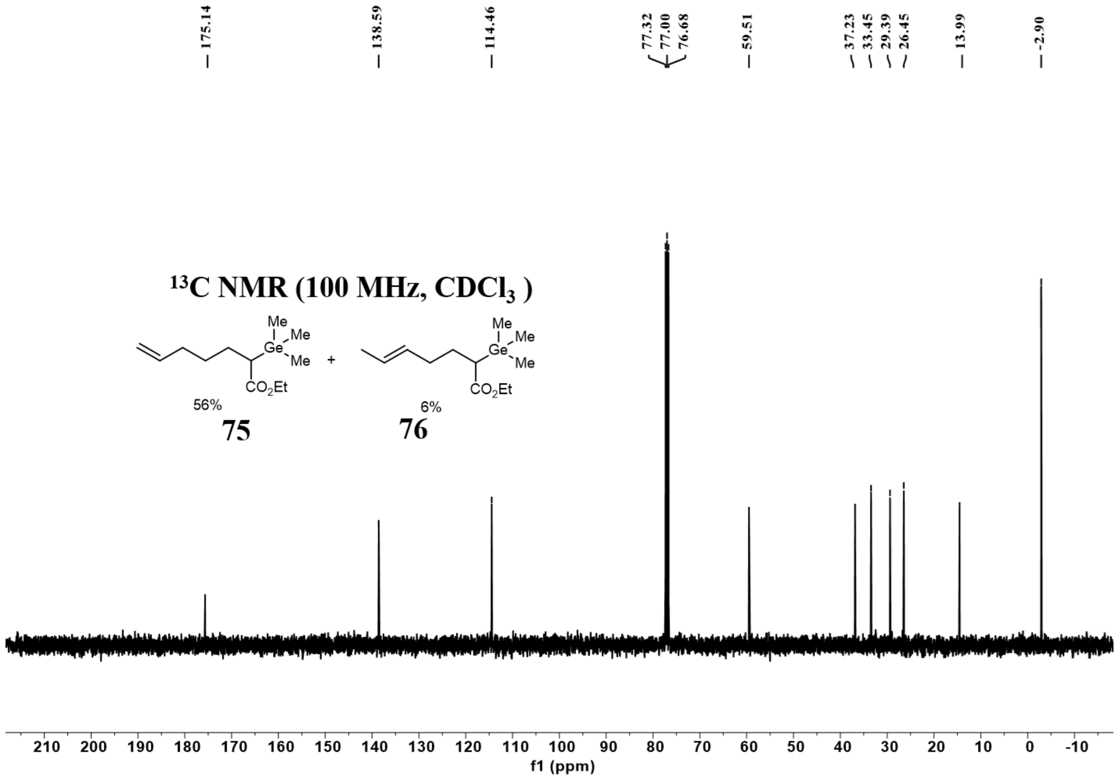

Supplementary Fig. 164. ^{13}C NMR of compound 75, 76. The sample has been recorded in 100 MHz, $CDCl_3$ at 25 ºC.

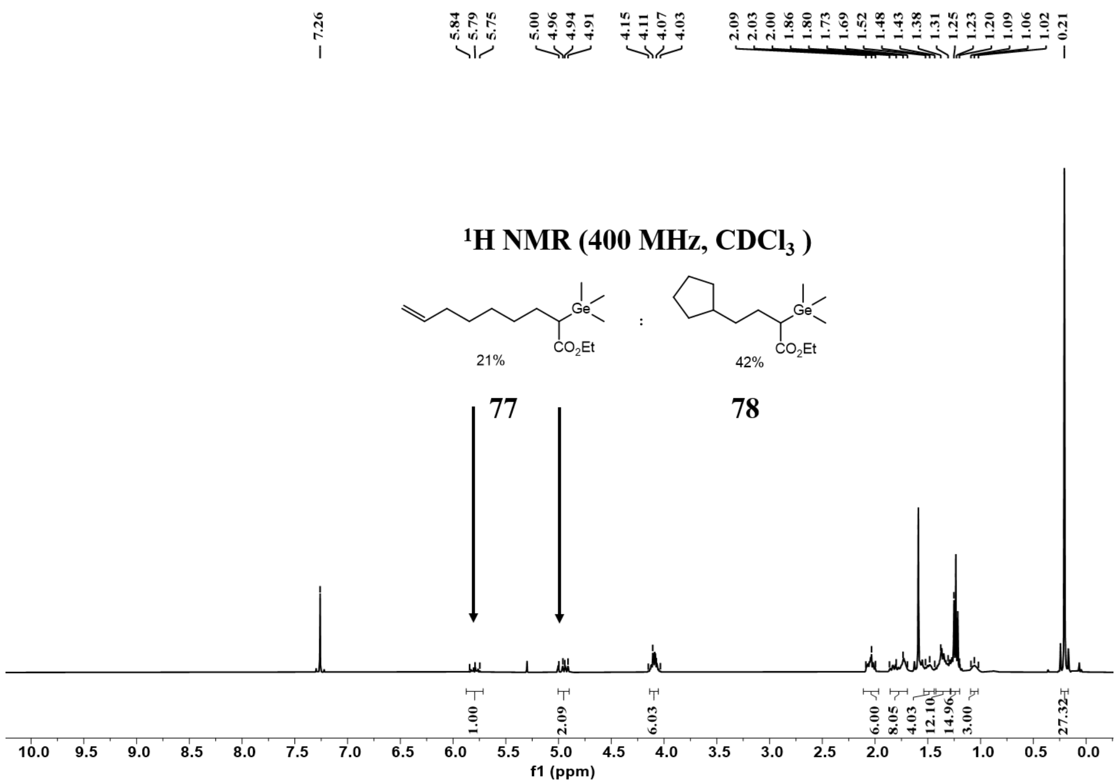

Supplementary Fig. 165. ^{1}H NMR of compound 77, 78. The sample has been recorded in 400 MHz, $CDCl_3$ at 25 ºC

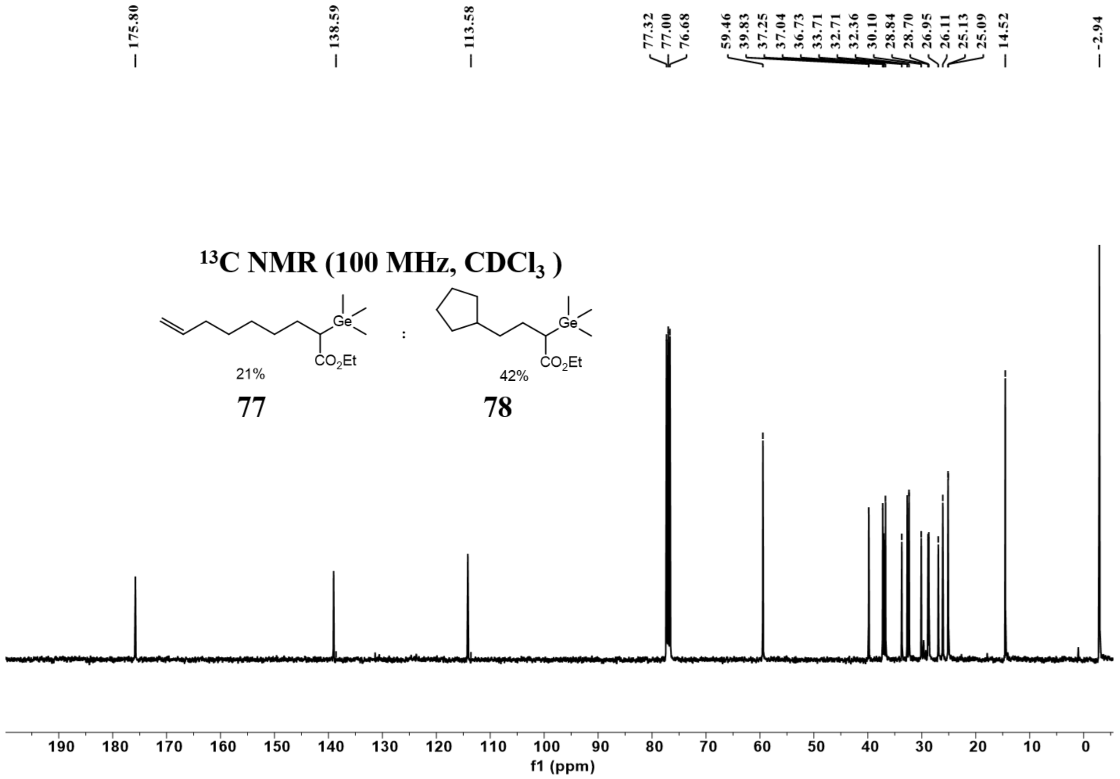

Supplementary Fig. 166. ^{13}C NMR of compound 77, 78. The sample has been recorded in 100 MHz, $CDCl_3$ at 25 ºC.

4. Supplementary References

[1] S. Yang, M. Chen, P. Tang, *Angew. Chem. Int. Ed.* **2019**, *58*, 7840–7844.

[2] J. Sun, Y. Zhou, R. Gu, X. Li, A. Liu, X. Zhang, *Nat Commun* **2022**, *13*, 7093.

[3] J. Yang, H. Song, X. Xiao, J. Wang, Y. Qin, *Org. Lett.* **2006**, *8*, 2187–2190.

[4] D. Barman, M. Annadhasan, R. Chandrasekar, P. K. Iyer, *Chem. Sci.* **2022**, *13*, 9004–9015.

[5] G. R. Pettit, N. Melody, J.-C. Chapuis, *J. Nat. Prod.* **2017**, *80*, 692–698.

[6] C.-T. Yang, Z.-Q. Zhang, J. Liang, J.-H. Liu, X.-Y. Lu, H.-H. Chen, L. Liu, *J. Am. Chem. Soc.* **2012**, *134*, 11124–11127.

[7] B. Zhao, R. Shang, G.-Z. Wang, S. Wang, H. Chen, Y. Fu, *ACS Catal.* **2020**, *10*, 1334–1343.

[8] G.-Z. Wang, R. Shang, W.-M. Cheng, Y. Fu, *J. Am. Chem. Soc.* **2017**, *139*, 18307–18312.

[9] J. Wang, Y. Gong, D. Sun, H. Gong, *Org. Chem. Front.* **2021**, *8*, 2944–2948.

[10] H. Zhao, C. Lu, S. Herbert, W. Zhang, Q. Shen, *J. Org. Chem.* **2021**, *86*, 2854–2865.

[11] G. Xiang, J. Yang, Z. Xu, W. Shi, Z. Luo, *Zhongguo Yaowu Huaxue Zazhi* **2006**, *16*, 135–139.

[12] V. Arango, J. J. Domínguez, W. Cardona, S. M. Robledo, D. L. Muñoz, B. Figadere, J. Sáez, *Med Chem Res* **2012**, *21*, 3445–3454.

[13] H. Chen, M. Farizyan, F. Ghiringhelli, M. Gemmeren, *Angew. Chem. Int. Ed.* **2020**, *59*, 12213–12220.

[14] X. Li, W. Si, Z. Liu, H. Qian, T. Wang, S. Leng, J. Sun, Y. Jiao, X. Zhang, *Org. Lett.* **2022**, *24*, 4070–4074.
